# Deciphering Antitumor Mechanism of Pien Tze Huang in Mice of Hepatocellular Carcinoma Based on Proteomics

**DOI:** 10.1155/2020/4876251

**Published:** 2020-12-03

**Authors:** Dancai Fan, Chang Liu, Li Li, Cheng Lu, Ning Zhao, Jun Shu, Xiaojuan He, Aiping Lu

**Affiliations:** ^1^The Second Clinical College of Guangzhou University of Chinese Medicine, Guangzhou 510006, China; ^2^Institute of Basic Research in Clinical Medicine, China Academy of Chinese Medical Sciences, Beijing 100700, China; ^3^Law Sau Fai Institute for Advancing Translational Medicine in Bone and Joint Diseases, School of Chinese Medicine, Hong Kong Baptist University, Hong Kong, SAR, China; ^4^Sino-America Chinmedomics Technology Collaboration Center, National TCM Key Laboratory of Serum Pharmacochemistry, Laboratory of Metabolomics, Department of Pharmaceutical Analysis, Heilongjiang University of Chinese Medicine, Harbin 150040, China; ^5^Institute of Clinical Medical Science, China-Japan Friendship Hospital, Beijing 100029, China; ^6^Academy of Integrative Medicine, Shanghai University of Traditional Chinese Medicine, Shanghai 201203, China

## Abstract

The Chinese formula Pien Tze Huang (PZH) has been used to treat hepatocellular carcinoma (HCC) and showed positive clinical effects. However, the antitumor mechanism of PZH in HCC remains unclear. In this study, HCC xenograft Balb/c mice were treated with PZH; then, proteomics detection and Ingenuity Pathway Analysis (IPA) were used to analyze the differentiated phosphorylated proteins in tumor tissues. The results indicated that PZH could inhibit tumor weight by 50.76%. Eighty-four upregulated and 11 downregulated phosphorylated proteins were identified in PZH-treated mice. Twenty signaling pathways were associated with inflammation (including the IL-6 and TNFR1/2 pathways), cancer growth (including the p53 and FAK pathways), and the cell cycle (including the G2/M and G1/S checkpoint regulation pathways). Moreover, TNF-*α*, IL-6, and several typical differentially expressed phosphorylated proteins (such as p-CCNB1, p-FOXO3, and p-STAT3) in tumor tissues, tumor cell viability, and cell cycle arrest assay *in vitro* further verify the results of IPA. These results revealed that PZH achieved antitumor activity in HCC; the underlying mechanisms of which were mainly through regulating the inflammation-associated cytokine secretion, cancer growth pathways, and induction of G2/M arrest. These data provided the potential molecular basis for PZH to act as a therapeutic drug or a supplement to chemotherapy drugs for human HCC in the future.

## 1. Introduction

Liver cancer, the fourth leading cause of cancer death worldwide, is one of the most common cancers in China [1, 2]. It is estimated to be responsible for more than 700,000 deaths in one year; the age-standardized 5-year relative survival rate of liver cancer is only 10.1% [3]. The most predominant type of liver cancer is hepatocellular carcinoma (HCC) [4]. Although there are various risk factors for inducing HCC, including viral infections, alcohol abuse, autoimmune hepatitis, diabetes mellitus, and obesity, these factors, especially hepatitis, point to induce chronic inflammation or even injury in the liver [1]. The mechanism of HCC has been shown to involve several pathways mainly related to cancer growth in HCC progression. In this context, previous studies have reported that inactivation of the tumor suppressor p53 pathway and alterations in the cell cycle are major defects in HCC [4–6]. Recently, increasing research has focused on inflammation, especially chronic inflammation and cytokines, in HCC. In the etiology and pathogenesis of HCC, persistent inflammation has been proven to exacerbate HCC, and most HCC cases arise in the context of liver injury and/or inflammation by accelerating fibrosis and cirrhosis, pushing the progression of HCC [5, 7]. It follows that the pathological progression of HCC can be characterized as chronic inflammation progressing to liver fibrosis and developing into HCC [4]. Some expression differences in cytokine secretion pathways associated with chronic inflammation have been recognized in HCC, such as nuclear factor-kappaB (NF-*κ*B), Akt, and mitogen-activated protein kinase (MAPK). Some preclinical animal studies have shown that inflammatory mediators such as IL-6 and TNF-*α* can be treated as potential targets for achieving effective therapy [8–10].

As a well-known Chinese formula, Pien Tze Huang (PZH) has been used worldwide, especially in Southeast and Northeast Asia. It was originally prescribed during the Chinese Ming Dynasty and mainly consists of *Panax notoginseng*, *Moschus*, *Calculus Bovis*, and *Snake Gall* [8]. It has been widely used to alleviate inflammation-related diseases, and animal experiments demonstrated that PZH displayed extensive regulation of inflammatory conditions by targeting multiple cellular processes, such as cytokines and pathways [11–14]. Currently, studies have reported that PZH can treat various precancers and cancers, mainly HCC and colon carcinoma [14–17]. However, the biological mechanism of its antitumor effects remains to be elucidated.

Protein phosphorylation is important for protein function and can affect molecular biological activity. The levels of altered protein phosphorylation are associated with regulating cellular signaling pathway and further recognizing the response to environmental stimuli. To distinguish among these changes, the relative abundance of each phosphorylation site can be compared with that of its parent protein to identify differential phosphorylation [18]. Proteomics can be used to provide a convenient way of understanding the phosphorylation sites in proteins and the associated distribution in relative abundance between groups [19]. Cancer is predominantly studied at the genetic level, but proteomics has long been seen as a promising complementary technology that could enable insight into the disease at the protein level [20]. Therefore, in this study, we examined the antitumor effects of PZH on a mouse model of HCC and further investigated the molecular targets by using proteomics to identify the protein phosphorylation sites and the network of key molecular pathways to deepen the understanding of the underlying mechanisms of PZH on HCC.

## 2. Materials and Methods

### 2.1. Cell

H22 murine hepatoma cells were obtained from the China Center for Type Culture Collection (CCTCC) and were cultured in RPMI 1640 medium supplemented with 10% FBS (Gibco, Thermo Fisher Scientific, USA) and 1% penicillin/streptomycin in 5% CO_2_ atmosphere at 37°C.

### 2.2. Animal

The four-week-old male Balb/c mice were supplied by Beijing Huafukang Bioscience Company (certification NO. SCXK (JING) 2014-0004). The mice were fed with food and water *ad libitum* and then were allowed to acclimatize themselves for one week before the initiation of the experiment. All the experiments on animals were performed under the Guidelines for the Care and Use of Laboratory Animals. All protocols used here received approval from the Research Ethics Committee of Institute of Basic Theory of Chinese Medicine, China Academy of Chinese Medical Sciences.

### 2.3. Implantation of Tumors and Treatment

PZH (China Food and Drug Administration approval No. Z35020243; Zhangzhou, China) was produced and authenticated by Zhangzhou Pien Tze Huang Pharmaceutical Co., Ltd. The drug samples were characterized by HPLC-MS/TOF as our previous publication [11]. Sorafenib (CAS No. 284461-73-0) was purchased from Wuhan Yuancheng Gongchuang Technology Co., Ltd. For treatment, we diluted the PZH powder in normal saline on the required concentration and administered the drug solution to mice by gavage every day. H22 cells (3 × 10^6^) were injected subcutaneously into the right flank region of each mouse. When tumor volumes reached 80-100 mm^3^, the mice were randomly divided into three groups with six mice in each group. In addition to six mice as the model group, the other mice were treated with 0.121 g/kg of Sorafenib (Sora) as the positive control group (equal to adult dosage) or 0.234 g/kg of PZH (equal to adult dosage) by gavage administration one time per day for 2 weeks. The mice were sacrificed after the day of the last treatment. At the termination of the experiments, the xenograft tumors were isolated and weighed. Tumor volume was measured with Vernier calipers and calculated. The tumor weight inhibition rates (TWI %) were calculated according to the following formula: TWI% = (1 − tumor weight of treatment group/tumor weight of model group) × 100% [21].

### 2.4. Proteomics Analysis

Proteomics was used to analyze the tumor tissues of the model group, Sora group, and PZH group with Phospho Explorer antibody microarray (PEX100) provided by Wayen Biotechnologies Inc. (Shanghai, China). A total of 584 phosphorylation sites were fixed on the PEX 100 to identify the key protein antibodies in multiple signaling pathways. The microarrays were scanned using a Sure Scan Dx Microarray Scanner for chip imaging, and the GenePix Pro 6.0 software was used to read the original data. In the comparison of different samples, the Phospho Ratio of a specific site in the treatment groups (Sora group and PZH group) was divided by the Phospho Ratio of the same site in the model group to obtain the intergroup phosphorylation level modulation ratios of all phosphorylated sites. The formula for calculating was as follows: Phospho Ratio = Phospho Ratio_Exp._/Phospho Radio_Con_. According to the experimental results and articles, we set 2 as the fold change threshold to obtain the modulation of phosphorylated sites between groups.

### 2.5. Ingenuity Pathway Analysis (IPA)

Differentially expressed phosphorylated proteins identified on the PEX 100 were analyzed by Ingenuity Pathway Analysis (IPA, Ingenuity Systems; http://www.ingenuity.com). The ratios as fold changes were input into the IPA, and networks were then algorithmically generated based on protein-protein interactions. Referring to online published articles, IPA established the pathway networks of identified proteins associated with the cancer pathway, cytokine pathway, and cell cycle pathway. To ensure that the results were universal and definitive, we excluded the pathways unrelated to cancer and set the threshold as -log (*p* value) >3.00.

### 2.6. Cytokine Measurement

Frozen tumor tissue samples were weighed and homogenized (100 mg tissue per mL of homogenization buffer). The homogenate was centrifugated, and the supernatant was collected for analysis. Then, 50 *μ*L supernatant was added to the mouse IL-6 and TNF-*α* ELISA kit plates (Dakewe Biotech Co., Ltd., Shenzhen, China), and the instructions were followed to measure the OD values and calculate the level of expression based on the standard curve.

### 2.7. Cell Viability Assay

Cell viability was measured by a cell counting kit-8 assay (CCK-8) that was obtained from Dojindo (Dojindo Molecular Technologies, Japan) according to the instructions. Briefly, H22 cells were seeded in 96-well plates at 0.5 × 10^4^ cells per well in 0.1 mL medium and cultured for 24 h. The cells were treated with different concentrations of PZH solution (0, 0.10, 0.25, 0.50, 0.75, and 1.00 mg/mL) for 24, 48, and 72 h. After treatment, the kit reagent WST-8 was added to the wells, and the cells were protected from light and incubated for 1 h at 37°C. Then, the optical density at 450 nm was measured using a microplate reader.

### 2.8. Cell Cycle Analysis

The cell cycle was tested using a Cycle Test DNA reagent kit (BD Biosciences, San Jose, CA, USA) by fluorescence-activated cell sorting (FACS). H22 cells were seeded on six-well plates at 1 × 10^6^ cells per well and cultured for 24 h. Then, the cells were treated with various concentrations (0, 0.10, 0.25, 0.50, 0.75, and 1.00 mg/mL) of PZH for 48 h, and the cell suspensions were adjusted to a concentration of 5 × 10^5^ cells/mL and fixed in 70% ethanol at 4°C overnight. The fixed cells were washed twice with cold PBS and then incubated for 30 min with RNase (8 *μ*g/mL) and PI (10 *μ*g/mL). The fluorescent signal was measured and analyzed on a BD Accuri C6 flow cytometer.

### 2.9. Western Blot Analysis

The typical protein expressions (p-FOXO3, p-STAT3, and p-CCNB1) in the proteomics database were further measured by Western blot. After treatment with PZH, the tumor tissue was isolated from mice and then made of homogenate to extract proteins. Total protein concentrations were determined by the Pierce BCA Protein Assay Kit (Thermo Scientific, Rockford, IL, USA). Aliquots of 30 *μ*g of proteins were separated by SDS-PAGE gel and transferred onto polyvinylidene fluoride (PVDF) membranes (Millipore, Bedford, MA, USA). After blocking with 5% BSA for 1 hour, membranes were incubated with primary antibodies overnight at 4°C and were incubated with secondary antibodies for 1 hour at room temperature. Then, developed the blot in Western blotting by X-ray, and the image analysis was based on the software Image-J.

### 2.10. Statistical Analysis

Data were analyzed using Graph Pad Prism 7.0. All data were expressed as mean ± SD. Student's *t*-test or one-way analysis of variance (ANOVA) was used for statistical analysis. *p* < 0.05 was considered to be statistically significant.

## 3. Results

### 3.1. Suppression of Xenograft Tumor Growth *In Vivo*

**Figure 1:**
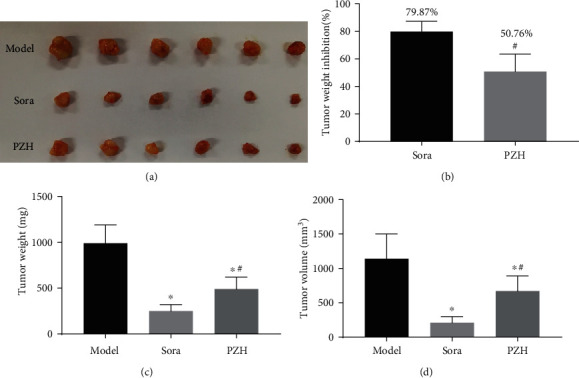
PZH inhibits xenograft tumor growth *in vivo*. Balb/c mice were injected with 3 × 10^6^ H22 cells in subcutaneously into the right flank region, when tumors had grown to the volume of 80-100 mm^3^, PZH (0.234 g/kg/d) or Sorafenib (0.121 g/kg/d), or normal saline was administrated for 14 days (once per day), and then, the tumor xenografts were excised completely from tissues. (a) A macroscopic view of the xenografted tumor after treatments from the groups indicated. (b) Analysis of tumor weight inhibition rate. (c) Analysis of tumor weight. (d) Analysis of tumor volume. ^∗^*p* < 0.05 vs. model group, ^#^*p* < 0.05 vs. Sora group (*n* = 6).

One week after injection of H22 cells, Balb/c mice generated significant xenograft tumors. In the Sora and PZH groups, the tumor weight inhibition rates were 79.87% and 50.76%, respectively. Treatment with Sora and PZH resulted in decreases in xenograft tumor weight and volume (Figure 1).

### 3.2. Changes of Phosphorylated Proteins in Tissues

**Figure 2:**
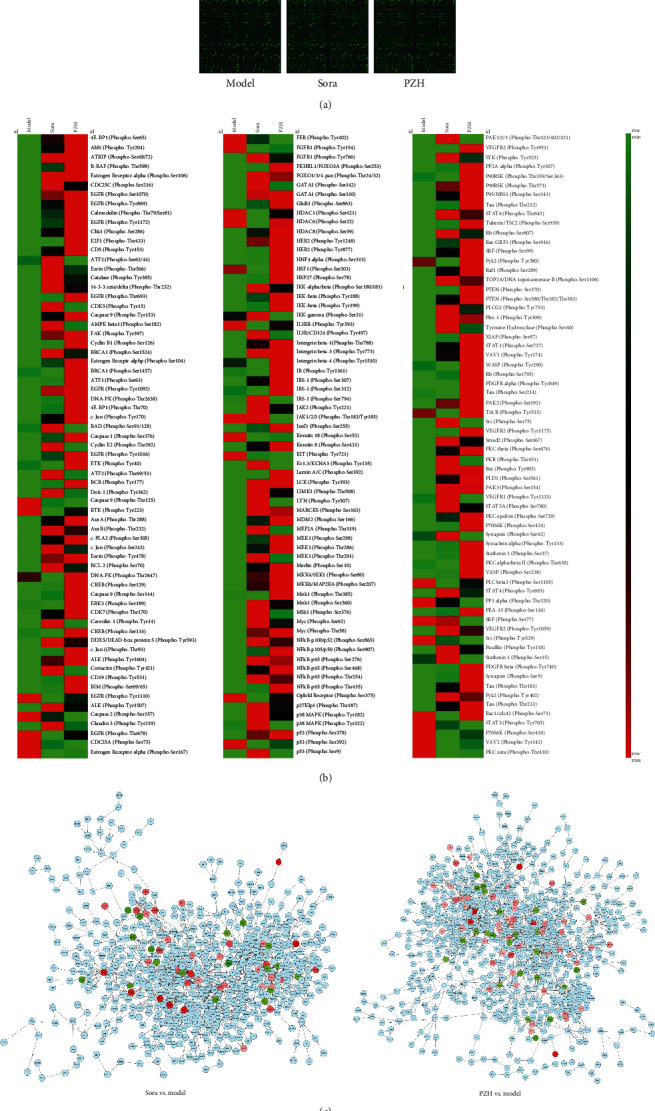
The change of phosphorylated proteins in tumor tissues after PZH treatment. (a) Scanned pictures of PEX100. The microarrays were scanned using a Sure Scan Dx Microarray Scanner, and the data quality of all sites was good. (b) The heat map was generated from normalized intensity data using the Morpheus tool (https://www.morpheusdata.com/). The heat map represented fold change in phosphorylation status. Each cell in the heat map showed a ratio of phosphorylated to unphosphorylated proteins. Red indicated upregulation while green represented downregulation in phosphorylation of signaling proteins, and the intensity of color depended on the degree of phosphorylation. In the figure, we showed the heat map as three parts with the same groups to be more clear. (c) Protein regulation network was established by the Cytoscape tool (https://cytoscape.org/) to demonstrate the interaction of phosphorylated proteins, and the central nodes were also shown visually in the networks. The color scheme was that red meant upregulation, green meant downregulation, and light blue meant no change.

We used the PEX100 to test the levels of phosphorylated proteins in the tumor tissues of the mice from the model group, Sora group, and PZH group (Supplementary Table 1-3). The scanned picture showed a good response of all sites in the microarrays, and the data quality was credible (Figure 2(a)).

To confirm the scope of selection, a fold change >2 was considered a significant change. Among the 584 detected phosphorylated protein sites, 60 were upregulated and 19 were downregulated in the tumor tissue with Sora treatment, and 84 were upregulated and 11 were downregulated in the tumor tissue with PZH treatment (Figure 2(b), Supplementary Table 4-5). The protein interaction network based on the changes in phosphorylated proteins showed the complex association among different proteins, and some key proteins that have been studied previously in the network were identified, such as p53^Ser378^, NF*κ*B-p65^Ser468^, and Bcl-2^Ser70^ (Figure 2(c)).

### 3.3. Pathways and Molecular Networks Involved in PZH Treatment

**Figure 3:**
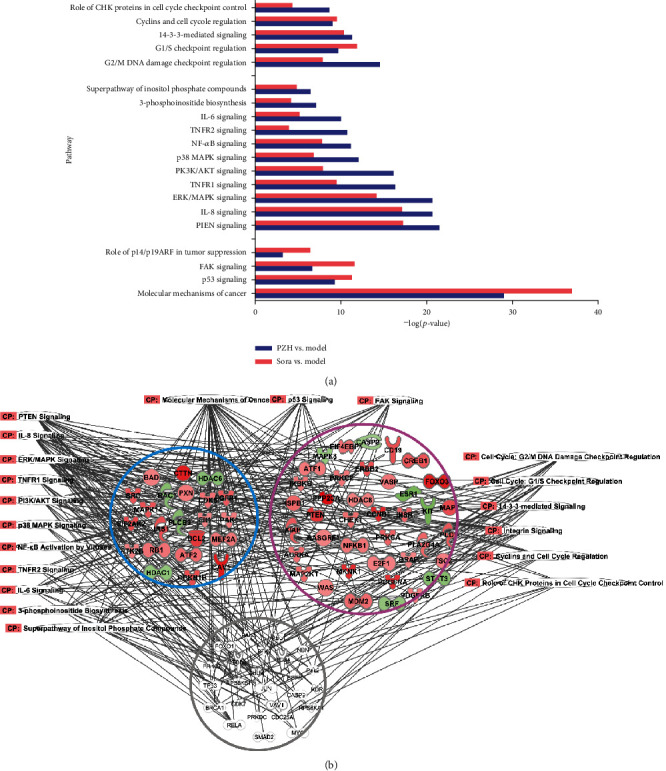
Comparison of involved pathways between PZH and Sora treatment. (a) The -log (*p* value) value of involved pathways in PZH and Sora groups. (b) The molecular network involved in those pathways regulated by PZH and Sora treatment.

The cancer-related pathways were identified with IPA. In the IPA system, the threshold was -log (*p* value) >3.0, indicating a *p* value < 0.001 for a significant association. The 20 pathways that met the threshold and the contribution degree for the PZH and Sora groups were shown in Figure 3(a). Among them, 11 were inflammation-related pathways, 4 were cancer growth-related pathways, and 5 were cell cycle-related pathways. The complicated molecular networks involved in these 20 pathways were shown in Figure 3(b).

**Figure 4:**
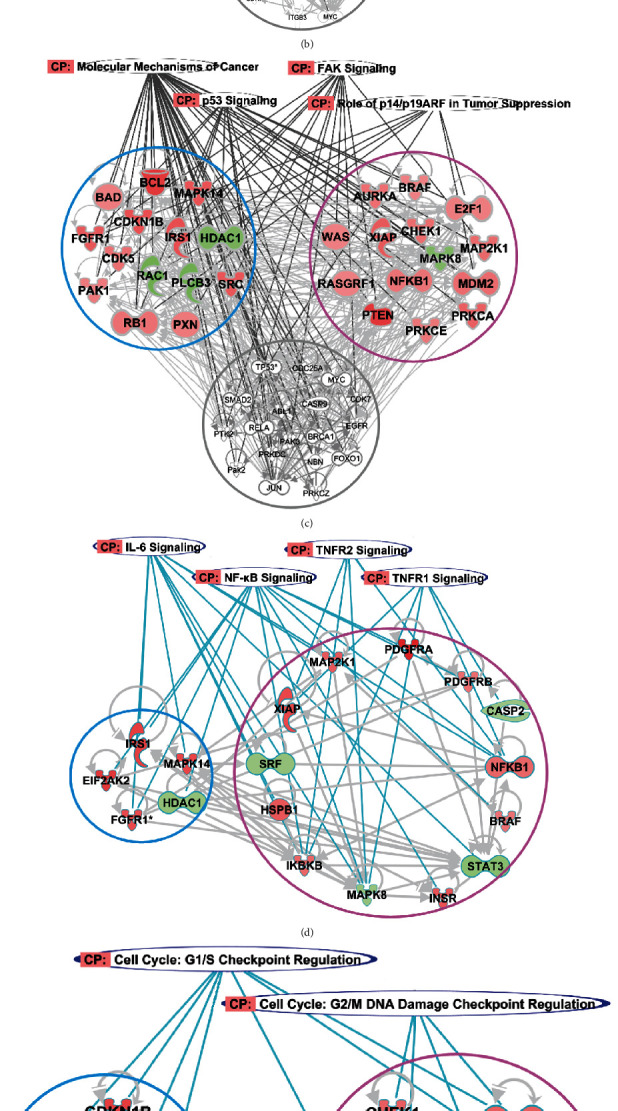
The key proteins and pathways of the molecule network involved in PZH and Sora treatment. (a) The molecular network based on the inflammation-related pathways. (b) The molecular network based on the cell cycle-related pathways. (c) The molecular network based on the cancer growth-related pathways. (d) PZH regulated more proteins enriched in IL-6, TNFR1/2, and NF-*κ*B pathways than Sora. (e) PZH regulated more proteins in G2/M phase, whereas Sora regulated more proteins in G1/S phase. (f) Sora regulated more proteins related to the activity of kinases enriched in p53 and FAK pathways. Pink cycle represented PZH group, blue cycle indicated Sora group, and gray cycle indicated both PZH group and Sora group.

To further analyze the underlying mechanism of the antitumor activity of PZH, the related molecule networks of the involved pathways were established with the IPA system.  Figure 4 showed that the complicated molecular networks involved in pathways associated with inflammation/cytokines (Figure 4(a)), cancer growth (Figure 4(b)), and the cell cycle (Figure 4(c)).

Regarding the molecular network related to inflammation pathways, 68 different phosphorylated proteins were involved. PZH singly regulated 33 phosphorylated proteins. Sora singly regulated 17 phosphorylated proteins (Figure 4(a)). Furthermore, PZH upregulated the phosphorylation of key node proteins, such as NF-*κ*B1, IKK*β*, FOXO3, XIAP, and downregulated STAT3, MAPK8, SRF, and CASP2. These proteins were mainly enriched in the IL-6 signaling pathway, TNF receptor 1 (TNFR1) signaling pathway, and TNFR2 signaling pathway (Figure 4(d)).

The cell cycle-related molecular network contained 42 different phosphorylated proteins. Of these proteins, PZH singly regulated 13 proteins, and Sora singly regulated 14 proteins (Figure 4(b)). By regulating the protein phosphorylation of CDKN1B, RB1, HDAC1, and HDAC6, Sora could increase cell cycle arrest at G1/S. In contrast, PZH introduced the cell cycle arrest at G2/M by regulating the phosphorylation of MDM2, CHEK1, AURKA, and CCNB1 (Figure 4(e)).

In the molecular network related to cancer growth pathways, 46 phosphorylated proteins were shown. Among them, 14 proteins were regulated by Sora, and 14 proteins were regulated by PZH. The two treatment groups had common key proteins, such as TP53, ABL1, and PAK2 (Figure 4(c)). While Sora upregulated more proteins associated with the activity of kinases such as PAK1, SRC, and the kinase substrate IRS1, these proteins were mainly enriched in the p53 pathway and FAK pathway (Figure 4(f)).

Interestingly, the NF-*κ*B, TNFR1, TNFR2, and IL-6 pathways related to inflammation; the p53 and FAK pathways related to cancer growth; and the G2/M checkpoint pathway related to the cell cycle were found to be more important to elucidate the mechanism of PZH and were further validated in the following experiments.

### 3.4. Validation

**Figure 5:**
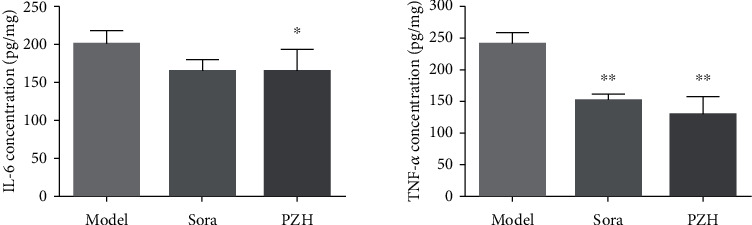
PZH decreases IL-6 and TNF-*α* in tumor tissue of HCC mice. IL-6 and TNF-*α* were examined by ELISA kits. ^∗^*p* < 0.05 vs. model group (*n* = 6).

The results of IPA analysis showed that PZH could regulate pathways associated with the production of IL-6 and TNF-*α*. The ELISA results indicated that PZH indeed decreased the levels of IL-6 and TNF-*α* in tumor tissue homogenates from HCC mice (Figure 5).

**Figure 6:**
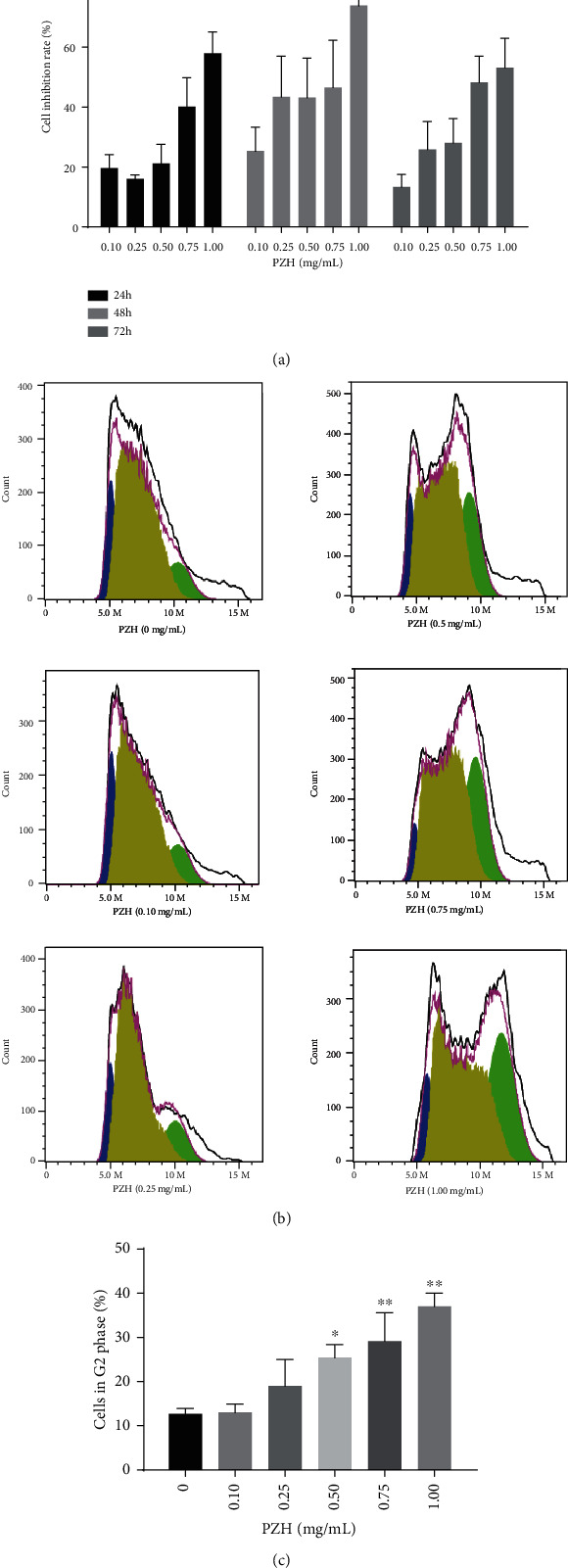
PZH inhibits H22 cells *in vitro*. (a) Analysis of cell growth inhibition rate. H22 cells were treated with different concentrations of PZH (0, 0.10, 0.25, 0.50, 0.75, and 1.00 mg/mL) for 24 h, 48 h, and 72 h, respectively. (b) Representational results of cell cycle analysis. The blue, yellow, and green colors represent the G1, S, and G2 phases, respectively. (c) Analysis of the cell percentage in G2/M phase. ^∗^*p* < 0.05, ^∗∗^*p* < 0.01 vs. 0 mg/mL group.

Cell viability assay indicated that 0.10, 0.25, 0.50, 0.75, and 1.00 mg/mL of PZH inhibited the viability of H22 cells at 24 h, 48 h, and 72 h, and the inhibition at high concentration (1.00 mg/mL) inhibited the viability of H22 cells by over 50.00%. The results showed that treatment with PZH could suppress H22 cell viability in a dosage-dependent manner (Figure 6(a)).

In the IPA results, PZH regulated pathways related to the cell cycle, especially in the G2/M checkpoint pathway. Cell cycle analysis revealed a decrease in the population of H22 cells in the S phase and an increase in the percentage of cells in the G2/M phase after treatment with five kinds of concentrations of PZH, and the response was dose-dependent (Figures 6(b) and 6(c)).

**Figure 7:**
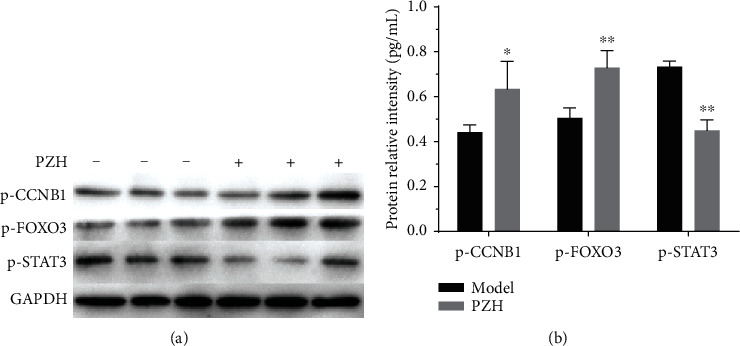
PZH upregulates the expression of p-CCNB1 and p-FOXO3 and downregulates the expression of p-STAT3 in tumor tissue of HCC mice. (a) Representative Western blot bands of p-CCNB1, p-FOXO3, and p-STAT3. (b) Analysis of relative protein levels. ^∗^*p* < 0.05, ^∗∗^*p* < 0.01 vs. model group.

Because of the high fold change and close relationship with the inflammation and G2/M cell cycle pathways, we chose three proteins (p-CCNB1, p-FOXO3, and p-STAT3) in the proteomics database to further verify. Western blot results showed that PZH could upregulate the expression of p-CCNB1 and p-FOXO3 and downregulate the expression of p-STAT3, which were coincident with the regulation from the partial result of proteomics test (Figure 7).

## 4. Discussion

HCC is regarded as an inflammation-induced cancer [22]. PZH is a famous traditional Chinese formula used in various diseases related to inflammation. Researchers have examined the contribution of PZH in some neoplastic diseases, especially colorectal cancer and liver cancer, which showed that PZH inhibited cancer cell proliferation and played an antitumor role [14, 15, 17]. In this study, we confirmed that PZH could significantly inhibit the tumor growth in an HCC mice model and cell proliferation of H22 cells *in vitro*.

Based on proteomics and IPA analysis, we found that although PZH and Sorafenib could regulate the same pathways, their action relevance was different. PZH played a stronger role in inflammation-related pathways, whereas Sorafenib focused more on cancer growth pathways. In addition, as for the cycle-related pathways, PZH was more involved in introducing cell cycle arrest at G2/M, whereas Sorafenib was more involved in promoting cell cycle arrest at G1/S. These results implied that the combination of the two drugs might have synergistic effects.

Emerging studies revealed that liver damage-mediated inflammation and carcinogenesis were closely related to the complex cross-talk among NF-*κ*B, c-Jun N-terminal kinase (JNK), and signal transducer and activator of transcription 3 (STAT3) signaling pathways, which could regulate the production of various inflammatory factors including TNF-*α* and IL-6 [22]. In addition, MAPK/p38 signaling pathway not only regulated the expression of proinflammatory cytokines but also played an important role in the activation of cell adhesion, migration, and invasion in HCC patients [23]. In this study, we found that although both PZH and Sorafenib regulated these inflammation-related pathways, but their targets were somewhat different, especially in TNFR1, TNFR2, and IL-6 pathways. PZH mainly regulated STAT3, FOXO3, IKK*β*, XIAP, and MAPK8. Among these, the STAT3 signaling pathway is a proinflammatory pathway and may be triggered by the tumor cells. FOXOs, act as a tumor suppressor, can promote antitumor activity through negatively regulating the expression of immunosuppressive proteins. In experimental studies, overexpression of FOXO3a inhibits the proliferation and invasiveness of cancer cells [24]. In this study, we showed that p-FOXO3 was upregulated whereas p-STAT3 was downregulated after PZH treatment. Interestingly, this was similar to our previous studies which have demonstrated PZH could inhibit p-STAT3 level in autoimmune encephalomyelitis rat [13].

In the cell cycle, there are some key proteins that control the triggers for the next stage. Phosphorylation can alter the activity of a protein, and the phosphorylation of a specific set of proteins at the cell cycle checkpoints can induce transition or arrest [25]. Cell cycle events in tumor cells at different stages can affect the progression and therapy of HCC [26]. Inducing cell cycle arrest can inhibit proliferation and increase apoptosis of tumor cells [27]. Our results showed that Sorafenib played a more important role in the G1/S checkpoint pathway and in proteins associated with G1/S phase. Some researchers have demonstrated that tumor cell proliferation and tumor growth inhibition could be also due to the induction of apoptosis and G2/M arrest [28, 29]. Our results demonstrated that besides G1/S arrest, PZH also suppressed tumor growth by inducing G2/M cell cycle arrest, which presented a different action mechanism with Sorafenib. Furthermore, Western blot analysis indicated that the level of p-CCNB1 was upregulated by PZH. Interestingly, some previous studies found that the upregulation of CCNB1 could promote the cell cycle to go forward, but there were other articles showed the result that upregulation of it would induce arrest of cell cycle especially arrest in G2/M [30].

Sorafenib is one of the anticancer drugs which was widely utilized in the clinical treatment of HCC. The major target of it is the serine-threonine kinase Raf-1, vascular endothelial growth factor receptor, and platelet-derived growth factor receptor [31]. In addition, previous studies and our results revealed that insulin and CDKs were the targets of Sorafenib that inhibited tumor growth [32–35]. In this study, we also found that both PZH and Sorafenib regulated the p53 and FAK signaling pathways to inhibit tumor growth. Although Sorafenib seems to be effective in prolonging median survival time in HCC patients, the response rate of it is actually low and may cause resistance in some patients [36]. Furthermore, the PI3K/Akt and PTEN/AKT pathways play roles in producing resistance to Sorafenib [37, 38]. Sorafenib slows cell cycle progression, prevents irradiated cells from reaching and accumulating at G2/M, and causes a reversible G1 delay [39]. Interestingly, our results indicated that PZH more forcefully impacted the PI3K and PTEN signaling pathways and induced G2/M arrest in the cell cycle. Furthermore, PZH had advantages in anti-inflammation and might make up for the deficiency of Sorafenib. PZH's effect suggests that its combination with Sorafenib might not only enhance the efficacy but also reduce the drug resistance to Sorafenib.

The limitations of our study were the small sample size of the proteomics experiment and the single animal model. In further studies, we will try to collect samples of patients from the clinic and increase the sample size for the microarray test to verify the mechanism of the antitumor effect of PZH more clearly.

In summary, our study demonstrated the underlying antitumor mechanisms of PZH in regulating the IL-6, TNFR1, and TNFR2 pathways and the G2/M DNA damage checkpoint regulation pathway to suppress inflammation and induce G2/M arrest in HCC. These findings provide a potential molecular basis for PZH to act as a therapeutic drug or as a supplement to chemotherapy drugs for HCC in the future.

## Data Availability

The data used to support the findings of this study are available from the corresponding author upon request.

## Supplementary Materials

Supplementary Materials The supplementary material are the processed data of Phospho Explorer antibody microarray (PEX100).
