# Supplementary material for: Deciphering Antitumor Mechanism of Pien Tze Huang in Mice of Hepatocellular Carcinoma Based on Proteomics
Source: J Immunol Res. 2020 Dec 3;2020:4876251. doi: 10.1155/2020/4876251 (PMC7728492; doi:10.1155/2020/4876251)
Supplement: Supplementary Materials — The supplementary material are the processed data of Phospho Explorer antibody microarray (PEX100). [file 4876251.f1.docx]

**Supplementary Table 1. The standard detection value of Model group**

| Name |  | FI_i_ | FI_j_ | X | Y | Z | I_i_ | I_j_ | Average | SD | CV |
| --- | --- | --- | --- | --- | --- | --- | --- | --- | --- | --- | --- |
| Positive Marker |  | 6704 | 6406 | 110 | 99.5 | 104.75 | 6384.04 | 6744.01 | 6564.02 | 254.54 | 0.04 |
| Positive Marker |  | 7319 | 6714 | 110 | 99.5 | 104.75 | 6969.68 | 7068.26 | 7018.97 | 69.70 | 0.01 |
| Negative control |  | 160 | 143 | 110 | 99.5 | 104.75 | 152.36 | 150.55 | 151.45 | 1.29 | 0.01 |
| Negative control |  | 193 | 164 | 110 | 99.5 | 104.75 | 183.79 | 172.65 | 178.22 | 7.87 | 0.04 |
| Negative control |  | 193 | 172 | 110 | 99.5 | 104.75 | 183.79 | 181.08 | 182.43 | 1.92 | 0.01 |
| Negative control |  | 196 | 194 | 110 | 99.5 | 104.75 | 186.65 | 204.24 | 195.44 | 12.44 | 0.06 |
| Empty |  | 48 | 54 | 110 | 99.5 | 104.75 | 45.71 | 56.85 | 51.28 | 7.88 | 0.15 |
| Empty |  | 49 | 55 | 110 | 99.5 | 104.75 | 46.66 | 57.90 | 52.28 | 7.95 | 0.15 |
| Empty |  | 57 | 70 | 110 | 99.5 | 104.75 | 54.28 | 73.69 | 63.99 | 13.73 | 0.21 |
| Empty |  | 58 | 68 | 110 | 99.5 | 104.75 | 55.23 | 71.59 | 63.41 | 11.57 | 0.18 |
| 14-3-3 beta/zeta (Ab-184/186) |  | 98 | 82 | 110 | 99.5 | 104.75 | 93.32 | 86.33 | 89.82 | 4.95 | 0.06 |
| 14-3-3 beta/zeta (Phospho-Ser184/186) |  | 90 | 68 | 110 | 99.5 | 104.75 | 85.70 | 71.59 | 78.65 | 9.98 | 0.13 |
| 14-3-3 theta/tau (Ab-232) |  | 80 | 80 | 110 | 99.5 | 104.75 | 76.18 | 84.22 | 80.20 | 5.68 | 0.07 |
| 14-3-3 theta/tau (Phospho-Ser232) |  | 153 | 113 | 110 | 99.5 | 104.75 | 145.70 | 118.96 | 132.33 | 18.90 | 0.14 |
| 14-3-3 zeta (Ab-58) |  | 94 | 81 | 110 | 99.5 | 104.75 | 89.51 | 85.27 | 87.39 | 3.00 | 0.03 |
| 14-3-3 zeta (Phospho-Ser58) |  | 82 | 198 | 110 | 99.5 | 104.75 | 78.09 | 208.45 | 143.27 | 92.18 | 0.64 |
| 14-3-3 zeta/delta (Ab-232) |  | 980 | 1586 | 110 | 99.5 | 104.75 | 933.23 | 1669.68 | 1301.46 | 520.75 | 0.40 |
| 14-3-3 zeta/delta (Phospho-Thr232) |  | 189 | 187 | 110 | 99.5 | 104.75 | 179.98 | 196.87 | 188.42 | 11.94 | 0.06 |
| 4E-BP1 (Ab-36) |  | 77 | 74 | 110 | 99.5 | 104.75 | 73.33 | 77.90 | 75.61 | 3.24 | 0.04 |
| 4E-BP1 (Ab-45) |  | 97 | 93 | 110 | 99.5 | 104.75 | 92.37 | 97.91 | 95.14 | 3.91 | 0.04 |
| 4E-BP1 (Ab-65) |  | 3347 | 3421 | 110 | 99.5 | 104.75 | 3187.26 | 3601.51 | 3394.38 | 292.92 | 0.09 |
| 4E-BP1 (Ab-70) |  | 165 | 153 | 110 | 99.5 | 104.75 | 157.13 | 161.07 | 159.10 | 2.79 | 0.02 |
| 4E-BP1 (Phospho-Ser65) |  | 67 | 70 | 110 | 99.5 | 104.75 | 63.80 | 73.69 | 68.75 | 6.99 | 0.10 |
| 4E-BP1 (Phospho-Thr36) |  | 110 | 222 | 110 | 99.5 | 104.75 | 104.75 | 233.71 | 169.23 | 91.19 | 0.54 |
| 4E-BP1 (Phospho-Thr45) |  | 89 | 76 | 110 | 99.5 | 104.75 | 84.75 | 80.01 | 82.38 | 3.35 | 0.04 |
| 4E-BP1 (Phospho-Thr70) |  | 62 | 66 | 110 | 99.5 | 104.75 | 59.04 | 69.48 | 64.26 | 7.38 | 0.11 |
| 6-phosphofructo-2-kinase/fructose-2,6-biphosphatase 2 (PFKFB2) (Ab-483) |  | 103 | 96 | 110 | 99.5 | 104.75 | 98.08 | 101.07 | 99.57 | 2.11 | 0.02 |
| 6-phosphofructo-2-kinase/fructose-2,6-biphosphatase 2 (PFKFB2) (Phospho-Ser483) |  | 72 | 73 | 110 | 99.5 | 104.75 | 68.56 | 76.85 | 72.71 | 5.86 | 0.08 |
| Abl1 (Ab-204) |  | 899 | 3862 | 110 | 99.5 | 104.75 | 856.09 | 4065.77 | 2460.93 | 2269.59 | 0.92 |
| Abl1 (Ab-754/735) |  | 69 | 68 | 110 | 99.5 | 104.75 | 65.71 | 71.59 | 68.65 | 4.16 | 0.06 |
| Abl1 (Phospho-Thr754/735) |  | 157 | 77 | 110 | 99.5 | 104.75 | 149.51 | 81.06 | 115.28 | 48.40 | 0.42 |
| Abl1 (Phospho-Tyr204) |  | 63 | 66 | 110 | 99.5 | 104.75 | 59.99 | 69.48 | 64.74 | 6.71 | 0.10 |
| Abl1 (Phospho-Tyr412) |  | 70 | 74 | 110 | 99.5 | 104.75 | 66.66 | 77.90 | 72.28 | 7.95 | 0.11 |
| ACC1 (Ab-79) |  | 93 | 102 | 110 | 99.5 | 104.75 | 88.56 | 107.38 | 97.97 | 13.31 | 0.14 |
| ACC1 (Ab-80) |  | 320 | 923 | 110 | 99.5 | 104.75 | 304.73 | 971.70 | 638.21 | 471.62 | 0.74 |
| ACC1 (Phospho-Ser79) |  | 67 | 237 | 110 | 99.5 | 104.75 | 63.80 | 249.51 | 156.65 | 131.31 | 0.84 |
| ACC1 (Phospho-Ser80) |  | 60 | 133 | 110 | 99.5 | 104.75 | 57.14 | 140.02 | 98.58 | 58.61 | 0.59 |
| ACK1 (Phospho-Tyr284) |  | 217 | 240 | 110 | 99.5 | 104.75 | 206.64 | 252.66 | 229.65 | 32.54 | 0.14 |
| Actin Pan (a/b/g) (Ab-55/53) |  | 183 | 312 | 110 | 99.5 | 104.75 | 174.27 | 328.46 | 251.36 | 109.03 | 0.43 |
| Actin Pan (a/b/g) (Phospho-Tyr55/53) |  | 145 | 68 | 110 | 99.5 | 104.75 | 138.08 | 71.59 | 104.83 | 47.02 | 0.45 |
| ADD1 (Ab-726) |  | 108 | 101 | 110 | 99.5 | 104.75 | 102.85 | 106.33 | 104.59 | 2.46 | 0.02 |
| ADD1 (Phospho-Ser726) |  | 158 | 84 | 110 | 99.5 | 104.75 | 150.46 | 88.43 | 119.45 | 43.86 | 0.37 |
| AFX/FOXO4 (Ab-197) |  | 127 | 109 | 110 | 99.5 | 104.75 | 120.94 | 114.75 | 117.84 | 4.38 | 0.04 |
| AFX/FOXO4 (Phospho-Ser197) |  | 85 | 80 | 110 | 99.5 | 104.75 | 80.94 | 84.22 | 82.58 | 2.32 | 0.03 |
| AKT1 (Ab-124) |  | 240 | 238 | 110 | 99.5 | 104.75 | 228.55 | 250.56 | 239.55 | 15.57 | 0.06 |
| AKT1 (Ab-246) |  | 78 | 78 | 110 | 99.5 | 104.75 | 74.28 | 82.12 | 78.20 | 5.54 | 0.07 |
| AKT1 (Ab-308) |  | 258 | 237 | 110 | 99.5 | 104.75 | 245.69 | 249.51 | 247.60 | 2.70 | 0.01 |
| AKT1 (Ab-326) |  | 152 | 163 | 110 | 99.5 | 104.75 | 144.75 | 171.60 | 158.17 | 18.99 | 0.12 |
| AKT1 (Ab-450) |  | 72 | 266 | 110 | 99.5 | 104.75 | 68.56 | 280.04 | 174.30 | 149.53 | 0.86 |
| AKT1 (Ab-473) |  | 88 | 70 | 110 | 99.5 | 104.75 | 83.80 | 73.69 | 78.75 | 7.15 | 0.09 |
| AKT1 (Ab-474) |  | 92 | 93 | 110 | 99.5 | 104.75 | 87.61 | 97.91 | 92.76 | 7.28 | 0.08 |
| AKT1 (Ab-72) |  | 93 | 97 | 110 | 99.5 | 104.75 | 88.56 | 102.12 | 95.34 | 9.59 | 0.10 |
| AKT1 (Phospho-Ser124) |  | 80 | 88 | 110 | 99.5 | 104.75 | 76.18 | 92.64 | 84.41 | 11.64 | 0.14 |
| AKT1 (Phospho-Ser246) |  | 77 | 70 | 110 | 99.5 | 104.75 | 73.33 | 73.69 | 73.51 | 0.26 | 0.00 |
| AKT1 (Phospho-Ser473) |  | 224 | 74 | 110 | 99.5 | 104.75 | 213.31 | 77.90 | 145.61 | 95.75 | 0.66 |
| AKT1 (Phospho-Thr308) |  | 223 | 174 | 110 | 99.5 | 104.75 | 212.36 | 183.18 | 197.77 | 20.63 | 0.10 |
| AKT1 (Phospho-Thr450) |  | 57 | 180 | 110 | 99.5 | 104.75 | 54.28 | 189.50 | 121.89 | 95.61 | 0.78 |
| AKT1 (Phospho-Thr72) |  | 68 | 65 | 110 | 99.5 | 104.75 | 64.75 | 68.43 | 66.59 | 2.60 | 0.04 |
| AKT1 (Phospho-Tyr326) |  | 276 | 81 | 110 | 99.5 | 104.75 | 262.83 | 85.27 | 174.05 | 125.55 | 0.72 |
| AKT1 (Phospho-Tyr474) |  | 69 | 69 | 110 | 99.5 | 104.75 | 65.71 | 72.64 | 69.17 | 4.90 | 0.07 |
| AKT1/2/3 (Ab-315) |  | 156 | 67 | 110 | 99.5 | 104.75 | 148.55 | 70.54 | 109.54 | 55.17 | 0.50 |
| AKT1S1 (Ab-246) |  | 1201 | 1300 | 110 | 99.5 | 104.75 | 1143.68 | 1368.59 | 1256.14 | 159.04 | 0.13 |
| AKT1S1 (Phospho-Thr246) |  | 226 | 242 | 110 | 99.5 | 104.75 | 215.21 | 254.77 | 234.99 | 27.97 | 0.12 |
| AKT2 (Ab-474) |  | 110 | 102 | 110 | 99.5 | 104.75 | 104.75 | 107.38 | 106.07 | 1.86 | 0.02 |
| AKT2 (Phospho-Ser474) |  | 172 | 140 | 110 | 99.5 | 104.75 | 163.79 | 147.39 | 155.59 | 11.60 | 0.07 |
| ALK (Ab-1507) |  | 78 | 71 | 110 | 99.5 | 104.75 | 74.28 | 74.75 | 74.51 | 0.33 | 0.00 |
| ALK (Ab-1604) |  | 130 | 75 | 110 | 99.5 | 104.75 | 123.80 | 78.96 | 101.38 | 31.71 | 0.31 |
| ALK (Phospho-Tyr1507) |  | 146 | 137 | 110 | 99.5 | 104.75 | 139.03 | 144.23 | 141.63 | 3.67 | 0.03 |
| ALK (Phospho-Tyr1604) |  | 129 | 117 | 110 | 99.5 | 104.75 | 122.84 | 123.17 | 123.01 | 0.23 | 0.00 |
| AMPK beta1 (Ab-182) |  | 306 | 354 | 110 | 99.5 | 104.75 | 291.40 | 372.68 | 332.04 | 57.48 | 0.17 |
| AMPK beta1 (Phospho-Ser182) |  | 67 | 67 | 110 | 99.5 | 104.75 | 63.80 | 70.54 | 67.17 | 4.76 | 0.07 |
| AMPK1 (Ab-172) |  | 86 | 67 | 110 | 99.5 | 104.75 | 81.90 | 70.54 | 76.22 | 8.03 | 0.11 |
| AMPK1 (Phospho-Thr172) |  | 92 | 102 | 110 | 99.5 | 104.75 | 87.61 | 107.38 | 97.50 | 13.98 | 0.14 |
| AMPK1/AMPK2 (Ab-485/491) |  | 99 | 95 | 110 | 99.5 | 104.75 | 94.28 | 100.01 | 97.14 | 4.06 | 0.04 |
| AMPK1/AMPK2 (Phospho-Ser485/491) |  | 99 | 101 | 110 | 99.5 | 104.75 | 94.28 | 106.33 | 100.30 | 8.52 | 0.08 |
| Amyloid beta A4 (Ab-743/668) |  | 766 | 336 | 110 | 99.5 | 104.75 | 729.44 | 353.73 | 541.58 | 265.67 | 0.49 |
| Amyloid beta A4 (Phospho-Thr743/668) |  | 165 | 195 | 110 | 99.5 | 104.75 | 157.13 | 205.29 | 181.21 | 34.06 | 0.19 |
| Androgen Receptor (Ab-213) |  | 129 | 107 | 110 | 99.5 | 104.75 | 122.84 | 112.65 | 117.74 | 7.21 | 0.06 |
| Androgen Receptor (Ab-650) |  | 163 | 162 | 110 | 99.5 | 104.75 | 155.22 | 170.55 | 162.88 | 10.84 | 0.07 |
| Androgen Receptor (Phospho-Ser213) |  | 97 | 89 | 110 | 99.5 | 104.75 | 92.37 | 93.70 | 93.03 | 0.94 | 0.01 |
| Androgen Receptor (Phospho-Ser650) |  | 110 | 86 | 110 | 99.5 | 104.75 | 104.75 | 90.54 | 97.64 | 10.05 | 0.10 |
| A-RAF (Ab-301/302) |  | 107 | 111 | 110 | 99.5 | 104.75 | 101.89 | 116.86 | 109.37 | 10.58 | 0.10 |
| A-RAF (Phospho-Tyr301/302) |  | 157 | 70 | 110 | 99.5 | 104.75 | 149.51 | 73.69 | 111.60 | 53.61 | 0.48 |
| Arrestin-1 (Ab-412) |  | 164 | 149 | 110 | 99.5 | 104.75 | 156.17 | 156.86 | 156.52 | 0.49 | 0.00 |
| Arrestin-1 (Phospho-Ser412) |  | 114 | 76 | 110 | 99.5 | 104.75 | 108.56 | 80.01 | 94.28 | 20.19 | 0.21 |
| ASK1 (Ab-83) |  | 95 | 87 | 110 | 99.5 | 104.75 | 90.47 | 91.59 | 91.03 | 0.80 | 0.01 |
| ASK1 (Ab-966) |  | 235 | 196 | 110 | 99.5 | 104.75 | 223.78 | 206.34 | 215.06 | 12.33 | 0.06 |
| ASK1 (Phospho-Ser83) |  | 118 | 64 | 110 | 99.5 | 104.75 | 112.37 | 67.38 | 89.87 | 31.81 | 0.35 |
| ASK1 (Phospho-Ser966) |  | 169 | 120 | 110 | 99.5 | 104.75 | 160.93 | 126.33 | 143.63 | 24.47 | 0.17 |
| ATF1 (Ab-63) |  | 192 | 194 | 110 | 99.5 | 104.75 | 182.84 | 204.24 | 193.54 | 15.13 | 0.08 |
| ATF1 (Phospho-Ser63) |  | 72 | 65 | 110 | 99.5 | 104.75 | 68.56 | 68.43 | 68.50 | 0.09 | 0.00 |
| ATF2 (Ab-112/94) |  | 101 | 73 | 110 | 99.5 | 104.75 | 96.18 | 76.85 | 86.52 | 13.67 | 0.16 |
| ATF2 (Ab-62/44) |  | 2026 | 1654 | 110 | 99.5 | 104.75 | 1929.30 | 1741.27 | 1835.29 | 132.96 | 0.07 |
| ATF2 (Ab-69/51) |  | 162 | 87 | 110 | 99.5 | 104.75 | 154.27 | 91.59 | 122.93 | 44.32 | 0.36 |
| ATF2 (Ab-71/53) |  | 85 | 67 | 110 | 99.5 | 104.75 | 80.94 | 70.54 | 75.74 | 7.36 | 0.10 |
| ATF2 (Ab-73/55) |  | 94 | 76 | 110 | 99.5 | 104.75 | 89.51 | 80.01 | 84.76 | 6.72 | 0.08 |
| ATF2 (Phospho-Ser112/94) |  | 235 | 74 | 110 | 99.5 | 104.75 | 223.78 | 77.90 | 150.84 | 103.15 | 0.68 |
| ATF2 (Phospho-Ser62/44) |  | 100 | 377 | 110 | 99.5 | 104.75 | 95.23 | 396.89 | 246.06 | 213.31 | 0.87 |
| ATF2 (Phospho-Thr69/51) |  | 91 | 81 | 110 | 99.5 | 104.75 | 86.66 | 85.27 | 85.97 | 0.98 | 0.01 |
| ATF2 (Phospho-Thr71/53) |  | 135 | 108 | 110 | 99.5 | 104.75 | 128.56 | 113.70 | 121.13 | 10.51 | 0.09 |
| ATF2 (Phospho-Thr73/55) |  | 138 | 121 | 110 | 99.5 | 104.75 | 131.41 | 127.38 | 129.40 | 2.85 | 0.02 |
| ATF4 (Ab-245) |  | 94 | 64 | 110 | 99.5 | 104.75 | 89.51 | 67.38 | 78.45 | 15.65 | 0.20 |
| ATF4 (Phospho-Ser245) |  | 79 | 246 | 110 | 99.5 | 104.75 | 75.23 | 258.98 | 167.10 | 129.93 | 0.78 |
| ATM (Ab-1981) |  | 83 | 85 | 110 | 99.5 | 104.75 | 79.04 | 89.48 | 84.26 | 7.39 | 0.09 |
| ATP1A1/Na+K+ ATPase1 (Ab-23) |  | 215 | 145 | 110 | 99.5 | 104.75 | 204.74 | 152.65 | 178.69 | 36.83 | 0.21 |
| ATP1A1/Na+K+ ATPase1 (Phospho-Ser23) |  | 164 | 155 | 110 | 99.5 | 104.75 | 156.17 | 163.18 | 159.68 | 4.95 | 0.03 |
| ATPase (Ab-16) |  | 69 | 71 | 110 | 99.5 | 104.75 | 65.71 | 74.75 | 70.23 | 6.39 | 0.09 |
| ATPase (Phospho-Ser16) |  | 70 | 73 | 110 | 99.5 | 104.75 | 66.66 | 76.85 | 71.76 | 7.21 | 0.10 |
| ATP-Citrate Lyase (Ab-454) |  | 104 | 95 | 110 | 99.5 | 104.75 | 99.04 | 100.01 | 99.52 | 0.69 | 0.01 |
| ATP-Citrate Lyase (Phospho-Ser454) |  | 74 | 119 | 110 | 99.5 | 104.75 | 70.47 | 125.28 | 97.87 | 38.76 | 0.40 |
| ATRIP (Ab-68/72) |  | 1659 | 1902 | 110 | 99.5 | 104.75 | 1579.82 | 2002.36 | 1791.09 | 298.78 | 0.17 |
| ATRIP (Phospho-Ser68/72) |  | 73 | 75 | 110 | 99.5 | 104.75 | 69.52 | 78.96 | 74.24 | 6.68 | 0.09 |
| AurA (Ab-288) |  | 123 | 85 | 110 | 99.5 | 104.75 | 117.13 | 89.48 | 103.31 | 19.55 | 0.19 |
| AurA (Ab-342) |  | 94 | 93 | 110 | 99.5 | 104.75 | 89.51 | 97.91 | 93.71 | 5.94 | 0.06 |
| AurA (Phospho-Ser342) |  | 85 | 151 | 110 | 99.5 | 104.75 | 80.94 | 158.97 | 119.96 | 55.17 | 0.46 |
| AurA (Phospho-Thr288) |  | 91 | 91 | 110 | 99.5 | 104.75 | 86.66 | 95.80 | 91.23 | 6.47 | 0.07 |
| AurB (Ab-12) |  | 70 | 66 | 110 | 99.5 | 104.75 | 66.66 | 69.48 | 68.07 | 2.00 | 0.03 |
| AurB (Ab-232) |  | 87 | 77 | 110 | 99.5 | 104.75 | 82.85 | 81.06 | 81.96 | 1.26 | 0.02 |
| AurB (Phospho-Thr232) |  | 75 | 71 | 110 | 99.5 | 104.75 | 71.42 | 74.75 | 73.08 | 2.35 | 0.03 |
| AurB (Phospho-Tyr12) |  | 78 | 74 | 110 | 99.5 | 104.75 | 74.28 | 77.90 | 76.09 | 2.56 | 0.03 |
| AurB/C (Ab-202/175) |  | 102 | 115 | 110 | 99.5 | 104.75 | 97.13 | 121.07 | 109.10 | 16.93 | 0.16 |
| AXL (Phospho-Tyr691) |  | 75 | 97 | 110 | 99.5 | 104.75 | 71.42 | 102.12 | 86.77 | 21.71 | 0.25 |
| BAD (Ab-112) |  | 159 | 153 | 110 | 99.5 | 104.75 | 151.41 | 161.07 | 156.24 | 6.83 | 0.04 |
| BAD (Ab-134) |  | 112 | 114 | 110 | 99.5 | 104.75 | 106.65 | 120.02 | 113.33 | 9.45 | 0.08 |
| BAD (Ab-136) |  | 118 | 112 | 110 | 99.5 | 104.75 | 112.37 | 117.91 | 115.14 | 3.92 | 0.03 |
| BAD (Ab-155) |  | 102 | 93 | 110 | 99.5 | 104.75 | 97.13 | 97.91 | 97.52 | 0.55 | 0.01 |
| BAD (Ab-91/128) |  | 176 | 69 | 110 | 99.5 | 104.75 | 167.60 | 72.64 | 120.12 | 67.15 | 0.56 |
| BAD (Phospho-Ser112) |  | 183 | 142 | 110 | 99.5 | 104.75 | 174.27 | 149.49 | 161.88 | 17.52 | 0.11 |
| BAD (Phospho-Ser134) |  | 76 | 74 | 110 | 99.5 | 104.75 | 72.37 | 77.90 | 75.14 | 3.91 | 0.05 |
| BAD (Phospho-Ser136) |  | 92 | 85 | 110 | 99.5 | 104.75 | 87.61 | 89.48 | 88.55 | 1.33 | 0.01 |
| BAD (Phospho-Ser155) |  | 92 | 85 | 110 | 99.5 | 104.75 | 87.61 | 89.48 | 88.55 | 1.33 | 0.01 |
| BAD (Phospho-Ser91/128) |  | 59 | 64 | 110 | 99.5 | 104.75 | 56.18 | 67.38 | 61.78 | 7.91 | 0.13 |
| BAX (Ab-167) |  | 120 | 124 | 110 | 99.5 | 104.75 | 114.27 | 130.54 | 122.41 | 11.50 | 0.09 |
| BAX (Phospho-Thr167) |  | 75 | 71 | 110 | 99.5 | 104.75 | 71.42 | 74.75 | 73.08 | 2.35 | 0.03 |
| BCL-2 (Ab-56) |  | 116 | 103 | 110 | 99.5 | 104.75 | 110.46 | 108.43 | 109.45 | 1.43 | 0.01 |
| BCL-2 (Ab-69) |  | 74 | 74 | 110 | 99.5 | 104.75 | 70.47 | 77.90 | 74.19 | 5.26 | 0.07 |
| BCL-2 (Ab-70) |  | 89 | 81 | 110 | 99.5 | 104.75 | 84.75 | 85.27 | 85.01 | 0.37 | 0.00 |
| BCL-2 (Phospho-Ser70) |  | 85 | 81 | 110 | 99.5 | 104.75 | 80.94 | 85.27 | 83.11 | 3.06 | 0.04 |
| BCL-2 (Phospho-Ser87) |  | 80 | 60 | 110 | 99.5 | 104.75 | 76.18 | 63.17 | 69.67 | 9.20 | 0.13 |
| BCL-2 (Phospho-Thr56) |  | 132 | 112 | 110 | 99.5 | 104.75 | 125.70 | 117.91 | 121.80 | 5.51 | 0.05 |
| BCL-2 (Phospho-Thr69) |  | 155 | 179 | 110 | 99.5 | 104.75 | 147.60 | 188.44 | 168.02 | 28.88 | 0.17 |
| BCL-6 (Ab-333) |  | 225 | 207 | 110 | 99.5 | 104.75 | 214.26 | 217.92 | 216.09 | 2.59 | 0.01 |
| BCL-XL (Ab-47) |  | 1116 | 1416 | 110 | 99.5 | 104.75 | 1062.74 | 1490.71 | 1276.72 | 302.63 | 0.24 |
| BCL-XL (Phospho-Ser62) |  | 339 | 338 | 110 | 99.5 | 104.75 | 322.82 | 355.83 | 339.33 | 23.34 | 0.07 |
| BCL-XL (Phospho-Thr47) |  | 74 | 78 | 110 | 99.5 | 104.75 | 70.47 | 82.12 | 76.29 | 8.24 | 0.11 |
| BCR (Ab-177) |  | 153 | 141 | 110 | 99.5 | 104.75 | 145.70 | 148.44 | 147.07 | 1.94 | 0.01 |
| BCR (Ab-360) |  | 84 | 75 | 110 | 99.5 | 104.75 | 79.99 | 78.96 | 79.47 | 0.73 | 0.01 |
| BCR (Phospho-Tyr177) |  | 107 | 103 | 110 | 99.5 | 104.75 | 101.89 | 108.43 | 105.16 | 4.63 | 0.04 |
| BCR (Phospho-Tyr360) |  | 145 | 150 | 110 | 99.5 | 104.75 | 138.08 | 157.91 | 148.00 | 14.03 | 0.09 |
| Beta actin |  | 3292 | 3371 | 110 | 99.5 | 104.75 | 3134.88 | 3548.87 | 3341.87 | 292.73 | 0.09 |
| BID (Ab-78) |  | 187 | 230 | 110 | 99.5 | 104.75 | 178.08 | 242.14 | 210.11 | 45.30 | 0.22 |
| BID (Phospho-Ser78) |  | 106 | 112 | 110 | 99.5 | 104.75 | 100.94 | 117.91 | 109.43 | 12.00 | 0.11 |
| BIM (Ab-69/65) |  | 90 | 92 | 110 | 99.5 | 104.75 | 85.70 | 96.85 | 91.28 | 7.88 | 0.09 |
| BIM (Phospho-Ser69/65) |  | 128 | 137 | 110 | 99.5 | 104.75 | 121.89 | 144.23 | 133.06 | 15.80 | 0.12 |
| BLNK (Ab-96) |  | 488 | 255 | 110 | 99.5 | 104.75 | 464.71 | 268.45 | 366.58 | 138.77 | 0.38 |
| BLNK (Phospho-Tyr84) |  | 91 | 77 | 110 | 99.5 | 104.75 | 86.66 | 81.06 | 83.86 | 3.96 | 0.05 |
| BLNK (Phospho-Tyr96) |  | 171 | 166 | 110 | 99.5 | 104.75 | 162.84 | 174.76 | 168.80 | 8.43 | 0.05 |
| B-RAF (Ab-446) |  | 80 | 81 | 110 | 99.5 | 104.75 | 76.18 | 85.27 | 80.73 | 6.43 | 0.08 |
| B-RAF (Ab-598) |  | 1073 | 2812 | 110 | 99.5 | 104.75 | 1021.79 | 2960.37 | 1991.08 | 1370.79 | 0.69 |
| B-RAF (Ab-601) |  | 79 | 72 | 110 | 99.5 | 104.75 | 75.23 | 75.80 | 75.51 | 0.40 | 0.01 |
| B-RAF (Phospho-Ser446) |  | 199 | 205 | 110 | 99.5 | 104.75 | 189.50 | 215.82 | 202.66 | 18.61 | 0.09 |
| B-RAF (Phospho-Ser601) |  | 87 | 72 | 110 | 99.5 | 104.75 | 82.85 | 75.80 | 79.32 | 4.98 | 0.06 |
| B-RAF (Phospho-Thr598) |  | 80 | 74 | 110 | 99.5 | 104.75 | 76.18 | 77.90 | 77.04 | 1.22 | 0.02 |
| BRCA1 (Ab-1457) |  | 101 | 267 | 110 | 99.5 | 104.75 | 96.18 | 281.09 | 188.63 | 130.75 | 0.69 |
| BRCA1 (Ab-1524) |  | 358 | 343 | 110 | 99.5 | 104.75 | 340.91 | 361.10 | 351.01 | 14.27 | 0.04 |
| BRCA1 (Phospho-Ser1423) |  | 1090 | 812 | 110 | 99.5 | 104.75 | 1037.98 | 854.84 | 946.41 | 129.49 | 0.14 |
| BRCA1 (Phospho-Ser1457) |  | 66 | 66 | 110 | 99.5 | 104.75 | 62.85 | 69.48 | 66.17 | 4.69 | 0.07 |
| BRCA1 (Phospho-Ser1524) |  | 108 | 95 | 110 | 99.5 | 104.75 | 102.85 | 100.01 | 101.43 | 2.00 | 0.02 |
| Breast tumor kinase (Phospho-Tyr447) |  | 92 | 89 | 110 | 99.5 | 104.75 | 87.61 | 93.70 | 90.65 | 4.30 | 0.05 |
| BTK (Ab-223) |  | 175 | 86 | 110 | 99.5 | 104.75 | 166.65 | 90.54 | 128.59 | 53.82 | 0.42 |
| BTK (Phospho-Tyr223) |  | 101 | 120 | 110 | 99.5 | 104.75 | 96.18 | 126.33 | 111.26 | 21.32 | 0.19 |
| BTK (Phospho-Tyr550) |  | 64 | 75 | 110 | 99.5 | 104.75 | 60.95 | 78.96 | 69.95 | 12.74 | 0.18 |
| c-Abl (Ab-412) |  | 280 | 132 | 110 | 99.5 | 104.75 | 266.64 | 138.96 | 202.80 | 90.28 | 0.45 |
| c-Abl (Phospho-Tyr245) |  | 177 | 70 | 110 | 99.5 | 104.75 | 168.55 | 73.69 | 121.12 | 67.08 | 0.55 |
| c-Abl (Phospho-Tyr412) |  | 246 | 245 | 110 | 99.5 | 104.75 | 234.26 | 257.93 | 246.09 | 16.74 | 0.07 |
| Calmodulin (Ab-79/81) |  | 139 | 1369 | 110 | 99.5 | 104.75 | 132.37 | 1441.23 | 786.80 | 925.51 | 1.18 |
| Calmodulin (Phospho-Thr79/Ser81) |  | 66 | 72 | 110 | 99.5 | 104.75 | 62.85 | 75.80 | 69.32 | 9.16 | 0.13 |
| Calsenilin/KCNIP3 (Ab-63) |  | 2043 | 2101 | 110 | 99.5 | 104.75 | 1945.49 | 2211.86 | 2078.67 | 188.35 | 0.09 |
| Calsenilin/KCNIP3 (Phospho-Ser63) |  | 810 | 549 | 110 | 99.5 | 104.75 | 771.34 | 577.97 | 674.65 | 136.74 | 0.20 |
| CaMK1-alpha (Ab-177) |  | 82 | 76 | 110 | 99.5 | 104.75 | 78.09 | 80.01 | 79.05 | 1.36 | 0.02 |
| CaMK1-alpha (Phospho-Thr177) |  | 82 | 68 | 110 | 99.5 | 104.75 | 78.09 | 71.59 | 74.84 | 4.60 | 0.06 |
| CaMK2 alpha/beta/delta (Phospho-Thr305) |  | 95 | 129 | 110 | 99.5 | 104.75 | 90.47 | 135.81 | 113.14 | 32.06 | 0.28 |
| CaMK2A (Ab-286) |  | 236 | 203 | 110 | 99.5 | 104.75 | 224.74 | 213.71 | 219.22 | 7.80 | 0.04 |
| CaMK2A (Phospho-Thr286) |  | 88 | 90 | 110 | 99.5 | 104.75 | 83.80 | 94.75 | 89.27 | 7.74 | 0.09 |
| CaMK2-beta/gamma/delta (Ab-287) |  | 614 | 636 | 110 | 99.5 | 104.75 | 584.70 | 669.56 | 627.13 | 60.01 | 0.10 |
| CaMK2-beta/gamma/delta (Phospho-Thr287) |  | 185 | 156 | 110 | 99.5 | 104.75 | 176.17 | 164.23 | 170.20 | 8.44 | 0.05 |
| CaMK4 (Ab-196/200) |  | 76 | 71 | 110 | 99.5 | 104.75 | 72.37 | 74.75 | 73.56 | 1.68 | 0.02 |
| CaMK4 (Phospho-Thr196/200) |  | 201 | 60 | 110 | 99.5 | 104.75 | 191.41 | 63.17 | 127.29 | 90.68 | 0.71 |
| Caspase 1 (Ab-376) |  | 183 | 179 | 110 | 99.5 | 104.75 | 174.27 | 188.44 | 181.36 | 10.03 | 0.06 |
| Caspase 1 (Phospho-Ser376) |  | 116 | 90 | 110 | 99.5 | 104.75 | 110.46 | 94.75 | 102.61 | 11.11 | 0.11 |
| Caspase 2 (Ab-157) |  | 86 | 74 | 110 | 99.5 | 104.75 | 81.90 | 77.90 | 79.90 | 2.82 | 0.04 |
| Caspase 2 (Phospho-Ser157) |  | 176 | 135 | 110 | 99.5 | 104.75 | 167.60 | 142.12 | 154.86 | 18.01 | 0.12 |
| Caspase 3 (Ab-150) |  | 71 | 64 | 110 | 99.5 | 104.75 | 67.61 | 67.38 | 67.49 | 0.17 | 0.00 |
| Caspase 3 (Phospho-Ser150) |  | 61 | 61 | 110 | 99.5 | 104.75 | 58.09 | 64.22 | 61.15 | 4.33 | 0.07 |
| Caspase 6 (Ab-257) |  | 102 | 109 | 110 | 99.5 | 104.75 | 97.13 | 114.75 | 105.94 | 12.46 | 0.12 |
| Caspase 6 (Phospho-Ser257) |  | 62 | 61 | 110 | 99.5 | 104.75 | 59.04 | 64.22 | 61.63 | 3.66 | 0.06 |
| Caspase 8 (Ab-347) |  | 124 | 144 | 110 | 99.5 | 104.75 | 118.08 | 151.60 | 134.84 | 23.70 | 0.18 |
| Caspase 8 (Phospho-Ser347) |  | 78 | 117 | 110 | 99.5 | 104.75 | 74.28 | 123.17 | 98.73 | 34.57 | 0.35 |
| Caspase 9 (Ab-125) |  | 80 | 78 | 110 | 99.5 | 104.75 | 76.18 | 82.12 | 79.15 | 4.20 | 0.05 |
| Caspase 9 (Ab-144) |  | 70 | 68 | 110 | 99.5 | 104.75 | 66.66 | 71.59 | 69.12 | 3.49 | 0.05 |
| Caspase 9 (Ab-153) |  | 279 | 1110 | 110 | 99.5 | 104.75 | 265.68 | 1168.57 | 717.13 | 638.44 | 0.89 |
| Caspase 9 (Ab-196) |  | 1514 | 2343 | 110 | 99.5 | 104.75 | 1441.74 | 2466.63 | 1954.18 | 724.70 | 0.37 |
| Caspase 9 (Phospho-Ser144) |  | 74 | 66 | 110 | 99.5 | 104.75 | 70.47 | 69.48 | 69.98 | 0.70 | 0.01 |
| Caspase 9 (Phospho-Ser196) |  | 586 | 845 | 110 | 99.5 | 104.75 | 558.03 | 889.59 | 723.81 | 234.44 | 0.32 |
| Caspase 9 (Phospho-Thr125) |  | 64 | 60 | 110 | 99.5 | 104.75 | 60.95 | 63.17 | 62.06 | 1.57 | 0.03 |
| Caspase 9 (Phospho-Tyr153) |  | 156 | 95 | 110 | 99.5 | 104.75 | 148.55 | 100.01 | 124.28 | 34.32 | 0.28 |
| Catalase (Ab-385) |  | 900 | 882 | 110 | 99.5 | 104.75 | 857.05 | 928.54 | 892.79 | 50.55 | 0.06 |
| Catalase (Phospho-Tyr385) |  | 146 | 105 | 110 | 99.5 | 104.75 | 139.03 | 110.54 | 124.79 | 20.15 | 0.16 |
| Catenin beta (Ab-33) |  | 77 | 77 | 110 | 99.5 | 104.75 | 73.33 | 81.06 | 77.19 | 5.47 | 0.07 |
| Catenin beta (Ab-37) |  | 266 | 339 | 110 | 99.5 | 104.75 | 253.30 | 356.89 | 305.10 | 73.24 | 0.24 |
| Catenin beta (Ab-41/45) |  | 118 | 93 | 110 | 99.5 | 104.75 | 112.37 | 97.91 | 105.14 | 10.23 | 0.10 |
| Catenin beta (Ab-489) |  | 69 | 70 | 110 | 99.5 | 104.75 | 65.71 | 73.69 | 69.70 | 5.65 | 0.08 |
| Catenin beta (Ab-654) |  | 133 | 137 | 110 | 99.5 | 104.75 | 126.65 | 144.23 | 135.44 | 12.43 | 0.09 |
| Catenin beta (Phospho-Ser33) |  | 172 | 206 | 110 | 99.5 | 104.75 | 163.79 | 216.87 | 190.33 | 37.53 | 0.20 |
| Catenin beta (Phospho-Ser37) |  | 422 | 64 | 110 | 99.5 | 104.75 | 401.86 | 67.38 | 234.62 | 236.51 | 1.01 |
| Catenin beta (Phospho-Thr41/Ser45) |  | 106 | 103 | 110 | 99.5 | 104.75 | 100.94 | 108.43 | 104.69 | 5.30 | 0.05 |
| Catenin beta (Phospho-Tyr489) |  | 70 | 67 | 110 | 99.5 | 104.75 | 66.66 | 70.54 | 68.60 | 2.74 | 0.04 |
| Catenin beta (Phospho-Tyr654) |  | 69 | 63 | 110 | 99.5 | 104.75 | 65.71 | 66.32 | 66.02 | 0.44 | 0.01 |
| Catenin delta-1 (Ab-228) |  | 145 | 164 | 110 | 99.5 | 104.75 | 138.08 | 172.65 | 155.37 | 24.45 | 0.16 |
| Catenin delta-1 (Phospho-Tyr228) |  | 286 | 285 | 110 | 99.5 | 104.75 | 272.35 | 300.04 | 286.19 | 19.58 | 0.07 |
| Caveolin-1 (Ab-14) |  | 90 | 86 | 110 | 99.5 | 104.75 | 85.70 | 90.54 | 88.12 | 3.42 | 0.04 |
| Caveolin-1 (Phospho-Tyr14) |  | 92 | 91 | 110 | 99.5 | 104.75 | 87.61 | 95.80 | 91.71 | 5.79 | 0.06 |
| CBL (Phospho-Tyr700) |  | 359 | 108 | 110 | 99.5 | 104.75 | 341.87 | 113.70 | 227.78 | 161.34 | 0.71 |
| CBL (Phospho-Tyr774) |  | 66 | 66 | 110 | 99.5 | 104.75 | 62.85 | 69.48 | 66.17 | 4.69 | 0.07 |
| CD19 (Ab-531) |  | 85 | 63 | 110 | 99.5 | 104.75 | 80.94 | 66.32 | 73.63 | 10.34 | 0.14 |
| CD19 (Phospho-Tyr531) |  | 103 | 103 | 110 | 99.5 | 104.75 | 98.08 | 108.43 | 103.26 | 7.32 | 0.07 |
| CD22/BL-CAM (Phospho-Tyr807) |  | 184 | 166 | 110 | 99.5 | 104.75 | 175.22 | 174.76 | 174.99 | 0.32 | 0.00 |
| CD227/mucin 1 (Ab-1243) |  | 94 | 82 | 110 | 99.5 | 104.75 | 89.51 | 86.33 | 87.92 | 2.25 | 0.03 |
| CD227/mucin 1 (Phospho-Tyr1243) |  | 147 | 168 | 110 | 99.5 | 104.75 | 139.98 | 176.86 | 158.42 | 26.08 | 0.16 |
| CD28 (Phospho-Tyr218) |  | 86 | 74 | 110 | 99.5 | 104.75 | 81.90 | 77.90 | 79.90 | 2.82 | 0.04 |
| CD32 (FcgammaRIIb) (Ab-292) |  | 1064 | 878 | 110 | 99.5 | 104.75 | 1013.22 | 924.33 | 968.77 | 62.86 | 0.06 |
| CD3Z (Ab-142) |  | 109 | 133 | 110 | 99.5 | 104.75 | 103.80 | 140.02 | 121.91 | 25.61 | 0.21 |
| CD3Z (Phospho-Tyr142) |  | 106 | 73 | 110 | 99.5 | 104.75 | 100.94 | 76.85 | 88.90 | 17.03 | 0.19 |
| CD4 (Ab-433) |  | 922 | 1090 | 110 | 99.5 | 104.75 | 878.00 | 1147.51 | 1012.75 | 190.58 | 0.19 |
| CD4 (Phospho-Ser433) |  | 125 | 255 | 110 | 99.5 | 104.75 | 119.03 | 268.45 | 193.74 | 105.66 | 0.55 |
| CD45 (Phospho-Ser1007) |  | 118 | 89 | 110 | 99.5 | 104.75 | 112.37 | 93.70 | 103.03 | 13.20 | 0.13 |
| CD5 (Ab-453) |  | 619 | 700 | 110 | 99.5 | 104.75 | 589.46 | 736.93 | 663.20 | 104.28 | 0.16 |
| CD5 (Phospho-Tyr453) |  | 103 | 70 | 110 | 99.5 | 104.75 | 98.08 | 73.69 | 85.89 | 17.25 | 0.20 |
| CDC25A (Ab-124) |  | 61 | 59 | 110 | 99.5 | 104.75 | 58.09 | 62.11 | 60.10 | 2.85 | 0.05 |
| CDC25A (Ab-178) |  | 83 | 73 | 110 | 99.5 | 104.75 | 79.04 | 76.85 | 77.95 | 1.55 | 0.02 |
| CDC25A (Ab-75) |  | 68 | 63 | 110 | 99.5 | 104.75 | 64.75 | 66.32 | 65.54 | 1.11 | 0.02 |
| CDC25A (Phospho-Ser124) |  | 97 | 89 | 110 | 99.5 | 104.75 | 92.37 | 93.70 | 93.03 | 0.94 | 0.01 |
| CDC25A (Phospho-Ser75) |  | 162 | 134 | 110 | 99.5 | 104.75 | 154.27 | 141.07 | 147.67 | 9.33 | 0.06 |
| CDC25B (Ab-323) |  | 78 | 76 | 110 | 99.5 | 104.75 | 74.28 | 80.01 | 77.14 | 4.05 | 0.05 |
| CDC25B (Ab-353) |  | 62 | 58 | 110 | 99.5 | 104.75 | 59.04 | 61.06 | 60.05 | 1.43 | 0.02 |
| CDC25B (Phospho-Ser323) |  | 75 | 83 | 110 | 99.5 | 104.75 | 71.42 | 87.38 | 79.40 | 11.28 | 0.14 |
| CDC25B (Phospho-Ser353) |  | 248 | 264 | 110 | 99.5 | 104.75 | 236.16 | 277.93 | 257.05 | 29.53 | 0.11 |
| CDC25C (Ab-216) |  | 1873 | 1870 | 110 | 99.5 | 104.75 | 1783.61 | 1968.67 | 1876.14 | 130.86 | 0.07 |
| CDC25C (Phospho-Ser216) |  | 125 | 105 | 110 | 99.5 | 104.75 | 119.03 | 110.54 | 114.79 | 6.01 | 0.05 |
| CDC25C (Phospho-Thr48) |  | 87 | 120 | 110 | 99.5 | 104.75 | 82.85 | 126.33 | 104.59 | 30.75 | 0.29 |
| CDK1/CDC2 (Ab-14) |  | 71 | 70 | 110 | 99.5 | 104.75 | 67.61 | 73.69 | 70.65 | 4.30 | 0.06 |
| CDK1/CDC2 (Ab-15) |  | 83 | 77 | 110 | 99.5 | 104.75 | 79.04 | 81.06 | 80.05 | 1.43 | 0.02 |
| CDK1/CDC2 (Phospho-Thr14) |  | 106 | 72 | 110 | 99.5 | 104.75 | 100.94 | 75.80 | 88.37 | 17.78 | 0.20 |
| CDK1/CDC2 (Phospho-Tyr15) |  | 143 | 130 | 110 | 99.5 | 104.75 | 136.18 | 136.86 | 136.52 | 0.48 | 0.00 |
| CDK2 (Ab-160) |  | 83 | 81 | 110 | 99.5 | 104.75 | 79.04 | 85.27 | 82.16 | 4.41 | 0.05 |
| CDK2 (Phospho-Thr160) |  | 412 | 83 | 110 | 99.5 | 104.75 | 392.34 | 87.38 | 239.86 | 215.64 | 0.90 |
| CDK5 (Ab-15) |  | 1264 | 1455 | 110 | 99.5 | 104.75 | 1203.67 | 1531.77 | 1367.72 | 232.00 | 0.17 |
| CDK5 (Phospho-Tyr15) |  | 247 | 197 | 110 | 99.5 | 104.75 | 235.21 | 207.39 | 221.30 | 19.67 | 0.09 |
| CDK7 (Ab-170) |  | 74 | 75 | 110 | 99.5 | 104.75 | 70.47 | 78.96 | 74.71 | 6.00 | 0.08 |
| CDK7 (Phospho-Thr170) |  | 76 | 78 | 110 | 99.5 | 104.75 | 72.37 | 82.12 | 77.24 | 6.89 | 0.09 |
| Chk1 (Ab-280) |  | 80 | 66 | 110 | 99.5 | 104.75 | 76.18 | 69.48 | 72.83 | 4.74 | 0.07 |
| Chk1 (Ab-286) |  | 554 | 757 | 110 | 99.5 | 104.75 | 527.56 | 796.94 | 662.25 | 190.48 | 0.29 |
| Chk1 (Ab-317) |  | 296 | 376 | 110 | 99.5 | 104.75 | 281.87 | 395.84 | 338.86 | 80.59 | 0.24 |
| Chk1 (Ab-345) |  | 114 | 107 | 110 | 99.5 | 104.75 | 108.56 | 112.65 | 110.60 | 2.89 | 0.03 |
| Chk1 (Phospho-Ser280) |  | 241 | 93 | 110 | 99.5 | 104.75 | 229.50 | 97.91 | 163.70 | 93.05 | 0.57 |
| Chk1 (Phospho-Ser286) |  | 80 | 79 | 110 | 99.5 | 104.75 | 76.18 | 83.17 | 79.68 | 4.94 | 0.06 |
| Chk1 (Phospho-Ser296) |  | 163 | 114 | 110 | 99.5 | 104.75 | 155.22 | 120.02 | 137.62 | 24.89 | 0.18 |
| Chk1 (Phospho-Ser301) |  | 58 | 59 | 110 | 99.5 | 104.75 | 55.23 | 62.11 | 58.67 | 4.87 | 0.08 |
| Chk1 (Phospho-Ser317) |  | 83 | 72 | 110 | 99.5 | 104.75 | 79.04 | 75.80 | 77.42 | 2.29 | 0.03 |
| Chk1 (Phospho-Ser345) |  | 89 | 99 | 110 | 99.5 | 104.75 | 84.75 | 104.22 | 94.49 | 13.77 | 0.15 |
| Chk2 (Ab-383) |  | 197 | 227 | 110 | 99.5 | 104.75 | 187.60 | 238.98 | 213.29 | 36.33 | 0.17 |
| Chk2 (Ab-387) |  | 260 | 273 | 110 | 99.5 | 104.75 | 247.59 | 287.40 | 267.50 | 28.15 | 0.11 |
| Chk2 (Ab-516) |  | 205 | 179 | 110 | 99.5 | 104.75 | 195.22 | 188.44 | 191.83 | 4.79 | 0.02 |
| Chk2 (Ab-68) |  | 1858 | 1929 | 110 | 99.5 | 104.75 | 1769.32 | 2030.78 | 1900.05 | 184.88 | 0.10 |
| Chk2 (Phospho-Ser516) |  | 120 | 107 | 110 | 99.5 | 104.75 | 114.27 | 112.65 | 113.46 | 1.15 | 0.01 |
| Chk2 (Phospho-Thr383) |  | 73 | 190 | 110 | 99.5 | 104.75 | 69.52 | 200.03 | 134.77 | 92.28 | 0.68 |
| Chk2 (Phospho-Thr387) |  | 209 | 193 | 110 | 99.5 | 104.75 | 199.03 | 203.18 | 201.10 | 2.94 | 0.01 |
| Chk2 (Phospho-Thr68) |  | 474 | 145 | 110 | 99.5 | 104.75 | 451.38 | 152.65 | 302.01 | 211.23 | 0.70 |
| c-Jun (Ab-170) |  | 251 | 97 | 110 | 99.5 | 104.75 | 239.02 | 102.12 | 170.57 | 96.80 | 0.57 |
| c-Jun (Ab-239) |  | 188 | 179 | 110 | 99.5 | 104.75 | 179.03 | 188.44 | 183.74 | 6.66 | 0.04 |
| c-Jun (Ab-243) |  | 122 | 133 | 110 | 99.5 | 104.75 | 116.18 | 140.02 | 128.10 | 16.86 | 0.13 |
| c-Jun (Ab-63) |  | 128 | 128 | 110 | 99.5 | 104.75 | 121.89 | 134.75 | 128.32 | 9.10 | 0.07 |
| c-Jun (Ab-91) |  | 124 | 109 | 110 | 99.5 | 104.75 | 118.08 | 114.75 | 116.42 | 2.36 | 0.02 |
| c-Jun (Ab-93) |  | 85 | 76 | 110 | 99.5 | 104.75 | 80.94 | 80.01 | 80.48 | 0.66 | 0.01 |
| c-Jun (Phospho-Ser243) |  | 106 | 138 | 110 | 99.5 | 104.75 | 100.94 | 145.28 | 123.11 | 31.35 | 0.25 |
| c-Jun (Phospho-Ser63) |  | 122 | 107 | 110 | 99.5 | 104.75 | 116.18 | 112.65 | 114.41 | 2.50 | 0.02 |
| c-Jun (Phospho-Ser73) |  | 308 | 197 | 110 | 99.5 | 104.75 | 293.30 | 207.39 | 250.35 | 60.74 | 0.24 |
| c-Jun (Phospho-Thr239) |  | 124 | 119 | 110 | 99.5 | 104.75 | 118.08 | 125.28 | 121.68 | 5.09 | 0.04 |
| c-Jun (Phospho-Thr91) |  | 138 | 119 | 110 | 99.5 | 104.75 | 131.41 | 125.28 | 128.35 | 4.34 | 0.03 |
| c-Jun (Phospho-Thr93) |  | 97 | 92 | 110 | 99.5 | 104.75 | 92.37 | 96.85 | 94.61 | 3.17 | 0.03 |
| c-Jun (Phospho-Tyr170) |  | 79 | 76 | 110 | 99.5 | 104.75 | 75.23 | 80.01 | 77.62 | 3.38 | 0.04 |
| CK1-A (Ab-321) |  | 77 | 77 | 110 | 99.5 | 104.75 | 73.33 | 81.06 | 77.19 | 5.47 | 0.07 |
| CK1-A (Phospho-Thr321) |  | 239 | 228 | 110 | 99.5 | 104.75 | 227.59 | 240.03 | 233.81 | 8.79 | 0.04 |
| CK1-A/A2 (Phospho-Tyr294) |  | 138 | 150 | 110 | 99.5 | 104.75 | 131.41 | 157.91 | 144.66 | 18.74 | 0.13 |
| CK2-b (Ab-209) |  | 100 | 119 | 110 | 99.5 | 104.75 | 95.23 | 125.28 | 110.25 | 21.25 | 0.19 |
| CK2-b (Phospho-Ser209) |  | 85 | 99 | 110 | 99.5 | 104.75 | 80.94 | 104.22 | 92.58 | 16.46 | 0.18 |
| Claudin 3 (Ab-219) |  | 65 | 166 | 110 | 99.5 | 104.75 | 61.90 | 174.76 | 118.33 | 79.80 | 0.67 |
| Claudin 3 (Phospho-Tyr219) |  | 281 | 241 | 110 | 99.5 | 104.75 | 267.59 | 253.72 | 260.65 | 9.81 | 0.04 |
| Claudin 6 (Phospho-Tyr219) |  | 122 | 111 | 110 | 99.5 | 104.75 | 116.18 | 116.86 | 116.52 | 0.48 | 0.00 |
| Claudin 7 (Ab-210) |  | 85 | 89 | 110 | 99.5 | 104.75 | 80.94 | 93.70 | 87.32 | 9.02 | 0.10 |
| Claudin 7 (Phospho-Tyr210) |  | 205 | 181 | 110 | 99.5 | 104.75 | 195.22 | 190.55 | 192.88 | 3.30 | 0.02 |
| Coagulation Factor III (Phospho-Ser290) |  | 83 | 77 | 110 | 99.5 | 104.75 | 79.04 | 81.06 | 80.05 | 1.43 | 0.02 |
| Cofilin (Ab-3) |  | 122 | 101 | 110 | 99.5 | 104.75 | 116.18 | 106.33 | 111.25 | 6.96 | 0.06 |
| Cofilin (Phospho-Ser3) |  | 158 | 135 | 110 | 99.5 | 104.75 | 150.46 | 142.12 | 146.29 | 5.89 | 0.04 |
| Connexin 43 (Ab-367) |  | 246 | 245 | 110 | 99.5 | 104.75 | 234.26 | 257.93 | 246.09 | 16.74 | 0.07 |
| Connexin 43 (Phospho-Ser367) |  | 139 | 347 | 110 | 99.5 | 104.75 | 132.37 | 365.31 | 248.84 | 164.72 | 0.66 |
| Cortactin (Ab-421) |  | 93 | 85 | 110 | 99.5 | 104.75 | 88.56 | 89.48 | 89.02 | 0.65 | 0.01 |
| Cortactin (Ab-466) |  | 144 | 144 | 110 | 99.5 | 104.75 | 137.13 | 151.60 | 144.36 | 10.23 | 0.07 |
| Cortactin (Phospho-Tyr421) |  | 121 | 101 | 110 | 99.5 | 104.75 | 115.23 | 106.33 | 110.78 | 6.29 | 0.06 |
| Cortactin (Phospho-Tyr466) |  | 94 | 84 | 110 | 99.5 | 104.75 | 89.51 | 88.43 | 88.97 | 0.76 | 0.01 |
| CPI17 alpha (Ab-38) |  | 117 | 96 | 110 | 99.5 | 104.75 | 111.42 | 101.07 | 106.24 | 7.32 | 0.07 |
| CPI17 alpha (Phospho-Thr38) |  | 90 | 88 | 110 | 99.5 | 104.75 | 85.70 | 92.64 | 89.17 | 4.91 | 0.06 |
| c-PLA2 (Ab-505) |  | 79 | 74 | 110 | 99.5 | 104.75 | 75.23 | 77.90 | 76.57 | 1.89 | 0.02 |
| c-PLA2 (Phospho-Ser505) |  | 77 | 69 | 110 | 99.5 | 104.75 | 73.33 | 72.64 | 72.98 | 0.48 | 0.01 |
| CREB (Ab-100) |  | 75 | 72 | 110 | 99.5 | 104.75 | 71.42 | 75.80 | 73.61 | 3.10 | 0.04 |
| CREB (Ab-121) |  | 99 | 89 | 110 | 99.5 | 104.75 | 94.28 | 93.70 | 93.99 | 0.41 | 0.00 |
| CREB (Ab-129) |  | 74 | 74 | 110 | 99.5 | 104.75 | 70.47 | 77.90 | 74.19 | 5.26 | 0.07 |
| CREB (Ab-133) |  | 83 | 82 | 110 | 99.5 | 104.75 | 79.04 | 86.33 | 82.68 | 5.15 | 0.06 |
| CREB (Ab-142) |  | 91 | 96 | 110 | 99.5 | 104.75 | 86.66 | 101.07 | 93.86 | 10.19 | 0.11 |
| CREB (Phospho-Ser121) |  | 126 | 74 | 110 | 99.5 | 104.75 | 119.99 | 77.90 | 98.95 | 29.76 | 0.30 |
| CREB (Phospho-Ser129) |  | 76 | 74 | 110 | 99.5 | 104.75 | 72.37 | 77.90 | 75.14 | 3.91 | 0.05 |
| CREB (Phospho-Ser133) |  | 94 | 90 | 110 | 99.5 | 104.75 | 89.51 | 94.75 | 92.13 | 3.70 | 0.04 |
| CREB (Phospho-Ser142) |  | 77 | 75 | 110 | 99.5 | 104.75 | 73.33 | 78.96 | 76.14 | 3.98 | 0.05 |
| CREB (Phospho-Thr100) |  | 173 | 137 | 110 | 99.5 | 104.75 | 164.74 | 144.23 | 154.49 | 14.51 | 0.09 |
| CrkII (Ab-221) |  | 453 | 424 | 110 | 99.5 | 104.75 | 431.38 | 446.37 | 438.88 | 10.60 | 0.02 |
| CrkII (Phospho-Tyr221) |  | 299 | 96 | 110 | 99.5 | 104.75 | 284.73 | 101.07 | 192.90 | 129.87 | 0.67 |
| CrkL (Phospho-Tyr207) |  | 79 | 77 | 110 | 99.5 | 104.75 | 75.23 | 81.06 | 78.15 | 4.12 | 0.05 |
| CXCR4 (Phospho-Ser339) |  | 58 | 60 | 110 | 99.5 | 104.75 | 55.23 | 63.17 | 59.20 | 5.61 | 0.09 |
| Cyclin B1 (Ab-126) |  | 130 | 532 | 110 | 99.5 | 104.75 | 123.80 | 560.07 | 341.93 | 308.49 | 0.90 |
| Cyclin B1 (Ab-147) |  | 72 | 65 | 110 | 99.5 | 104.75 | 68.56 | 68.43 | 68.50 | 0.09 | 0.00 |
| Cyclin B1 (Phospho-Ser126) |  | 92 | 74 | 110 | 99.5 | 104.75 | 87.61 | 77.90 | 82.76 | 6.86 | 0.08 |
| Cyclin B1 (Phospho-Ser147) |  | 84 | 227 | 110 | 99.5 | 104.75 | 79.99 | 238.98 | 159.48 | 112.42 | 0.70 |
| Cyclin C (Phospho-Ser275) |  | 89 | 87 | 110 | 99.5 | 104.75 | 84.75 | 91.59 | 88.17 | 4.84 | 0.05 |
| Cyclin D1 (Ab-286) |  | 1048 | 1068 | 110 | 99.5 | 104.75 | 997.98 | 1124.35 | 1061.17 | 89.36 | 0.08 |
| Cyclin D1 (Ab-90) |  | 118 | 148 | 110 | 99.5 | 104.75 | 112.37 | 155.81 | 134.09 | 30.72 | 0.23 |
| Cyclin D1 (Phospho-Thr286) |  | 396 | 531 | 110 | 99.5 | 104.75 | 377.10 | 559.02 | 468.06 | 128.64 | 0.27 |
| Cyclin D2 (Ab-280) |  | 77 | 72 | 110 | 99.5 | 104.75 | 73.33 | 75.80 | 74.56 | 1.75 | 0.02 |
| Cyclin D3 (Ab-283) |  | 179 | 208 | 110 | 99.5 | 104.75 | 170.46 | 218.97 | 194.72 | 34.31 | 0.18 |
| Cyclin D3 (Phospho-Thr283) |  | 65 | 65 | 110 | 99.5 | 104.75 | 61.90 | 68.43 | 65.16 | 4.62 | 0.07 |
| Cyclin E1 (Ab-395) |  | 126 | 126 | 110 | 99.5 | 104.75 | 119.99 | 132.65 | 126.32 | 8.95 | 0.07 |
| Cyclin E1 (Ab-77) |  | 1852 | 1924 | 110 | 99.5 | 104.75 | 1763.61 | 2025.52 | 1894.56 | 185.20 | 0.10 |
| Cyclin E1 (Phospho-Thr395) |  | 124 | 110 | 110 | 99.5 | 104.75 | 118.08 | 115.80 | 116.94 | 1.61 | 0.01 |
| Cyclin E1 (Phospho-Thr77) |  | 385 | 195 | 110 | 99.5 | 104.75 | 366.63 | 205.29 | 285.96 | 114.08 | 0.40 |
| Cyclin E2 (Ab-392) |  | 123 | 133 | 110 | 99.5 | 104.75 | 117.13 | 140.02 | 128.57 | 16.18 | 0.13 |
| Cyclin E2 (Phospho-Thr392) |  | 91 | 67 | 110 | 99.5 | 104.75 | 86.66 | 70.54 | 78.60 | 11.40 | 0.15 |
| DAB1 (Ab-220) |  | 150 | 130 | 110 | 99.5 | 104.75 | 142.84 | 136.86 | 139.85 | 4.23 | 0.03 |
| DAB1 (Ab-232) |  | 78 | 67 | 110 | 99.5 | 104.75 | 74.28 | 70.54 | 72.41 | 2.65 | 0.04 |
| DAB1 (Phospho-Tyr220) |  | 131 | 113 | 110 | 99.5 | 104.75 | 124.75 | 118.96 | 121.86 | 4.09 | 0.03 |
| DAB1 (Phospho-Tyr232) |  | 63 | 64 | 110 | 99.5 | 104.75 | 59.99 | 67.38 | 63.69 | 5.22 | 0.08 |
| DAPP1 (Ab-139) |  | 94 | 85 | 110 | 99.5 | 104.75 | 89.51 | 89.48 | 89.50 | 0.02 | 0.00 |
| DAPP1 (Phospho-Tyr139) |  | 71 | 62 | 110 | 99.5 | 104.75 | 67.61 | 65.27 | 66.44 | 1.65 | 0.02 |
| DARPP-32 (Ab-34) |  | 250 | 146 | 110 | 99.5 | 104.75 | 238.07 | 153.70 | 195.89 | 59.65 | 0.30 |
| DARPP-32 (Ab-75) |  | 174 | 76 | 110 | 99.5 | 104.75 | 165.70 | 80.01 | 122.85 | 60.59 | 0.49 |
| DARPP-32 (Phospho-Thr34) |  | 143 | 122 | 110 | 99.5 | 104.75 | 136.18 | 128.44 | 132.31 | 5.47 | 0.04 |
| DARPP-32 (Phospho-Thr75) |  | 146 | 175 | 110 | 99.5 | 104.75 | 139.03 | 184.23 | 161.63 | 31.96 | 0.20 |
| DAXX (Phospho-Ser668) |  | 161 | 145 | 110 | 99.5 | 104.75 | 153.32 | 152.65 | 152.98 | 0.47 | 0.00 |
| DDX5/DEAD-box protein 5 (Ab-593) |  | 93 | 92 | 110 | 99.5 | 104.75 | 88.56 | 96.85 | 92.71 | 5.86 | 0.06 |
| DDX5/DEAD-box protein 5 (Phospho-Tyr593) |  | 125 | 89 | 110 | 99.5 | 104.75 | 119.03 | 93.70 | 106.37 | 17.92 | 0.17 |
| DNA-PK (Ab-2056) |  | 64 | 62 | 110 | 99.5 | 104.75 | 60.95 | 65.27 | 63.11 | 3.06 | 0.05 |
| DNA-PK (Ab-2638) |  | 164 | 202 | 110 | 99.5 | 104.75 | 156.17 | 212.66 | 184.42 | 39.94 | 0.22 |
| DNA-PK (Ab-2647) |  | 67 | 70 | 110 | 99.5 | 104.75 | 63.80 | 73.69 | 68.75 | 6.99 | 0.10 |
| DNA-PK (Phospho-Thr2638) |  | 73 | 71 | 110 | 99.5 | 104.75 | 69.52 | 74.75 | 72.13 | 3.70 | 0.05 |
| DNA-PK (Phospho-Thr2647) |  | 70 | 66 | 110 | 99.5 | 104.75 | 66.66 | 69.48 | 68.07 | 2.00 | 0.03 |
| Dok-1 (Ab-362) |  | 113 | 111 | 110 | 99.5 | 104.75 | 107.61 | 116.86 | 112.23 | 6.54 | 0.06 |
| Dok-1 (Ab-398) |  | 237 | 145 | 110 | 99.5 | 104.75 | 225.69 | 152.65 | 189.17 | 51.65 | 0.27 |
| Dok-1 (Phospho-Tyr362) |  | 86 | 82 | 110 | 99.5 | 104.75 | 81.90 | 86.33 | 84.11 | 3.13 | 0.04 |
| Dok-1 (Phospho-Tyr398) |  | 69 | 75 | 110 | 99.5 | 104.75 | 65.71 | 78.96 | 72.33 | 9.37 | 0.13 |
| Dok-2 (Ab-299) |  | 135 | 110 | 110 | 99.5 | 104.75 | 128.56 | 115.80 | 122.18 | 9.02 | 0.07 |
| Dok-2 (Phospho-Tyr299) |  | 119 | 253 | 110 | 99.5 | 104.75 | 113.32 | 266.35 | 189.83 | 108.21 | 0.57 |
| DYN1 (Ab-774) |  | 2552 | 2988 | 110 | 99.5 | 104.75 | 2430.20 | 3145.66 | 2787.93 | 505.91 | 0.18 |
| DYN1 (Phospho-Ser774) |  | 453 | 170 | 110 | 99.5 | 104.75 | 431.38 | 178.97 | 305.17 | 178.48 | 0.58 |
| E2F1 (Ab-433) |  | 447 | 891 | 110 | 99.5 | 104.75 | 425.67 | 938.01 | 681.84 | 362.28 | 0.53 |
| E2F1 (Phospho-Thr433) |  | 105 | 76 | 110 | 99.5 | 104.75 | 99.99 | 80.01 | 90.00 | 14.13 | 0.16 |
| EEF2 (Ab-56) |  | 387 | 361 | 110 | 99.5 | 104.75 | 368.53 | 380.05 | 374.29 | 8.14 | 0.02 |
| EEF2 (Phospho-Thr56) |  | 159 | 119 | 110 | 99.5 | 104.75 | 151.41 | 125.28 | 138.35 | 18.48 | 0.13 |
| eEF2K (Ab-366) |  | 301 | 337 | 110 | 99.5 | 104.75 | 286.63 | 354.78 | 320.71 | 48.19 | 0.15 |
| eEF2K (Phospho-Ser366) |  | 229 | 183 | 110 | 99.5 | 104.75 | 218.07 | 192.66 | 205.36 | 17.97 | 0.09 |
| EGFR (Ab-1016) |  | 141 | 156 | 110 | 99.5 | 104.75 | 134.27 | 164.23 | 149.25 | 21.19 | 0.14 |
| EGFR (Ab-1069) |  | 61 | 60 | 110 | 99.5 | 104.75 | 58.09 | 63.17 | 60.63 | 3.59 | 0.06 |
| EGFR (Ab-1070) |  | 1095 | 1328 | 110 | 99.5 | 104.75 | 1042.74 | 1398.07 | 1220.40 | 251.26 | 0.21 |
| EGFR (Ab-1092) |  | 265 | 228 | 110 | 99.5 | 104.75 | 252.35 | 240.03 | 246.19 | 8.71 | 0.04 |
| EGFR (Ab-1110) |  | 160 | 164 | 110 | 99.5 | 104.75 | 152.36 | 172.65 | 162.51 | 14.35 | 0.09 |
| EGFR (Ab-1172) |  | 924 | 816 | 110 | 99.5 | 104.75 | 879.90 | 859.06 | 869.48 | 14.74 | 0.02 |
| EGFR (Ab-1197) |  | 112 | 95 | 110 | 99.5 | 104.75 | 106.65 | 100.01 | 103.33 | 4.70 | 0.05 |
| EGFR (Ab-678) |  | 80 | 68 | 110 | 99.5 | 104.75 | 76.18 | 71.59 | 73.88 | 3.25 | 0.04 |
| EGFR (Ab-693) |  | 596 | 279 | 110 | 99.5 | 104.75 | 567.55 | 293.72 | 430.64 | 193.63 | 0.45 |
| EGFR (Ab-869) |  | 1494 | 1614 | 110 | 99.5 | 104.75 | 1422.70 | 1699.16 | 1560.93 | 195.49 | 0.13 |
| EGFR (Ab-998) |  | 77 | 73 | 110 | 99.5 | 104.75 | 73.33 | 76.85 | 75.09 | 2.49 | 0.03 |
| EGFR (Phospho-Ser1070) |  | 81 | 82 | 110 | 99.5 | 104.75 | 77.13 | 86.33 | 81.73 | 6.50 | 0.08 |
| EGFR (Phospho-Thr678) |  | 150 | 179 | 110 | 99.5 | 104.75 | 142.84 | 188.44 | 165.64 | 32.25 | 0.19 |
| EGFR (Phospho-Thr693) |  | 77 | 64 | 110 | 99.5 | 104.75 | 73.33 | 67.38 | 70.35 | 4.21 | 0.06 |
| EGFR (Phospho-Tyr1016) |  | 100 | 96 | 110 | 99.5 | 104.75 | 95.23 | 101.07 | 98.15 | 4.13 | 0.04 |
| EGFR (Phospho-Tyr1069) |  | 95 | 126 | 110 | 99.5 | 104.75 | 90.47 | 132.65 | 111.56 | 29.83 | 0.27 |
| EGFR (Phospho-Tyr1092) |  | 93 | 86 | 110 | 99.5 | 104.75 | 88.56 | 90.54 | 89.55 | 1.40 | 0.02 |
| EGFR (Phospho-Tyr1110) |  | 291 | 245 | 110 | 99.5 | 104.75 | 277.11 | 257.93 | 267.52 | 13.57 | 0.05 |
| EGFR (Phospho-Tyr1172) |  | 76 | 75 | 110 | 99.5 | 104.75 | 72.37 | 78.96 | 75.67 | 4.66 | 0.06 |
| EGFR (Phospho-Tyr1197) |  | 317 | 96 | 110 | 99.5 | 104.75 | 301.87 | 101.07 | 201.47 | 141.99 | 0.70 |
| EGFR (Phospho-Tyr869) |  | 143 | 115 | 110 | 99.5 | 104.75 | 136.18 | 121.07 | 128.62 | 10.68 | 0.08 |
| eIF2A (Ab-51) |  | 204 | 197 | 110 | 99.5 | 104.75 | 194.26 | 207.39 | 200.83 | 9.28 | 0.05 |
| eIF2A (Phospho-Ser51) |  | 140 | 129 | 110 | 99.5 | 104.75 | 133.32 | 135.81 | 134.56 | 1.76 | 0.01 |
| eIF4B (Phospho-Ser422) |  | 68 | 68 | 110 | 99.5 | 104.75 | 64.75 | 71.59 | 68.17 | 4.83 | 0.07 |
| eIF4E (Ab-209) |  | 103 | 78 | 110 | 99.5 | 104.75 | 98.08 | 82.12 | 90.10 | 11.29 | 0.13 |
| eIF4E (Phospho-Ser209) |  | 196 | 87 | 110 | 99.5 | 104.75 | 186.65 | 91.59 | 139.12 | 67.21 | 0.48 |
| eIF4G (Ab-1108) |  | 343 | 448 | 110 | 99.5 | 104.75 | 326.63 | 471.64 | 399.13 | 102.54 | 0.26 |
| eIF4G (Phospho-Ser1108) |  | 95 | 82 | 110 | 99.5 | 104.75 | 90.47 | 86.33 | 88.40 | 2.93 | 0.03 |
| Elk1 (Ab-383) |  | 110 | 101 | 110 | 99.5 | 104.75 | 104.75 | 106.33 | 105.54 | 1.12 | 0.01 |
| Elk1 (Ab-389) |  | 271 | 174 | 110 | 99.5 | 104.75 | 258.07 | 183.18 | 220.62 | 52.95 | 0.24 |
| Elk1 (Ab-417) |  | 130 | 108 | 110 | 99.5 | 104.75 | 123.80 | 113.70 | 118.75 | 7.14 | 0.06 |
| Elk1 (Phospho-Ser383) |  | 711 | 628 | 110 | 99.5 | 104.75 | 677.07 | 661.14 | 669.10 | 11.26 | 0.02 |
| Elk1 (Phospho-Ser389) |  | 75 | 70 | 110 | 99.5 | 104.75 | 71.42 | 73.69 | 72.56 | 1.61 | 0.02 |
| Elk1 (Phospho-Thr417) |  | 715 | 100 | 110 | 99.5 | 104.75 | 680.88 | 105.28 | 393.08 | 407.01 | 1.04 |
| eNOS (Ab-1177) |  | 235 | 108 | 110 | 99.5 | 104.75 | 223.78 | 113.70 | 168.74 | 77.84 | 0.46 |
| eNOS (Ab-1179) |  | 130 | 112 | 110 | 99.5 | 104.75 | 123.80 | 117.91 | 120.85 | 4.16 | 0.03 |
| eNOS (Ab-495) |  | 80 | 73 | 110 | 99.5 | 104.75 | 76.18 | 76.85 | 76.52 | 0.47 | 0.01 |
| eNOS (Ab-615) |  | 97 | 106 | 110 | 99.5 | 104.75 | 92.37 | 111.59 | 101.98 | 13.59 | 0.13 |
| eNOS (Phospho-Ser1177) |  | 109 | 102 | 110 | 99.5 | 104.75 | 103.80 | 107.38 | 105.59 | 2.53 | 0.02 |
| eNOS (Phospho-Ser615) |  | 140 | 152 | 110 | 99.5 | 104.75 | 133.32 | 160.02 | 146.67 | 18.88 | 0.13 |
| eNOS (Phospho-Thr495) |  | 171 | 109 | 110 | 99.5 | 104.75 | 162.84 | 114.75 | 138.79 | 34.00 | 0.24 |
| EPB41 (Ab-418/660) |  | 170 | 164 | 110 | 99.5 | 104.75 | 161.89 | 172.65 | 167.27 | 7.61 | 0.05 |
| EPB41 (Phospho-Tyr418/660) |  | 94 | 114 | 110 | 99.5 | 104.75 | 89.51 | 120.02 | 104.76 | 21.57 | 0.21 |
| EPHA2/3/4 (Ab-588/596) |  | 118 | 113 | 110 | 99.5 | 104.75 | 112.37 | 118.96 | 115.67 | 4.66 | 0.04 |
| EPHA2/3/4 (Phospho-Tyr588/596) |  | 86 | 72 | 110 | 99.5 | 104.75 | 81.90 | 75.80 | 78.85 | 4.31 | 0.05 |
| EPHB1/2 (Ab-594/604) |  | 75 | 68 | 110 | 99.5 | 104.75 | 71.42 | 71.59 | 71.50 | 0.12 | 0.00 |
| EPHB1/2 (Phospho-Tyr594/604) |  | 81 | 74 | 110 | 99.5 | 104.75 | 77.13 | 77.90 | 77.52 | 0.54 | 0.01 |
| Ephrin B1 (Ab-317) |  | 75 | 76 | 110 | 99.5 | 104.75 | 71.42 | 80.01 | 75.72 | 6.07 | 0.08 |
| Ephrin B1 (Phospho-Tyr317) |  | 83 | 76 | 110 | 99.5 | 104.75 | 79.04 | 80.01 | 79.52 | 0.69 | 0.01 |
| Ephrin B1/B2/B3 (Phospho-Tyr324) |  | 86 | 78 | 110 | 99.5 | 104.75 | 81.90 | 82.12 | 82.01 | 0.16 | 0.00 |
| Ephrin B2 (Ab-330) |  | 289 | 407 | 110 | 99.5 | 104.75 | 275.21 | 428.47 | 351.84 | 108.38 | 0.31 |
| Ephrin B2 (Phospho-Tyr330) |  | 96 | 72 | 110 | 99.5 | 104.75 | 91.42 | 75.80 | 83.61 | 11.04 | 0.13 |
| Epo-R (Ab-368) |  | 108 | 95 | 110 | 99.5 | 104.75 | 102.85 | 100.01 | 101.43 | 2.00 | 0.02 |
| Epo-R (Phospho-Tyr368) |  | 85 | 81 | 110 | 99.5 | 104.75 | 80.94 | 85.27 | 83.11 | 3.06 | 0.04 |
| ERK3 (Ab-189) |  | 64 | 63 | 110 | 99.5 | 104.75 | 60.95 | 66.32 | 63.63 | 3.80 | 0.06 |
| ERK3 (Phospho-Ser189) |  | 64 | 65 | 110 | 99.5 | 104.75 | 60.95 | 68.43 | 64.69 | 5.29 | 0.08 |
| ERK8 (Phospho-Thr175/Tyr177) |  | 235 | 71 | 110 | 99.5 | 104.75 | 223.78 | 74.75 | 149.27 | 105.39 | 0.71 |
| Estrogen Receptor-alpha (Ab-104) |  | 489 | 208 | 110 | 99.5 | 104.75 | 465.66 | 218.97 | 342.32 | 174.43 | 0.51 |
| Estrogen Receptor-alpha (Ab-106) |  | 2433 | 2320 | 110 | 99.5 | 104.75 | 2316.88 | 2442.41 | 2379.65 | 88.76 | 0.04 |
| Estrogen Receptor-alpha (Ab-118) |  | 98 | 82 | 110 | 99.5 | 104.75 | 93.32 | 86.33 | 89.82 | 4.95 | 0.06 |
| Estrogen Receptor-alpha (Ab-167) |  | 130 | 119 | 110 | 99.5 | 104.75 | 123.80 | 125.28 | 124.54 | 1.05 | 0.01 |
| Estrogen Receptor-alpha (Phospho-Ser104) |  | 111 | 99 | 110 | 99.5 | 104.75 | 105.70 | 104.22 | 104.96 | 1.05 | 0.01 |
| Estrogen Receptor-alpha (Phospho-Ser106) |  | 122 | 115 | 110 | 99.5 | 104.75 | 116.18 | 121.07 | 118.62 | 3.46 | 0.03 |
| Estrogen Receptor-alpha (Phospho-Ser118) |  | 294 | 112 | 110 | 99.5 | 104.75 | 279.97 | 117.91 | 198.94 | 114.59 | 0.58 |
| Estrogen Receptor-alpha (Phospho-Ser167) |  | 1012 | 162 | 110 | 99.5 | 104.75 | 963.70 | 170.55 | 567.12 | 560.84 | 0.99 |
| ETK (Ab-40) |  | 108 | 124 | 110 | 99.5 | 104.75 | 102.85 | 130.54 | 116.69 | 19.58 | 0.17 |
| ETK (Ab-566) |  | 119 | 144 | 110 | 99.5 | 104.75 | 113.32 | 151.60 | 132.46 | 27.07 | 0.20 |
| ETK (Phospho-Tyr40) |  | 89 | 68 | 110 | 99.5 | 104.75 | 84.75 | 71.59 | 78.17 | 9.31 | 0.12 |
| Ezrin (Ab-353) |  | 149 | 118 | 110 | 99.5 | 104.75 | 141.89 | 124.23 | 133.06 | 12.49 | 0.09 |
| Ezrin (Ab-478) |  | 68 | 69 | 110 | 99.5 | 104.75 | 64.75 | 72.64 | 68.70 | 5.58 | 0.08 |
| Ezrin (Ab-566) |  | 666 | 674 | 110 | 99.5 | 104.75 | 634.21 | 709.56 | 671.89 | 53.28 | 0.08 |
| Ezrin (Phospho-Thr566) |  | 104 | 84 | 110 | 99.5 | 104.75 | 99.04 | 88.43 | 93.73 | 7.50 | 0.08 |
| Ezrin (Phospho-Tyr353) |  | 237 | 394 | 110 | 99.5 | 104.75 | 225.69 | 414.79 | 320.24 | 133.71 | 0.42 |
| Ezrin (Phospho-Tyr478) |  | 68 | 66 | 110 | 99.5 | 104.75 | 64.75 | 69.48 | 67.12 | 3.34 | 0.05 |
| FADD (Phospho-Ser194) |  | 645 | 734 | 110 | 99.5 | 104.75 | 614.22 | 772.73 | 693.47 | 112.09 | 0.16 |
| FAK (Ab-397) |  | 374 | 249 | 110 | 99.5 | 104.75 | 356.15 | 262.14 | 309.14 | 66.48 | 0.22 |
| FAK (Ab-407) |  | 74 | 72 | 110 | 99.5 | 104.75 | 70.47 | 75.80 | 73.13 | 3.77 | 0.05 |
| FAK (Ab-576) |  | 79 | 79 | 110 | 99.5 | 104.75 | 75.23 | 83.17 | 79.20 | 5.61 | 0.07 |
| FAK (Ab-861) |  | 100 | 88 | 110 | 99.5 | 104.75 | 95.23 | 92.64 | 93.94 | 1.83 | 0.02 |
| FAK (Ab-910) |  | 1042 | 2605 | 110 | 99.5 | 104.75 | 992.27 | 2742.45 | 1867.36 | 1237.57 | 0.66 |
| FAK (Ab-925) |  | 107 | 106 | 110 | 99.5 | 104.75 | 101.89 | 111.59 | 106.74 | 6.86 | 0.06 |
| FAK (Phospho-Ser910) |  | 315 | 204 | 110 | 99.5 | 104.75 | 299.97 | 214.76 | 257.36 | 60.25 | 0.23 |
| FAK (Phospho-Tyr397) |  | 64 | 63 | 110 | 99.5 | 104.75 | 60.95 | 66.32 | 63.63 | 3.80 | 0.06 |
| FAK (Phospho-Tyr407) |  | 227 | 61 | 110 | 99.5 | 104.75 | 216.17 | 64.22 | 140.19 | 107.44 | 0.77 |
| FAK (Phospho-Tyr576) |  | 150 | 88 | 110 | 99.5 | 104.75 | 142.84 | 92.64 | 117.74 | 35.50 | 0.30 |
| FAK (Phospho-Tyr861) |  | 89 | 104 | 110 | 99.5 | 104.75 | 84.75 | 109.49 | 97.12 | 17.49 | 0.18 |
| FAK (Phospho-Tyr925) |  | 97 | 89 | 110 | 99.5 | 104.75 | 92.37 | 93.70 | 93.03 | 0.94 | 0.01 |
| FAS (Ab-291) |  | 191 | 139 | 110 | 99.5 | 104.75 | 181.88 | 146.33 | 164.11 | 25.14 | 0.15 |
| FAS (Phospho-Tyr291) |  | 122 | 63 | 110 | 99.5 | 104.75 | 116.18 | 66.32 | 91.25 | 35.25 | 0.39 |
| FER (Ab-402) |  | 134 | 176 | 110 | 99.5 | 104.75 | 127.60 | 185.29 | 156.45 | 40.79 | 0.26 |
| FER (Phospho-Tyr402) |  | 632 | 726 | 110 | 99.5 | 104.75 | 601.84 | 764.31 | 683.07 | 114.88 | 0.17 |
| FGFR1 (Ab-154) |  | 93 | 91 | 110 | 99.5 | 104.75 | 88.56 | 95.80 | 92.18 | 5.12 | 0.06 |
| FGFR1 (Ab-766) |  | 219 | 168 | 110 | 99.5 | 104.75 | 208.55 | 176.86 | 192.71 | 22.40 | 0.12 |
| FGFR1 (Phospho-Tyr154) |  | 364 | 313 | 110 | 99.5 | 104.75 | 346.63 | 329.52 | 338.07 | 12.10 | 0.04 |
| FGFR1 (Phospho-Tyr654) |  | 471 | 629 | 110 | 99.5 | 104.75 | 448.52 | 662.19 | 555.35 | 151.09 | 0.27 |
| FGFR1 (Phospho-Tyr766) |  | 73 | 83 | 110 | 99.5 | 104.75 | 69.52 | 87.38 | 78.45 | 12.63 | 0.16 |
| Filamin A (Ab-2152) |  | 195 | 191 | 110 | 99.5 | 104.75 | 185.69 | 201.08 | 193.39 | 10.88 | 0.06 |
| Filamin A (Phospho-Ser2152) |  | 100 | 92 | 110 | 99.5 | 104.75 | 95.23 | 96.85 | 96.04 | 1.15 | 0.01 |
| FKHR (Ab-256) |  | 102 | 92 | 110 | 99.5 | 104.75 | 97.13 | 96.85 | 96.99 | 0.20 | 0.00 |
| FKHR (Ab-319) |  | 96 | 85 | 110 | 99.5 | 104.75 | 91.42 | 89.48 | 90.45 | 1.37 | 0.02 |
| FKHR (Phospho-Ser256) |  | 94 | 88 | 110 | 99.5 | 104.75 | 89.51 | 92.64 | 91.08 | 2.21 | 0.02 |
| FKHR (Phospho-Ser319) |  | 90 | 86 | 110 | 99.5 | 104.75 | 85.70 | 90.54 | 88.12 | 3.42 | 0.04 |
| FKHR/FOXO1A (Ab-329) |  | 73 | 72 | 110 | 99.5 | 104.75 | 69.52 | 75.80 | 72.66 | 4.44 | 0.06 |
| FKHR/FOXO1A (Phospho-Ser329) |  | 59 | 59 | 110 | 99.5 | 104.75 | 56.18 | 62.11 | 59.15 | 4.19 | 0.07 |
| FKHRL1/FOXO3A (Ab-253) |  | 289 | 318 | 110 | 99.5 | 104.75 | 275.21 | 334.78 | 304.99 | 42.12 | 0.14 |
| FKHRL1/FOXO3A (Phospho-Ser253) |  | 78 | 77 | 110 | 99.5 | 104.75 | 74.28 | 81.06 | 77.67 | 4.80 | 0.06 |
| FLT3 (Ab-599) |  | 71 | 75 | 110 | 99.5 | 104.75 | 67.61 | 78.96 | 73.28 | 8.02 | 0.11 |
| FLT3 (Phospho-Tyr599) |  | 89 | 85 | 110 | 99.5 | 104.75 | 84.75 | 89.48 | 87.12 | 3.35 | 0.04 |
| FLT3 (Phospho-Tyr842) |  | 85 | 85 | 110 | 99.5 | 104.75 | 80.94 | 89.48 | 85.21 | 6.04 | 0.07 |
| FLT3 (Phospho-Tyr969) |  | 82 | 77 | 110 | 99.5 | 104.75 | 78.09 | 81.06 | 79.57 | 2.10 | 0.03 |
| Fos (Ab-232) |  | 140 | 105 | 110 | 99.5 | 104.75 | 133.32 | 110.54 | 121.93 | 16.11 | 0.13 |
| Fos (Ab-374) |  | 299 | 400 | 110 | 99.5 | 104.75 | 284.73 | 421.11 | 352.92 | 96.43 | 0.27 |
| Fos (Phospho-Ser362) |  | 70 | 71 | 110 | 99.5 | 104.75 | 66.66 | 74.75 | 70.70 | 5.72 | 0.08 |
| Fos (Phospho-Thr232) |  | 72 | 75 | 110 | 99.5 | 104.75 | 68.56 | 78.96 | 73.76 | 7.35 | 0.10 |
| FosB (Ab-27) |  | 71 | 73 | 110 | 99.5 | 104.75 | 67.61 | 76.85 | 72.23 | 6.53 | 0.09 |
| FosB (Phospho-Ser27) |  | 64 | 168 | 110 | 99.5 | 104.75 | 60.95 | 176.86 | 118.90 | 81.97 | 0.69 |
| FOXO1/3/4-pan (Ab-24/32) |  | 111 | 103 | 110 | 99.5 | 104.75 | 105.70 | 108.43 | 107.07 | 1.93 | 0.02 |
| FOXO1/3/4-pan (Phospho-Thr24/32) |  | 59 | 60 | 110 | 99.5 | 104.75 | 56.18 | 63.17 | 59.67 | 4.94 | 0.08 |
| FOXO1A/3A (Phospho-Ser322/325) |  | 72 | 200 | 110 | 99.5 | 104.75 | 68.56 | 210.55 | 139.56 | 100.40 | 0.72 |
| FRS2 (Phospho-Tyr436) |  | 77 | 81 | 110 | 99.5 | 104.75 | 73.33 | 85.27 | 79.30 | 8.45 | 0.11 |
| Fyn (Phospho-Tyr530) |  | 60 | 63 | 110 | 99.5 | 104.75 | 57.14 | 66.32 | 61.73 | 6.50 | 0.11 |
| G3BP-1 (Ab-232) |  | 248 | 230 | 110 | 99.5 | 104.75 | 236.16 | 242.14 | 239.15 | 4.22 | 0.02 |
| G3BP-1 (Phospho-Ser232) |  | 118 | 119 | 110 | 99.5 | 104.75 | 112.37 | 125.28 | 118.82 | 9.13 | 0.08 |
| Gab1 (Ab-627) |  | 186 | 150 | 110 | 99.5 | 104.75 | 177.12 | 157.91 | 167.52 | 13.58 | 0.08 |
| Gab1 (Ab-659) |  | 72 | 143 | 110 | 99.5 | 104.75 | 68.56 | 150.55 | 109.55 | 57.97 | 0.53 |
| Gab1 (Phospho-Tyr627) |  | 294 | 236 | 110 | 99.5 | 104.75 | 279.97 | 248.45 | 264.21 | 22.29 | 0.08 |
| Gab1 (Phospho-Tyr659) |  | 69 | 91 | 110 | 99.5 | 104.75 | 65.71 | 95.80 | 80.75 | 21.28 | 0.26 |
| Gab2 (Ab-159) |  | 69 | 71 | 110 | 99.5 | 104.75 | 65.71 | 74.75 | 70.23 | 6.39 | 0.09 |
| Gab2 (Ab-623) |  | 115 | 98 | 110 | 99.5 | 104.75 | 109.51 | 103.17 | 106.34 | 4.48 | 0.04 |
| Gab2 (Phospho-Ser159) |  | 94 | 84 | 110 | 99.5 | 104.75 | 89.51 | 88.43 | 88.97 | 0.76 | 0.01 |
| Gab2 (Phospho-Tyr643) |  | 1705 | 1758 | 110 | 99.5 | 104.75 | 1623.63 | 1850.76 | 1737.19 | 160.61 | 0.09 |
| GABA-RB (Ab-434) |  | 68 | 65 | 110 | 99.5 | 104.75 | 64.75 | 68.43 | 66.59 | 2.60 | 0.04 |
| GABA-RB (Phospho-Ser434) |  | 201 | 195 | 110 | 99.5 | 104.75 | 191.41 | 205.29 | 198.35 | 9.82 | 0.05 |
| GAP43 (Ab-41) |  | 173 | 168 | 110 | 99.5 | 104.75 | 164.74 | 176.86 | 170.80 | 8.57 | 0.05 |
| GAP43 (Phospho-Ser41) |  | 118 | 102 | 110 | 99.5 | 104.75 | 112.37 | 107.38 | 109.88 | 3.53 | 0.03 |
| GAPDH |  | 205 | 201 | 110 | 99.5 | 104.75 | 195.22 | 211.61 | 203.41 | 11.59 | 0.06 |
| GATA1 (Ab-142) |  | 114 | 100 | 110 | 99.5 | 104.75 | 108.56 | 105.28 | 106.92 | 2.32 | 0.02 |
| GATA1 (Ab-310) |  | 241 | 216 | 110 | 99.5 | 104.75 | 229.50 | 227.40 | 228.45 | 1.49 | 0.01 |
| GATA1 (Phospho-Ser142) |  | 112 | 120 | 110 | 99.5 | 104.75 | 106.65 | 126.33 | 116.49 | 13.91 | 0.12 |
| GATA1 (Phospho-Ser310) |  | 119 | 156 | 110 | 99.5 | 104.75 | 113.32 | 164.23 | 138.78 | 36.00 | 0.26 |
| GluR1 (Ab-849) |  | 2710 | 2966 | 110 | 99.5 | 104.75 | 2580.66 | 3122.50 | 2851.58 | 383.14 | 0.13 |
| GluR1 (Ab-863) |  | 64 | 66 | 110 | 99.5 | 104.75 | 60.95 | 69.48 | 65.21 | 6.04 | 0.09 |
| GluR1 (Phospho-Ser849) |  | 156 | 107 | 110 | 99.5 | 104.75 | 148.55 | 112.65 | 130.60 | 25.39 | 0.19 |
| GluR1 (Phospho-Ser863) |  | 115 | 70 | 110 | 99.5 | 104.75 | 109.51 | 73.69 | 91.60 | 25.33 | 0.28 |
| GluR2 (Ab-880) |  | 1837 | 1687 | 110 | 99.5 | 104.75 | 1749.33 | 1776.01 | 1762.67 | 18.87 | 0.01 |
| GluR2 (Phospho-Ser880) |  | 94 | 91 | 110 | 99.5 | 104.75 | 89.51 | 95.80 | 92.66 | 4.45 | 0.05 |
| GRB10/Growth factor receptor-bound protein 10 (Ab-67) |  | 72 | 65 | 110 | 99.5 | 104.75 | 68.56 | 68.43 | 68.50 | 0.09 | 0.00 |
| GRB10/Growth factor receptor-bound protein 10 (Phospho-Tyr67) |  | 67 | 63 | 110 | 99.5 | 104.75 | 63.80 | 66.32 | 65.06 | 1.78 | 0.03 |
| GRF-1 (Phospho-Tyr1105) |  | 239 | 220 | 110 | 99.5 | 104.75 | 227.59 | 231.61 | 229.60 | 2.84 | 0.01 |
| GRK1 (Ab-21) |  | 73 | 75 | 110 | 99.5 | 104.75 | 69.52 | 78.96 | 74.24 | 6.68 | 0.09 |
| GRK1 (Phospho-Ser21) |  | 87 | 82 | 110 | 99.5 | 104.75 | 82.85 | 86.33 | 84.59 | 2.46 | 0.03 |
| GRK2 (Ab-29) |  | 250 | 727 | 110 | 99.5 | 104.75 | 238.07 | 765.36 | 501.71 | 372.85 | 0.74 |
| GRK2 (Phospho-Ser29) |  | 189 | 158 | 110 | 99.5 | 104.75 | 179.98 | 166.34 | 173.16 | 9.65 | 0.06 |
| GRK2 (Phospho-Ser685) |  | 70 | 74 | 110 | 99.5 | 104.75 | 66.66 | 77.90 | 72.28 | 7.95 | 0.11 |
| GSK3 alpha (Ab-21) |  | 115 | 165 | 110 | 99.5 | 104.75 | 109.51 | 173.71 | 141.61 | 45.39 | 0.32 |
| GSK3 alpha (Phospho-Ser21) |  | 508 | 92 | 110 | 99.5 | 104.75 | 483.75 | 96.85 | 290.30 | 273.58 | 0.94 |
| GSK3 alpha/beta (Ab-216/279) |  | 81 | 74 | 110 | 99.5 | 104.75 | 77.13 | 77.90 | 77.52 | 0.54 | 0.01 |
| GSK3 alpha/beta (Phospho-Tyr216/279) |  | 119 | 81 | 110 | 99.5 | 104.75 | 113.32 | 85.27 | 99.30 | 19.83 | 0.20 |
| GSK3 beta (Ab-9) |  | 107 | 96 | 110 | 99.5 | 104.75 | 101.89 | 101.07 | 101.48 | 0.59 | 0.01 |
| GSK3 beta (Phospho-Ser9) |  | 163 | 293 | 110 | 99.5 | 104.75 | 155.22 | 308.46 | 231.84 | 108.36 | 0.47 |
| GTPase activating protein (Ab-387) |  | 82 | 76 | 110 | 99.5 | 104.75 | 78.09 | 80.01 | 79.05 | 1.36 | 0.02 |
| GTPase activating protein (Phospho-Ser387) |  | 60 | 152 | 110 | 99.5 | 104.75 | 57.14 | 160.02 | 108.58 | 72.75 | 0.67 |
| HCK (Phospho-Tyr410) |  | 178 | 97 | 110 | 99.5 | 104.75 | 169.50 | 102.12 | 135.81 | 47.65 | 0.35 |
| HDAC1 (Ab-421) |  | 1298 | 5310 | 110 | 99.5 | 104.75 | 1236.05 | 5590.18 | 3413.11 | 3078.83 | 0.90 |
| HDAC1 (Phospho-Ser421) |  | 495 | 653 | 110 | 99.5 | 104.75 | 471.38 | 687.45 | 579.41 | 152.79 | 0.26 |
| HDAC2 (Ab-394) |  | 146 | 256 | 110 | 99.5 | 104.75 | 139.03 | 269.51 | 204.27 | 92.26 | 0.45 |
| HDAC2 (Phospho-Ser394) |  | 211 | 167 | 110 | 99.5 | 104.75 | 200.93 | 175.81 | 188.37 | 17.76 | 0.09 |
| HDAC3 (Ab-424) |  | 319 | 487 | 110 | 99.5 | 104.75 | 303.78 | 512.70 | 408.24 | 147.73 | 0.36 |
| HDAC3 (Phospho-Ser424) |  | 373 | 78 | 110 | 99.5 | 104.75 | 355.20 | 82.12 | 218.66 | 193.10 | 0.88 |
| HDAC4 (Ab-632) |  | 73 | 69 | 110 | 99.5 | 104.75 | 69.52 | 72.64 | 71.08 | 2.21 | 0.03 |
| HDAC4 (Phospho-Ser632) |  | 163 | 139 | 110 | 99.5 | 104.75 | 155.22 | 146.33 | 150.78 | 6.28 | 0.04 |
| HDAC5 (Ab-259) |  | 85 | 100 | 110 | 99.5 | 104.75 | 80.94 | 105.28 | 93.11 | 17.21 | 0.18 |
| HDAC5 (Ab-498) |  | 118 | 111 | 110 | 99.5 | 104.75 | 112.37 | 116.86 | 114.61 | 3.17 | 0.03 |
| HDAC5 (Phospho-Ser259) |  | 75 | 81 | 110 | 99.5 | 104.75 | 71.42 | 85.27 | 78.35 | 9.80 | 0.13 |
| HDAC5 (Phospho-Ser498) |  | 141 | 156 | 110 | 99.5 | 104.75 | 134.27 | 164.23 | 149.25 | 21.19 | 0.14 |
| HDAC6 (Ab-22) |  | 589 | 682 | 110 | 99.5 | 104.75 | 560.89 | 717.98 | 639.44 | 111.08 | 0.17 |
| HDAC6 (Phospho-Ser22) |  | 232 | 236 | 110 | 99.5 | 104.75 | 220.93 | 248.45 | 234.69 | 19.46 | 0.08 |
| HDAC8 (Ab-39) |  | 227 | 209 | 110 | 99.5 | 104.75 | 216.17 | 220.03 | 218.10 | 2.73 | 0.01 |
| HDAC8 (Phospho-Ser39) |  | 144 | 130 | 110 | 99.5 | 104.75 | 137.13 | 136.86 | 136.99 | 0.19 | 0.00 |
| HER2 (Ab-1112) |  | 88 | 131 | 110 | 99.5 | 104.75 | 83.80 | 137.91 | 110.86 | 38.26 | 0.35 |
| HER2 (Ab-1221/1222) |  | 748 | 665 | 110 | 99.5 | 104.75 | 712.30 | 700.09 | 706.19 | 8.64 | 0.01 |
| HER2 (Ab-1248) |  | 239 | 229 | 110 | 99.5 | 104.75 | 227.59 | 241.08 | 234.34 | 9.54 | 0.04 |
| HER2 (Ab-686) |  | 79 | 93 | 110 | 99.5 | 104.75 | 75.23 | 97.91 | 86.57 | 16.04 | 0.19 |
| HER2 (Ab-877) |  | 88 | 91 | 110 | 99.5 | 104.75 | 83.80 | 95.80 | 89.80 | 8.49 | 0.09 |
| HER2 (Phospho-Thr686) |  | 79 | 139 | 110 | 99.5 | 104.75 | 75.23 | 146.33 | 110.78 | 50.28 | 0.45 |
| HER2 (Phospho-Tyr1221/Tyr1222) |  | 127 | 536 | 110 | 99.5 | 104.75 | 120.94 | 564.28 | 342.61 | 313.49 | 0.92 |
| HER2 (Phospho-Tyr1248) |  | 133 | 99 | 110 | 99.5 | 104.75 | 126.65 | 104.22 | 115.44 | 15.86 | 0.14 |
| HER2 (Phospho-Tyr877) |  | 136 | 139 | 110 | 99.5 | 104.75 | 129.51 | 146.33 | 137.92 | 11.90 | 0.09 |
| HER3/ErbB3 (Ab-1222) |  | 85 | 93 | 110 | 99.5 | 104.75 | 80.94 | 97.91 | 89.43 | 12.00 | 0.13 |
| HER3/ErbB3 (Ab-1289) |  | 100 | 105 | 110 | 99.5 | 104.75 | 95.23 | 110.54 | 102.88 | 10.83 | 0.11 |
| HER3/ErbB3 (Phospho-Tyr1222) |  | 78 | 78 | 110 | 99.5 | 104.75 | 74.28 | 82.12 | 78.20 | 5.54 | 0.07 |
| HER3/ErbB3 (Phospho-Tyr1289) |  | 80 | 78 | 110 | 99.5 | 104.75 | 76.18 | 82.12 | 79.15 | 4.20 | 0.05 |
| HER4/ErbB4 (Ab-1284) |  | 403 | 685 | 110 | 99.5 | 104.75 | 383.77 | 721.14 | 552.45 | 238.56 | 0.43 |
| HER4/ErbB4 (Phospho-Tyr1284) |  | 455 | 443 | 110 | 99.5 | 104.75 | 433.28 | 466.37 | 449.83 | 23.40 | 0.05 |
| Histone H2A.X (Ab-139) |  | 407 | 491 | 110 | 99.5 | 104.75 | 387.58 | 516.91 | 452.24 | 91.45 | 0.20 |
| Histone H2A.X (Phospho-Ser139) |  | 215 | 404 | 110 | 99.5 | 104.75 | 204.74 | 425.32 | 315.03 | 155.97 | 0.50 |
| Histone H3.1 (Ab-10) |  | 242 | 236 | 110 | 99.5 | 104.75 | 230.45 | 248.45 | 239.45 | 12.73 | 0.05 |
| Histone H3.1 (Phospho-Ser10) |  | 110 | 61 | 110 | 99.5 | 104.75 | 104.75 | 64.22 | 84.48 | 28.66 | 0.34 |
| HNF4 alpha (Ab-313) |  | 92 | 82 | 110 | 99.5 | 104.75 | 87.61 | 86.33 | 86.97 | 0.91 | 0.01 |
| HNF4 alpha (Phospho-Ser313) |  | 115 | 101 | 110 | 99.5 | 104.75 | 109.51 | 106.33 | 107.92 | 2.25 | 0.02 |
| HRS (Ab-334) |  | 84 | 82 | 110 | 99.5 | 104.75 | 79.99 | 86.33 | 83.16 | 4.48 | 0.05 |
| HRS (Phospho-Tyr334) |  | 89 | 87 | 110 | 99.5 | 104.75 | 84.75 | 91.59 | 88.17 | 4.84 | 0.05 |
| HSF1 (Ab-303) |  | 79 | 73 | 110 | 99.5 | 104.75 | 75.23 | 76.85 | 76.04 | 1.15 | 0.02 |
| HSF1 (Phospho-Ser303) |  | 85 | 80 | 110 | 99.5 | 104.75 | 80.94 | 84.22 | 82.58 | 2.32 | 0.03 |
| HSL (Ab-552/563) |  | 146 | 164 | 110 | 99.5 | 104.75 | 139.03 | 172.65 | 155.84 | 23.77 | 0.15 |
| HSL (Ab-554) |  | 74 | 215 | 110 | 99.5 | 104.75 | 70.47 | 226.34 | 148.41 | 110.22 | 0.74 |
| HSL (Phospho-Ser552/563) |  | 81 | 83 | 110 | 99.5 | 104.75 | 77.13 | 87.38 | 82.26 | 7.24 | 0.09 |
| HSL (Phospho-Ser554) |  | 92 | 65 | 110 | 99.5 | 104.75 | 87.61 | 68.43 | 78.02 | 13.56 | 0.17 |
| HSP27 (Ab-15) |  | 147 | 127 | 110 | 99.5 | 104.75 | 139.98 | 133.70 | 136.84 | 4.44 | 0.03 |
| HSP27 (Ab-78) |  | 65 | 66 | 110 | 99.5 | 104.75 | 61.90 | 69.48 | 65.69 | 5.36 | 0.08 |
| HSP27 (Ab-82) |  | 126 | 91 | 110 | 99.5 | 104.75 | 119.99 | 95.80 | 107.89 | 17.10 | 0.16 |
| HSP27 (Phospho-Ser15) |  | 479 | 174 | 110 | 99.5 | 104.75 | 456.14 | 183.18 | 319.66 | 193.01 | 0.60 |
| HSP27 (Phospho-Ser78) |  | 78 | 73 | 110 | 99.5 | 104.75 | 74.28 | 76.85 | 75.56 | 1.82 | 0.02 |
| HSP27 (Phospho-Ser82) |  | 305 | 78 | 110 | 99.5 | 104.75 | 290.44 | 82.12 | 186.28 | 147.31 | 0.79 |
| HSP90 co-chaperone Cdc37 (Ab-13) |  | 91 | 81 | 110 | 99.5 | 104.75 | 86.66 | 85.27 | 85.97 | 0.98 | 0.01 |
| HSP90 co-chaperone Cdc37 (Phospho-Ser13) |  | 72 | 66 | 110 | 99.5 | 104.75 | 68.56 | 69.48 | 69.02 | 0.65 | 0.01 |
| HSP90B (Ab-226) |  | 76 | 71 | 110 | 99.5 | 104.75 | 72.37 | 74.75 | 73.56 | 1.68 | 0.02 |
| HSP90B (Ab-254) |  | 88 | 140 | 110 | 99.5 | 104.75 | 83.80 | 147.39 | 115.59 | 44.96 | 0.39 |
| HSP90B (Phospho-Ser226) |  | 1059 | 149 | 110 | 99.5 | 104.75 | 1008.46 | 156.86 | 582.66 | 602.17 | 1.03 |
| HSP90B (Phospho-Ser254) |  | 80 | 74 | 110 | 99.5 | 104.75 | 76.18 | 77.90 | 77.04 | 1.22 | 0.02 |
| ICAM-1 (Ab-512) |  | 76 | 72 | 110 | 99.5 | 104.75 | 72.37 | 75.80 | 74.09 | 2.42 | 0.03 |
| ICAM-1 (Phospho-Tyr512) |  | 101 | 90 | 110 | 99.5 | 104.75 | 96.18 | 94.75 | 95.46 | 1.01 | 0.01 |
| ICK (Phospho-Tyr159) |  | 110 | 101 | 110 | 99.5 | 104.75 | 104.75 | 106.33 | 105.54 | 1.12 | 0.01 |
| IGF1R (Ab-1161) |  | 73 | 68 | 110 | 99.5 | 104.75 | 69.52 | 71.59 | 70.55 | 1.47 | 0.02 |
| IGF1R (Ab-1165/1166) |  | 73 | 69 | 110 | 99.5 | 104.75 | 69.52 | 72.64 | 71.08 | 2.21 | 0.03 |
| IGF1R (Phospho-Tyr1161) |  | 146 | 114 | 110 | 99.5 | 104.75 | 139.03 | 120.02 | 129.52 | 13.45 | 0.10 |
| IGF1R (Phospho-Tyr1165/1166) |  | 183 | 140 | 110 | 99.5 | 104.75 | 174.27 | 147.39 | 160.83 | 19.01 | 0.12 |
| IGF2R (Ab-2409) |  | 86 | 94 | 110 | 99.5 | 104.75 | 81.90 | 98.96 | 90.43 | 12.07 | 0.13 |
| IGF2R (Phospho-Ser2409) |  | 72 | 74 | 110 | 99.5 | 104.75 | 68.56 | 77.90 | 73.23 | 6.61 | 0.09 |
| IGFBP-3 (Ab-183) |  | 107 | 113 | 110 | 99.5 | 104.75 | 101.89 | 118.96 | 110.43 | 12.07 | 0.11 |
| IkB-alpha (Ab-32/36) |  | 125 | 85 | 110 | 99.5 | 104.75 | 119.03 | 89.48 | 104.26 | 20.89 | 0.20 |
| IkB-alpha (Ab-42) |  | 208 | 118 | 110 | 99.5 | 104.75 | 198.07 | 124.23 | 161.15 | 52.22 | 0.32 |
| IkB-alpha (Phospho-Ser32/36) |  | 1602 | 1582 | 110 | 99.5 | 104.75 | 1525.54 | 1665.47 | 1595.51 | 98.95 | 0.06 |
| IkB-alpha (Phospho-Tyr305) |  | 59 | 63 | 110 | 99.5 | 104.75 | 56.18 | 66.32 | 61.25 | 7.17 | 0.12 |
| IkB-alpha (Phospho-Tyr42) |  | 72 | 74 | 110 | 99.5 | 104.75 | 68.56 | 77.90 | 73.23 | 6.61 | 0.09 |
| IkB-beta (Ab-19) |  | 82 | 79 | 110 | 99.5 | 104.75 | 78.09 | 83.17 | 80.63 | 3.59 | 0.04 |
| IkB-beta (Phospho-Ser23) |  | 86 | 72 | 110 | 99.5 | 104.75 | 81.90 | 75.80 | 78.85 | 4.31 | 0.05 |
| IkB-beta (Phospho-Thr19) |  | 234 | 207 | 110 | 99.5 | 104.75 | 222.83 | 217.92 | 220.38 | 3.47 | 0.02 |
| IkB-epsilon (Ab-22) |  | 1375 | 1503 | 110 | 99.5 | 104.75 | 1309.38 | 1582.30 | 1445.84 | 192.99 | 0.13 |
| IkB-epsilon (Phospho-Ser22) |  | 384 | 124 | 110 | 99.5 | 104.75 | 365.67 | 130.54 | 248.11 | 166.26 | 0.67 |
| IKK-alpha (Ab-23) |  | 258 | 226 | 110 | 99.5 | 104.75 | 245.69 | 237.92 | 241.81 | 5.49 | 0.02 |
| IKK-alpha (Phospho-Thr23) |  | 118 | 115 | 110 | 99.5 | 104.75 | 112.37 | 121.07 | 116.72 | 6.15 | 0.05 |
| IKK-alpha/beta (Ab-176/177) |  | 1565 | 2027 | 110 | 99.5 | 104.75 | 1490.31 | 2133.95 | 1812.13 | 455.13 | 0.25 |
| IKK-alpha/beta (Ab-180/181) |  | 328 | 84 | 110 | 99.5 | 104.75 | 312.35 | 88.43 | 200.39 | 158.33 | 0.79 |
| IKK-alpha/beta (Phospho-Ser180/181) |  | 63 | 67 | 110 | 99.5 | 104.75 | 59.99 | 70.54 | 65.26 | 7.45 | 0.11 |
| IKK-beta (Ab-188) |  | 253 | 206 | 110 | 99.5 | 104.75 | 240.93 | 216.87 | 228.90 | 17.01 | 0.07 |
| IKK-beta (Ab-199) |  | 715 | 855 | 110 | 99.5 | 104.75 | 680.88 | 900.11 | 790.49 | 155.02 | 0.20 |
| IKK-beta (Phospho-Tyr188) |  | 68 | 64 | 110 | 99.5 | 104.75 | 64.75 | 67.38 | 66.07 | 1.85 | 0.03 |
| IKK-beta (Phospho-Tyr199) |  | 178 | 70 | 110 | 99.5 | 104.75 | 169.50 | 73.69 | 121.60 | 67.75 | 0.56 |
| IKK-gamma (Ab-31) |  | 761 | 1328 | 110 | 99.5 | 104.75 | 724.68 | 1398.07 | 1061.37 | 476.16 | 0.45 |
| IKK-gamma (Ab-85) |  | 159 | 147 | 110 | 99.5 | 104.75 | 151.41 | 154.76 | 153.08 | 2.37 | 0.02 |
| IKK-gamma (Phospho-Ser31) |  | 108 | 73 | 110 | 99.5 | 104.75 | 102.85 | 76.85 | 89.85 | 18.38 | 0.20 |
| IKK-gamma (Phospho-Ser85) |  | 166 | 148 | 110 | 99.5 | 104.75 | 158.08 | 155.81 | 156.94 | 1.60 | 0.01 |
| IL-10R-alpha (Ab-496) |  | 149 | 160 | 110 | 99.5 | 104.75 | 141.89 | 168.44 | 155.17 | 18.78 | 0.12 |
| IL-10R-alpha (Phospho-Tyr496) |  | 127 | 467 | 110 | 99.5 | 104.75 | 120.94 | 491.64 | 306.29 | 262.13 | 0.86 |
| IL-13R/CD213a1 (Ab-405) |  | 100 | 87 | 110 | 99.5 | 104.75 | 95.23 | 91.59 | 93.41 | 2.57 | 0.03 |
| IL-13R/CD213a1 (Phospho-Tyr405) |  | 214 | 1019 | 110 | 99.5 | 104.75 | 203.79 | 1072.77 | 638.28 | 614.46 | 0.96 |
| IL-2RA/CD25 (Ab-268) |  | 70 | 72 | 110 | 99.5 | 104.75 | 66.66 | 75.80 | 71.23 | 6.46 | 0.09 |
| IL-2RA/CD25 (Phospho-Ser268) |  | 63 | 212 | 110 | 99.5 | 104.75 | 59.99 | 223.19 | 141.59 | 115.39 | 0.81 |
| IL3RB (Ab-593) |  | 89 | 76 | 110 | 99.5 | 104.75 | 84.75 | 80.01 | 82.38 | 3.35 | 0.04 |
| IL3RB (Phospho-Tyr593) |  | 274 | 217 | 110 | 99.5 | 104.75 | 260.92 | 228.45 | 244.69 | 22.96 | 0.09 |
| IL-4R/CD124 (Ab-497) |  | 170 | 153 | 110 | 99.5 | 104.75 | 161.89 | 161.07 | 161.48 | 0.58 | 0.00 |
| IL-4R/CD124 (Phospho-Tyr497) |  | 105 | 99 | 110 | 99.5 | 104.75 | 99.99 | 104.22 | 102.11 | 2.99 | 0.03 |
| IL7R/CD127 (Phospho-Tyr449) |  | 288 | 397 | 110 | 99.5 | 104.75 | 274.25 | 417.95 | 346.10 | 101.61 | 0.29 |
| Integrin beta-1 (Ab-788) |  | 200 | 197 | 110 | 99.5 | 104.75 | 190.45 | 207.39 | 198.92 | 11.98 | 0.06 |
| Integrin beta-1 (Ab-789) |  | 351 | 533 | 110 | 99.5 | 104.75 | 334.25 | 561.12 | 447.69 | 160.43 | 0.36 |
| Integrin beta-1 (Phospho-Thr788) |  | 101 | 80 | 110 | 99.5 | 104.75 | 96.18 | 84.22 | 90.20 | 8.46 | 0.09 |
| Integrin beta-3 (Ab-773) |  | 126 | 117 | 110 | 99.5 | 104.75 | 119.99 | 123.17 | 121.58 | 2.25 | 0.02 |
| Integrin beta-3 (Ab-785) |  | 81 | 81 | 110 | 99.5 | 104.75 | 77.13 | 85.27 | 81.20 | 5.76 | 0.07 |
| Integrin beta-3 (Phospho-Tyr773) |  | 135 | 194 | 110 | 99.5 | 104.75 | 128.56 | 204.24 | 166.40 | 53.51 | 0.32 |
| Integrin beta-3 (Phospho-Tyr785) |  | 82 | 323 | 110 | 99.5 | 104.75 | 78.09 | 340.04 | 209.06 | 185.23 | 0.89 |
| Integrin beta-4 (Ab-1510) |  | 73 | 72 | 110 | 99.5 | 104.75 | 69.52 | 75.80 | 72.66 | 4.44 | 0.06 |
| Integrin beta-4 (Phospho-Tyr1510) |  | 88 | 84 | 110 | 99.5 | 104.75 | 83.80 | 88.43 | 86.12 | 3.28 | 0.04 |
| Interferon-alpha/beta receptor alpha chain (Ab-466) |  | 244 | 270 | 110 | 99.5 | 104.75 | 232.35 | 284.25 | 258.30 | 36.69 | 0.14 |
| Interferon-alpha/beta receptor alpha chain (Phospho-Tyr466) |  | 91 | 93 | 110 | 99.5 | 104.75 | 86.66 | 97.91 | 92.28 | 7.96 | 0.09 |
| Interferon-gamma receptor alpha chain precursor (Ab-457) |  | 84 | 86 | 110 | 99.5 | 104.75 | 79.99 | 90.54 | 85.26 | 7.46 | 0.09 |
| Interferon-gamma receptor alpha chain precursor (Phospho-Tyr457) |  | 119 | 84 | 110 | 99.5 | 104.75 | 113.32 | 88.43 | 100.88 | 17.60 | 0.17 |
| IR (Ab-1361) |  | 86 | 81 | 110 | 99.5 | 104.75 | 81.90 | 85.27 | 83.58 | 2.39 | 0.03 |
| IR (Phospho-Tyr1355) |  | 87 | 91 | 110 | 99.5 | 104.75 | 82.85 | 95.80 | 89.32 | 9.16 | 0.10 |
| IR (Phospho-Tyr1361) |  | 127 | 90 | 110 | 99.5 | 104.75 | 120.94 | 94.75 | 107.84 | 18.52 | 0.17 |
| IRS-1 (Ab-307) |  | 135 | 122 | 110 | 99.5 | 104.75 | 128.56 | 128.44 | 128.50 | 0.08 | 0.00 |
| IRS-1 (Ab-312) |  | 276 | 192 | 110 | 99.5 | 104.75 | 262.83 | 202.13 | 232.48 | 42.92 | 0.18 |
| IRS-1 (Ab-323) |  | 163 | 101 | 110 | 99.5 | 104.75 | 155.22 | 106.33 | 130.77 | 34.57 | 0.26 |
| IRS-1 (Ab-636) |  | 91 | 80 | 110 | 99.5 | 104.75 | 86.66 | 84.22 | 85.44 | 1.72 | 0.02 |
| IRS-1 (Ab-639) |  | 953 | 203 | 110 | 99.5 | 104.75 | 907.52 | 213.71 | 560.61 | 490.59 | 0.88 |
| IRS-1 (Ab-794) |  | 70 | 67 | 110 | 99.5 | 104.75 | 66.66 | 70.54 | 68.60 | 2.74 | 0.04 |
| IRS-1 (Phospho-Ser1101) |  | 99 | 106 | 110 | 99.5 | 104.75 | 94.28 | 111.59 | 102.93 | 12.25 | 0.12 |
| IRS-1 (Phospho-Ser307) |  | 99 | 86 | 110 | 99.5 | 104.75 | 94.28 | 90.54 | 92.41 | 2.64 | 0.03 |
| IRS-1 (Phospho-Ser312) |  | 120 | 79 | 110 | 99.5 | 104.75 | 114.27 | 83.17 | 98.72 | 21.99 | 0.22 |
| IRS-1 (Phospho-Ser323) |  | 63 | 60 | 110 | 99.5 | 104.75 | 59.99 | 63.17 | 61.58 | 2.24 | 0.04 |
| IRS-1 (Phospho-Ser612) |  | 222 | 62 | 110 | 99.5 | 104.75 | 211.40 | 65.27 | 138.34 | 103.33 | 0.75 |
| IRS-1 (Phospho-Ser636) |  | 139 | 67 | 110 | 99.5 | 104.75 | 132.37 | 70.54 | 101.45 | 43.72 | 0.43 |
| IRS-1 (Phospho-Ser639) |  | 236 | 62 | 110 | 99.5 | 104.75 | 224.74 | 65.27 | 145.00 | 112.76 | 0.78 |
| IRS-1 (Phospho-Ser794) |  | 70 | 69 | 110 | 99.5 | 104.75 | 66.66 | 72.64 | 69.65 | 4.23 | 0.06 |
| JAK1 (Ab-1022) |  | 147 | 137 | 110 | 99.5 | 104.75 | 139.98 | 144.23 | 142.11 | 3.00 | 0.02 |
| JAK1 (Phospho-Tyr1022) |  | 74 | 75 | 110 | 99.5 | 104.75 | 70.47 | 78.96 | 74.71 | 6.00 | 0.08 |
| JAK2 (Ab-1007) |  | 347 | 378 | 110 | 99.5 | 104.75 | 330.44 | 397.94 | 364.19 | 47.73 | 0.13 |
| JAK2 (Ab-221) |  | 144 | 112 | 110 | 99.5 | 104.75 | 137.13 | 117.91 | 127.52 | 13.59 | 0.11 |
| JAK2 (Phospho-Tyr1007) |  | 68 | 85 | 110 | 99.5 | 104.75 | 64.75 | 89.48 | 77.12 | 17.49 | 0.23 |
| JAK2 (Phospho-Tyr221) |  | 79 | 76 | 110 | 99.5 | 104.75 | 75.23 | 80.01 | 77.62 | 3.38 | 0.04 |
| JNK1/2/3 (Ab-183/185) |  | 83 | 86 | 110 | 99.5 | 104.75 | 79.04 | 90.54 | 84.79 | 8.13 | 0.10 |
| JNK1/2/3 (Phospho-Thr183/Tyr185) |  | 86 | 89 | 110 | 99.5 | 104.75 | 81.90 | 93.70 | 87.80 | 8.34 | 0.10 |
| JunB (Ab-259) |  | 164 | 178 | 110 | 99.5 | 104.75 | 156.17 | 187.39 | 171.78 | 22.08 | 0.13 |
| JunB (Ab-79) |  | 150 | 232 | 110 | 99.5 | 104.75 | 142.84 | 244.24 | 193.54 | 71.70 | 0.37 |
| JunB (Phospho-Ser259) |  | 111 | 361 | 110 | 99.5 | 104.75 | 105.70 | 380.05 | 242.88 | 193.99 | 0.80 |
| JunB (Phospho-Ser79) |  | 131 | 407 | 110 | 99.5 | 104.75 | 124.75 | 428.47 | 276.61 | 214.77 | 0.78 |
| JunD (Ab-255) |  | 108 | 87 | 110 | 99.5 | 104.75 | 102.85 | 91.59 | 97.22 | 7.96 | 0.08 |
| JunD (Phospho-Ser255) |  | 140 | 120 | 110 | 99.5 | 104.75 | 133.32 | 126.33 | 129.82 | 4.94 | 0.04 |
| Keratin 18 (Ab-33) |  | 106 | 90 | 110 | 99.5 | 104.75 | 100.94 | 94.75 | 97.84 | 4.38 | 0.04 |
| Keratin 18 (Ab-52) |  | 74 | 80 | 110 | 99.5 | 104.75 | 70.47 | 84.22 | 77.34 | 9.72 | 0.13 |
| Keratin 18 (Phospho-Ser33) |  | 70 | 67 | 110 | 99.5 | 104.75 | 66.66 | 70.54 | 68.60 | 2.74 | 0.04 |
| Keratin 18 (Phospho-Ser52) |  | 173 | 145 | 110 | 99.5 | 104.75 | 164.74 | 152.65 | 158.70 | 8.55 | 0.05 |
| Keratin 8 (Ab-431) |  | 136 | 234 | 110 | 99.5 | 104.75 | 129.51 | 246.35 | 187.93 | 82.62 | 0.44 |
| Keratin 8 (Ab-73) |  | 104 | 97 | 110 | 99.5 | 104.75 | 99.04 | 102.12 | 100.58 | 2.18 | 0.02 |
| Keratin 8 (Phospho-Ser431) |  | 240 | 242 | 110 | 99.5 | 104.75 | 228.55 | 254.77 | 241.66 | 18.54 | 0.08 |
| Keratin 8 (Phospho-Ser73) |  | 69 | 64 | 110 | 99.5 | 104.75 | 65.71 | 67.38 | 66.54 | 1.18 | 0.02 |
| KIT (Ab-721) |  | 90 | 104 | 110 | 99.5 | 104.75 | 85.70 | 109.49 | 97.60 | 16.82 | 0.17 |
| KIT (Ab-936) |  | 85 | 80 | 110 | 99.5 | 104.75 | 80.94 | 84.22 | 82.58 | 2.32 | 0.03 |
| KIT (Phospho-Tyr703) |  | 66 | 69 | 110 | 99.5 | 104.75 | 62.85 | 72.64 | 67.75 | 6.92 | 0.10 |
| KIT (Phospho-Tyr721) |  | 284 | 1020 | 110 | 99.5 | 104.75 | 270.45 | 1073.82 | 672.13 | 568.07 | 0.85 |
| KIT (Phospho-Tyr936) |  | 119 | 131 | 110 | 99.5 | 104.75 | 113.32 | 137.91 | 125.62 | 17.39 | 0.14 |
| KSR (Ab-392) |  | 79 | 84 | 110 | 99.5 | 104.75 | 75.23 | 88.43 | 81.83 | 9.34 | 0.11 |
| KSR (Phospho-Ser392) |  | 61 | 223 | 110 | 99.5 | 104.75 | 58.09 | 234.77 | 146.43 | 124.93 | 0.85 |
| Kv1.3/KCNA3 (Ab-135) |  | 150 | 137 | 110 | 99.5 | 104.75 | 142.84 | 144.23 | 143.53 | 0.98 | 0.01 |
| Kv1.3/KCNA3 (Phospho-Tyr135) |  | 167 | 142 | 110 | 99.5 | 104.75 | 159.03 | 149.49 | 154.26 | 6.74 | 0.04 |
| Kv2.1/Kcnb1 (Phospho-Tyr128) |  | 371 | 438 | 110 | 99.5 | 104.75 | 353.29 | 461.11 | 407.20 | 76.24 | 0.19 |
| Lamin A (Ab-22) |  | 72 | 70 | 110 | 99.5 | 104.75 | 68.56 | 73.69 | 71.13 | 3.63 | 0.05 |
| Lamin A (Phospho-Ser22) |  | 363 | 246 | 110 | 99.5 | 104.75 | 345.68 | 258.98 | 302.33 | 61.30 | 0.20 |
| Lamin A/C (Ab-392) |  | 2806 | 3500 | 110 | 99.5 | 104.75 | 2672.08 | 3684.67 | 3178.38 | 716.01 | 0.23 |
| Lamin A/C (Phospho-Ser392) |  | 72 | 74 | 110 | 99.5 | 104.75 | 68.56 | 77.90 | 73.23 | 6.61 | 0.09 |
| LAT (Ab-161) |  | 83 | 81 | 110 | 99.5 | 104.75 | 79.04 | 85.27 | 82.16 | 4.41 | 0.05 |
| LAT (Ab-171) |  | 361 | 292 | 110 | 99.5 | 104.75 | 343.77 | 307.41 | 325.59 | 25.71 | 0.08 |
| LAT (Ab-191) |  | 237 | 222 | 110 | 99.5 | 104.75 | 225.69 | 233.71 | 229.70 | 5.67 | 0.02 |
| LAT (Phospho-Tyr171) |  | 69 | 70 | 110 | 99.5 | 104.75 | 65.71 | 73.69 | 69.70 | 5.65 | 0.08 |
| LAT (Phospho-Tyr191) |  | 205 | 312 | 110 | 99.5 | 104.75 | 195.22 | 328.46 | 261.84 | 94.22 | 0.36 |
| LCK (Ab-192) |  | 103 | 92 | 110 | 99.5 | 104.75 | 98.08 | 96.85 | 97.47 | 0.87 | 0.01 |
| LCK (Ab-393) |  | 2711 | 1177 | 110 | 99.5 | 104.75 | 2581.61 | 1239.10 | 1910.36 | 949.30 | 0.50 |
| LCK (Ab-504) |  | 122 | 125 | 110 | 99.5 | 104.75 | 116.18 | 131.60 | 123.89 | 10.90 | 0.09 |
| LCK (Ab-59) |  | 72 | 73 | 110 | 99.5 | 104.75 | 68.56 | 76.85 | 72.71 | 5.86 | 0.08 |
| LCK (Phospho-Ser59) |  | 80 | 115 | 110 | 99.5 | 104.75 | 76.18 | 121.07 | 98.62 | 31.74 | 0.32 |
| LCK (Phospho-Tyr192) |  | 68 | 63 | 110 | 99.5 | 104.75 | 64.75 | 66.32 | 65.54 | 1.11 | 0.02 |
| LCK (Phospho-Tyr393) |  | 156 | 146 | 110 | 99.5 | 104.75 | 148.55 | 153.70 | 151.13 | 3.64 | 0.02 |
| LCK (Phospho-Tyr504) |  | 197 | 66 | 110 | 99.5 | 104.75 | 187.60 | 69.48 | 128.54 | 83.52 | 0.65 |
| LIMK1 (Ab-508) |  | 370 | 302 | 110 | 99.5 | 104.75 | 352.34 | 317.93 | 335.14 | 24.33 | 0.07 |
| LIMK1 (Phospho-Thr508) |  | 91 | 86 | 110 | 99.5 | 104.75 | 86.66 | 90.54 | 88.60 | 2.74 | 0.03 |
| LIMK1/2 (Ab-508/505) |  | 698 | 356 | 110 | 99.5 | 104.75 | 664.69 | 374.78 | 519.74 | 204.99 | 0.39 |
| LKB1 (Ab-189) |  | 96 | 89 | 110 | 99.5 | 104.75 | 91.42 | 93.70 | 92.56 | 1.61 | 0.02 |
| LKB1 (Ab-334) |  | 295 | 531 | 110 | 99.5 | 104.75 | 280.92 | 559.02 | 419.97 | 196.64 | 0.47 |
| LKB1 (Ab-428) |  | 78 | 79 | 110 | 99.5 | 104.75 | 74.28 | 83.17 | 78.72 | 6.29 | 0.08 |
| LKB1 (Phospho-Ser428) |  | 157 | 89 | 110 | 99.5 | 104.75 | 149.51 | 93.70 | 121.60 | 39.46 | 0.32 |
| LKB1 (Phospho-Thr189) |  | 191 | 71 | 110 | 99.5 | 104.75 | 181.88 | 74.75 | 128.32 | 75.76 | 0.59 |
| LYN (Ab-507) |  | 2272 | 3486 | 110 | 99.5 | 104.75 | 2163.56 | 3669.93 | 2916.75 | 1065.17 | 0.37 |
| LYN (Phospho-Tyr507) |  | 129 | 100 | 110 | 99.5 | 104.75 | 122.84 | 105.28 | 114.06 | 12.42 | 0.11 |
| MAP3K1/MEKK1 (Phospho-Thr1381) |  | 94 | 131 | 110 | 99.5 | 104.75 | 89.51 | 137.91 | 113.71 | 34.22 | 0.30 |
| MAP3K7/TAK1 (Ab-184) |  | 89 | 94 | 110 | 99.5 | 104.75 | 84.75 | 98.96 | 91.86 | 10.05 | 0.11 |
| MAP3K7/TAK1 (Ab-187) |  | 94 | 97 | 110 | 99.5 | 104.75 | 89.51 | 102.12 | 95.82 | 8.91 | 0.09 |
| MAP3K7/TAK1 (Ab-439) |  | 233 | 219 | 110 | 99.5 | 104.75 | 221.88 | 230.56 | 226.22 | 6.13 | 0.03 |
| MAP3K7/TAK1 (Phospho-Thr184) |  | 116 | 82 | 110 | 99.5 | 104.75 | 110.46 | 86.33 | 98.40 | 17.07 | 0.17 |
| MAP3K8/COT (Ab-290) |  | 182 | 165 | 110 | 99.5 | 104.75 | 173.31 | 173.71 | 173.51 | 0.28 | 0.00 |
| MAP3K8/COT (Ab-400) |  | 77 | 66 | 110 | 99.5 | 104.75 | 73.33 | 69.48 | 71.40 | 2.72 | 0.04 |
| MAP3K8/COT (Phospho-Thr290) |  | 64 | 60 | 110 | 99.5 | 104.75 | 60.95 | 63.17 | 62.06 | 1.57 | 0.03 |
| MAPKAPK2 (Ab-272) |  | 106 | 107 | 110 | 99.5 | 104.75 | 100.94 | 112.65 | 106.79 | 8.28 | 0.08 |
| MAPKAPK2 (Phospho-Ser272) |  | 126 | 151 | 110 | 99.5 | 104.75 | 119.99 | 158.97 | 139.48 | 27.56 | 0.20 |
| MAPKAPK2 (Phospho-Thr222) |  | 92 | 87 | 110 | 99.5 | 104.75 | 87.61 | 91.59 | 89.60 | 2.82 | 0.03 |
| MAPKAPK2 (Phospho-Thr334) |  | 63 | 204 | 110 | 99.5 | 104.75 | 59.99 | 214.76 | 137.38 | 109.44 | 0.80 |
| MARCKS (Ab-158) |  | 238 | 154 | 110 | 99.5 | 104.75 | 226.64 | 162.13 | 194.38 | 45.62 | 0.23 |
| MARCKS (Ab-163) |  | 77 | 71 | 110 | 99.5 | 104.75 | 73.33 | 74.75 | 74.04 | 1.00 | 0.01 |
| MARCKS (Phospho-Ser158) |  | 103 | 101 | 110 | 99.5 | 104.75 | 98.08 | 106.33 | 102.21 | 5.83 | 0.06 |
| MARCKS (Phospho-Ser163) |  | 112 | 85 | 110 | 99.5 | 104.75 | 106.65 | 89.48 | 98.07 | 12.14 | 0.12 |
| M-CSF Receptor (Ab-561) |  | 87 | 85 | 110 | 99.5 | 104.75 | 82.85 | 89.48 | 86.17 | 4.69 | 0.05 |
| M-CSF Receptor (Ab-809) |  | 113 | 77 | 110 | 99.5 | 104.75 | 107.61 | 81.06 | 94.33 | 18.77 | 0.20 |
| M-CSF Receptor (Phospho-Tyr561) |  | 168 | 109 | 110 | 99.5 | 104.75 | 159.98 | 114.75 | 137.37 | 31.98 | 0.23 |
| M-CSF Receptor (Phospho-Tyr809) |  | 243 | 250 | 110 | 99.5 | 104.75 | 231.40 | 263.19 | 247.30 | 22.48 | 0.09 |
| MDM2 (Ab-166) |  | 4124 | 5218 | 110 | 99.5 | 104.75 | 3927.17 | 5493.32 | 4710.25 | 1107.43 | 0.24 |
| MDM2 (Phospho-Ser166) |  | 95 | 81 | 110 | 99.5 | 104.75 | 90.47 | 85.27 | 87.87 | 3.67 | 0.04 |
| MDM4 (Phospho-Ser367) |  | 72 | 78 | 110 | 99.5 | 104.75 | 68.56 | 82.12 | 75.34 | 9.58 | 0.13 |
| MEF2A (Ab-312) |  | 2807 | 1778 | 110 | 99.5 | 104.75 | 2673.03 | 1871.81 | 2272.42 | 566.54 | 0.25 |
| MEF2A (Ab-319) |  | 363 | 301 | 110 | 99.5 | 104.75 | 345.68 | 316.88 | 331.28 | 20.36 | 0.06 |
| MEF2A (Ab-408) |  | 455 | 470 | 110 | 99.5 | 104.75 | 433.28 | 494.80 | 464.04 | 43.50 | 0.09 |
| MEF2A (Phospho-Ser408) |  | 90 | 88 | 110 | 99.5 | 104.75 | 85.70 | 92.64 | 89.17 | 4.91 | 0.06 |
| MEF2A (Phospho-Thr312) |  | 352 | 285 | 110 | 99.5 | 104.75 | 335.20 | 300.04 | 317.62 | 24.86 | 0.08 |
| MEF2A (Phospho-Thr319) |  | 191 | 183 | 110 | 99.5 | 104.75 | 181.88 | 192.66 | 187.27 | 7.62 | 0.04 |
| MEF2C (Ab-396) |  | 99 | 93 | 110 | 99.5 | 104.75 | 94.28 | 97.91 | 96.09 | 2.57 | 0.03 |
| MEF2C (Phospho-Ser396) |  | 209 | 190 | 110 | 99.5 | 104.75 | 199.03 | 200.03 | 199.53 | 0.71 | 0.00 |
| MEF2D (Phospho-Ser444) |  | 74 | 86 | 110 | 99.5 | 104.75 | 70.47 | 90.54 | 80.50 | 14.19 | 0.18 |
| MEK1 (Ab-217) |  | 129 | 128 | 110 | 99.5 | 104.75 | 122.84 | 134.75 | 128.80 | 8.42 | 0.07 |
| MEK1 (Ab-221) |  | 85 | 80 | 110 | 99.5 | 104.75 | 80.94 | 84.22 | 82.58 | 2.32 | 0.03 |
| MEK1 (Ab-286) |  | 234 | 332 | 110 | 99.5 | 104.75 | 222.83 | 349.52 | 286.17 | 89.58 | 0.31 |
| MEK1 (Ab-291) |  | 429 | 425 | 110 | 99.5 | 104.75 | 408.53 | 447.42 | 427.97 | 27.51 | 0.06 |
| MEK1 (Ab-298) |  | 499 | 221 | 110 | 99.5 | 104.75 | 475.18 | 232.66 | 353.92 | 171.49 | 0.48 |
| MEK1 (Phospho-Ser217) |  | 470 | 64 | 110 | 99.5 | 104.75 | 447.57 | 67.38 | 257.47 | 268.84 | 1.04 |
| MEK1 (Phospho-Ser221) |  | 128 | 117 | 110 | 99.5 | 104.75 | 121.89 | 123.17 | 122.53 | 0.91 | 0.01 |
| MEK1 (Phospho-Ser298) |  | 62 | 146 | 110 | 99.5 | 104.75 | 59.04 | 153.70 | 106.37 | 66.94 | 0.63 |
| MEK1 (Phospho-Thr286) |  | 506 | 431 | 110 | 99.5 | 104.75 | 481.85 | 453.74 | 467.80 | 19.88 | 0.04 |
| MEK1 (Phospho-Thr291) |  | 114 | 157 | 110 | 99.5 | 104.75 | 108.56 | 165.28 | 136.92 | 40.11 | 0.29 |
| MEK2 (Ab-394) |  | 120 | 97 | 110 | 99.5 | 104.75 | 114.27 | 102.12 | 108.20 | 8.59 | 0.08 |
| MEK2 (Phospho-Thr394) |  | 365 | 85 | 110 | 99.5 | 104.75 | 347.58 | 89.48 | 218.53 | 182.50 | 0.84 |
| MER/SKY (Phospho-Tyr749/Tyr681) |  | 76 | 82 | 110 | 99.5 | 104.75 | 72.37 | 86.33 | 79.35 | 9.87 | 0.12 |
| Merlin (Ab-10) |  | 280 | 1600 | 110 | 99.5 | 104.75 | 266.64 | 1684.42 | 975.53 | 1002.53 | 1.03 |
| Merlin (Ab-518) |  | 81 | 75 | 110 | 99.5 | 104.75 | 77.13 | 78.96 | 78.05 | 1.29 | 0.02 |
| Merlin (Phospho-Ser10) |  | 81 | 82 | 110 | 99.5 | 104.75 | 77.13 | 86.33 | 81.73 | 6.50 | 0.08 |
| Merlin (Phospho-Ser518) |  | 81 | 77 | 110 | 99.5 | 104.75 | 77.13 | 81.06 | 79.10 | 2.78 | 0.04 |
| Met (Ab-1003) |  | 88 | 124 | 110 | 99.5 | 104.75 | 83.80 | 130.54 | 107.17 | 33.05 | 0.31 |
| Met (Ab-1234) |  | 111 | 127 | 110 | 99.5 | 104.75 | 105.70 | 133.70 | 119.70 | 19.80 | 0.17 |
| Met (Ab-1349) |  | 116 | 90 | 110 | 99.5 | 104.75 | 110.46 | 94.75 | 102.61 | 11.11 | 0.11 |
| Met (Phospho-Tyr1003) |  | 56 | 59 | 110 | 99.5 | 104.75 | 53.33 | 62.11 | 57.72 | 6.21 | 0.11 |
| Met (Phospho-Tyr1234) |  | 119 | 285 | 110 | 99.5 | 104.75 | 113.32 | 300.04 | 206.68 | 132.03 | 0.64 |
| Met (Phospho-Tyr1349) |  | 99 | 165 | 110 | 99.5 | 104.75 | 94.28 | 173.71 | 133.99 | 56.17 | 0.42 |
| Met (Phospho-Tyr1356) |  | 4431 | 547 | 110 | 99.5 | 104.75 | 4219.52 | 575.86 | 2397.69 | 2576.46 | 1.07 |
| MITF (Ab-73) |  | 519 | 510 | 110 | 99.5 | 104.75 | 494.23 | 536.91 | 515.57 | 30.18 | 0.06 |
| MITF (Phospho-Ser73) |  | 136 | 222 | 110 | 99.5 | 104.75 | 129.51 | 233.71 | 181.61 | 73.68 | 0.41 |
| MKK3/MAP2K3 (Ab-189) |  | 269 | 201 | 110 | 99.5 | 104.75 | 256.16 | 211.61 | 233.88 | 31.51 | 0.13 |
| MKK3/MAP2K3 (Ab-222) |  | 72 | 69 | 110 | 99.5 | 104.75 | 68.56 | 72.64 | 70.60 | 2.88 | 0.04 |
| MKK3/MAP2K3 (Phospho-Ser189) |  | 81 | 77 | 110 | 99.5 | 104.75 | 77.13 | 81.06 | 79.10 | 2.78 | 0.04 |
| MKK3/MAP2K3 (Phospho-Thr222) |  | 94 | 91 | 110 | 99.5 | 104.75 | 89.51 | 95.80 | 92.66 | 4.45 | 0.05 |
| MKK4/SEK1 (Ab-257) |  | 64 | 65 | 110 | 99.5 | 104.75 | 60.95 | 68.43 | 64.69 | 5.29 | 0.08 |
| MKK4/SEK1 (Ab-261) |  | 1244 | 1032 | 110 | 99.5 | 104.75 | 1184.63 | 1086.45 | 1135.54 | 69.42 | 0.06 |
| MKK4/SEK1 (Ab-80) |  | 762 | 766 | 110 | 99.5 | 104.75 | 725.63 | 806.42 | 766.02 | 57.12 | 0.07 |
| MKK4/SEK1 (Phospho-Ser257) |  | 68 | 67 | 110 | 99.5 | 104.75 | 64.75 | 70.54 | 67.64 | 4.09 | 0.06 |
| MKK4/SEK1 (Phospho-Ser80) |  | 137 | 109 | 110 | 99.5 | 104.75 | 130.46 | 114.75 | 122.61 | 11.11 | 0.09 |
| MKK4/SEK1 (Phospho-Thr261) |  | 86 | 91 | 110 | 99.5 | 104.75 | 81.90 | 95.80 | 88.85 | 9.83 | 0.11 |
| MKK6/MAP2K6 (Ab-207) |  | 587 | 649 | 110 | 99.5 | 104.75 | 558.98 | 683.24 | 621.11 | 87.86 | 0.14 |
| MKK6/MAP2K6 (Phospho-Ser207) |  | 245 | 82 | 110 | 99.5 | 104.75 | 233.31 | 86.33 | 159.82 | 103.93 | 0.65 |
| MKK7/MAP2K7 (Ab-271) |  | 122 | 108 | 110 | 99.5 | 104.75 | 116.18 | 113.70 | 114.94 | 1.75 | 0.02 |
| MKK7/MAP2K7 (Phospho-Ser271) |  | 73 | 156 | 110 | 99.5 | 104.75 | 69.52 | 164.23 | 116.87 | 66.97 | 0.57 |
| MKK7/MAP2K7 (Phospho-Thr275) |  | 202 | 203 | 110 | 99.5 | 104.75 | 192.36 | 213.71 | 203.04 | 15.10 | 0.07 |
| MKP-1 (Ab-359) |  | 117 | 111 | 110 | 99.5 | 104.75 | 111.42 | 116.86 | 114.14 | 3.85 | 0.03 |
| MKP-1 (Phospho-Ser359) |  | 71 | 69 | 110 | 99.5 | 104.75 | 67.61 | 72.64 | 70.13 | 3.56 | 0.05 |
| MKP-1/2 (Ab-296/318) |  | 209 | 223 | 110 | 99.5 | 104.75 | 199.03 | 234.77 | 216.90 | 25.27 | 0.12 |
| MKP-1/2 (Phospho-Ser296/318) |  | 279 | 231 | 110 | 99.5 | 104.75 | 265.68 | 243.19 | 254.44 | 15.91 | 0.06 |
| Mnk1 (Ab-385) |  | 90 | 98 | 110 | 99.5 | 104.75 | 85.70 | 103.17 | 94.44 | 12.35 | 0.13 |
| Mnk1 (Phospho-Thr385) |  | 96 | 102 | 110 | 99.5 | 104.75 | 91.42 | 107.38 | 99.40 | 11.29 | 0.11 |
| MSK1 (Ab-360) |  | 1605 | 2448 | 110 | 99.5 | 104.75 | 1528.40 | 2577.17 | 2052.78 | 741.59 | 0.36 |
| MSK1 (Ab-376) |  | 164 | 142 | 110 | 99.5 | 104.75 | 156.17 | 149.49 | 152.83 | 4.72 | 0.03 |
| MSK1 (Ab-581) |  | 126 | 106 | 110 | 99.5 | 104.75 | 119.99 | 111.59 | 115.79 | 5.94 | 0.05 |
| MSK1 (Phospho-Ser212) |  | 541 | 313 | 110 | 99.5 | 104.75 | 515.18 | 329.52 | 422.35 | 131.28 | 0.31 |
| MSK1 (Phospho-Ser360) |  | 166 | 274 | 110 | 99.5 | 104.75 | 158.08 | 288.46 | 223.27 | 92.19 | 0.41 |
| MSK1 (Phospho-Ser376) |  | 93 | 85 | 110 | 99.5 | 104.75 | 88.56 | 89.48 | 89.02 | 0.65 | 0.01 |
| MSK1 (Phospho-Thr581) |  | 66 | 65 | 110 | 99.5 | 104.75 | 62.85 | 68.43 | 65.64 | 3.95 | 0.06 |
| MSK2 (Phospho-Thr568) |  | 99 | 87 | 110 | 99.5 | 104.75 | 94.28 | 91.59 | 92.93 | 1.90 | 0.02 |
| Mst1/Mst2 (Ab-183) |  | 79 | 80 | 110 | 99.5 | 104.75 | 75.23 | 84.22 | 79.73 | 6.36 | 0.08 |
| Mst1/Mst2 (Phospho-Thr183) |  | 77 | 65 | 110 | 99.5 | 104.75 | 73.33 | 68.43 | 70.88 | 3.46 | 0.05 |
| mTOR (Ab-2446) |  | 61 | 61 | 110 | 99.5 | 104.75 | 58.09 | 64.22 | 61.15 | 4.33 | 0.07 |
| mTOR (Ab-2448) |  | 70 | 70 | 110 | 99.5 | 104.75 | 66.66 | 73.69 | 70.18 | 4.97 | 0.07 |
| mTOR (Ab-2481) |  | 114 | 123 | 110 | 99.5 | 104.75 | 108.56 | 129.49 | 119.02 | 14.80 | 0.12 |
| mTOR (Phospho-Ser2448) |  | 242 | 229 | 110 | 99.5 | 104.75 | 230.45 | 241.08 | 235.77 | 7.52 | 0.03 |
| mTOR (Phospho-Ser2481) |  | 125 | 130 | 110 | 99.5 | 104.75 | 119.03 | 136.86 | 127.95 | 12.60 | 0.10 |
| mTOR (Phospho-Thr2446) |  | 199 | 66 | 110 | 99.5 | 104.75 | 189.50 | 69.48 | 129.49 | 84.87 | 0.66 |
| Myc (Ab-358) |  | 3300 | 1413 | 110 | 99.5 | 104.75 | 3142.50 | 1487.56 | 2315.03 | 1170.22 | 0.51 |
| Myc (Ab-373) |  | 219 | 168 | 110 | 99.5 | 104.75 | 208.55 | 176.86 | 192.71 | 22.40 | 0.12 |
| Myc (Ab-58) |  | 91 | 63 | 110 | 99.5 | 104.75 | 86.66 | 66.32 | 76.49 | 14.38 | 0.19 |
| Myc (Ab-62) |  | 100 | 108 | 110 | 99.5 | 104.75 | 95.23 | 113.70 | 104.46 | 13.06 | 0.13 |
| Myc (Phospho-Ser373) |  | 105 | 83 | 110 | 99.5 | 104.75 | 99.99 | 87.38 | 93.68 | 8.92 | 0.10 |
| Myc (Phospho-Ser62) |  | 100 | 160 | 110 | 99.5 | 104.75 | 95.23 | 168.44 | 131.83 | 51.77 | 0.39 |
| Myc (Phospho-Thr358) |  | 423 | 92 | 110 | 99.5 | 104.75 | 402.81 | 96.85 | 249.83 | 216.34 | 0.87 |
| Myc (Phospho-Thr58) |  | 88 | 72 | 110 | 99.5 | 104.75 | 83.80 | 75.80 | 79.80 | 5.66 | 0.07 |
| Myosin regulatory light chain 2 (Ab-18) |  | 166 | 188 | 110 | 99.5 | 104.75 | 158.08 | 197.92 | 178.00 | 28.17 | 0.16 |
| Myosin regulatory light chain 2 (Phospho-Ser18) |  | 116 | 104 | 110 | 99.5 | 104.75 | 110.46 | 109.49 | 109.98 | 0.69 | 0.01 |
| MYPT1 (Phospho-Thr696) |  | 106 | 78 | 110 | 99.5 | 104.75 | 100.94 | 82.12 | 91.53 | 13.31 | 0.15 |
| MYPT1 (Phospho-Thr853) |  | 80 | 100 | 110 | 99.5 | 104.75 | 76.18 | 105.28 | 90.73 | 20.57 | 0.23 |
| MYT1 (Ab-83) |  | 252 | 312 | 110 | 99.5 | 104.75 | 239.97 | 328.46 | 284.22 | 62.57 | 0.22 |
| NFAT3 (Ab-168/170) |  | 122 | 111 | 110 | 99.5 | 104.75 | 116.18 | 116.86 | 116.52 | 0.48 | 0.00 |
| NFAT3 (Ab-676) |  | 274 | 232 | 110 | 99.5 | 104.75 | 260.92 | 244.24 | 252.58 | 11.80 | 0.05 |
| NFAT4 (Ab-165) |  | 66 | 64 | 110 | 99.5 | 104.75 | 62.85 | 67.38 | 65.11 | 3.20 | 0.05 |
| NFAT4 (Phospho-Ser165) |  | 102 | 74 | 110 | 99.5 | 104.75 | 97.13 | 77.90 | 87.52 | 13.60 | 0.16 |
| NFkB-p100/p52 (Ab-865) |  | 767 | 816 | 110 | 99.5 | 104.75 | 730.39 | 859.06 | 794.72 | 90.98 | 0.11 |
| NFkB-p100/p52 (Ab-869) |  | 168 | 335 | 110 | 99.5 | 104.75 | 159.98 | 352.68 | 256.33 | 136.26 | 0.53 |
| NFkB-p100/p52 (Phospho-Ser865) |  | 349 | 83 | 110 | 99.5 | 104.75 | 332.34 | 87.38 | 209.86 | 173.22 | 0.83 |
| NFkB-p100/p52 (Phospho-Ser869) |  | 276 | 72 | 110 | 99.5 | 104.75 | 262.83 | 75.80 | 169.31 | 132.25 | 0.78 |
| NFkB-p100/p52 (Phospho-Ser872) |  | 577 | 114 | 110 | 99.5 | 104.75 | 549.46 | 120.02 | 334.74 | 303.66 | 0.91 |
| NFkB-p105/p50 (Ab-337) |  | 118 | 109 | 110 | 99.5 | 104.75 | 112.37 | 114.75 | 113.56 | 1.69 | 0.01 |
| NFkB-p105/p50 (Ab-893) |  | 261 | 108 | 110 | 99.5 | 104.75 | 248.54 | 113.70 | 181.12 | 95.35 | 0.53 |
| NFkB-p105/p50 (Ab-907) |  | 249 | 164 | 110 | 99.5 | 104.75 | 237.12 | 172.65 | 204.88 | 45.58 | 0.22 |
| NFkB-p105/p50 (Ab-927) |  | 941 | 726 | 110 | 99.5 | 104.75 | 896.09 | 764.31 | 830.20 | 93.18 | 0.11 |
| NFkB-p105/p50 (Ab-932) |  | 80 | 78 | 110 | 99.5 | 104.75 | 76.18 | 82.12 | 79.15 | 4.20 | 0.05 |
| NFkB-p105/p50 (Phospho-Ser337) |  | 830 | 647 | 110 | 99.5 | 104.75 | 790.39 | 681.14 | 735.76 | 77.25 | 0.10 |
| NFkB-p105/p50 (Phospho-Ser893) |  | 91 | 77 | 110 | 99.5 | 104.75 | 86.66 | 81.06 | 83.86 | 3.96 | 0.05 |
| NFkB-p105/p50 (Phospho-Ser907) |  | 114 | 93 | 110 | 99.5 | 104.75 | 108.56 | 97.91 | 103.23 | 7.53 | 0.07 |
| NFkB-p105/p50 (Phospho-Ser927) |  | 66 | 93 | 110 | 99.5 | 104.75 | 62.85 | 97.91 | 80.38 | 24.79 | 0.31 |
| NFkB-p105/p50 (Phospho-Ser932) |  | 86 | 83 | 110 | 99.5 | 104.75 | 81.90 | 87.38 | 84.64 | 3.88 | 0.05 |
| NFkB-p65 (Ab-254) |  | 132 | 113 | 110 | 99.5 | 104.75 | 125.70 | 118.96 | 122.33 | 4.76 | 0.04 |
| NFkB-p65 (Ab-276) |  | 104 | 97 | 110 | 99.5 | 104.75 | 99.04 | 102.12 | 100.58 | 2.18 | 0.02 |
| NFkB-p65 (Ab-281) |  | 105 | 115 | 110 | 99.5 | 104.75 | 99.99 | 121.07 | 110.53 | 14.91 | 0.13 |
| NFkB-p65 (Ab-311) |  | 108 | 92 | 110 | 99.5 | 104.75 | 102.85 | 96.85 | 99.85 | 4.24 | 0.04 |
| NFkB-p65 (Ab-435) |  | 109 | 106 | 110 | 99.5 | 104.75 | 103.80 | 111.59 | 107.70 | 5.51 | 0.05 |
| NFkB-p65 (Ab-468) |  | 216 | 171 | 110 | 99.5 | 104.75 | 205.69 | 180.02 | 192.86 | 18.15 | 0.09 |
| NFkB-p65 (Ab-505) |  | 174 | 206 | 110 | 99.5 | 104.75 | 165.70 | 216.87 | 191.28 | 36.19 | 0.19 |
| NFkB-p65 (Ab-529) |  | 239 | 209 | 110 | 99.5 | 104.75 | 227.59 | 220.03 | 223.81 | 5.35 | 0.02 |
| NFkB-p65 (Ab-536) |  | 98 | 93 | 110 | 99.5 | 104.75 | 93.32 | 97.91 | 95.61 | 3.24 | 0.03 |
| NFkB-p65 (Phospho-Ser276) |  | 189 | 112 | 110 | 99.5 | 104.75 | 179.98 | 117.91 | 148.94 | 43.89 | 0.29 |
| NFkB-p65 (Phospho-Ser311) |  | 123 | 135 | 110 | 99.5 | 104.75 | 117.13 | 142.12 | 129.63 | 17.67 | 0.14 |
| NFkB-p65 (Phospho-Ser468) |  | 423 | 79 | 110 | 99.5 | 104.75 | 402.81 | 83.17 | 242.99 | 226.02 | 0.93 |
| NFkB-p65 (Phospho-Ser529) |  | 122 | 443 | 110 | 99.5 | 104.75 | 116.18 | 466.37 | 291.28 | 247.63 | 0.85 |
| NFkB-p65 (Phospho-Ser536) |  | 86 | 73 | 110 | 99.5 | 104.75 | 81.90 | 76.85 | 79.37 | 3.57 | 0.04 |
| NFkB-p65 (Phospho-Thr254) |  | 102 | 69 | 110 | 99.5 | 104.75 | 97.13 | 72.64 | 84.89 | 17.32 | 0.20 |
| NFkB-p65 (Phospho-Thr435) |  | 123 | 97 | 110 | 99.5 | 104.75 | 117.13 | 102.12 | 109.62 | 10.61 | 0.10 |
| NFkB-p65 (Phospho-Thr505) |  | 149 | 127 | 110 | 99.5 | 104.75 | 141.89 | 133.70 | 137.79 | 5.79 | 0.04 |
| NMDAR1 (Ab-897) |  | 104 | 89 | 110 | 99.5 | 104.75 | 99.04 | 93.70 | 96.37 | 3.78 | 0.04 |
| NMDAR1 (Phospho-Ser897) |  | 189 | 157 | 110 | 99.5 | 104.75 | 179.98 | 165.28 | 172.63 | 10.39 | 0.06 |
| NMDAR2A/B (Phospho-Tyr1246/1252) |  | 316 | 196 | 110 | 99.5 | 104.75 | 300.92 | 206.34 | 253.63 | 66.88 | 0.26 |
| NMDAR2B (Ab-1472) |  | 844 | 1245 | 110 | 99.5 | 104.75 | 803.72 | 1310.69 | 1057.20 | 358.48 | 0.34 |
| NMDAR2B (Phospho-Tyr1472) |  | 87 | 308 | 110 | 99.5 | 104.75 | 82.85 | 324.25 | 203.55 | 170.70 | 0.84 |
| Opioid Receptor (Ab-375) |  | 61 | 61 | 110 | 99.5 | 104.75 | 58.09 | 64.22 | 61.15 | 4.33 | 0.07 |
| Opioid Receptor (Phospho-Ser375) |  | 345 | 222 | 110 | 99.5 | 104.75 | 328.53 | 233.71 | 281.12 | 67.05 | 0.24 |
| p130Cas (Ab-165) |  | 91 | 84 | 110 | 99.5 | 104.75 | 86.66 | 88.43 | 87.54 | 1.26 | 0.01 |
| p130Cas (Ab-410) |  | 68 | 65 | 110 | 99.5 | 104.75 | 64.75 | 68.43 | 66.59 | 2.60 | 0.04 |
| p130Cas (Phospho-Tyr165) |  | 80 | 72 | 110 | 99.5 | 104.75 | 76.18 | 75.80 | 75.99 | 0.27 | 0.00 |
| p130Cas (Phospho-Tyr410) |  | 61 | 64 | 110 | 99.5 | 104.75 | 58.09 | 67.38 | 62.73 | 6.57 | 0.10 |
| p21Cip1 (Ab-145) |  | 105 | 107 | 110 | 99.5 | 104.75 | 99.99 | 112.65 | 106.32 | 8.95 | 0.08 |
| p21Cip1 (Phospho-Thr145) |  | 134 | 61 | 110 | 99.5 | 104.75 | 127.60 | 64.22 | 95.91 | 44.82 | 0.47 |
| p27Kip1 (Ab-10) |  | 79 | 75 | 110 | 99.5 | 104.75 | 75.23 | 78.96 | 77.09 | 2.64 | 0.03 |
| p27Kip1 (Ab-187) |  | 91 | 84 | 110 | 99.5 | 104.75 | 86.66 | 88.43 | 87.54 | 1.26 | 0.01 |
| p27Kip1 (Phospho-Ser10) |  | 282 | 74 | 110 | 99.5 | 104.75 | 268.54 | 77.90 | 173.22 | 134.80 | 0.78 |
| p27Kip1 (Phospho-Thr187) |  | 83 | 65 | 110 | 99.5 | 104.75 | 79.04 | 68.43 | 73.73 | 7.50 | 0.10 |
| p300 (Ab-89) |  | 457 | 415 | 110 | 99.5 | 104.75 | 435.19 | 436.90 | 436.04 | 1.21 | 0.00 |
| p38 MAPK (Ab-180) |  | 69 | 65 | 110 | 99.5 | 104.75 | 65.71 | 68.43 | 67.07 | 1.93 | 0.03 |
| p38 MAPK (Ab-182) |  | 2558 | 2772 | 110 | 99.5 | 104.75 | 2435.91 | 2918.26 | 2677.09 | 341.07 | 0.13 |
| p38 MAPK (Ab-322) |  | 67 | 168 | 110 | 99.5 | 104.75 | 63.80 | 176.86 | 120.33 | 79.95 | 0.66 |
| p38 MAPK (Phospho-Thr180) |  | 96 | 93 | 110 | 99.5 | 104.75 | 91.42 | 97.91 | 94.66 | 4.59 | 0.05 |
| p38 MAPK (Phospho-Tyr182) |  | 96 | 96 | 110 | 99.5 | 104.75 | 91.42 | 101.07 | 96.24 | 6.82 | 0.07 |
| p38 MAPK (Phospho-Tyr322) |  | 69 | 71 | 110 | 99.5 | 104.75 | 65.71 | 74.75 | 70.23 | 6.39 | 0.09 |
| p44/42 MAPK (Ab-202) |  | 92 | 87 | 110 | 99.5 | 104.75 | 87.61 | 91.59 | 89.60 | 2.82 | 0.03 |
| p44/42 MAPK (Ab-204) |  | 69 | 76 | 110 | 99.5 | 104.75 | 65.71 | 80.01 | 72.86 | 10.11 | 0.14 |
| p44/42 MAPK (Phospho-Thr202) |  | 147 | 136 | 110 | 99.5 | 104.75 | 139.98 | 143.18 | 141.58 | 2.26 | 0.02 |
| p44/42 MAPK (Phospho-Tyr204) |  | 108 | 226 | 110 | 99.5 | 104.75 | 102.85 | 237.92 | 170.39 | 95.52 | 0.56 |
| p53 (Ab-15) |  | 91 | 85 | 110 | 99.5 | 104.75 | 86.66 | 89.48 | 88.07 | 2.00 | 0.02 |
| p53 (Ab-18) |  | 207 | 230 | 110 | 99.5 | 104.75 | 197.12 | 242.14 | 219.63 | 31.83 | 0.14 |
| p53 (Ab-20) |  | 628 | 477 | 110 | 99.5 | 104.75 | 598.03 | 502.17 | 550.10 | 67.78 | 0.12 |
| p53 (Ab-315) |  | 490 | 704 | 110 | 99.5 | 104.75 | 466.61 | 741.15 | 603.88 | 194.12 | 0.32 |
| p53 (Ab-33) |  | 142 | 139 | 110 | 99.5 | 104.75 | 135.22 | 146.33 | 140.78 | 7.86 | 0.06 |
| p53 (Ab-37) |  | 126 | 158 | 110 | 99.5 | 104.75 | 119.99 | 166.34 | 143.16 | 32.77 | 0.23 |
| p53 (Ab-376) |  | 73 | 71 | 110 | 99.5 | 104.75 | 69.52 | 74.75 | 72.13 | 3.70 | 0.05 |
| p53 (Ab-378) |  | 63 | 65 | 110 | 99.5 | 104.75 | 59.99 | 68.43 | 64.21 | 5.97 | 0.09 |
| p53 (Ab-387) |  | 106 | 103 | 110 | 99.5 | 104.75 | 100.94 | 108.43 | 104.69 | 5.30 | 0.05 |
| p53 (Ab-392) |  | 110 | 81 | 110 | 99.5 | 104.75 | 104.75 | 85.27 | 95.01 | 13.77 | 0.14 |
| p53 (Ab-46) |  | 101 | 78 | 110 | 99.5 | 104.75 | 96.18 | 82.12 | 89.15 | 9.94 | 0.11 |
| p53 (Ab-6) |  | 73 | 73 | 110 | 99.5 | 104.75 | 69.52 | 76.85 | 73.18 | 5.19 | 0.07 |
| p53 (Ab-9) |  | 120 | 116 | 110 | 99.5 | 104.75 | 114.27 | 122.12 | 118.20 | 5.55 | 0.05 |
| p53 (Phospho-Ser15) |  | 146 | 157 | 110 | 99.5 | 104.75 | 139.03 | 165.28 | 152.16 | 18.56 | 0.12 |
| p53 (Phospho-Ser20) |  | 101 | 87 | 110 | 99.5 | 104.75 | 96.18 | 91.59 | 93.88 | 3.24 | 0.03 |
| p53 (Phospho-Ser315) |  | 163 | 156 | 110 | 99.5 | 104.75 | 155.22 | 164.23 | 159.73 | 6.37 | 0.04 |
| p53 (Phospho-Ser33) |  | 82 | 78 | 110 | 99.5 | 104.75 | 78.09 | 82.12 | 80.10 | 2.85 | 0.04 |
| p53 (Phospho-Ser366) |  | 258 | 212 | 110 | 99.5 | 104.75 | 245.69 | 223.19 | 234.44 | 15.91 | 0.07 |
| p53 (Phospho-Ser37) |  | 176 | 150 | 110 | 99.5 | 104.75 | 167.60 | 157.91 | 162.76 | 6.85 | 0.04 |
| p53 (Phospho-Ser378) |  | 63 | 63 | 110 | 99.5 | 104.75 | 59.99 | 66.32 | 63.16 | 4.48 | 0.07 |
| p53 (Phospho-Ser392) |  | 158 | 110 | 110 | 99.5 | 104.75 | 150.46 | 115.80 | 133.13 | 24.50 | 0.18 |
| p53 (Phospho-Ser46) |  | 218 | 245 | 110 | 99.5 | 104.75 | 207.60 | 257.93 | 232.76 | 35.59 | 0.15 |
| p53 (Phospho-Ser6) |  | 80 | 78 | 110 | 99.5 | 104.75 | 76.18 | 82.12 | 79.15 | 4.20 | 0.05 |
| p53 (Phospho-Ser9) |  | 78 | 73 | 110 | 99.5 | 104.75 | 74.28 | 76.85 | 75.56 | 1.82 | 0.02 |
| p53 (Phospho-Thr18) |  | 958 | 144 | 110 | 99.5 | 104.75 | 912.28 | 151.60 | 531.94 | 537.88 | 1.01 |
| p53 (Phospho-Thr81) |  | 202 | 239 | 110 | 99.5 | 104.75 | 192.36 | 251.61 | 221.98 | 41.90 | 0.19 |
| p63 (Phospho-Ser455) |  | 65 | 114 | 110 | 99.5 | 104.75 | 61.90 | 120.02 | 90.96 | 41.10 | 0.45 |
| P70S6K (Ab-229) |  | 265 | 212 | 110 | 99.5 | 104.75 | 252.35 | 223.19 | 237.77 | 20.62 | 0.09 |
| P70S6K (Ab-371) |  | 111 | 75 | 110 | 99.5 | 104.75 | 105.70 | 78.96 | 92.33 | 18.91 | 0.20 |
| P70S6K (Ab-411) |  | 73 | 64 | 110 | 99.5 | 104.75 | 69.52 | 67.38 | 68.45 | 1.51 | 0.02 |
| P70S6K (Ab-418) |  | 62 | 61 | 110 | 99.5 | 104.75 | 59.04 | 64.22 | 61.63 | 3.66 | 0.06 |
| P70S6K (Ab-421) |  | 107 | 100 | 110 | 99.5 | 104.75 | 101.89 | 105.28 | 103.58 | 2.39 | 0.02 |
| P70S6K (Ab-424) |  | 90 | 89 | 110 | 99.5 | 104.75 | 85.70 | 93.70 | 89.70 | 5.65 | 0.06 |
| P70S6K (Ab-427) |  | 64 | 63 | 110 | 99.5 | 104.75 | 60.95 | 66.32 | 63.63 | 3.80 | 0.06 |
| P70S6K (Phospho-Ser371) |  | 252 | 126 | 110 | 99.5 | 104.75 | 239.97 | 132.65 | 186.31 | 75.89 | 0.41 |
| P70S6K (Phospho-Ser411) |  | 70 | 68 | 110 | 99.5 | 104.75 | 66.66 | 71.59 | 69.12 | 3.49 | 0.05 |
| P70S6K (Phospho-Ser418) |  | 219 | 184 | 110 | 99.5 | 104.75 | 208.55 | 193.71 | 201.13 | 10.49 | 0.05 |
| P70S6K (Phospho-Ser424) |  | 86 | 79 | 110 | 99.5 | 104.75 | 81.90 | 83.17 | 82.53 | 0.90 | 0.01 |
| P70S6K (Phospho-Thr229) |  | 123 | 65 | 110 | 99.5 | 104.75 | 117.13 | 68.43 | 92.78 | 34.44 | 0.37 |
| P70S6K (Phospho-Thr389) |  | 160 | 132 | 110 | 99.5 | 104.75 | 152.36 | 138.96 | 145.66 | 9.47 | 0.07 |
| P70S6K (Phospho-Thr421) |  | 176 | 71 | 110 | 99.5 | 104.75 | 167.60 | 74.75 | 121.17 | 65.66 | 0.54 |
| P70S6K-beta (Ab-423) |  | 197 | 212 | 110 | 99.5 | 104.75 | 187.60 | 223.19 | 205.39 | 25.16 | 0.12 |
| P70S6K-beta (Phospho-Ser423) |  | 327 | 286 | 110 | 99.5 | 104.75 | 311.39 | 301.09 | 306.24 | 7.29 | 0.02 |
| P73 (Ab-99) |  | 348 | 192 | 110 | 99.5 | 104.75 | 331.39 | 202.13 | 266.76 | 91.40 | 0.34 |
| P73 (Phospho-Tyr99) |  | 112 | 111 | 110 | 99.5 | 104.75 | 106.65 | 116.86 | 111.76 | 7.21 | 0.06 |
| P90RSK (Ab-359/363) |  | 3421 | 3402 | 110 | 99.5 | 104.75 | 3257.73 | 3581.50 | 3419.61 | 228.95 | 0.07 |
| P90RSK (Ab-380) |  | 100 | 90 | 110 | 99.5 | 104.75 | 95.23 | 94.75 | 94.99 | 0.34 | 0.00 |
| P90RSK (Ab-573) |  | 901 | 936 | 110 | 99.5 | 104.75 | 858.00 | 985.39 | 921.69 | 90.08 | 0.10 |
| P90RSK (Phospho-Ser380) |  | 192 | 167 | 110 | 99.5 | 104.75 | 182.84 | 175.81 | 179.32 | 4.97 | 0.03 |
| P90RSK (Phospho-Thr359/Ser363) |  | 260 | 236 | 110 | 99.5 | 104.75 | 247.59 | 248.45 | 248.02 | 0.61 | 0.00 |
| P90RSK (Phospho-Thr573) |  | 91 | 76 | 110 | 99.5 | 104.75 | 86.66 | 80.01 | 83.33 | 4.70 | 0.06 |
| P95/NBS1 (Ab-343) |  | 1009 | 827 | 110 | 99.5 | 104.75 | 960.84 | 870.64 | 915.74 | 63.79 | 0.07 |
| P95/NBS1 (Phospho-Ser343) |  | 89 | 89 | 110 | 99.5 | 104.75 | 84.75 | 93.70 | 89.22 | 6.32 | 0.07 |
| PAK1 (Ab-204) |  | 86 | 84 | 110 | 99.5 | 104.75 | 81.90 | 88.43 | 85.16 | 4.62 | 0.05 |
| PAK1 (Ab-212) |  | 150 | 137 | 110 | 99.5 | 104.75 | 142.84 | 144.23 | 143.53 | 0.98 | 0.01 |
| PAK1 (Phospho-Ser204) |  | 290 | 266 | 110 | 99.5 | 104.75 | 276.16 | 280.04 | 278.10 | 2.74 | 0.01 |
| PAK1 (Phospho-Thr212) |  | 140 | 188 | 110 | 99.5 | 104.75 | 133.32 | 197.92 | 165.62 | 45.68 | 0.28 |
| PAK1/2 (Ab-199) |  | 105 | 101 | 110 | 99.5 | 104.75 | 99.99 | 106.33 | 103.16 | 4.48 | 0.04 |
| PAK1/2 (Phospho-Ser199) |  | 61 | 63 | 110 | 99.5 | 104.75 | 58.09 | 66.32 | 62.21 | 5.82 | 0.09 |
| PAK1/2/3 (Ab-141) |  | 97 | 92 | 110 | 99.5 | 104.75 | 92.37 | 96.85 | 94.61 | 3.17 | 0.03 |
| PAK1/2/3 (Ab-423/402/421) |  | 4781 | 5507 | 110 | 99.5 | 104.75 | 4552.82 | 5797.57 | 5175.19 | 880.17 | 0.17 |
| PAK1/2/3 (Phospho-Ser141) |  | 282 | 64 | 110 | 99.5 | 104.75 | 268.54 | 67.38 | 167.96 | 142.24 | 0.85 |
| PAK1/2/3 (Phospho-Thr423/402/421) |  | 214 | 72 | 110 | 99.5 | 104.75 | 203.79 | 75.80 | 139.79 | 90.50 | 0.65 |
| PAK2 (Ab-192) |  | 91 | 137 | 110 | 99.5 | 104.75 | 86.66 | 144.23 | 115.44 | 40.71 | 0.35 |
| PAK2 (Ab-197) |  | 160 | 168 | 110 | 99.5 | 104.75 | 152.36 | 176.86 | 164.61 | 17.32 | 0.11 |
| PAK2 (Phospho-Ser192) |  | 66 | 67 | 110 | 99.5 | 104.75 | 62.85 | 70.54 | 66.69 | 5.43 | 0.08 |
| PAK2 (Phospho-Ser20) |  | 74 | 70 | 110 | 99.5 | 104.75 | 70.47 | 73.69 | 72.08 | 2.28 | 0.03 |
| PAK3 (Ab-154) |  | 86 | 86 | 110 | 99.5 | 104.75 | 81.90 | 90.54 | 86.22 | 6.11 | 0.07 |
| PAK3 (Phospho-Ser154) |  | 73 | 68 | 110 | 99.5 | 104.75 | 69.52 | 71.59 | 70.55 | 1.47 | 0.02 |
| PAK4 (Ab-474) |  | 324 | 231 | 110 | 99.5 | 104.75 | 308.54 | 243.19 | 275.86 | 46.21 | 0.17 |
| Paxillin (Ab-118) |  | 78 | 76 | 110 | 99.5 | 104.75 | 74.28 | 80.01 | 77.14 | 4.05 | 0.05 |
| Paxillin (Ab-31) |  | 94 | 93 | 110 | 99.5 | 104.75 | 89.51 | 97.91 | 93.71 | 5.94 | 0.06 |
| Paxillin (Phospho-Tyr118) |  | 110 | 80 | 110 | 99.5 | 104.75 | 104.75 | 84.22 | 94.49 | 14.52 | 0.15 |
| Paxillin (Phospho-Tyr31) |  | 88 | 83 | 110 | 99.5 | 104.75 | 83.80 | 87.38 | 85.59 | 2.53 | 0.03 |
| PDGFR alpha (Ab-849) |  | 68 | 215 | 110 | 99.5 | 104.75 | 64.75 | 226.34 | 145.55 | 114.26 | 0.79 |
| PDGFR alpha (Phospho-Tyr849) |  | 83 | 77 | 110 | 99.5 | 104.75 | 79.04 | 81.06 | 80.05 | 1.43 | 0.02 |
| PDGFR beta (Ab-1009) |  | 92 | 93 | 110 | 99.5 | 104.75 | 87.61 | 97.91 | 92.76 | 7.28 | 0.08 |
| PDGFR beta (Ab-1021) |  | 96 | 97 | 110 | 99.5 | 104.75 | 91.42 | 102.12 | 96.77 | 7.57 | 0.08 |
| PDGFR beta (Ab-740) |  | 153 | 116 | 110 | 99.5 | 104.75 | 145.70 | 122.12 | 133.91 | 16.67 | 0.12 |
| PDGFR beta (Ab-751) |  | 275 | 386 | 110 | 99.5 | 104.75 | 261.88 | 406.37 | 334.12 | 102.17 | 0.31 |
| PDGFR beta (Phospho-Tyr1021) |  | 235 | 223 | 110 | 99.5 | 104.75 | 223.78 | 234.77 | 229.28 | 7.77 | 0.03 |
| PDGFR beta (Phospho-Tyr740) |  | 197 | 156 | 110 | 99.5 | 104.75 | 187.60 | 164.23 | 175.91 | 16.52 | 0.09 |
| PDGFR beta (Phospho-Tyr751) |  | 95 | 328 | 110 | 99.5 | 104.75 | 90.47 | 345.31 | 217.89 | 180.20 | 0.83 |
| PDK1 (Ab-241) |  | 1511 | 1278 | 110 | 99.5 | 104.75 | 1438.88 | 1345.43 | 1392.16 | 66.08 | 0.05 |
| PDK1 (Phospho-Ser241) |  | 534 | 477 | 110 | 99.5 | 104.75 | 508.51 | 502.17 | 505.34 | 4.49 | 0.01 |
| PEA-15 (Ab-116) |  | 68 | 67 | 110 | 99.5 | 104.75 | 64.75 | 70.54 | 67.64 | 4.09 | 0.06 |
| PEA-15 (Phospho-Ser104) |  | 77 | 89 | 110 | 99.5 | 104.75 | 73.33 | 93.70 | 83.51 | 14.40 | 0.17 |
| PEA-15 (Phospho-Ser116) |  | 84 | 67 | 110 | 99.5 | 104.75 | 79.99 | 70.54 | 75.26 | 6.69 | 0.09 |
| PECAM-1 (Ab-713) |  | 1884 | 2448 | 110 | 99.5 | 104.75 | 1794.08 | 2577.17 | 2185.62 | 553.72 | 0.25 |
| PECAM-1 (Phospho-Tyr713) |  | 214 | 132 | 110 | 99.5 | 104.75 | 203.79 | 138.96 | 171.38 | 45.84 | 0.27 |
| PI3-kinase p85-alpha (Phospho-Tyr607) |  | 65 | 128 | 110 | 99.5 | 104.75 | 61.90 | 134.75 | 98.33 | 51.52 | 0.52 |
| PI3-kinase p85-subunit alpha/gamma (Ab-467/199) |  | 89 | 85 | 110 | 99.5 | 104.75 | 84.75 | 89.48 | 87.12 | 3.35 | 0.04 |
| PI3-kinase p85-subunit alpha/gamma (Phospho-Tyr467/Tyr199) |  | 112 | 465 | 110 | 99.5 | 104.75 | 106.65 | 489.54 | 298.09 | 270.74 | 0.91 |
| Pim-1 (Ab-309) |  | 439 | 112 | 110 | 99.5 | 104.75 | 418.05 | 117.91 | 267.98 | 212.23 | 0.79 |
| Pim-1 (Phospho-Tyr309) |  | 81 | 80 | 110 | 99.5 | 104.75 | 77.13 | 84.22 | 80.68 | 5.01 | 0.06 |
| PIP5K (Phospho-Ser307) |  | 67 | 64 | 110 | 99.5 | 104.75 | 63.80 | 67.38 | 65.59 | 2.53 | 0.04 |
| PKA CAT (Ab-197) |  | 111 | 91 | 110 | 99.5 | 104.75 | 105.70 | 95.80 | 100.75 | 7.00 | 0.07 |
| PKA CAT (Phospho-Thr197) |  | 101 | 92 | 110 | 99.5 | 104.75 | 96.18 | 96.85 | 96.52 | 0.48 | 0.00 |
| PKA-R2B (Phospho-Ser113) |  | 214 | 199 | 110 | 99.5 | 104.75 | 203.79 | 209.50 | 206.64 | 4.04 | 0.02 |
| PKC alpha (Ab-657) |  | 71 | 68 | 110 | 99.5 | 104.75 | 67.61 | 71.59 | 69.60 | 2.81 | 0.04 |
| PKC alpha (Phospho-Tyr657) |  | 175 | 168 | 110 | 99.5 | 104.75 | 166.65 | 176.86 | 171.76 | 7.22 | 0.04 |
| PKC alpha/beta II (Ab-638) |  | 77 | 60 | 110 | 99.5 | 104.75 | 73.33 | 63.17 | 68.25 | 7.18 | 0.11 |
| PKC alpha/beta II (Phospho-Thr638) |  | 68 | 65 | 110 | 99.5 | 104.75 | 64.75 | 68.43 | 66.59 | 2.60 | 0.04 |
| PKC beta/PKCB (Ab-661) |  | 81 | 81 | 110 | 99.5 | 104.75 | 77.13 | 85.27 | 81.20 | 5.76 | 0.07 |
| PKC beta/PKCB (Phospho-Ser661) |  | 76 | 110 | 110 | 99.5 | 104.75 | 72.37 | 115.80 | 94.09 | 30.71 | 0.33 |
| PKC delta (Ab-505) |  | 85 | 64 | 110 | 99.5 | 104.75 | 80.94 | 67.38 | 74.16 | 9.59 | 0.13 |
| PKC delta (Ab-645) |  | 376 | 130 | 110 | 99.5 | 104.75 | 358.05 | 136.86 | 247.46 | 156.41 | 0.63 |
| PKC delta (Phospho-Ser645) |  | 386 | 65 | 110 | 99.5 | 104.75 | 367.58 | 68.43 | 218.00 | 211.53 | 0.97 |
| PKC delta (Phospho-Thr505) |  | 86 | 87 | 110 | 99.5 | 104.75 | 81.90 | 91.59 | 86.74 | 6.86 | 0.08 |
| PKC delta (Phospho-Tyr313) |  | 82 | 76 | 110 | 99.5 | 104.75 | 78.09 | 80.01 | 79.05 | 1.36 | 0.02 |
| PKC delta (Phospho-Tyr52) |  | 66 | 67 | 110 | 99.5 | 104.75 | 62.85 | 70.54 | 66.69 | 5.43 | 0.08 |
| PKC delta (Phospho-Tyr64) |  | 82 | 89 | 110 | 99.5 | 104.75 | 78.09 | 93.70 | 85.89 | 11.04 | 0.13 |
| PKC epsilon (Ab-729) |  | 75 | 76 | 110 | 99.5 | 104.75 | 71.42 | 80.01 | 75.72 | 6.07 | 0.08 |
| PKC epsilon (Phospho-Ser729) |  | 73 | 63 | 110 | 99.5 | 104.75 | 69.52 | 66.32 | 67.92 | 2.26 | 0.03 |
| PKC pan activation site |  | 95 | 84 | 110 | 99.5 | 104.75 | 90.47 | 88.43 | 89.45 | 1.44 | 0.02 |
| PKC pan activation site (Phospho) |  | 158 | 160 | 110 | 99.5 | 104.75 | 150.46 | 168.44 | 159.45 | 12.72 | 0.08 |
| PKC theta (Ab-676) |  | 128 | 103 | 110 | 99.5 | 104.75 | 121.89 | 108.43 | 115.16 | 9.51 | 0.08 |
| PKC theta (Phospho-Ser676) |  | 91 | 84 | 110 | 99.5 | 104.75 | 86.66 | 88.43 | 87.54 | 1.26 | 0.01 |
| PKC theta (Phospho-Thr538) |  | 95 | 66 | 110 | 99.5 | 104.75 | 90.47 | 69.48 | 79.97 | 14.84 | 0.19 |
| PKC zeta (Ab-410) |  | 101 | 95 | 110 | 99.5 | 104.75 | 96.18 | 100.01 | 98.10 | 2.71 | 0.03 |
| PKC zeta (Ab-560) |  | 94 | 79 | 110 | 99.5 | 104.75 | 89.51 | 83.17 | 86.34 | 4.49 | 0.05 |
| PKC zeta (Phospho-Thr410) |  | 623 | 620 | 110 | 99.5 | 104.75 | 593.27 | 652.71 | 622.99 | 42.04 | 0.07 |
| PKC zeta (Phospho-Thr560) |  | 156 | 174 | 110 | 99.5 | 104.75 | 148.55 | 183.18 | 165.87 | 24.48 | 0.15 |
| PKD1/PKC mu (Ab-205) |  | 87 | 100 | 110 | 99.5 | 104.75 | 82.85 | 105.28 | 94.06 | 15.86 | 0.17 |
| PKD1/PKC mu (Ab-463) |  | 198 | 215 | 110 | 99.5 | 104.75 | 188.55 | 226.34 | 207.45 | 26.72 | 0.13 |
| PKD1/PKC mu (Ab-744/748) |  | 202 | 124 | 110 | 99.5 | 104.75 | 192.36 | 130.54 | 161.45 | 43.71 | 0.27 |
| PKD1/PKC mu (Ab-910) |  | 82 | 69 | 110 | 99.5 | 104.75 | 78.09 | 72.64 | 75.36 | 3.85 | 0.05 |
| PKD1/PKC mu (Phospho-Ser205) |  | 78 | 230 | 110 | 99.5 | 104.75 | 74.28 | 242.14 | 158.21 | 118.69 | 0.75 |
| PKD1/PKC mu (Phospho-Ser910) |  | 102 | 62 | 110 | 99.5 | 104.75 | 97.13 | 65.27 | 81.20 | 22.53 | 0.28 |
| PKD1/PKC mu (Phospho-Tyr463) |  | 141 | 117 | 110 | 99.5 | 104.75 | 134.27 | 123.17 | 128.72 | 7.85 | 0.06 |
| PKD2 (Ab-876) |  | 64 | 60 | 110 | 99.5 | 104.75 | 60.95 | 63.17 | 62.06 | 1.57 | 0.03 |
| PKD2 (Phospho-Ser876) |  | 146 | 132 | 110 | 99.5 | 104.75 | 139.03 | 138.96 | 139.00 | 0.05 | 0.00 |
| PKR (Ab-446) |  | 68 | 65 | 110 | 99.5 | 104.75 | 64.75 | 68.43 | 66.59 | 2.60 | 0.04 |
| PKR (Ab-451) |  | 104 | 94 | 110 | 99.5 | 104.75 | 99.04 | 98.96 | 99.00 | 0.05 | 0.00 |
| PKR (Phospho-Thr446) |  | 69 | 71 | 110 | 99.5 | 104.75 | 65.71 | 74.75 | 70.23 | 6.39 | 0.09 |
| PKR (Phospho-Thr451) |  | 76 | 80 | 110 | 99.5 | 104.75 | 72.37 | 84.22 | 78.30 | 8.38 | 0.11 |
| PLC beta3 (Ab-1105) |  | 75 | 75 | 110 | 99.5 | 104.75 | 71.42 | 78.96 | 75.19 | 5.33 | 0.07 |
| PLC beta3 (Ab-537) |  | 81 | 70 | 110 | 99.5 | 104.75 | 77.13 | 73.69 | 75.41 | 2.43 | 0.03 |
| PLC beta3 (Phospho-Ser1105) |  | 65 | 89 | 110 | 99.5 | 104.75 | 61.90 | 93.70 | 77.80 | 22.48 | 0.29 |
| PLC beta3 (Phospho-Ser537) |  | 84 | 80 | 110 | 99.5 | 104.75 | 79.99 | 84.22 | 82.11 | 2.99 | 0.04 |
| PLCG1 (Ab-771) |  | 72 | 72 | 110 | 99.5 | 104.75 | 68.56 | 75.80 | 72.18 | 5.12 | 0.07 |
| PLCG1 (Ab-783) |  | 82 | 102 | 110 | 99.5 | 104.75 | 78.09 | 107.38 | 92.73 | 20.72 | 0.22 |
| PLCG1 (Phospho-Tyr1253) |  | 76 | 83 | 110 | 99.5 | 104.75 | 72.37 | 87.38 | 79.88 | 10.61 | 0.13 |
| PLCG1 (Phospho-Tyr771) |  | 77 | 211 | 110 | 99.5 | 104.75 | 73.33 | 222.13 | 147.73 | 105.22 | 0.71 |
| PLCG1 (Phospho-Tyr783) |  | 110 | 165 | 110 | 99.5 | 104.75 | 104.75 | 173.71 | 139.23 | 48.76 | 0.35 |
| PLCG2 (Ab-1217) |  | 125 | 120 | 110 | 99.5 | 104.75 | 119.03 | 126.33 | 122.68 | 5.16 | 0.04 |
| PLCG2 (Ab-753) |  | 236 | 264 | 110 | 99.5 | 104.75 | 224.74 | 277.93 | 251.33 | 37.61 | 0.15 |
| PLCG2 (Phospho-Tyr1217) |  | 101 | 104 | 110 | 99.5 | 104.75 | 96.18 | 109.49 | 102.83 | 9.41 | 0.09 |
| PLCG2 (Phospho-Tyr753) |  | 64 | 68 | 110 | 99.5 | 104.75 | 60.95 | 71.59 | 66.27 | 7.53 | 0.11 |
| PLD1 (Ab-561) |  | 100 | 94 | 110 | 99.5 | 104.75 | 95.23 | 98.96 | 97.09 | 2.64 | 0.03 |
| PLD1 (Phospho-Ser561) |  | 86 | 73 | 110 | 99.5 | 104.75 | 81.90 | 76.85 | 79.37 | 3.57 | 0.04 |
| PLD2 (Phospho-Tyr169) |  | 131 | 123 | 110 | 99.5 | 104.75 | 124.75 | 129.49 | 127.12 | 3.35 | 0.03 |
| PLK1 (Ab-210) |  | 164 | 190 | 110 | 99.5 | 104.75 | 156.17 | 200.03 | 178.10 | 31.01 | 0.17 |
| PLK1 (Phospho-Thr210) |  | 67 | 67 | 110 | 99.5 | 104.75 | 63.80 | 70.54 | 67.17 | 4.76 | 0.07 |
| PP1 alpha (Ab-320) |  | 149 | 164 | 110 | 99.5 | 104.75 | 141.89 | 172.65 | 157.27 | 21.75 | 0.14 |
| PP1 alpha (Phospho-Thr320) |  | 175 | 170 | 110 | 99.5 | 104.75 | 166.65 | 178.97 | 172.81 | 8.71 | 0.05 |
| PP2A-alpha (Ab-307) |  | 1252 | 1250 | 110 | 99.5 | 104.75 | 1192.25 | 1315.95 | 1254.10 | 87.48 | 0.07 |
| PP2A-alpha (Phospho-Tyr307) |  | 78 | 71 | 110 | 99.5 | 104.75 | 74.28 | 74.75 | 74.51 | 0.33 | 0.00 |
| PPAR-BP (Ab-1457) |  | 159 | 151 | 110 | 99.5 | 104.75 | 151.41 | 158.97 | 155.19 | 5.34 | 0.03 |
| PPAR-BP (Phospho-Thr1457) |  | 82 | 68 | 110 | 99.5 | 104.75 | 78.09 | 71.59 | 74.84 | 4.60 | 0.06 |
| PPAR-gamma (Ab-112) |  | 1707 | 2579 | 110 | 99.5 | 104.75 | 1625.53 | 2715.08 | 2170.30 | 770.43 | 0.35 |
| PPAR-gamma (Phospho-Ser112) |  | 83 | 74 | 110 | 99.5 | 104.75 | 79.04 | 77.90 | 78.47 | 0.80 | 0.01 |
| Progesterone Receptor (Ab-190) |  | 178 | 146 | 110 | 99.5 | 104.75 | 169.50 | 153.70 | 161.60 | 11.17 | 0.07 |
| Progesterone Receptor (Phospho-Ser190) |  | 155 | 81 | 110 | 99.5 | 104.75 | 147.60 | 85.27 | 116.44 | 44.07 | 0.38 |
| PTEN (Ab-370) |  | 497 | 454 | 110 | 99.5 | 104.75 | 473.28 | 477.95 | 475.62 | 3.31 | 0.01 |
| PTEN (Ab-380) |  | 828 | 772 | 110 | 99.5 | 104.75 | 788.48 | 812.73 | 800.61 | 17.15 | 0.02 |
| PTEN (Ab-380/382/383) |  | 464 | 313 | 110 | 99.5 | 104.75 | 441.85 | 329.52 | 385.68 | 79.44 | 0.21 |
| PTEN (Phospho-Ser370) |  | 103 | 99 | 110 | 99.5 | 104.75 | 98.08 | 104.22 | 101.15 | 4.34 | 0.04 |
| PTEN (Phospho-Ser380) |  | 160 | 112 | 110 | 99.5 | 104.75 | 152.36 | 117.91 | 135.14 | 24.36 | 0.18 |
| PTEN (Phospho-Ser380/Thr382/Thr383) |  | 92 | 93 | 110 | 99.5 | 104.75 | 87.61 | 97.91 | 92.76 | 7.28 | 0.08 |
| PTPRA (Phospho-Tyr798) |  | 98 | 85 | 110 | 99.5 | 104.75 | 93.32 | 89.48 | 91.40 | 2.71 | 0.03 |
| Pyk2 (Ab-402) |  | 92 | 77 | 110 | 99.5 | 104.75 | 87.61 | 81.06 | 84.34 | 4.63 | 0.05 |
| Pyk2 (Ab-580) |  | 659 | 593 | 110 | 99.5 | 104.75 | 627.55 | 624.29 | 625.92 | 2.30 | 0.00 |
| Pyk2 (Ab-881) |  | 217 | 270 | 110 | 99.5 | 104.75 | 206.64 | 284.25 | 245.44 | 54.87 | 0.22 |
| Pyk2 (Phospho-Tyr402) |  | 139 | 92 | 110 | 99.5 | 104.75 | 132.37 | 96.85 | 114.61 | 25.11 | 0.22 |
| Pyk2 (Phospho-Tyr579) |  | 141 | 142 | 110 | 99.5 | 104.75 | 134.27 | 149.49 | 141.88 | 10.76 | 0.08 |
| Pyk2 (Phospho-Tyr580) |  | 77 | 129 | 110 | 99.5 | 104.75 | 73.33 | 135.81 | 104.57 | 44.18 | 0.42 |
| Pyk2 (Phospho-Tyr881) |  | 210 | 80 | 110 | 99.5 | 104.75 | 199.98 | 84.22 | 142.10 | 81.85 | 0.58 |
| Rac1/cdc42 (Ab-71) |  | 137 | 139 | 110 | 99.5 | 104.75 | 130.46 | 146.33 | 138.40 | 11.22 | 0.08 |
| Rac1/cdc42 (Phospho-Ser71) |  | 201 | 336 | 110 | 99.5 | 104.75 | 191.41 | 353.73 | 272.57 | 114.78 | 0.42 |
| RAD51 (Ab-309) |  | 118 | 141 | 110 | 99.5 | 104.75 | 112.37 | 148.44 | 130.40 | 25.51 | 0.20 |
| RAD51 (Phospho-Tyr315) |  | 76 | 70 | 110 | 99.5 | 104.75 | 72.37 | 73.69 | 73.03 | 0.93 | 0.01 |
| RAD52 (Phospho-Tyr104) |  | 137 | 143 | 110 | 99.5 | 104.75 | 130.46 | 150.55 | 140.50 | 14.20 | 0.10 |
| Raf1 (Ab-259) |  | 176 | 121 | 110 | 99.5 | 104.75 | 167.60 | 127.38 | 147.49 | 28.44 | 0.19 |
| Raf1 (Ab-289) |  | 322 | 541 | 110 | 99.5 | 104.75 | 306.63 | 569.55 | 438.09 | 185.91 | 0.42 |
| Raf1 (Ab-296) |  | 244 | 116 | 110 | 99.5 | 104.75 | 232.35 | 122.12 | 177.24 | 77.95 | 0.44 |
| Raf1 (Ab-338) |  | 142 | 142 | 110 | 99.5 | 104.75 | 135.22 | 149.49 | 142.36 | 10.09 | 0.07 |
| Raf1 (Ab-341) |  | 119 | 101 | 110 | 99.5 | 104.75 | 113.32 | 106.33 | 109.82 | 4.94 | 0.05 |
| Raf1 (Ab-43) |  | 143 | 168 | 110 | 99.5 | 104.75 | 136.18 | 176.86 | 156.52 | 28.77 | 0.18 |
| Raf1 (Ab-621) |  | 104 | 90 | 110 | 99.5 | 104.75 | 99.04 | 94.75 | 96.89 | 3.03 | 0.03 |
| Raf1 (Phospho-Ser259) |  | 191 | 114 | 110 | 99.5 | 104.75 | 181.88 | 120.02 | 150.95 | 43.75 | 0.29 |
| Raf1 (Phospho-Ser289) |  | 68 | 87 | 110 | 99.5 | 104.75 | 64.75 | 91.59 | 78.17 | 18.98 | 0.24 |
| Raf1 (Phospho-Ser296) |  | 65 | 69 | 110 | 99.5 | 104.75 | 61.90 | 72.64 | 67.27 | 7.60 | 0.11 |
| Raf1 (Phospho-Ser338) |  | 75 | 62 | 110 | 99.5 | 104.75 | 71.42 | 65.27 | 68.35 | 4.35 | 0.06 |
| Raf1 (Phospho-Ser43) |  | 63 | 167 | 110 | 99.5 | 104.75 | 59.99 | 175.81 | 117.90 | 81.90 | 0.69 |
| Raf1 (Phospho-Ser621) |  | 68 | 175 | 110 | 99.5 | 104.75 | 64.75 | 184.23 | 124.49 | 84.48 | 0.68 |
| Raf1 (Phospho-Tyr341) |  | 118 | 101 | 110 | 99.5 | 104.75 | 112.37 | 106.33 | 109.35 | 4.27 | 0.04 |
| RapGEF1 (Phospho-Tyr504) |  | 65 | 79 | 110 | 99.5 | 104.75 | 61.90 | 83.17 | 72.53 | 15.04 | 0.21 |
| Ras-GRF1 (Ab-916) |  | 314 | 650 | 110 | 99.5 | 104.75 | 299.01 | 684.30 | 491.66 | 272.44 | 0.55 |
| Ras-GRF1 (Phospho-Ser916) |  | 77 | 73 | 110 | 99.5 | 104.75 | 73.33 | 76.85 | 75.09 | 2.49 | 0.03 |
| Rb (Ab-608) |  | 90 | 80 | 110 | 99.5 | 104.75 | 85.70 | 84.22 | 84.96 | 1.05 | 0.01 |
| Rb (Ab-780) |  | 360 | 389 | 110 | 99.5 | 104.75 | 342.82 | 409.53 | 376.17 | 47.17 | 0.13 |
| Rb (Ab-795) |  | 221 | 311 | 110 | 99.5 | 104.75 | 210.45 | 327.41 | 268.93 | 82.70 | 0.31 |
| Rb (Ab-807) |  | 529 | 567 | 110 | 99.5 | 104.75 | 503.75 | 596.92 | 550.33 | 65.88 | 0.12 |
| Rb (Ab-811) |  | 119 | 134 | 110 | 99.5 | 104.75 | 113.32 | 141.07 | 127.20 | 19.62 | 0.15 |
| Rb (Phospho-Ser608) |  | 90 | 87 | 110 | 99.5 | 104.75 | 85.70 | 91.59 | 88.65 | 4.16 | 0.05 |
| Rb (Phospho-Ser780) |  | 76 | 73 | 110 | 99.5 | 104.75 | 72.37 | 76.85 | 74.61 | 3.17 | 0.04 |
| Rb (Phospho-Ser795) |  | 122 | 160 | 110 | 99.5 | 104.75 | 116.18 | 168.44 | 142.31 | 36.96 | 0.26 |
| Rb (Phospho-Ser807) |  | 80 | 77 | 110 | 99.5 | 104.75 | 76.18 | 81.06 | 78.62 | 3.45 | 0.04 |
| Rb (Phospho-Ser811) |  | 140 | 164 | 110 | 99.5 | 104.75 | 133.32 | 172.65 | 152.99 | 27.81 | 0.18 |
| Rb (Phospho-Thr821) |  | 63 | 68 | 110 | 99.5 | 104.75 | 59.99 | 71.59 | 65.79 | 8.20 | 0.12 |
| Rb-like-2 (RBL2) (Ab-952) |  | 68 | 68 | 110 | 99.5 | 104.75 | 64.75 | 71.59 | 68.17 | 4.83 | 0.07 |
| Rel (Ab-503) |  | 154 | 145 | 110 | 99.5 | 104.75 | 146.65 | 152.65 | 149.65 | 4.24 | 0.03 |
| Rel (Phospho-Ser503) |  | 553 | 73 | 110 | 99.5 | 104.75 | 526.61 | 76.85 | 301.73 | 318.02 | 1.05 |
| RelB (Ab-552) |  | 92 | 64 | 110 | 99.5 | 104.75 | 87.61 | 67.38 | 77.49 | 14.31 | 0.18 |
| RelB (Phospho-Ser552) |  | 217 | 91 | 110 | 99.5 | 104.75 | 206.64 | 95.80 | 151.22 | 78.38 | 0.52 |
| Ret (Ab-905) |  | 100 | 89 | 110 | 99.5 | 104.75 | 95.23 | 93.70 | 94.46 | 1.08 | 0.01 |
| Ret (Phospho-Tyr905) |  | 78 | 74 | 110 | 99.5 | 104.75 | 74.28 | 77.90 | 76.09 | 2.56 | 0.03 |
| RGS16 (Phospho-Tyr168) |  | 74 | 70 | 110 | 99.5 | 104.75 | 70.47 | 73.69 | 72.08 | 2.28 | 0.03 |
| Rho/Rac guanine nucleotide exchange factor 2 (Ab-885) |  | 67 | 63 | 110 | 99.5 | 104.75 | 63.80 | 66.32 | 65.06 | 1.78 | 0.03 |
| Rho/Rac guanine nucleotide exchange factor 2 (Phospho-Ser885) |  | 149 | 69 | 110 | 99.5 | 104.75 | 141.89 | 72.64 | 107.26 | 48.97 | 0.46 |
| RhoA (Ab-188) |  | 347 | 405 | 110 | 99.5 | 104.75 | 330.44 | 426.37 | 378.40 | 67.83 | 0.18 |
| RSK1/2/3/4 (Ab-221/227/218/232) |  | 97 | 98 | 110 | 99.5 | 104.75 | 92.37 | 103.17 | 97.77 | 7.64 | 0.08 |
| RSK1/2/3/4 (Phospho-Ser221/227/218/232) |  | 88 | 62 | 110 | 99.5 | 104.75 | 83.80 | 65.27 | 74.54 | 13.10 | 0.18 |
| RyR2 (Ab-2808) |  | 114 | 143 | 110 | 99.5 | 104.75 | 108.56 | 150.55 | 129.55 | 29.69 | 0.23 |
| RyR2 (Phospho-Ser2808) |  | 66 | 177 | 110 | 99.5 | 104.75 | 62.85 | 186.34 | 124.59 | 87.32 | 0.70 |
| S6 Ribosomal Protein (Ab-235) |  | 102 | 75 | 110 | 99.5 | 104.75 | 97.13 | 78.96 | 88.04 | 12.85 | 0.15 |
| S6 Ribosomal Protein (Phospho-Ser235) |  | 75 | 58 | 110 | 99.5 | 104.75 | 71.42 | 61.06 | 66.24 | 7.33 | 0.11 |
| SAPK/JNK (Ab-183) |  | 76 | 76 | 110 | 99.5 | 104.75 | 72.37 | 80.01 | 76.19 | 5.40 | 0.07 |
| SAPK/JNK (Ab-185) |  | 118 | 134 | 110 | 99.5 | 104.75 | 112.37 | 141.07 | 126.72 | 20.30 | 0.16 |
| SAPK/JNK (Phospho-Thr183) |  | 155 | 219 | 110 | 99.5 | 104.75 | 147.60 | 230.56 | 189.08 | 58.66 | 0.31 |
| SAPK/JNK (Phospho-Tyr185) |  | 86 | 77 | 110 | 99.5 | 104.75 | 81.90 | 81.06 | 81.48 | 0.59 | 0.01 |
| Shc (Ab-349) |  | 969 | 1002 | 110 | 99.5 | 104.75 | 922.75 | 1054.87 | 988.81 | 93.42 | 0.09 |
| Shc (Ab-427) |  | 368 | 362 | 110 | 99.5 | 104.75 | 350.44 | 381.10 | 365.77 | 21.68 | 0.06 |
| Shc (Phospho-Tyr349) |  | 84 | 99 | 110 | 99.5 | 104.75 | 79.99 | 104.22 | 92.11 | 17.14 | 0.19 |
| Shc (Phospho-Tyr427) |  | 91 | 85 | 110 | 99.5 | 104.75 | 86.66 | 89.48 | 88.07 | 2.00 | 0.02 |
| SHP-1 (Phospho-Tyr536) |  | 76 | 81 | 110 | 99.5 | 104.75 | 72.37 | 85.27 | 78.82 | 9.12 | 0.12 |
| SHP-2 (Ab-542) |  | 107 | 98 | 110 | 99.5 | 104.75 | 101.89 | 103.17 | 102.53 | 0.90 | 0.01 |
| SHP-2 (Ab-580) |  | 143 | 119 | 110 | 99.5 | 104.75 | 136.18 | 125.28 | 130.73 | 7.70 | 0.06 |
| SHP-2 (Phospho-Tyr542) |  | 341 | 78 | 110 | 99.5 | 104.75 | 324.73 | 82.12 | 203.42 | 171.55 | 0.84 |
| SHP-2 (Phospho-Tyr580) |  | 297 | 88 | 110 | 99.5 | 104.75 | 282.83 | 92.64 | 187.73 | 134.48 | 0.72 |
| SLP-76 (Ab-128) |  | 172 | 101 | 110 | 99.5 | 104.75 | 163.79 | 106.33 | 135.06 | 40.63 | 0.30 |
| SLP-76 (Phospho-Tyr128) |  | 98 | 219 | 110 | 99.5 | 104.75 | 93.32 | 230.56 | 161.94 | 97.04 | 0.60 |
| Smad1 (Ab-187) |  | 79 | 85 | 110 | 99.5 | 104.75 | 75.23 | 89.48 | 82.36 | 10.08 | 0.12 |
| Smad1 (Ab-465) |  | 62 | 66 | 110 | 99.5 | 104.75 | 59.04 | 69.48 | 64.26 | 7.38 | 0.11 |
| Smad1 (Phospho-Ser187) |  | 69 | 67 | 110 | 99.5 | 104.75 | 65.71 | 70.54 | 68.12 | 3.41 | 0.05 |
| Smad1 (Phospho-Ser465) |  | 60 | 60 | 110 | 99.5 | 104.75 | 57.14 | 63.17 | 60.15 | 4.26 | 0.07 |
| Smad2 (Ab-220) |  | 65 | 94 | 110 | 99.5 | 104.75 | 61.90 | 98.96 | 80.43 | 26.21 | 0.33 |
| Smad2 (Ab-245) |  | 58 | 58 | 110 | 99.5 | 104.75 | 55.23 | 61.06 | 58.15 | 4.12 | 0.07 |
| Smad2 (Ab-250) |  | 81 | 93 | 110 | 99.5 | 104.75 | 77.13 | 97.91 | 87.52 | 14.69 | 0.17 |
| Smad2 (Ab-255) |  | 287 | 491 | 110 | 99.5 | 104.75 | 273.30 | 516.91 | 395.10 | 172.25 | 0.44 |
| Smad2 (Ab-467) |  | 84 | 88 | 110 | 99.5 | 104.75 | 79.99 | 92.64 | 86.32 | 8.95 | 0.10 |
| Smad2 (Phospho-Ser250) |  | 75 | 75 | 110 | 99.5 | 104.75 | 71.42 | 78.96 | 75.19 | 5.33 | 0.07 |
| Smad2 (Phospho-Ser467) |  | 68 | 61 | 110 | 99.5 | 104.75 | 64.75 | 64.22 | 64.49 | 0.38 | 0.01 |
| Smad2 (Phospho-Thr220) |  | 67 | 68 | 110 | 99.5 | 104.75 | 63.80 | 71.59 | 67.70 | 5.51 | 0.08 |
| Smad2/3 (Ab-8) |  | 181 | 213 | 110 | 99.5 | 104.75 | 172.36 | 224.24 | 198.30 | 36.68 | 0.18 |
| Smad2/3 (Phospho-Thr8) |  | 361 | 504 | 110 | 99.5 | 104.75 | 343.77 | 530.59 | 437.18 | 132.10 | 0.30 |
| Smad3 (Ab-179) |  | 133 | 149 | 110 | 99.5 | 104.75 | 126.65 | 156.86 | 141.76 | 21.36 | 0.15 |
| Smad3 (Ab-204) |  | 89 | 102 | 110 | 99.5 | 104.75 | 84.75 | 107.38 | 96.07 | 16.00 | 0.17 |
| Smad3 (Ab-213) |  | 62 | 60 | 110 | 99.5 | 104.75 | 59.04 | 63.17 | 61.10 | 2.92 | 0.05 |
| Smad3 (Ab-425) |  | 78 | 79 | 110 | 99.5 | 104.75 | 74.28 | 83.17 | 78.72 | 6.29 | 0.08 |
| Smad3 (Phospho-Ser204) |  | 64 | 57 | 110 | 99.5 | 104.75 | 60.95 | 60.01 | 60.48 | 0.66 | 0.01 |
| Smad3 (Phospho-Ser208) |  | 86 | 69 | 110 | 99.5 | 104.75 | 81.90 | 72.64 | 77.27 | 6.54 | 0.08 |
| Smad3 (Phospho-Ser213) |  | 434 | 175 | 110 | 99.5 | 104.75 | 413.29 | 184.23 | 298.76 | 161.96 | 0.54 |
| Smad3 (Phospho-Ser425) |  | 162 | 64 | 110 | 99.5 | 104.75 | 154.27 | 67.38 | 110.82 | 61.44 | 0.55 |
| Smad3 (Phospho-Thr179) |  | 92 | 96 | 110 | 99.5 | 104.75 | 87.61 | 101.07 | 94.34 | 9.51 | 0.10 |
| SMC1 (Ab-957) |  | 70 | 69 | 110 | 99.5 | 104.75 | 66.66 | 72.64 | 69.65 | 4.23 | 0.06 |
| SMC1 (Phospho-Ser957) |  | 92 | 81 | 110 | 99.5 | 104.75 | 87.61 | 85.27 | 86.44 | 1.65 | 0.02 |
| SP1 (Ab-739) |  | 97 | 82 | 110 | 99.5 | 104.75 | 92.37 | 86.33 | 89.35 | 4.27 | 0.05 |
| SP1 (Phospho-Thr739) |  | 108 | 430 | 110 | 99.5 | 104.75 | 102.85 | 452.69 | 277.77 | 247.38 | 0.89 |
| Src (Ab-418) |  | 2830 | 3257 | 110 | 99.5 | 104.75 | 2694.93 | 3428.85 | 3061.89 | 518.96 | 0.17 |
| Src (Ab-529) |  | 95 | 86 | 110 | 99.5 | 104.75 | 90.47 | 90.54 | 90.50 | 0.05 | 0.00 |
| Src (Ab-75) |  | 92 | 91 | 110 | 99.5 | 104.75 | 87.61 | 95.80 | 91.71 | 5.79 | 0.06 |
| Src (Phospho-Ser75) |  | 60 | 63 | 110 | 99.5 | 104.75 | 57.14 | 66.32 | 61.73 | 6.50 | 0.11 |
| Src (Phospho-Tyr216) |  | 191 | 199 | 110 | 99.5 | 104.75 | 181.88 | 209.50 | 195.69 | 19.53 | 0.10 |
| Src (Phospho-Tyr418) |  | 112 | 99 | 110 | 99.5 | 104.75 | 106.65 | 104.22 | 105.44 | 1.72 | 0.02 |
| Src (Phospho-Tyr529) |  | 114 | 97 | 110 | 99.5 | 104.75 | 108.56 | 102.12 | 105.34 | 4.55 | 0.04 |
| SREBP-1 (Ab-439) |  | 103 | 104 | 110 | 99.5 | 104.75 | 98.08 | 109.49 | 103.79 | 8.06 | 0.08 |
| SREBP-1 (Phospho-Ser439) |  | 63 | 63 | 110 | 99.5 | 104.75 | 59.99 | 66.32 | 63.16 | 4.48 | 0.07 |
| SRF (Ab-77) |  | 83 | 78 | 110 | 99.5 | 104.75 | 79.04 | 82.12 | 80.58 | 2.18 | 0.03 |
| SRF (Ab-99) |  | 694 | 864 | 110 | 99.5 | 104.75 | 660.88 | 909.59 | 785.23 | 175.86 | 0.22 |
| SRF (Phospho-Ser77) |  | 104 | 81 | 110 | 99.5 | 104.75 | 99.04 | 85.27 | 92.16 | 9.73 | 0.11 |
| SRF (Phospho-Ser99) |  | 146 | 119 | 110 | 99.5 | 104.75 | 139.03 | 125.28 | 132.16 | 9.72 | 0.07 |
| STAM2 (Ab-192) |  | 79 | 80 | 110 | 99.5 | 104.75 | 75.23 | 84.22 | 79.73 | 6.36 | 0.08 |
| STAM2 (Phospho-Tyr192) |  | 316 | 253 | 110 | 99.5 | 104.75 | 300.92 | 266.35 | 283.63 | 24.44 | 0.09 |
| STAT1 (Ab-701) |  | 79 | 71 | 110 | 99.5 | 104.75 | 75.23 | 74.75 | 74.99 | 0.34 | 0.00 |
| STAT1 (Ab-727) |  | 197 | 179 | 110 | 99.5 | 104.75 | 187.60 | 188.44 | 188.02 | 0.60 | 0.00 |
| STAT1 (Phospho-Ser727) |  | 80 | 77 | 110 | 99.5 | 104.75 | 76.18 | 81.06 | 78.62 | 3.45 | 0.04 |
| STAT1 (Phospho-Tyr701) |  | 253 | 111 | 110 | 99.5 | 104.75 | 240.93 | 116.86 | 178.89 | 87.73 | 0.49 |
| STAT2 (Ab-690) |  | 88 | 85 | 110 | 99.5 | 104.75 | 83.80 | 89.48 | 86.64 | 4.02 | 0.05 |
| STAT2 (Phospho-Tyr690) |  | 101 | 90 | 110 | 99.5 | 104.75 | 96.18 | 94.75 | 95.46 | 1.01 | 0.01 |
| STAT3 (Ab-705) |  | 168 | 176 | 110 | 99.5 | 104.75 | 159.98 | 185.29 | 172.63 | 17.89 | 0.10 |
| STAT3 (Ab-727) |  | 84 | 84 | 110 | 99.5 | 104.75 | 79.99 | 88.43 | 84.21 | 5.97 | 0.07 |
| STAT3 (Phospho-Ser727) |  | 76 | 74 | 110 | 99.5 | 104.75 | 72.37 | 77.90 | 75.14 | 3.91 | 0.05 |
| STAT3 (Phospho-Tyr705) |  | 422 | 383 | 110 | 99.5 | 104.75 | 401.86 | 403.21 | 402.53 | 0.95 | 0.00 |
| STAT4 (Ab-693) |  | 107 | 110 | 110 | 99.5 | 104.75 | 101.89 | 115.80 | 108.85 | 9.84 | 0.09 |
| STAT4 (Phospho-Tyr693) |  | 112 | 121 | 110 | 99.5 | 104.75 | 106.65 | 127.38 | 117.02 | 14.66 | 0.13 |
| STAT5A (Ab-694) |  | 76 | 74 | 110 | 99.5 | 104.75 | 72.37 | 77.90 | 75.14 | 3.91 | 0.05 |
| STAT5A (Ab-780) |  | 133 | 88 | 110 | 99.5 | 104.75 | 126.65 | 92.64 | 109.65 | 24.05 | 0.22 |
| STAT5A (Phospho-Ser725) |  | 93 | 63 | 110 | 99.5 | 104.75 | 88.56 | 66.32 | 77.44 | 15.72 | 0.20 |
| STAT5A (Phospho-Ser780) |  | 95 | 94 | 110 | 99.5 | 104.75 | 90.47 | 98.96 | 94.71 | 6.01 | 0.06 |
| STAT5A (Phospho-Tyr694) |  | 99 | 89 | 110 | 99.5 | 104.75 | 94.28 | 93.70 | 93.99 | 0.41 | 0.00 |
| STAT5B (Ab-731) |  | 93 | 83 | 110 | 99.5 | 104.75 | 88.56 | 87.38 | 87.97 | 0.84 | 0.01 |
| STAT5B (Phospho-Ser731) |  | 67 | 123 | 110 | 99.5 | 104.75 | 63.80 | 129.49 | 96.65 | 46.45 | 0.48 |
| STAT6 (Ab-641) |  | 118 | 110 | 110 | 99.5 | 104.75 | 112.37 | 115.80 | 114.09 | 2.43 | 0.02 |
| STAT6 (Ab-645) |  | 1420 | 1237 | 110 | 99.5 | 104.75 | 1352.23 | 1302.27 | 1327.25 | 35.33 | 0.03 |
| STAT6 (Phospho-Thr645) |  | 186 | 167 | 110 | 99.5 | 104.75 | 177.12 | 175.81 | 176.47 | 0.93 | 0.01 |
| STAT6 (Phospho-Tyr641) |  | 174 | 207 | 110 | 99.5 | 104.75 | 165.70 | 217.92 | 191.81 | 36.93 | 0.19 |
| Stathmin 1 (Ab-15) |  | 95 | 83 | 110 | 99.5 | 104.75 | 90.47 | 87.38 | 88.92 | 2.18 | 0.02 |
| Stathmin 1 (Ab-24) |  | 75 | 74 | 110 | 99.5 | 104.75 | 71.42 | 77.90 | 74.66 | 4.58 | 0.06 |
| Stathmin 1 (Ab-37) |  | 80 | 77 | 110 | 99.5 | 104.75 | 76.18 | 81.06 | 78.62 | 3.45 | 0.04 |
| Stathmin 1 (Phospho-Ser15) |  | 120 | 98 | 110 | 99.5 | 104.75 | 114.27 | 103.17 | 108.72 | 7.85 | 0.07 |
| Stathmin 1 (Phospho-Ser24) |  | 85 | 85 | 110 | 99.5 | 104.75 | 80.94 | 89.48 | 85.21 | 6.04 | 0.07 |
| Stathmin 1 (Phospho-Ser37) |  | 74 | 74 | 110 | 99.5 | 104.75 | 70.47 | 77.90 | 74.19 | 5.26 | 0.07 |
| Survivin (Ab-117) |  | 70 | 67 | 110 | 99.5 | 104.75 | 66.66 | 70.54 | 68.60 | 2.74 | 0.04 |
| Survivin (Phospho-Thr117) |  | 64 | 66 | 110 | 99.5 | 104.75 | 60.95 | 69.48 | 65.21 | 6.04 | 0.09 |
| SYK (Ab-348) |  | 132 | 177 | 110 | 99.5 | 104.75 | 125.70 | 186.34 | 156.02 | 42.88 | 0.27 |
| SYK (Ab-525) |  | 711 | 1476 | 110 | 99.5 | 104.75 | 677.07 | 1553.88 | 1115.47 | 620.00 | 0.56 |
| SYK (Phospho-Tyr323) |  | 94 | 96 | 110 | 99.5 | 104.75 | 89.51 | 101.07 | 95.29 | 8.17 | 0.09 |
| SYK (Phospho-Tyr348) |  | 67 | 67 | 110 | 99.5 | 104.75 | 63.80 | 70.54 | 67.17 | 4.76 | 0.07 |
| SYK (Phospho-Tyr525) |  | 60 | 60 | 110 | 99.5 | 104.75 | 57.14 | 63.17 | 60.15 | 4.26 | 0.07 |
| Synapsin (Ab-62) |  | 73 | 69 | 110 | 99.5 | 104.75 | 69.52 | 72.64 | 71.08 | 2.21 | 0.03 |
| Synapsin (Ab-9) |  | 77 | 78 | 110 | 99.5 | 104.75 | 73.33 | 82.12 | 77.72 | 6.22 | 0.08 |
| Synapsin (Phospho-Ser62) |  | 69 | 62 | 110 | 99.5 | 104.75 | 65.71 | 65.27 | 65.49 | 0.31 | 0.00 |
| Synapsin (Phospho-Ser9) |  | 112 | 97 | 110 | 99.5 | 104.75 | 106.65 | 102.12 | 104.39 | 3.21 | 0.03 |
| Synaptotagmin (Ab-202) |  | 209 | 179 | 110 | 99.5 | 104.75 | 199.03 | 188.44 | 193.73 | 7.48 | 0.04 |
| Synaptotagmin (Ab-309) |  | 88 | 91 | 110 | 99.5 | 104.75 | 83.80 | 95.80 | 89.80 | 8.49 | 0.09 |
| Synaptotagmin (Phospho-Ser309) |  | 65 | 64 | 110 | 99.5 | 104.75 | 61.90 | 67.38 | 64.64 | 3.87 | 0.06 |
| Synaptotagmin (Phospho-Thr202) |  | 66 | 65 | 110 | 99.5 | 104.75 | 62.85 | 68.43 | 65.64 | 3.95 | 0.06 |
| Synuclein alpha (Ab-125) |  | 239 | 114 | 110 | 99.5 | 104.75 | 227.59 | 120.02 | 173.80 | 76.07 | 0.44 |
| Synuclein alpha (Ab-133) |  | 90 | 77 | 110 | 99.5 | 104.75 | 85.70 | 81.06 | 83.38 | 3.28 | 0.04 |
| Synuclein alpha (Phospho-Tyr125) |  | 125 | 104 | 110 | 99.5 | 104.75 | 119.03 | 109.49 | 114.26 | 6.75 | 0.06 |
| Synuclein alpha (Phospho-Tyr133) |  | 75 | 78 | 110 | 99.5 | 104.75 | 71.42 | 82.12 | 76.77 | 7.56 | 0.10 |
| Synuclein alpha (Phospho-Tyr136) |  | 100 | 95 | 110 | 99.5 | 104.75 | 95.23 | 100.01 | 97.62 | 3.38 | 0.03 |
| Tau (Ab-181) |  | 111 | 99 | 110 | 99.5 | 104.75 | 105.70 | 104.22 | 104.96 | 1.05 | 0.01 |
| Tau (Ab-205) |  | 319 | 226 | 110 | 99.5 | 104.75 | 303.78 | 237.92 | 270.85 | 46.56 | 0.17 |
| Tau (Ab-212) |  | 1122 | 970 | 110 | 99.5 | 104.75 | 1068.45 | 1021.18 | 1044.82 | 33.42 | 0.03 |
| Tau (Ab-214) |  | 205 | 182 | 110 | 99.5 | 104.75 | 195.22 | 191.60 | 193.41 | 2.55 | 0.01 |
| Tau (Ab-231) |  | 99 | 96 | 110 | 99.5 | 104.75 | 94.28 | 101.07 | 97.67 | 4.80 | 0.05 |
| Tau (Ab-235) |  | 5195 | 4524 | 110 | 99.5 | 104.75 | 4947.06 | 4762.70 | 4854.88 | 130.36 | 0.03 |
| Tau (Ab-262) |  | 100 | 88 | 110 | 99.5 | 104.75 | 95.23 | 92.64 | 93.94 | 1.83 | 0.02 |
| Tau (Ab-356) |  | 1921 | 1779 | 110 | 99.5 | 104.75 | 1829.32 | 1872.87 | 1851.09 | 30.80 | 0.02 |
| Tau (Ab-396) |  | 326 | 399 | 110 | 99.5 | 104.75 | 310.44 | 420.05 | 365.25 | 77.51 | 0.21 |
| Tau (Ab-404) |  | 315 | 280 | 110 | 99.5 | 104.75 | 299.97 | 294.77 | 297.37 | 3.67 | 0.01 |
| Tau (Ab-422) |  | 86 | 84 | 110 | 99.5 | 104.75 | 81.90 | 88.43 | 85.16 | 4.62 | 0.05 |
| Tau (Phospho-Ser214) |  | 113 | 108 | 110 | 99.5 | 104.75 | 107.61 | 113.70 | 110.65 | 4.31 | 0.04 |
| Tau (Phospho-Ser235) |  | 303 | 275 | 110 | 99.5 | 104.75 | 288.54 | 289.51 | 289.02 | 0.69 | 0.00 |
| Tau (Phospho-Ser262) |  | 79 | 78 | 110 | 99.5 | 104.75 | 75.23 | 82.12 | 78.67 | 4.87 | 0.06 |
| Tau (Phospho-Ser356) |  | 112 | 101 | 110 | 99.5 | 104.75 | 106.65 | 106.33 | 106.49 | 0.23 | 0.00 |
| Tau (Phospho-Ser396) |  | 443 | 148 | 110 | 99.5 | 104.75 | 421.86 | 155.81 | 288.83 | 188.12 | 0.65 |
| Tau (Phospho-Ser404) |  | 135 | 115 | 110 | 99.5 | 104.75 | 128.56 | 121.07 | 124.81 | 5.30 | 0.04 |
| Tau (Phospho-Ser422) |  | 92 | 85 | 110 | 99.5 | 104.75 | 87.61 | 89.48 | 88.55 | 1.33 | 0.01 |
| Tau (Phospho-Thr181) |  | 143 | 140 | 110 | 99.5 | 104.75 | 136.18 | 147.39 | 141.78 | 7.93 | 0.06 |
| Tau (Phospho-Thr205) |  | 150 | 119 | 110 | 99.5 | 104.75 | 142.84 | 125.28 | 134.06 | 12.42 | 0.09 |
| Tau (Phospho-Thr212) |  | 129 | 114 | 110 | 99.5 | 104.75 | 122.84 | 120.02 | 121.43 | 2.00 | 0.02 |
| Tau (Phospho-Thr231) |  | 127 | 146 | 110 | 99.5 | 104.75 | 120.94 | 153.70 | 137.32 | 23.17 | 0.17 |
| TFII-I (Phospho-Tyr248) |  | 73 | 70 | 110 | 99.5 | 104.75 | 69.52 | 73.69 | 71.60 | 2.95 | 0.04 |
| TGFBR1 (Ab-165) |  | 80 | 81 | 110 | 99.5 | 104.75 | 76.18 | 85.27 | 80.73 | 6.43 | 0.08 |
| TGFBR2 (Ab-250) |  | 567 | 1207 | 110 | 99.5 | 104.75 | 539.94 | 1270.69 | 905.31 | 516.72 | 0.57 |
| TIE2 (Phospho-Tyr1108) |  | 119 | 105 | 110 | 99.5 | 104.75 | 113.32 | 110.54 | 111.93 | 1.97 | 0.02 |
| TIF-IA (Ab-649) |  | 88 | 97 | 110 | 99.5 | 104.75 | 83.80 | 102.12 | 92.96 | 12.95 | 0.14 |
| TIF-IA (Phospho-Ser649) |  | 124 | 123 | 110 | 99.5 | 104.75 | 118.08 | 129.49 | 123.79 | 8.07 | 0.07 |
| TLK1 (Ab-764) |  | 398 | 437 | 110 | 99.5 | 104.75 | 379.00 | 460.06 | 419.53 | 57.31 | 0.14 |
| TOP2A/DNA topoisomerase II (Ab-1106) |  | 633 | 422 | 110 | 99.5 | 104.75 | 602.79 | 444.27 | 523.53 | 112.09 | 0.21 |
| TOP2A/DNA topoisomerase II (Phospho-Ser1106) |  | 72 | 132 | 110 | 99.5 | 104.75 | 68.56 | 138.96 | 103.76 | 49.78 | 0.48 |
| Trk A (Ab-496) |  | 83 | 77 | 110 | 99.5 | 104.75 | 79.04 | 81.06 | 80.05 | 1.43 | 0.02 |
| Trk A (Phospho-Tyr680/681) |  | 79 | 69 | 110 | 99.5 | 104.75 | 75.23 | 72.64 | 73.94 | 1.83 | 0.02 |
| Trk A (Phospho-Tyr701) |  | 94 | 89 | 110 | 99.5 | 104.75 | 89.51 | 93.70 | 91.60 | 2.96 | 0.03 |
| Trk A (Phospho-Tyr791) |  | 75 | 71 | 110 | 99.5 | 104.75 | 71.42 | 74.75 | 73.08 | 2.35 | 0.03 |
| Trk B (Ab-515) |  | 242 | 302 | 110 | 99.5 | 104.75 | 230.45 | 317.93 | 274.19 | 61.86 | 0.23 |
| Trk B (Phospho-Tyr515) |  | 158 | 169 | 110 | 99.5 | 104.75 | 150.46 | 177.92 | 164.19 | 19.42 | 0.12 |
| Trk B (Phospho-Tyr705) |  | 105 | 73 | 110 | 99.5 | 104.75 | 99.99 | 76.85 | 88.42 | 16.36 | 0.19 |
| Tuberin/TSC2 (Ab-1462) |  | 68 | 70 | 110 | 99.5 | 104.75 | 64.75 | 73.69 | 69.22 | 6.32 | 0.09 |
| Tuberin/TSC2 (Ab-939) |  | 835 | 856 | 110 | 99.5 | 104.75 | 795.15 | 901.17 | 848.16 | 74.97 | 0.09 |
| Tuberin/TSC2 (Ab-981) |  | 121 | 113 | 110 | 99.5 | 104.75 | 115.23 | 118.96 | 117.09 | 2.64 | 0.02 |
| Tuberin/TSC2 (Phospho-Ser939) |  | 109 | 113 | 110 | 99.5 | 104.75 | 103.80 | 118.96 | 111.38 | 10.72 | 0.10 |
| Tuberin/TSC2 (Phospho-Thr1462) |  | 144 | 150 | 110 | 99.5 | 104.75 | 137.13 | 157.91 | 147.52 | 14.70 | 0.10 |
| TYK2 (Ab-1054) |  | 86 | 86 | 110 | 99.5 | 104.75 | 81.90 | 90.54 | 86.22 | 6.11 | 0.07 |
| TYK2 (Phospho-Tyr1054) |  | 97 | 95 | 110 | 99.5 | 104.75 | 92.37 | 100.01 | 96.19 | 5.40 | 0.06 |
| Tyrosine Hydroxylase (Ab-19) |  | 235 | 147 | 110 | 99.5 | 104.75 | 223.78 | 154.76 | 189.27 | 48.81 | 0.26 |
| Tyrosine Hydroxylase (Ab-31) |  | 199 | 94 | 110 | 99.5 | 104.75 | 189.50 | 98.96 | 144.23 | 64.02 | 0.44 |
| Tyrosine Hydroxylase (Ab-40) |  | 290 | 200 | 110 | 99.5 | 104.75 | 276.16 | 210.55 | 243.36 | 46.39 | 0.19 |
| Tyrosine Hydroxylase (Ab-8) |  | 269 | 212 | 110 | 99.5 | 104.75 | 256.16 | 223.19 | 239.67 | 23.32 | 0.10 |
| Tyrosine Hydroxylase (Phospho-Ser19) |  | 91 | 81 | 110 | 99.5 | 104.75 | 86.66 | 85.27 | 85.97 | 0.98 | 0.01 |
| Tyrosine Hydroxylase (Phospho-Ser31) |  | 78 | 75 | 110 | 99.5 | 104.75 | 74.28 | 78.96 | 76.62 | 3.31 | 0.04 |
| Tyrosine Hydroxylase (Phospho-Ser40) |  | 79 | 73 | 110 | 99.5 | 104.75 | 75.23 | 76.85 | 76.04 | 1.15 | 0.02 |
| Tyrosine Hydroxylase (Phospho-Ser8) |  | 101 | 127 | 110 | 99.5 | 104.75 | 96.18 | 133.70 | 114.94 | 26.53 | 0.23 |
| VASP (Ab-157) |  | 652 | 773 | 110 | 99.5 | 104.75 | 620.88 | 813.79 | 717.33 | 136.40 | 0.19 |
| VASP (Ab-238) |  | 92 | 95 | 110 | 99.5 | 104.75 | 87.61 | 100.01 | 93.81 | 8.77 | 0.09 |
| VASP (Phospho-Ser157) |  | 200 | 141 | 110 | 99.5 | 104.75 | 190.45 | 148.44 | 169.45 | 29.71 | 0.18 |
| VASP (Phospho-Ser238) |  | 97 | 94 | 110 | 99.5 | 104.75 | 92.37 | 98.96 | 95.67 | 4.66 | 0.05 |
| VAV1 (Ab-160) |  | 200 | 187 | 110 | 99.5 | 104.75 | 190.45 | 196.87 | 193.66 | 4.53 | 0.02 |
| VAV1 (Ab-174) |  | 169 | 157 | 110 | 99.5 | 104.75 | 160.93 | 165.28 | 163.11 | 3.08 | 0.02 |
| VAV1 (Phospho-Tyr174) |  | 80 | 69 | 110 | 99.5 | 104.75 | 76.18 | 72.64 | 74.41 | 2.50 | 0.03 |
| VAV2 (Ab-142) |  | 79 | 74 | 110 | 99.5 | 104.75 | 75.23 | 77.90 | 76.57 | 1.89 | 0.02 |
| VAV2 (Phospho-Tyr142) |  | 394 | 234 | 110 | 99.5 | 104.75 | 375.20 | 246.35 | 310.77 | 91.11 | 0.29 |
| VE-Cadherin (Phospho-Tyr731) |  | 1708 | 1397 | 110 | 99.5 | 104.75 | 1626.48 | 1470.71 | 1548.60 | 110.15 | 0.07 |
| VEGFR1 (Ab-1333) |  | 101 | 109 | 110 | 99.5 | 104.75 | 96.18 | 114.75 | 105.47 | 13.13 | 0.12 |
| VEGFR1 (Phospho-Tyr1333) |  | 94 | 86 | 110 | 99.5 | 104.75 | 89.51 | 90.54 | 90.03 | 0.72 | 0.01 |
| VEGFR2 (Ab-1054) |  | 95 | 91 | 110 | 99.5 | 104.75 | 90.47 | 95.80 | 93.13 | 3.77 | 0.04 |
| VEGFR2 (Ab-1059) |  | 66 | 64 | 110 | 99.5 | 104.75 | 62.85 | 67.38 | 65.11 | 3.20 | 0.05 |
| VEGFR2 (Ab-1175) |  | 149 | 122 | 110 | 99.5 | 104.75 | 141.89 | 128.44 | 135.16 | 9.51 | 0.07 |
| VEGFR2 (Ab-1214) |  | 2727 | 2227 | 110 | 99.5 | 104.75 | 2596.85 | 2344.51 | 2470.68 | 178.43 | 0.07 |
| VEGFR2 (Ab-951) |  | 4816 | 3294 | 110 | 99.5 | 104.75 | 4586.15 | 3467.80 | 4026.97 | 790.79 | 0.20 |
| VEGFR2 (Phospho-Tyr1054) |  | 106 | 93 | 110 | 99.5 | 104.75 | 100.94 | 97.91 | 99.42 | 2.15 | 0.02 |
| VEGFR2 (Phospho-Tyr1059) |  | 82 | 69 | 110 | 99.5 | 104.75 | 78.09 | 72.64 | 75.36 | 3.85 | 0.05 |
| VEGFR2 (Phospho-Tyr1175) |  | 99 | 93 | 110 | 99.5 | 104.75 | 94.28 | 97.91 | 96.09 | 2.57 | 0.03 |
| VEGFR2 (Phospho-Tyr1214) |  | 136 | 140 | 110 | 99.5 | 104.75 | 129.51 | 147.39 | 138.45 | 12.64 | 0.09 |
| VEGFR2 (Phospho-Tyr951) |  | 137 | 144 | 110 | 99.5 | 104.75 | 130.46 | 151.60 | 141.03 | 14.95 | 0.11 |
| Vinculin (Ab-821) |  | 65 | 73 | 110 | 99.5 | 104.75 | 61.90 | 76.85 | 69.37 | 10.57 | 0.15 |
| Vinculin (Phospho-Tyr821) |  | 380 | 119 | 110 | 99.5 | 104.75 | 361.86 | 125.28 | 243.57 | 167.29 | 0.69 |
| WASP (Ab-290) |  | 97 | 172 | 110 | 99.5 | 104.75 | 92.37 | 181.08 | 136.72 | 62.72 | 0.46 |
| WASP (Phospho-Tyr290) |  | 72 | 70 | 110 | 99.5 | 104.75 | 68.56 | 73.69 | 71.13 | 3.63 | 0.05 |
| WAVE1 (Ab-125) |  | 76 | 76 | 110 | 99.5 | 104.75 | 72.37 | 80.01 | 76.19 | 5.40 | 0.07 |
| WAVE1 (Phospho-Tyr125) |  | 88 | 75 | 110 | 99.5 | 104.75 | 83.80 | 78.96 | 81.38 | 3.42 | 0.04 |
| WEE1 (Ab-53) |  | 88 | 87 | 110 | 99.5 | 104.75 | 83.80 | 91.59 | 87.70 | 5.51 | 0.06 |
| WEE1 (Phospho-Ser53) |  | 66 | 67 | 110 | 99.5 | 104.75 | 62.85 | 70.54 | 66.69 | 5.43 | 0.08 |
| WEE1 (Phospho-Ser642) |  | 99 | 147 | 110 | 99.5 | 104.75 | 94.28 | 154.76 | 124.52 | 42.77 | 0.34 |
| WWOX (Phospho-Tyr33) |  | 111 | 97 | 110 | 99.5 | 104.75 | 105.70 | 102.12 | 103.91 | 2.53 | 0.02 |
| XIAP (Ab-87) |  | 190 | 183 | 110 | 99.5 | 104.75 | 180.93 | 192.66 | 186.79 | 8.29 | 0.04 |
| XIAP (Phospho-Ser87) |  | 74 | 69 | 110 | 99.5 | 104.75 | 70.47 | 72.64 | 71.55 | 1.54 | 0.02 |
| Zap-70 (Ab-292) |  | 188 | 185 | 110 | 99.5 | 104.75 | 179.03 | 194.76 | 186.89 | 11.13 | 0.06 |
| Zap-70 (Ab-319) |  | 412 | 401 | 110 | 99.5 | 104.75 | 392.34 | 422.16 | 407.25 | 21.09 | 0.05 |
| Zap-70 (Ab-493) |  | 144 | 123 | 110 | 99.5 | 104.75 | 137.13 | 129.49 | 133.31 | 5.40 | 0.04 |
| Zap-70 (Phospho-Tyr292) |  | 72 | 66 | 110 | 99.5 | 104.75 | 68.56 | 69.48 | 69.02 | 0.65 | 0.01 |
| Zap-70 (Phospho-Tyr315) |  | 121 | 111 | 110 | 99.5 | 104.75 | 115.23 | 116.86 | 116.04 | 1.15 | 0.01 |
| Zap-70 (Phospho-Tyr319) |  | 143 | 137 | 110 | 99.5 | 104.75 | 136.18 | 144.23 | 140.20 | 5.69 | 0.04 |
| Zap-70 (Phospho-Tyr493) |  | 129 | 235 | 110 | 99.5 | 104.75 | 122.84 | 247.40 | 185.12 | 88.07 | 0.48 |

**Supplementary Table 2. The standard detection value of Sora group**

| Name | FI_i_ | FI_j_ | X | Y | Z | I_i_ | I_j_ | Average | SD | CV |
| --- | --- | --- | --- | --- | --- | --- | --- | --- | --- | --- |
| Positive Marker | 6519 | 6070 | 140.5 | 128 | 134.25 | 6229.01 | 6366.39 | 6297.70 | 97.14 | 0.02 |
| Positive Marker | 6018 | 6104 | 140.5 | 128 | 134.25 | 5750.30 | 6402.05 | 6076.17 | 460.86 | 0.08 |
| Negative control | 169 | 176 | 140.5 | 128 | 134.25 | 161.48 | 184.59 | 173.04 | 16.34 | 0.09 |
| Negative control | 167 | 166 | 140.5 | 128 | 134.25 | 159.57 | 174.11 | 166.84 | 10.28 | 0.06 |
| Negative control | 163 | 169 | 140.5 | 128 | 134.25 | 155.75 | 177.25 | 166.50 | 15.20 | 0.09 |
| Negative control | 171 | 162 | 140.5 | 128 | 134.25 | 163.39 | 169.91 | 166.65 | 4.61 | 0.03 |
| Empty | 69 | 66 | 140.5 | 128 | 134.25 | 65.93 | 69.22 | 67.58 | 2.33 | 0.03 |
| Empty | 66 | 66 | 140.5 | 128 | 134.25 | 63.06 | 69.22 | 66.14 | 4.35 | 0.07 |
| Empty | 55 | 52 | 140.5 | 128 | 134.25 | 52.55 | 54.54 | 53.55 | 1.40 | 0.03 |
| Empty | 55 | 51 | 140.5 | 128 | 134.25 | 52.55 | 53.49 | 53.02 | 0.66 | 0.01 |
| 14-3-3 beta/zeta (Ab-184/186) | 119 | 86 | 140.5 | 128 | 134.25 | 113.71 | 90.20 | 101.95 | 16.62 | 0.16 |
| 14-3-3 beta/zeta (Phospho-Ser184/186) | 98 | 99 | 140.5 | 128 | 134.25 | 93.64 | 103.83 | 98.74 | 7.21 | 0.07 |
| 14-3-3 theta/tau (Ab-232) | 92 | 96 | 140.5 | 128 | 134.25 | 87.91 | 100.69 | 94.30 | 9.04 | 0.10 |
| 14-3-3 theta/tau (Phospho-Ser232) | 165 | 123 | 140.5 | 128 | 134.25 | 157.66 | 129.01 | 143.33 | 20.26 | 0.14 |
| 14-3-3 zeta (Ab-58) | 112 | 101 | 140.5 | 128 | 134.25 | 107.02 | 105.93 | 106.47 | 0.77 | 0.01 |
| 14-3-3 zeta (Phospho-Ser58) | 99 | 165 | 140.5 | 128 | 134.25 | 94.60 | 173.06 | 133.83 | 55.48 | 0.41 |
| 14-3-3 zeta/delta (Ab-232) | 1092 | 1214 | 140.5 | 128 | 134.25 | 1043.42 | 1273.28 | 1158.35 | 162.53 | 0.14 |
| 14-3-3 zeta/delta (Phospho-Thr232) | 269 | 233 | 140.5 | 128 | 134.25 | 257.03 | 244.38 | 250.71 | 8.95 | 0.04 |
| 4E-BP1 (Ab-36) | 112 | 77 | 140.5 | 128 | 134.25 | 107.02 | 80.76 | 93.89 | 18.57 | 0.20 |
| 4E-BP1 (Ab-45) | 138 | 93 | 140.5 | 128 | 134.25 | 131.86 | 97.54 | 114.70 | 24.27 | 0.21 |
| 4E-BP1 (Ab-65) | 4343 | 2023 | 140.5 | 128 | 134.25 | 4149.81 | 2121.78 | 3135.79 | 1434.03 | 0.46 |
| 4E-BP1 (Ab-70) | 171 | 250 | 140.5 | 128 | 134.25 | 163.39 | 262.21 | 212.80 | 69.87 | 0.33 |
| 4E-BP1 (Phospho-Ser65) | 133 | 107 | 140.5 | 128 | 134.25 | 127.08 | 112.22 | 119.65 | 10.51 | 0.09 |
| 4E-BP1 (Phospho-Thr36) | 264 | 209 | 140.5 | 128 | 134.25 | 252.26 | 219.21 | 235.73 | 23.37 | 0.10 |
| 4E-BP1 (Phospho-Thr45) | 140 | 96 | 140.5 | 128 | 134.25 | 133.77 | 100.69 | 117.23 | 23.39 | 0.20 |
| 4E-BP1 (Phospho-Thr70) | 129 | 77 | 140.5 | 128 | 134.25 | 123.26 | 80.76 | 102.01 | 30.05 | 0.29 |
| 6-phosphofructo-2-kinase/fructose-2,6-biphosphatase 2 (PFKFB2) (Ab-483) | 96 | 86 | 140.5 | 128 | 134.25 | 91.73 | 90.20 | 90.96 | 1.08 | 0.01 |
| 6-phosphofructo-2-kinase/fructose-2,6-biphosphatase 2 (PFKFB2) (Phospho-Ser483) | 157 | 88 | 140.5 | 128 | 134.25 | 150.02 | 92.30 | 121.16 | 40.81 | 0.34 |
| Abl1 (Ab-204) | 1164 | 3761 | 140.5 | 128 | 134.25 | 1112.22 | 3944.64 | 2528.43 | 2002.82 | 0.79 |
| Abl1 (Ab-754/735) | 78 | 67 | 140.5 | 128 | 134.25 | 74.53 | 70.27 | 72.40 | 3.01 | 0.04 |
| Abl1 (Phospho-Thr754/735) | 134 | 87 | 140.5 | 128 | 134.25 | 128.04 | 91.25 | 109.64 | 26.02 | 0.24 |
| Abl1 (Phospho-Tyr204) | 97 | 291 | 140.5 | 128 | 134.25 | 92.69 | 305.21 | 198.95 | 150.28 | 0.76 |
| Abl1 (Phospho-Tyr412) | 101 | 73 | 140.5 | 128 | 134.25 | 96.51 | 76.56 | 86.54 | 14.10 | 0.16 |
| ACC1 (Ab-79) | 121 | 124 | 140.5 | 128 | 134.25 | 115.62 | 130.05 | 122.84 | 10.21 | 0.08 |
| ACC1 (Ab-80) | 1111 | 1117 | 140.5 | 128 | 134.25 | 1061.58 | 1171.54 | 1116.56 | 77.76 | 0.07 |
| ACC1 (Phospho-Ser79) | 98 | 68 | 140.5 | 128 | 134.25 | 93.64 | 71.32 | 82.48 | 15.78 | 0.19 |
| ACC1 (Phospho-Ser80) | 70 | 63 | 140.5 | 128 | 134.25 | 66.89 | 66.08 | 66.48 | 0.57 | 0.01 |
| ACK1 (Phospho-Tyr284) | 114 | 161 | 140.5 | 128 | 134.25 | 108.93 | 168.86 | 138.90 | 42.38 | 0.31 |
| Actin Pan (a/b/g) (Ab-55/53) | 278 | 144 | 140.5 | 128 | 134.25 | 265.63 | 151.03 | 208.33 | 81.04 | 0.39 |
| Actin Pan (a/b/g) (Phospho-Tyr55/53) | 219 | 92 | 140.5 | 128 | 134.25 | 209.26 | 96.49 | 152.88 | 79.74 | 0.52 |
| ADD1 (Ab-726) | 122 | 115 | 140.5 | 128 | 134.25 | 116.57 | 120.62 | 118.59 | 2.86 | 0.02 |
| ADD1 (Phospho-Ser726) | 175 | 89 | 140.5 | 128 | 134.25 | 167.22 | 93.35 | 130.28 | 52.23 | 0.40 |
| AFX/FOXO4 (Ab-197) | 118 | 96 | 140.5 | 128 | 134.25 | 112.75 | 100.69 | 106.72 | 8.53 | 0.08 |
| AFX/FOXO4 (Phospho-Ser197) | 108 | 96 | 140.5 | 128 | 134.25 | 103.20 | 100.69 | 101.94 | 1.77 | 0.02 |
| AKT1 (Ab-124) | 257 | 238 | 140.5 | 128 | 134.25 | 245.57 | 249.62 | 247.59 | 2.87 | 0.01 |
| AKT1 (Ab-246) | 95 | 83 | 140.5 | 128 | 134.25 | 90.77 | 87.05 | 88.91 | 2.63 | 0.03 |
| AKT1 (Ab-308) | 236 | 229 | 140.5 | 128 | 134.25 | 225.50 | 240.18 | 232.84 | 10.38 | 0.04 |
| AKT1 (Ab-326) | 188 | 177 | 140.5 | 128 | 134.25 | 179.64 | 185.64 | 182.64 | 4.25 | 0.02 |
| AKT1 (Ab-450) | 385 | 373 | 140.5 | 128 | 134.25 | 367.87 | 391.21 | 379.54 | 16.50 | 0.04 |
| AKT1 (Ab-473) | 112 | 74 | 140.5 | 128 | 134.25 | 107.02 | 77.61 | 92.32 | 20.79 | 0.23 |
| AKT1 (Ab-474) | 148 | 101 | 140.5 | 128 | 134.25 | 141.42 | 105.93 | 123.67 | 25.09 | 0.20 |
| AKT1 (Ab-72) | 133 | 92 | 140.5 | 128 | 134.25 | 127.08 | 96.49 | 111.79 | 21.63 | 0.19 |
| AKT1 (Phospho-Ser124) | 82 | 73 | 140.5 | 128 | 134.25 | 78.35 | 76.56 | 77.46 | 1.26 | 0.02 |
| AKT1 (Phospho-Ser246) | 80 | 75 | 140.5 | 128 | 134.25 | 76.44 | 78.66 | 77.55 | 1.57 | 0.02 |
| AKT1 (Phospho-Ser473) | 106 | 87 | 140.5 | 128 | 134.25 | 101.28 | 91.25 | 96.27 | 7.10 | 0.07 |
| AKT1 (Phospho-Thr308) | 333 | 198 | 140.5 | 128 | 134.25 | 318.19 | 207.67 | 262.93 | 78.15 | 0.30 |
| AKT1 (Phospho-Thr450) | 76 | 215 | 140.5 | 128 | 134.25 | 72.62 | 225.50 | 149.06 | 108.10 | 0.73 |
| AKT1 (Phospho-Thr72) | 81 | 79 | 140.5 | 128 | 134.25 | 77.40 | 82.86 | 80.13 | 3.86 | 0.05 |
| AKT1 (Phospho-Tyr326) | 97 | 90 | 140.5 | 128 | 134.25 | 92.69 | 94.39 | 93.54 | 1.21 | 0.01 |
| AKT1 (Phospho-Tyr474) | 82 | 82 | 140.5 | 128 | 134.25 | 78.35 | 86.00 | 82.18 | 5.41 | 0.07 |
| AKT1/2/3 (Ab-315) | 74 | 77 | 140.5 | 128 | 134.25 | 70.71 | 80.76 | 75.73 | 7.11 | 0.09 |
| AKT1S1 (Ab-246) | 2005 | 1423 | 140.5 | 128 | 134.25 | 1915.81 | 1492.48 | 1704.15 | 299.34 | 0.18 |
| AKT1S1 (Phospho-Thr246) | 274 | 222 | 140.5 | 128 | 134.25 | 261.81 | 232.84 | 247.33 | 20.49 | 0.08 |
| AKT2 (Ab-474) | 119 | 104 | 140.5 | 128 | 134.25 | 113.71 | 109.08 | 111.39 | 3.27 | 0.03 |
| AKT2 (Phospho-Ser474) | 180 | 151 | 140.5 | 128 | 134.25 | 171.99 | 158.37 | 165.18 | 9.63 | 0.06 |
| ALK (Ab-1507) | 78 | 77 | 140.5 | 128 | 134.25 | 74.53 | 80.76 | 77.65 | 4.40 | 0.06 |
| ALK (Ab-1604) | 149 | 228 | 140.5 | 128 | 134.25 | 142.37 | 239.13 | 190.75 | 68.42 | 0.36 |
| ALK (Phospho-Tyr1507) | 427 | 321 | 140.5 | 128 | 134.25 | 408.01 | 336.67 | 372.34 | 50.44 | 0.14 |
| ALK (Phospho-Tyr1604) | 794 | 128 | 140.5 | 128 | 134.25 | 758.68 | 134.25 | 446.46 | 441.54 | 0.99 |
| AMPK beta1 (Ab-182) | 355 | 175 | 140.5 | 128 | 134.25 | 339.21 | 183.54 | 261.38 | 110.07 | 0.42 |
| AMPK beta1 (Phospho-Ser182) | 87 | 342 | 140.5 | 128 | 134.25 | 83.13 | 358.70 | 220.91 | 194.86 | 0.88 |
| AMPK1 (Ab-172) | 140 | 103 | 140.5 | 128 | 134.25 | 133.77 | 108.03 | 120.90 | 18.20 | 0.15 |
| AMPK1 (Phospho-Thr172) | 113 | 134 | 140.5 | 128 | 134.25 | 107.97 | 140.54 | 124.26 | 23.03 | 0.19 |
| AMPK1/AMPK2 (Ab-485/491) | 110 | 99 | 140.5 | 128 | 134.25 | 105.11 | 103.83 | 104.47 | 0.90 | 0.01 |
| AMPK1/AMPK2 (Phospho-Ser485/491) | 122 | 113 | 140.5 | 128 | 134.25 | 116.57 | 118.52 | 117.55 | 1.38 | 0.01 |
| Amyloid beta A4 (Ab-743/668) | 403 | 353 | 140.5 | 128 | 134.25 | 385.07 | 370.24 | 377.65 | 10.49 | 0.03 |
| Amyloid beta A4 (Phospho-Thr743/668) | 191 | 188 | 140.5 | 128 | 134.25 | 182.50 | 197.18 | 189.84 | 10.38 | 0.05 |
| Androgen Receptor (Ab-213) | 130 | 128 | 140.5 | 128 | 134.25 | 124.22 | 134.25 | 129.23 | 7.09 | 0.05 |
| Androgen Receptor (Ab-650) | 174 | 161 | 140.5 | 128 | 134.25 | 166.26 | 168.86 | 167.56 | 1.84 | 0.01 |
| Androgen Receptor (Phospho-Ser213) | 103 | 108 | 140.5 | 128 | 134.25 | 98.42 | 113.27 | 105.85 | 10.50 | 0.10 |
| Androgen Receptor (Phospho-Ser650) | 136 | 107 | 140.5 | 128 | 134.25 | 129.95 | 112.22 | 121.09 | 12.53 | 0.10 |
| A-RAF (Ab-301/302) | 121 | 118 | 140.5 | 128 | 134.25 | 115.62 | 123.76 | 119.69 | 5.76 | 0.05 |
| A-RAF (Phospho-Tyr301/302) | 193 | 203 | 140.5 | 128 | 134.25 | 184.41 | 212.91 | 198.66 | 20.15 | 0.10 |
| Arrestin-1 (Ab-412) | 208 | 188 | 140.5 | 128 | 134.25 | 198.75 | 197.18 | 197.96 | 1.11 | 0.01 |
| Arrestin-1 (Phospho-Ser412) | 195 | 78 | 140.5 | 128 | 134.25 | 186.33 | 81.81 | 134.07 | 73.90 | 0.55 |
| ASK1 (Ab-83) | 114 | 96 | 140.5 | 128 | 134.25 | 108.93 | 100.69 | 104.81 | 5.83 | 0.06 |
| ASK1 (Ab-966) | 237 | 221 | 140.5 | 128 | 134.25 | 226.46 | 231.79 | 229.12 | 3.77 | 0.02 |
| ASK1 (Phospho-Ser83) | 161 | 142 | 140.5 | 128 | 134.25 | 153.84 | 148.93 | 151.39 | 3.47 | 0.02 |
| ASK1 (Phospho-Ser966) | 211 | 148 | 140.5 | 128 | 134.25 | 201.61 | 155.23 | 178.42 | 32.80 | 0.18 |
| ATF1 (Ab-63) | 222 | 201 | 140.5 | 128 | 134.25 | 212.12 | 210.81 | 211.47 | 0.93 | 0.00 |
| ATF1 (Phospho-Ser63) | 82 | 75 | 140.5 | 128 | 134.25 | 78.35 | 78.66 | 78.51 | 0.22 | 0.00 |
| ATF2 (Ab-112/94) | 124 | 92 | 140.5 | 128 | 134.25 | 118.48 | 96.49 | 107.49 | 15.55 | 0.14 |
| ATF2 (Ab-62/44) | 1584 | 1518 | 140.5 | 128 | 134.25 | 1513.54 | 1592.12 | 1552.83 | 55.57 | 0.04 |
| ATF2 (Ab-69/51) | 176 | 119 | 140.5 | 128 | 134.25 | 168.17 | 124.81 | 146.49 | 30.66 | 0.21 |
| ATF2 (Ab-71/53) | 111 | 76 | 140.5 | 128 | 134.25 | 106.06 | 79.71 | 92.89 | 18.63 | 0.20 |
| ATF2 (Ab-73/55) | 121 | 83 | 140.5 | 128 | 134.25 | 115.62 | 87.05 | 101.34 | 20.20 | 0.20 |
| ATF2 (Phospho-Ser112/94) | 293 | 132 | 140.5 | 128 | 134.25 | 279.97 | 138.45 | 209.21 | 100.07 | 0.48 |
| ATF2 (Phospho-Ser62/44) | 497 | 464 | 140.5 | 128 | 134.25 | 474.89 | 486.66 | 480.77 | 8.32 | 0.02 |
| ATF2 (Phospho-Thr69/51) | 409 | 110 | 140.5 | 128 | 134.25 | 390.81 | 115.37 | 253.09 | 194.76 | 0.77 |
| ATF2 (Phospho-Thr71/53) | 159 | 101 | 140.5 | 128 | 134.25 | 151.93 | 105.93 | 128.93 | 32.52 | 0.25 |
| ATF2 (Phospho-Thr73/55) | 230 | 140 | 140.5 | 128 | 134.25 | 219.77 | 146.84 | 183.30 | 51.57 | 0.28 |
| ATF4 (Ab-245) | 105 | 81 | 140.5 | 128 | 134.25 | 100.33 | 84.96 | 92.64 | 10.87 | 0.12 |
| ATF4 (Phospho-Ser245) | 371 | 115 | 140.5 | 128 | 134.25 | 354.50 | 120.62 | 237.56 | 165.38 | 0.70 |
| ATM (Ab-1981) | 119 | 83 | 140.5 | 128 | 134.25 | 113.71 | 87.05 | 100.38 | 18.85 | 0.19 |
| ATP1A1/Na+K+ ATPase1 (Ab-23) | 252 | 235 | 140.5 | 128 | 134.25 | 240.79 | 246.47 | 243.63 | 4.02 | 0.02 |
| ATP1A1/Na+K+ ATPase1 (Phospho-Ser23) | 157 | 136 | 140.5 | 128 | 134.25 | 150.02 | 142.64 | 146.33 | 5.22 | 0.04 |
| ATPase (Ab-16) | 85 | 66 | 140.5 | 128 | 134.25 | 81.22 | 69.22 | 75.22 | 8.48 | 0.11 |
| ATPase (Phospho-Ser16) | 82 | 76 | 140.5 | 128 | 134.25 | 78.35 | 79.71 | 79.03 | 0.96 | 0.01 |
| ATP-Citrate Lyase (Ab-454) | 123 | 113 | 140.5 | 128 | 134.25 | 117.53 | 118.52 | 118.02 | 0.70 | 0.01 |
| ATP-Citrate Lyase (Phospho-Ser454) | 155 | 70 | 140.5 | 128 | 134.25 | 148.10 | 73.42 | 110.76 | 52.81 | 0.48 |
| ATRIP (Ab-68/72) | 1797 | 1846 | 140.5 | 128 | 134.25 | 1717.06 | 1936.14 | 1826.60 | 154.91 | 0.08 |
| ATRIP (Phospho-Ser68/72) | 82 | 192 | 140.5 | 128 | 134.25 | 78.35 | 201.38 | 139.86 | 86.99 | 0.62 |
| AurA (Ab-288) | 129 | 103 | 140.5 | 128 | 134.25 | 123.26 | 108.03 | 115.65 | 10.77 | 0.09 |
| AurA (Ab-342) | 117 | 102 | 140.5 | 128 | 134.25 | 111.80 | 106.98 | 109.39 | 3.40 | 0.03 |
| AurA (Phospho-Ser342) | 229 | 195 | 140.5 | 128 | 134.25 | 218.81 | 204.52 | 211.67 | 10.11 | 0.05 |
| AurA (Phospho-Thr288) | 121 | 208 | 140.5 | 128 | 134.25 | 115.62 | 218.16 | 166.89 | 72.51 | 0.43 |
| AurB (Ab-12) | 93 | 80 | 140.5 | 128 | 134.25 | 88.86 | 83.91 | 86.38 | 3.50 | 0.04 |
| AurB (Ab-232) | 95 | 93 | 140.5 | 128 | 134.25 | 90.77 | 97.54 | 94.16 | 4.78 | 0.05 |
| AurB (Phospho-Thr232) | 153 | 140 | 140.5 | 128 | 134.25 | 146.19 | 146.84 | 146.51 | 0.45 | 0.00 |
| AurB (Phospho-Tyr12) | 87 | 97 | 140.5 | 128 | 134.25 | 83.13 | 101.74 | 92.43 | 13.16 | 0.14 |
| AurB/C (Ab-202/175) | 140 | 125 | 140.5 | 128 | 134.25 | 133.77 | 131.10 | 132.44 | 1.89 | 0.01 |
| AXL (Phospho-Tyr691) | 118 | 140 | 140.5 | 128 | 134.25 | 112.75 | 146.84 | 129.79 | 24.10 | 0.19 |
| BAD (Ab-112) | 185 | 169 | 140.5 | 128 | 134.25 | 176.77 | 177.25 | 177.01 | 0.34 | 0.00 |
| BAD (Ab-134) | 131 | 121 | 140.5 | 128 | 134.25 | 125.17 | 126.91 | 126.04 | 1.23 | 0.01 |
| BAD (Ab-136) | 134 | 129 | 140.5 | 128 | 134.25 | 128.04 | 135.30 | 131.67 | 5.13 | 0.04 |
| BAD (Ab-155) | 112 | 97 | 140.5 | 128 | 134.25 | 107.02 | 101.74 | 104.38 | 3.73 | 0.04 |
| BAD (Ab-91/128) | 86 | 81 | 140.5 | 128 | 134.25 | 82.17 | 84.96 | 83.56 | 1.97 | 0.02 |
| BAD (Phospho-Ser112) | 152 | 141 | 140.5 | 128 | 134.25 | 145.24 | 147.88 | 146.56 | 1.87 | 0.01 |
| BAD (Phospho-Ser134) | 89 | 81 | 140.5 | 128 | 134.25 | 85.04 | 84.96 | 85.00 | 0.06 | 0.00 |
| BAD (Phospho-Ser136) | 118 | 132 | 140.5 | 128 | 134.25 | 112.75 | 138.45 | 125.60 | 18.17 | 0.14 |
| BAD (Phospho-Ser155) | 111 | 92 | 140.5 | 128 | 134.25 | 106.06 | 96.49 | 101.28 | 6.77 | 0.07 |
| BAD (Phospho-Ser91/128) | 76 | 103 | 140.5 | 128 | 134.25 | 72.62 | 108.03 | 90.32 | 25.04 | 0.28 |
| BAX (Ab-167) | 146 | 129 | 140.5 | 128 | 134.25 | 139.51 | 135.30 | 137.40 | 2.97 | 0.02 |
| BAX (Phospho-Thr167) | 85 | 74 | 140.5 | 128 | 134.25 | 81.22 | 77.61 | 79.42 | 2.55 | 0.03 |
| BCL-2 (Ab-56) | 126 | 129 | 140.5 | 128 | 134.25 | 120.40 | 135.30 | 127.85 | 10.54 | 0.08 |
| BCL-2 (Ab-69) | 96 | 79 | 140.5 | 128 | 134.25 | 91.73 | 82.86 | 87.29 | 6.27 | 0.07 |
| BCL-2 (Ab-70) | 93 | 93 | 140.5 | 128 | 134.25 | 88.86 | 97.54 | 93.20 | 6.14 | 0.07 |
| BCL-2 (Phospho-Ser70) | 347 | 357 | 140.5 | 128 | 134.25 | 331.56 | 374.43 | 353.00 | 30.31 | 0.09 |
| BCL-2 (Phospho-Ser87) | 103 | 79 | 140.5 | 128 | 134.25 | 98.42 | 82.86 | 90.64 | 11.00 | 0.12 |
| BCL-2 (Phospho-Thr56) | 185 | 128 | 140.5 | 128 | 134.25 | 176.77 | 134.25 | 155.51 | 30.07 | 0.19 |
| BCL-2 (Phospho-Thr69) | 211 | 108 | 140.5 | 128 | 134.25 | 201.61 | 113.27 | 157.44 | 62.47 | 0.40 |
| BCL-6 (Ab-333) | 302 | 263 | 140.5 | 128 | 134.25 | 288.57 | 275.84 | 282.20 | 9.00 | 0.03 |
| BCL-XL (Ab-47) | 1095 | 1239 | 140.5 | 128 | 134.25 | 1046.29 | 1299.50 | 1172.89 | 179.05 | 0.15 |
| BCL-XL (Phospho-Ser62) | 700 | 365 | 140.5 | 128 | 134.25 | 668.86 | 382.82 | 525.84 | 202.26 | 0.38 |
| BCL-XL (Phospho-Thr47) | 83 | 78 | 140.5 | 128 | 134.25 | 79.31 | 81.81 | 80.56 | 1.77 | 0.02 |
| BCR (Ab-177) | 172 | 137 | 140.5 | 128 | 134.25 | 164.35 | 143.69 | 154.02 | 14.61 | 0.09 |
| BCR (Ab-360) | 82 | 78 | 140.5 | 128 | 134.25 | 78.35 | 81.81 | 80.08 | 2.44 | 0.03 |
| BCR (Phospho-Tyr177) | 137 | 130 | 140.5 | 128 | 134.25 | 130.91 | 136.35 | 133.63 | 3.85 | 0.03 |
| BCR (Phospho-Tyr360) | 177 | 125 | 140.5 | 128 | 134.25 | 169.13 | 131.10 | 150.11 | 26.89 | 0.18 |
| Beta actin | 3453 | 3084 | 140.5 | 128 | 134.25 | 3299.40 | 3234.59 | 3266.99 | 45.83 | 0.01 |
| BID (Ab-78) | 270 | 299 | 140.5 | 128 | 134.25 | 257.99 | 313.60 | 285.79 | 39.32 | 0.14 |
| BID (Phospho-Ser78) | 114 | 102 | 140.5 | 128 | 134.25 | 108.93 | 106.98 | 107.95 | 1.38 | 0.01 |
| BIM (Ab-69/65) | 107 | 101 | 140.5 | 128 | 134.25 | 102.24 | 105.93 | 104.09 | 2.61 | 0.03 |
| BIM (Phospho-Ser69/65) | 150 | 162 | 140.5 | 128 | 134.25 | 143.33 | 169.91 | 156.62 | 18.80 | 0.12 |
| BLNK (Ab-96) | 399 | 713 | 140.5 | 128 | 134.25 | 381.25 | 747.81 | 564.53 | 259.20 | 0.46 |
| BLNK (Phospho-Tyr84) | 122 | 105 | 140.5 | 128 | 134.25 | 116.57 | 110.13 | 113.35 | 4.56 | 0.04 |
| BLNK (Phospho-Tyr96) | 212 | 165 | 140.5 | 128 | 134.25 | 202.57 | 173.06 | 187.81 | 20.87 | 0.11 |
| B-RAF (Ab-446) | 90 | 73 | 140.5 | 128 | 134.25 | 86.00 | 76.56 | 81.28 | 6.67 | 0.08 |
| B-RAF (Ab-598) | 1390 | 2394 | 140.5 | 128 | 134.25 | 1328.17 | 2510.89 | 1919.53 | 836.31 | 0.44 |
| B-RAF (Ab-601) | 83 | 74 | 140.5 | 128 | 134.25 | 79.31 | 77.61 | 78.46 | 1.20 | 0.02 |
| B-RAF (Phospho-Ser446) | 253 | 97 | 140.5 | 128 | 134.25 | 241.75 | 101.74 | 171.74 | 99.00 | 0.58 |
| B-RAF (Phospho-Ser601) | 92 | 104 | 140.5 | 128 | 134.25 | 87.91 | 109.08 | 98.49 | 14.97 | 0.15 |
| B-RAF (Phospho-Thr598) | 89 | 84 | 140.5 | 128 | 134.25 | 85.04 | 88.10 | 86.57 | 2.16 | 0.02 |
| BRCA1 (Ab-1457) | 360 | 397 | 140.5 | 128 | 134.25 | 343.99 | 416.38 | 380.19 | 51.19 | 0.13 |
| BRCA1 (Ab-1524) | 366 | 386 | 140.5 | 128 | 134.25 | 349.72 | 404.85 | 377.28 | 38.98 | 0.10 |
| BRCA1 (Phospho-Ser1423) | 756 | 853 | 140.5 | 128 | 134.25 | 722.37 | 894.65 | 808.51 | 121.82 | 0.15 |
| BRCA1 (Phospho-Ser1457) | 78 | 64 | 140.5 | 128 | 134.25 | 74.53 | 67.13 | 70.83 | 5.24 | 0.07 |
| BRCA1 (Phospho-Ser1524) | 116 | 377 | 140.5 | 128 | 134.25 | 110.84 | 395.41 | 253.12 | 201.22 | 0.79 |
| Breast tumor kinase (Phospho-Tyr447) | 116 | 94 | 140.5 | 128 | 134.25 | 110.84 | 98.59 | 104.71 | 8.66 | 0.08 |
| BTK (Ab-223) | 322 | 304 | 140.5 | 128 | 134.25 | 307.68 | 318.84 | 313.26 | 7.90 | 0.03 |
| BTK (Phospho-Tyr223) | 108 | 100 | 140.5 | 128 | 134.25 | 103.20 | 104.88 | 104.04 | 1.19 | 0.01 |
| BTK (Phospho-Tyr550) | 83 | 542 | 140.5 | 128 | 134.25 | 79.31 | 568.46 | 323.89 | 345.89 | 1.07 |
| c-Abl (Ab-412) | 185 | 134 | 140.5 | 128 | 134.25 | 176.77 | 140.54 | 158.66 | 25.62 | 0.16 |
| c-Abl (Phospho-Tyr245) | 79 | 195 | 140.5 | 128 | 134.25 | 75.49 | 204.52 | 140.00 | 91.24 | 0.65 |
| c-Abl (Phospho-Tyr412) | 194 | 246 | 140.5 | 128 | 134.25 | 185.37 | 258.01 | 221.69 | 51.37 | 0.23 |
| Calmodulin (Ab-79/81) | 1029 | 74 | 140.5 | 128 | 134.25 | 983.23 | 77.61 | 530.42 | 640.36 | 1.21 |
| Calmodulin (Phospho-Thr79/Ser81) | 118 | 128 | 140.5 | 128 | 134.25 | 112.75 | 134.25 | 123.50 | 15.20 | 0.12 |
| Calsenilin/KCNIP3 (Ab-63) | 1873 | 2058 | 140.5 | 128 | 134.25 | 1789.68 | 2158.49 | 1974.08 | 260.79 | 0.13 |
| Calsenilin/KCNIP3 (Phospho-Ser63) | 908 | 711 | 140.5 | 128 | 134.25 | 867.61 | 745.72 | 806.66 | 86.19 | 0.11 |
| CaMK1-alpha (Ab-177) | 337 | 70 | 140.5 | 128 | 134.25 | 322.01 | 73.42 | 197.71 | 175.78 | 0.89 |
| CaMK1-alpha (Phospho-Thr177) | 246 | 204 | 140.5 | 128 | 134.25 | 235.06 | 213.96 | 224.51 | 14.92 | 0.07 |
| CaMK2 alpha/beta/delta (Phospho-Thr305) | 78 | 126 | 140.5 | 128 | 134.25 | 74.53 | 132.15 | 103.34 | 40.74 | 0.39 |
| CaMK2A (Ab-286) | 280 | 256 | 140.5 | 128 | 134.25 | 267.54 | 268.50 | 268.02 | 0.68 | 0.00 |
| CaMK2A (Phospho-Thr286) | 108 | 97 | 140.5 | 128 | 134.25 | 103.20 | 101.74 | 102.47 | 1.03 | 0.01 |
| CaMK2-beta/gamma/delta (Ab-287) | 526 | 492 | 140.5 | 128 | 134.25 | 502.60 | 516.02 | 509.31 | 9.49 | 0.02 |
| CaMK2-beta/gamma/delta (Phospho-Thr287) | 203 | 191 | 140.5 | 128 | 134.25 | 193.97 | 200.33 | 197.15 | 4.49 | 0.02 |
| CaMK4 (Ab-196/200) | 91 | 71 | 140.5 | 128 | 134.25 | 86.95 | 74.47 | 80.71 | 8.83 | 0.11 |
| CaMK4 (Phospho-Thr196/200) | 76 | 252 | 140.5 | 128 | 134.25 | 72.62 | 264.30 | 168.46 | 135.54 | 0.80 |
| Caspase 1 (Ab-376) | 225 | 176 | 140.5 | 128 | 134.25 | 214.99 | 184.59 | 199.79 | 21.49 | 0.11 |
| Caspase 1 (Phospho-Ser376) | 162 | 100 | 140.5 | 128 | 134.25 | 154.79 | 104.88 | 129.84 | 35.29 | 0.27 |
| Caspase 2 (Ab-157) | 210 | 89 | 140.5 | 128 | 134.25 | 200.66 | 93.35 | 147.00 | 75.88 | 0.52 |
| Caspase 2 (Phospho-Ser157) | 195 | 175 | 140.5 | 128 | 134.25 | 186.33 | 183.54 | 184.94 | 1.97 | 0.01 |
| Caspase 3 (Ab-150) | 82 | 74 | 140.5 | 128 | 134.25 | 78.35 | 77.61 | 77.98 | 0.52 | 0.01 |
| Caspase 3 (Phospho-Ser150) | 71 | 60 | 140.5 | 128 | 134.25 | 67.84 | 62.93 | 65.39 | 3.47 | 0.05 |
| Caspase 6 (Ab-257) | 115 | 113 | 140.5 | 128 | 134.25 | 109.88 | 118.52 | 114.20 | 6.10 | 0.05 |
| Caspase 6 (Phospho-Ser257) | 74 | 63 | 140.5 | 128 | 134.25 | 70.71 | 66.08 | 68.39 | 3.28 | 0.05 |
| Caspase 8 (Ab-347) | 157 | 114 | 140.5 | 128 | 134.25 | 150.02 | 119.57 | 134.79 | 21.53 | 0.16 |
| Caspase 8 (Phospho-Ser347) | 113 | 87 | 140.5 | 128 | 134.25 | 107.97 | 91.25 | 99.61 | 11.83 | 0.12 |
| Caspase 9 (Ab-125) | 352 | 95 | 140.5 | 128 | 134.25 | 336.34 | 99.64 | 217.99 | 167.37 | 0.77 |
| Caspase 9 (Ab-144) | 84 | 69 | 140.5 | 128 | 134.25 | 80.26 | 72.37 | 76.32 | 5.58 | 0.07 |
| Caspase 9 (Ab-153) | 258 | 385 | 140.5 | 128 | 134.25 | 246.52 | 403.80 | 325.16 | 111.21 | 0.34 |
| Caspase 9 (Ab-196) | 1967 | 2049 | 140.5 | 128 | 134.25 | 1879.50 | 2149.05 | 2014.27 | 190.60 | 0.09 |
| Caspase 9 (Phospho-Ser144) | 117 | 72 | 140.5 | 128 | 134.25 | 111.80 | 75.52 | 93.66 | 25.65 | 0.27 |
| Caspase 9 (Phospho-Ser196) | 685 | 480 | 140.5 | 128 | 134.25 | 654.53 | 503.44 | 578.98 | 106.84 | 0.18 |
| Caspase 9 (Phospho-Thr125) | 74 | 63 | 140.5 | 128 | 134.25 | 70.71 | 66.08 | 68.39 | 3.28 | 0.05 |
| Caspase 9 (Phospho-Tyr153) | 114 | 347 | 140.5 | 128 | 134.25 | 108.93 | 363.94 | 236.44 | 180.32 | 0.76 |
| Catalase (Ab-385) | 419 | 446 | 140.5 | 128 | 134.25 | 400.36 | 467.78 | 434.07 | 47.67 | 0.11 |
| Catalase (Phospho-Tyr385) | 112 | 144 | 140.5 | 128 | 134.25 | 107.02 | 151.03 | 129.02 | 31.12 | 0.24 |
| Catenin beta (Ab-33) | 95 | 85 | 140.5 | 128 | 134.25 | 90.77 | 89.15 | 89.96 | 1.15 | 0.01 |
| Catenin beta (Ab-37) | 377 | 341 | 140.5 | 128 | 134.25 | 360.23 | 357.65 | 358.94 | 1.82 | 0.01 |
| Catenin beta (Ab-41/45) | 182 | 142 | 140.5 | 128 | 134.25 | 173.90 | 148.93 | 161.42 | 17.66 | 0.11 |
| Catenin beta (Ab-489) | 84 | 79 | 140.5 | 128 | 134.25 | 80.26 | 82.86 | 81.56 | 1.83 | 0.02 |
| Catenin beta (Ab-654) | 170 | 195 | 140.5 | 128 | 134.25 | 162.44 | 204.52 | 183.48 | 29.76 | 0.16 |
| Catenin beta (Phospho-Ser33) | 306 | 265 | 140.5 | 128 | 134.25 | 292.39 | 277.94 | 285.16 | 10.22 | 0.04 |
| Catenin beta (Phospho-Ser37) | 115 | 71 | 140.5 | 128 | 134.25 | 109.88 | 74.47 | 92.18 | 25.04 | 0.27 |
| Catenin beta (Phospho-Thr41/Ser45) | 122 | 107 | 140.5 | 128 | 134.25 | 116.57 | 112.22 | 114.40 | 3.07 | 0.03 |
| Catenin beta (Phospho-Tyr489) | 87 | 93 | 140.5 | 128 | 134.25 | 83.13 | 97.54 | 90.34 | 10.19 | 0.11 |
| Catenin beta (Phospho-Tyr654) | 129 | 77 | 140.5 | 128 | 134.25 | 123.26 | 80.76 | 102.01 | 30.05 | 0.29 |
| Catenin delta-1 (Ab-228) | 143 | 146 | 140.5 | 128 | 134.25 | 136.64 | 153.13 | 144.88 | 11.66 | 0.08 |
| Catenin delta-1 (Phospho-Tyr228) | 345 | 315 | 140.5 | 128 | 134.25 | 329.65 | 330.38 | 330.02 | 0.51 | 0.00 |
| Caveolin-1 (Ab-14) | 101 | 105 | 140.5 | 128 | 134.25 | 96.51 | 110.13 | 103.32 | 9.63 | 0.09 |
| Caveolin-1 (Phospho-Tyr14) | 141 | 945 | 140.5 | 128 | 134.25 | 134.73 | 991.14 | 562.94 | 605.58 | 1.08 |
| CBL (Phospho-Tyr700) | 287 | 487 | 140.5 | 128 | 134.25 | 274.23 | 510.78 | 392.51 | 167.26 | 0.43 |
| CBL (Phospho-Tyr774) | 78 | 90 | 140.5 | 128 | 134.25 | 74.53 | 94.39 | 84.46 | 14.05 | 0.17 |
| CD19 (Ab-531) | 81 | 65 | 140.5 | 128 | 134.25 | 77.40 | 68.17 | 72.79 | 6.52 | 0.09 |
| CD19 (Phospho-Tyr531) | 135 | 114 | 140.5 | 128 | 134.25 | 128.99 | 119.57 | 124.28 | 6.67 | 0.05 |
| CD22/BL-CAM (Phospho-Tyr807) | 196 | 77 | 140.5 | 128 | 134.25 | 187.28 | 80.76 | 134.02 | 75.32 | 0.56 |
| CD227/mucin 1 (Ab-1243) | 118 | 95 | 140.5 | 128 | 134.25 | 112.75 | 99.64 | 106.19 | 9.27 | 0.09 |
| CD227/mucin 1 (Phospho-Tyr1243) | 201 | 136 | 140.5 | 128 | 134.25 | 192.06 | 142.64 | 167.35 | 34.94 | 0.21 |
| CD28 (Phospho-Tyr218) | 95 | 108 | 140.5 | 128 | 134.25 | 90.77 | 113.27 | 102.02 | 15.91 | 0.16 |
| CD32 (FcgammaRIIb) (Ab-292) | 1160 | 924 | 140.5 | 128 | 134.25 | 1108.40 | 969.12 | 1038.76 | 98.49 | 0.09 |
| CD3Z (Ab-142) | 148 | 156 | 140.5 | 128 | 134.25 | 141.42 | 163.62 | 152.52 | 15.70 | 0.10 |
| CD3Z (Phospho-Tyr142) | 102 | 280 | 140.5 | 128 | 134.25 | 97.46 | 293.67 | 195.57 | 138.74 | 0.71 |
| CD4 (Ab-433) | 840 | 1265 | 140.5 | 128 | 134.25 | 802.63 | 1326.77 | 1064.70 | 370.62 | 0.35 |
| CD4 (Phospho-Ser433) | 187 | 178 | 140.5 | 128 | 134.25 | 178.68 | 186.69 | 182.69 | 5.66 | 0.03 |
| CD45 (Phospho-Ser1007) | 345 | 85 | 140.5 | 128 | 134.25 | 329.65 | 89.15 | 209.40 | 170.06 | 0.81 |
| CD5 (Ab-453) | 608 | 553 | 140.5 | 128 | 134.25 | 580.95 | 580.00 | 580.48 | 0.67 | 0.00 |
| CD5 (Phospho-Tyr453) | 156 | 88 | 140.5 | 128 | 134.25 | 149.06 | 92.30 | 120.68 | 40.14 | 0.33 |
| CDC25A (Ab-124) | 81 | 74 | 140.5 | 128 | 134.25 | 77.40 | 77.61 | 77.51 | 0.15 | 0.00 |
| CDC25A (Ab-178) | 149 | 125 | 140.5 | 128 | 134.25 | 142.37 | 131.10 | 136.74 | 7.97 | 0.06 |
| CDC25A (Ab-75) | 77 | 236 | 140.5 | 128 | 134.25 | 73.57 | 247.52 | 160.55 | 123.00 | 0.77 |
| CDC25A (Phospho-Ser124) | 149 | 146 | 140.5 | 128 | 134.25 | 142.37 | 153.13 | 147.75 | 7.61 | 0.05 |
| CDC25A (Phospho-Ser75) | 125 | 114 | 140.5 | 128 | 134.25 | 119.44 | 119.57 | 119.50 | 0.09 | 0.00 |
| CDC25B (Ab-323) | 99 | 79 | 140.5 | 128 | 134.25 | 94.60 | 82.86 | 88.73 | 8.30 | 0.09 |
| CDC25B (Ab-353) | 76 | 70 | 140.5 | 128 | 134.25 | 72.62 | 73.42 | 73.02 | 0.56 | 0.01 |
| CDC25B (Phospho-Ser323) | 102 | 66 | 140.5 | 128 | 134.25 | 97.46 | 69.22 | 83.34 | 19.97 | 0.24 |
| CDC25B (Phospho-Ser353) | 125 | 364 | 140.5 | 128 | 134.25 | 119.44 | 381.77 | 250.61 | 185.50 | 0.74 |
| CDC25C (Ab-216) | 2380 | 1850 | 140.5 | 128 | 134.25 | 2274.13 | 1940.33 | 2107.23 | 236.03 | 0.11 |
| CDC25C (Phospho-Ser216) | 146 | 339 | 140.5 | 128 | 134.25 | 139.51 | 355.55 | 247.53 | 152.77 | 0.62 |
| CDC25C (Phospho-Thr48) | 144 | 132 | 140.5 | 128 | 134.25 | 137.59 | 138.45 | 138.02 | 0.60 | 0.00 |
| CDK1/CDC2 (Ab-14) | 84 | 86 | 140.5 | 128 | 134.25 | 80.26 | 90.20 | 85.23 | 7.03 | 0.08 |
| CDK1/CDC2 (Ab-15) | 101 | 81 | 140.5 | 128 | 134.25 | 96.51 | 84.96 | 90.73 | 8.17 | 0.09 |
| CDK1/CDC2 (Phospho-Thr14) | 213 | 202 | 140.5 | 128 | 134.25 | 203.52 | 211.86 | 207.69 | 5.90 | 0.03 |
| CDK1/CDC2 (Phospho-Tyr15) | 187 | 170 | 140.5 | 128 | 134.25 | 178.68 | 178.30 | 178.49 | 0.27 | 0.00 |
| CDK2 (Ab-160) | 104 | 105 | 140.5 | 128 | 134.25 | 99.37 | 110.13 | 104.75 | 7.60 | 0.07 |
| CDK2 (Phospho-Thr160) | 101 | 93 | 140.5 | 128 | 134.25 | 96.51 | 97.54 | 97.02 | 0.73 | 0.01 |
| CDK5 (Ab-15) | 1984 | 1723 | 140.5 | 128 | 134.25 | 1895.74 | 1807.13 | 1851.44 | 62.66 | 0.03 |
| CDK5 (Phospho-Tyr15) | 1139 | 319 | 140.5 | 128 | 134.25 | 1088.33 | 334.58 | 711.45 | 532.99 | 0.75 |
| CDK7 (Ab-170) | 91 | 90 | 140.5 | 128 | 134.25 | 86.95 | 94.39 | 90.67 | 5.26 | 0.06 |
| CDK7 (Phospho-Thr170) | 437 | 105 | 140.5 | 128 | 134.25 | 417.56 | 110.13 | 263.84 | 217.39 | 0.82 |
| Chk1 (Ab-280) | 122 | 84 | 140.5 | 128 | 134.25 | 116.57 | 88.10 | 102.34 | 20.13 | 0.20 |
| Chk1 (Ab-286) | 863 | 816 | 140.5 | 128 | 134.25 | 824.61 | 855.84 | 840.23 | 22.09 | 0.03 |
| Chk1 (Ab-317) | 373 | 330 | 140.5 | 128 | 134.25 | 356.41 | 346.11 | 351.26 | 7.28 | 0.02 |
| Chk1 (Ab-345) | 125 | 95 | 140.5 | 128 | 134.25 | 119.44 | 99.64 | 109.54 | 14.00 | 0.13 |
| Chk1 (Phospho-Ser280) | 345 | 327 | 140.5 | 128 | 134.25 | 329.65 | 342.97 | 336.31 | 9.41 | 0.03 |
| Chk1 (Phospho-Ser286) | 82 | 271 | 140.5 | 128 | 134.25 | 78.35 | 284.23 | 181.29 | 145.58 | 0.80 |
| Chk1 (Phospho-Ser296) | 225 | 236 | 140.5 | 128 | 134.25 | 214.99 | 247.52 | 231.26 | 23.00 | 0.10 |
| Chk1 (Phospho-Ser301) | 67 | 74 | 140.5 | 128 | 134.25 | 64.02 | 77.61 | 70.82 | 9.61 | 0.14 |
| Chk1 (Phospho-Ser317) | 82 | 79 | 140.5 | 128 | 134.25 | 78.35 | 82.86 | 80.60 | 3.19 | 0.04 |
| Chk1 (Phospho-Ser345) | 105 | 83 | 140.5 | 128 | 134.25 | 100.33 | 87.05 | 93.69 | 9.39 | 0.10 |
| Chk2 (Ab-383) | 200 | 131 | 140.5 | 128 | 134.25 | 191.10 | 137.40 | 164.25 | 37.98 | 0.23 |
| Chk2 (Ab-387) | 307 | 274 | 140.5 | 128 | 134.25 | 293.34 | 287.38 | 290.36 | 4.22 | 0.01 |
| Chk2 (Ab-516) | 231 | 213 | 140.5 | 128 | 134.25 | 220.72 | 223.40 | 222.06 | 1.89 | 0.01 |
| Chk2 (Ab-68) | 1786 | 1724 | 140.5 | 128 | 134.25 | 1706.55 | 1808.18 | 1757.37 | 71.86 | 0.04 |
| Chk2 (Phospho-Ser516) | 179 | 136 | 140.5 | 128 | 134.25 | 171.04 | 142.64 | 156.84 | 20.08 | 0.13 |
| Chk2 (Phospho-Thr383) | 80 | 68 | 140.5 | 128 | 134.25 | 76.44 | 71.32 | 73.88 | 3.62 | 0.05 |
| Chk2 (Phospho-Thr387) | 84 | 227 | 140.5 | 128 | 134.25 | 80.26 | 238.08 | 159.17 | 111.60 | 0.70 |
| Chk2 (Phospho-Thr68) | 381 | 482 | 140.5 | 128 | 134.25 | 364.05 | 505.54 | 434.79 | 100.04 | 0.23 |
| c-Jun (Ab-170) | 273 | 201 | 140.5 | 128 | 134.25 | 260.86 | 210.81 | 235.84 | 35.38 | 0.15 |
| c-Jun (Ab-239) | 248 | 201 | 140.5 | 128 | 134.25 | 236.97 | 210.81 | 223.89 | 18.49 | 0.08 |
| c-Jun (Ab-243) | 228 | 123 | 140.5 | 128 | 134.25 | 217.86 | 129.01 | 173.43 | 62.83 | 0.36 |
| c-Jun (Ab-63) | 350 | 208 | 140.5 | 128 | 134.25 | 334.43 | 218.16 | 276.29 | 82.22 | 0.30 |
| c-Jun (Ab-91) | 131 | 111 | 140.5 | 128 | 134.25 | 125.17 | 116.42 | 120.80 | 6.19 | 0.05 |
| c-Jun (Ab-93) | 110 | 88 | 140.5 | 128 | 134.25 | 105.11 | 92.30 | 98.70 | 9.06 | 0.09 |
| c-Jun (Phospho-Ser243) | 548 | 366 | 140.5 | 128 | 134.25 | 523.62 | 383.87 | 453.75 | 98.82 | 0.22 |
| c-Jun (Phospho-Ser63) | 177 | 332 | 140.5 | 128 | 134.25 | 169.13 | 348.21 | 258.67 | 126.63 | 0.49 |
| c-Jun (Phospho-Ser73) | 324 | 359 | 140.5 | 128 | 134.25 | 309.59 | 376.53 | 343.06 | 47.34 | 0.14 |
| c-Jun (Phospho-Thr239) | 158 | 145 | 140.5 | 128 | 134.25 | 150.97 | 152.08 | 151.53 | 0.78 | 0.01 |
| c-Jun (Phospho-Thr91) | 168 | 136 | 140.5 | 128 | 134.25 | 160.53 | 142.64 | 151.58 | 12.65 | 0.08 |
| c-Jun (Phospho-Thr93) | 129 | 112 | 140.5 | 128 | 134.25 | 123.26 | 117.47 | 120.37 | 4.10 | 0.03 |
| c-Jun (Phospho-Tyr170) | 259 | 94 | 140.5 | 128 | 134.25 | 247.48 | 98.59 | 173.03 | 105.28 | 0.61 |
| CK1-A (Ab-321) | 88 | 80 | 140.5 | 128 | 134.25 | 84.09 | 83.91 | 84.00 | 0.13 | 0.00 |
| CK1-A (Phospho-Thr321) | 257 | 231 | 140.5 | 128 | 134.25 | 245.57 | 242.28 | 243.92 | 2.33 | 0.01 |
| CK1-A/A2 (Phospho-Tyr294) | 124 | 160 | 140.5 | 128 | 134.25 | 118.48 | 167.81 | 143.15 | 34.88 | 0.24 |
| CK2-b (Ab-209) | 112 | 120 | 140.5 | 128 | 134.25 | 107.02 | 125.86 | 116.44 | 13.32 | 0.11 |
| CK2-b (Phospho-Ser209) | 110 | 98 | 140.5 | 128 | 134.25 | 105.11 | 102.79 | 103.95 | 1.64 | 0.02 |
| Claudin 3 (Ab-219) | 86 | 79 | 140.5 | 128 | 134.25 | 82.17 | 82.86 | 82.52 | 0.48 | 0.01 |
| Claudin 3 (Phospho-Tyr219) | 266 | 306 | 140.5 | 128 | 134.25 | 254.17 | 320.94 | 287.55 | 47.22 | 0.16 |
| Claudin 6 (Phospho-Tyr219) | 125 | 131 | 140.5 | 128 | 134.25 | 119.44 | 137.40 | 128.42 | 12.70 | 0.10 |
| Claudin 7 (Ab-210) | 99 | 89 | 140.5 | 128 | 134.25 | 94.60 | 93.35 | 93.97 | 0.88 | 0.01 |
| Claudin 7 (Phospho-Tyr210) | 196 | 205 | 140.5 | 128 | 134.25 | 187.28 | 215.01 | 201.15 | 19.61 | 0.10 |
| Coagulation Factor III (Phospho-Ser290) | 122 | 98 | 140.5 | 128 | 134.25 | 116.57 | 102.79 | 109.68 | 9.75 | 0.09 |
| Cofilin (Ab-3) | 132 | 94 | 140.5 | 128 | 134.25 | 126.13 | 98.59 | 112.36 | 19.47 | 0.17 |
| Cofilin (Phospho-Ser3) | 133 | 138 | 140.5 | 128 | 134.25 | 127.08 | 144.74 | 135.91 | 12.48 | 0.09 |
| Connexin 43 (Ab-367) | 269 | 276 | 140.5 | 128 | 134.25 | 257.03 | 289.48 | 273.26 | 22.94 | 0.08 |
| Connexin 43 (Phospho-Ser367) | 479 | 190 | 140.5 | 128 | 134.25 | 457.69 | 199.28 | 328.48 | 182.73 | 0.56 |
| Cortactin (Ab-421) | 116 | 89 | 140.5 | 128 | 134.25 | 110.84 | 93.35 | 102.09 | 12.37 | 0.12 |
| Cortactin (Ab-466) | 154 | 137 | 140.5 | 128 | 134.25 | 147.15 | 143.69 | 145.42 | 2.45 | 0.02 |
| Cortactin (Phospho-Tyr421) | 754 | 518 | 140.5 | 128 | 134.25 | 720.46 | 543.29 | 631.88 | 125.28 | 0.20 |
| Cortactin (Phospho-Tyr466) | 202 | 111 | 140.5 | 128 | 134.25 | 193.01 | 116.42 | 154.72 | 54.16 | 0.35 |
| CPI17 alpha (Ab-38) | 127 | 121 | 140.5 | 128 | 134.25 | 121.35 | 126.91 | 124.13 | 3.93 | 0.03 |
| CPI17 alpha (Phospho-Thr38) | 146 | 118 | 140.5 | 128 | 134.25 | 139.51 | 123.76 | 131.63 | 11.13 | 0.08 |
| c-PLA2 (Ab-505) | 90 | 89 | 140.5 | 128 | 134.25 | 86.00 | 93.35 | 89.67 | 5.20 | 0.06 |
| c-PLA2 (Phospho-Ser505) | 250 | 79 | 140.5 | 128 | 134.25 | 238.88 | 82.86 | 160.87 | 110.32 | 0.69 |
| CREB (Ab-100) | 87 | 84 | 140.5 | 128 | 134.25 | 83.13 | 88.10 | 85.62 | 3.52 | 0.04 |
| CREB (Ab-121) | 115 | 104 | 140.5 | 128 | 134.25 | 109.88 | 109.08 | 109.48 | 0.57 | 0.01 |
| CREB (Ab-129) | 87 | 70 | 140.5 | 128 | 134.25 | 83.13 | 73.42 | 78.27 | 6.87 | 0.09 |
| CREB (Ab-133) | 111 | 86 | 140.5 | 128 | 134.25 | 106.06 | 90.20 | 98.13 | 11.22 | 0.11 |
| CREB (Ab-142) | 157 | 109 | 140.5 | 128 | 134.25 | 150.02 | 114.32 | 132.17 | 25.24 | 0.19 |
| CREB (Phospho-Ser121) | 177 | 105 | 140.5 | 128 | 134.25 | 169.13 | 110.13 | 139.63 | 41.72 | 0.30 |
| CREB (Phospho-Ser129) | 96 | 74 | 140.5 | 128 | 134.25 | 91.73 | 77.61 | 84.67 | 9.98 | 0.12 |
| CREB (Phospho-Ser133) | 99 | 95 | 140.5 | 128 | 134.25 | 94.60 | 99.64 | 97.12 | 3.57 | 0.04 |
| CREB (Phospho-Ser142) | 90 | 195 | 140.5 | 128 | 134.25 | 86.00 | 204.52 | 145.26 | 83.81 | 0.58 |
| CREB (Phospho-Thr100) | 182 | 219 | 140.5 | 128 | 134.25 | 173.90 | 229.69 | 201.80 | 39.45 | 0.20 |
| CrkII (Ab-221) | 481 | 425 | 140.5 | 128 | 134.25 | 459.60 | 445.75 | 452.68 | 9.79 | 0.02 |
| CrkII (Phospho-Tyr221) | 357 | 348 | 140.5 | 128 | 134.25 | 341.12 | 364.99 | 353.06 | 16.88 | 0.05 |
| CrkL (Phospho-Tyr207) | 86 | 96 | 140.5 | 128 | 134.25 | 82.17 | 100.69 | 91.43 | 13.09 | 0.14 |
| CXCR4 (Phospho-Ser339) | 73 | 271 | 140.5 | 128 | 134.25 | 69.75 | 284.23 | 176.99 | 151.66 | 0.86 |
| Cyclin B1 (Ab-126) | 156 | 709 | 140.5 | 128 | 134.25 | 149.06 | 743.62 | 446.34 | 420.42 | 0.94 |
| Cyclin B1 (Ab-147) | 92 | 76 | 140.5 | 128 | 134.25 | 87.91 | 79.71 | 83.81 | 5.80 | 0.07 |
| Cyclin B1 (Phospho-Ser126) | 159 | 89 | 140.5 | 128 | 134.25 | 151.93 | 93.35 | 122.64 | 41.42 | 0.34 |
| Cyclin B1 (Phospho-Ser147) | 112 | 86 | 140.5 | 128 | 134.25 | 107.02 | 90.20 | 98.61 | 11.89 | 0.12 |
| Cyclin C (Phospho-Ser275) | 99 | 105 | 140.5 | 128 | 134.25 | 94.60 | 110.13 | 102.36 | 10.98 | 0.11 |
| Cyclin D1 (Ab-286) | 1095 | 1393 | 140.5 | 128 | 134.25 | 1046.29 | 1461.02 | 1253.65 | 293.26 | 0.23 |
| Cyclin D1 (Ab-90) | 173 | 161 | 140.5 | 128 | 134.25 | 165.30 | 168.86 | 167.08 | 2.52 | 0.02 |
| Cyclin D1 (Phospho-Thr286) | 673 | 514 | 140.5 | 128 | 134.25 | 643.06 | 539.10 | 591.08 | 73.51 | 0.12 |
| Cyclin D2 (Ab-280) | 92 | 478 | 140.5 | 128 | 134.25 | 87.91 | 501.34 | 294.62 | 292.34 | 0.99 |
| Cyclin D3 (Ab-283) | 392 | 181 | 140.5 | 128 | 134.25 | 374.56 | 189.84 | 282.20 | 130.62 | 0.46 |
| Cyclin D3 (Phospho-Thr283) | 64 | 60 | 140.5 | 128 | 134.25 | 61.15 | 62.93 | 62.04 | 1.26 | 0.02 |
| Cyclin E1 (Ab-395) | 152 | 135 | 140.5 | 128 | 134.25 | 145.24 | 141.59 | 143.42 | 2.58 | 0.02 |
| Cyclin E1 (Ab-77) | 2206 | 2364 | 140.5 | 128 | 134.25 | 2107.87 | 2479.43 | 2293.65 | 262.73 | 0.11 |
| Cyclin E1 (Phospho-Thr395) | 156 | 127 | 140.5 | 128 | 134.25 | 149.06 | 133.20 | 141.13 | 11.21 | 0.08 |
| Cyclin E1 (Phospho-Thr77) | 342 | 235 | 140.5 | 128 | 134.25 | 326.79 | 246.47 | 286.63 | 56.79 | 0.20 |
| Cyclin E2 (Ab-392) | 190 | 165 | 140.5 | 128 | 134.25 | 181.55 | 173.06 | 177.30 | 6.00 | 0.03 |
| Cyclin E2 (Phospho-Thr392) | 213 | 201 | 140.5 | 128 | 134.25 | 203.52 | 210.81 | 207.17 | 5.15 | 0.02 |
| DAB1 (Ab-220) | 132 | 110 | 140.5 | 128 | 134.25 | 126.13 | 115.37 | 120.75 | 7.61 | 0.06 |
| DAB1 (Ab-232) | 130 | 98 | 140.5 | 128 | 134.25 | 124.22 | 102.79 | 113.50 | 15.15 | 0.13 |
| DAB1 (Phospho-Tyr220) | 142 | 141 | 140.5 | 128 | 134.25 | 135.68 | 147.88 | 141.78 | 8.63 | 0.06 |
| DAB1 (Phospho-Tyr232) | 88 | 88 | 140.5 | 128 | 134.25 | 84.09 | 92.30 | 88.19 | 5.81 | 0.07 |
| DAPP1 (Ab-139) | 111 | 93 | 140.5 | 128 | 134.25 | 106.06 | 97.54 | 101.80 | 6.03 | 0.06 |
| DAPP1 (Phospho-Tyr139) | 83 | 138 | 140.5 | 128 | 134.25 | 79.31 | 144.74 | 112.02 | 46.27 | 0.41 |
| DARPP-32 (Ab-34) | 279 | 517 | 140.5 | 128 | 134.25 | 266.59 | 542.24 | 404.42 | 194.92 | 0.48 |
| DARPP-32 (Ab-75) | 209 | 227 | 140.5 | 128 | 134.25 | 199.70 | 238.08 | 218.89 | 27.14 | 0.12 |
| DARPP-32 (Phospho-Thr34) | 168 | 133 | 140.5 | 128 | 134.25 | 160.53 | 139.49 | 150.01 | 14.87 | 0.10 |
| DARPP-32 (Phospho-Thr75) | 188 | 215 | 140.5 | 128 | 134.25 | 179.64 | 225.50 | 202.57 | 32.43 | 0.16 |
| DAXX (Phospho-Ser668) | 356 | 260 | 140.5 | 128 | 134.25 | 340.16 | 272.70 | 306.43 | 47.71 | 0.16 |
| DDX5/DEAD-box protein 5 (Ab-593) | 126 | 91 | 140.5 | 128 | 134.25 | 120.40 | 95.44 | 107.92 | 17.64 | 0.16 |
| DDX5/DEAD-box protein 5 (Phospho-Tyr593) | 163 | 563 | 140.5 | 128 | 134.25 | 155.75 | 590.49 | 373.12 | 307.41 | 0.82 |
| DNA-PK (Ab-2056) | 78 | 71 | 140.5 | 128 | 134.25 | 74.53 | 74.47 | 74.50 | 0.04 | 0.00 |
| DNA-PK (Ab-2638) | 223 | 214 | 140.5 | 128 | 134.25 | 213.08 | 224.45 | 218.76 | 8.04 | 0.04 |
| DNA-PK (Ab-2647) | 80 | 253 | 140.5 | 128 | 134.25 | 76.44 | 265.35 | 170.90 | 133.58 | 0.78 |
| DNA-PK (Phospho-Thr2638) | 107 | 80 | 140.5 | 128 | 134.25 | 102.24 | 83.91 | 93.07 | 12.96 | 0.14 |
| DNA-PK (Phospho-Thr2647) | 93 | 69 | 140.5 | 128 | 134.25 | 88.86 | 72.37 | 80.62 | 11.66 | 0.14 |
| Dok-1 (Ab-362) | 128 | 107 | 140.5 | 128 | 134.25 | 122.31 | 112.22 | 117.27 | 7.13 | 0.06 |
| Dok-1 (Ab-398) | 207 | 180 | 140.5 | 128 | 134.25 | 197.79 | 188.79 | 193.29 | 6.37 | 0.03 |
| Dok-1 (Phospho-Tyr362) | 323 | 107 | 140.5 | 128 | 134.25 | 308.63 | 112.22 | 210.43 | 138.88 | 0.66 |
| Dok-1 (Phospho-Tyr398) | 100 | 88 | 140.5 | 128 | 134.25 | 95.55 | 92.30 | 93.92 | 2.30 | 0.02 |
| Dok-2 (Ab-299) | 220 | 190 | 140.5 | 128 | 134.25 | 210.21 | 199.28 | 204.75 | 7.73 | 0.04 |
| Dok-2 (Phospho-Tyr299) | 370 | 312 | 140.5 | 128 | 134.25 | 353.54 | 327.23 | 340.39 | 18.60 | 0.05 |
| DYN1 (Ab-774) | 2977 | 2625 | 140.5 | 128 | 134.25 | 2844.57 | 2753.17 | 2798.87 | 64.63 | 0.02 |
| DYN1 (Phospho-Ser774) | 418 | 195 | 140.5 | 128 | 134.25 | 399.41 | 204.52 | 301.96 | 137.80 | 0.46 |
| E2F1 (Ab-433) | 868 | 918 | 140.5 | 128 | 134.25 | 829.39 | 962.82 | 896.11 | 94.35 | 0.11 |
| E2F1 (Phospho-Thr433) | 124 | 102 | 140.5 | 128 | 134.25 | 118.48 | 106.98 | 112.73 | 8.13 | 0.07 |
| EEF2 (Ab-56) | 479 | 417 | 140.5 | 128 | 134.25 | 457.69 | 437.36 | 447.53 | 14.38 | 0.03 |
| EEF2 (Phospho-Thr56) | 176 | 142 | 140.5 | 128 | 134.25 | 168.17 | 148.93 | 158.55 | 13.60 | 0.09 |
| eEF2K (Ab-366) | 353 | 296 | 140.5 | 128 | 134.25 | 337.30 | 310.45 | 323.88 | 18.98 | 0.06 |
| eEF2K (Phospho-Ser366) | 230 | 178 | 140.5 | 128 | 134.25 | 219.77 | 186.69 | 203.23 | 23.39 | 0.12 |
| EGFR (Ab-1016) | 185 | 167 | 140.5 | 128 | 134.25 | 176.77 | 175.15 | 175.96 | 1.14 | 0.01 |
| EGFR (Ab-1069) | 71 | 66 | 140.5 | 128 | 134.25 | 67.84 | 69.22 | 68.53 | 0.98 | 0.01 |
| EGFR (Ab-1070) | 859 | 837 | 140.5 | 128 | 134.25 | 820.79 | 877.87 | 849.33 | 40.36 | 0.05 |
| EGFR (Ab-1092) | 204 | 189 | 140.5 | 128 | 134.25 | 194.93 | 198.23 | 196.58 | 2.34 | 0.01 |
| EGFR (Ab-1110) | 165 | 158 | 140.5 | 128 | 134.25 | 157.66 | 165.71 | 161.69 | 5.70 | 0.04 |
| EGFR (Ab-1172) | 956 | 839 | 140.5 | 128 | 134.25 | 913.47 | 879.97 | 896.72 | 23.69 | 0.03 |
| EGFR (Ab-1197) | 257 | 124 | 140.5 | 128 | 134.25 | 245.57 | 130.05 | 187.81 | 81.68 | 0.43 |
| EGFR (Ab-678) | 96 | 93 | 140.5 | 128 | 134.25 | 91.73 | 97.54 | 94.64 | 4.11 | 0.04 |
| EGFR (Ab-693) | 466 | 414 | 140.5 | 128 | 134.25 | 445.27 | 434.21 | 439.74 | 7.82 | 0.02 |
| EGFR (Ab-869) | 1595 | 1642 | 140.5 | 128 | 134.25 | 1524.05 | 1722.18 | 1623.11 | 140.10 | 0.09 |
| EGFR (Ab-998) | 75 | 128 | 140.5 | 128 | 134.25 | 71.66 | 134.25 | 102.96 | 44.26 | 0.43 |
| EGFR (Phospho-Ser1070) | 114 | 102 | 140.5 | 128 | 134.25 | 108.93 | 106.98 | 107.95 | 1.38 | 0.01 |
| EGFR (Phospho-Thr678) | 108 | 95 | 140.5 | 128 | 134.25 | 103.20 | 99.64 | 101.42 | 2.52 | 0.02 |
| EGFR (Phospho-Thr693) | 85 | 79 | 140.5 | 128 | 134.25 | 81.22 | 82.86 | 82.04 | 1.16 | 0.01 |
| EGFR (Phospho-Tyr1016) | 206 | 121 | 140.5 | 128 | 134.25 | 196.84 | 126.91 | 161.87 | 49.45 | 0.31 |
| EGFR (Phospho-Tyr1069) | 183 | 98 | 140.5 | 128 | 134.25 | 174.86 | 102.79 | 138.82 | 50.96 | 0.37 |
| EGFR (Phospho-Tyr1092) | 113 | 115 | 140.5 | 128 | 134.25 | 107.97 | 120.62 | 114.29 | 8.94 | 0.08 |
| EGFR (Phospho-Tyr1110) | 113 | 172 | 140.5 | 128 | 134.25 | 107.97 | 180.40 | 144.19 | 51.21 | 0.36 |
| EGFR (Phospho-Tyr1172) | 99 | 84 | 140.5 | 128 | 134.25 | 94.60 | 88.10 | 91.35 | 4.59 | 0.05 |
| EGFR (Phospho-Tyr1197) | 486 | 136 | 140.5 | 128 | 134.25 | 464.38 | 142.64 | 303.51 | 227.50 | 0.75 |
| EGFR (Phospho-Tyr869) | 334 | 149 | 140.5 | 128 | 134.25 | 319.14 | 156.28 | 237.71 | 115.16 | 0.48 |
| eIF2A (Ab-51) | 277 | 269 | 140.5 | 128 | 134.25 | 264.68 | 282.13 | 273.41 | 12.34 | 0.05 |
| eIF2A (Phospho-Ser51) | 240 | 240 | 140.5 | 128 | 134.25 | 229.32 | 251.72 | 240.52 | 15.84 | 0.07 |
| eIF4B (Phospho-Ser422) | 77 | 190 | 140.5 | 128 | 134.25 | 73.57 | 199.28 | 136.43 | 88.89 | 0.65 |
| eIF4E (Ab-209) | 107 | 89 | 140.5 | 128 | 134.25 | 102.24 | 93.35 | 97.79 | 6.29 | 0.06 |
| eIF4E (Phospho-Ser209) | 162 | 211 | 140.5 | 128 | 134.25 | 154.79 | 221.30 | 188.05 | 47.03 | 0.25 |
| eIF4G (Ab-1108) | 529 | 532 | 140.5 | 128 | 134.25 | 505.47 | 557.98 | 531.72 | 37.13 | 0.07 |
| eIF4G (Phospho-Ser1108) | 93 | 82 | 140.5 | 128 | 134.25 | 88.86 | 86.00 | 87.43 | 2.02 | 0.02 |
| Elk1 (Ab-383) | 109 | 119 | 140.5 | 128 | 134.25 | 104.15 | 124.81 | 114.48 | 14.61 | 0.13 |
| Elk1 (Ab-389) | 223 | 184 | 140.5 | 128 | 134.25 | 213.08 | 192.98 | 203.03 | 14.21 | 0.07 |
| Elk1 (Ab-417) | 136 | 111 | 140.5 | 128 | 134.25 | 129.95 | 116.42 | 123.19 | 9.57 | 0.08 |
| Elk1 (Phospho-Ser383) | 807 | 871 | 140.5 | 128 | 134.25 | 771.10 | 913.53 | 842.32 | 100.71 | 0.12 |
| Elk1 (Phospho-Ser389) | 92 | 95 | 140.5 | 128 | 134.25 | 87.91 | 99.64 | 93.77 | 8.30 | 0.09 |
| Elk1 (Phospho-Thr417) | 156 | 595 | 140.5 | 128 | 134.25 | 149.06 | 624.05 | 386.56 | 335.87 | 0.87 |
| eNOS (Ab-1177) | 278 | 188 | 140.5 | 128 | 134.25 | 265.63 | 197.18 | 231.41 | 48.40 | 0.21 |
| eNOS (Ab-1179) | 291 | 156 | 140.5 | 128 | 134.25 | 278.06 | 163.62 | 220.84 | 80.92 | 0.37 |
| eNOS (Ab-495) | 88 | 75 | 140.5 | 128 | 134.25 | 84.09 | 78.66 | 81.37 | 3.83 | 0.05 |
| eNOS (Ab-615) | 120 | 109 | 140.5 | 128 | 134.25 | 114.66 | 114.32 | 114.49 | 0.24 | 0.00 |
| eNOS (Phospho-Ser1177) | 135 | 333 | 140.5 | 128 | 134.25 | 128.99 | 349.26 | 239.13 | 155.75 | 0.65 |
| eNOS (Phospho-Ser615) | 161 | 151 | 140.5 | 128 | 134.25 | 153.84 | 158.37 | 156.11 | 3.21 | 0.02 |
| eNOS (Phospho-Thr495) | 156 | 189 | 140.5 | 128 | 134.25 | 149.06 | 198.23 | 173.64 | 34.77 | 0.20 |
| EPB41 (Ab-418/660) | 203 | 202 | 140.5 | 128 | 134.25 | 193.97 | 211.86 | 202.92 | 12.65 | 0.06 |
| EPB41 (Phospho-Tyr418/660) | 113 | 63 | 140.5 | 128 | 134.25 | 107.97 | 66.08 | 87.02 | 29.63 | 0.34 |
| EPHA2/3/4 (Ab-588/596) | 122 | 122 | 140.5 | 128 | 134.25 | 116.57 | 127.96 | 122.26 | 8.05 | 0.07 |
| EPHA2/3/4 (Phospho-Tyr588/596) | 126 | 79 | 140.5 | 128 | 134.25 | 120.40 | 82.86 | 101.63 | 26.54 | 0.26 |
| EPHB1/2 (Ab-594/604) | 81 | 83 | 140.5 | 128 | 134.25 | 77.40 | 87.05 | 82.22 | 6.83 | 0.08 |
| EPHB1/2 (Phospho-Tyr594/604) | 95 | 73 | 140.5 | 128 | 134.25 | 90.77 | 76.56 | 83.67 | 10.05 | 0.12 |
| Ephrin B1 (Ab-317) | 87 | 87 | 140.5 | 128 | 134.25 | 83.13 | 91.25 | 87.19 | 5.74 | 0.07 |
| Ephrin B1 (Phospho-Tyr317) | 93 | 79 | 140.5 | 128 | 134.25 | 88.86 | 82.86 | 85.86 | 4.25 | 0.05 |
| Ephrin B1/B2/B3 (Phospho-Tyr324) | 78 | 71 | 140.5 | 128 | 134.25 | 74.53 | 74.47 | 74.50 | 0.04 | 0.00 |
| Ephrin B2 (Ab-330) | 334 | 335 | 140.5 | 128 | 134.25 | 319.14 | 351.36 | 335.25 | 22.78 | 0.07 |
| Ephrin B2 (Phospho-Tyr330) | 89 | 80 | 140.5 | 128 | 134.25 | 85.04 | 83.91 | 84.47 | 0.80 | 0.01 |
| Epo-R (Ab-368) | 164 | 132 | 140.5 | 128 | 134.25 | 156.70 | 138.45 | 147.57 | 12.91 | 0.09 |
| Epo-R (Phospho-Tyr368) | 85 | 90 | 140.5 | 128 | 134.25 | 81.22 | 94.39 | 87.81 | 9.32 | 0.11 |
| ERK3 (Ab-189) | 73 | 68 | 140.5 | 128 | 134.25 | 69.75 | 71.32 | 70.54 | 1.11 | 0.02 |
| ERK3 (Phospho-Ser189) | 71 | 60 | 140.5 | 128 | 134.25 | 67.84 | 62.93 | 65.39 | 3.47 | 0.05 |
| ERK8 (Phospho-Thr175/Tyr177) | 74 | 74 | 140.5 | 128 | 134.25 | 70.71 | 77.61 | 74.16 | 4.88 | 0.07 |
| Estrogen Receptor-alpha (Ab-104) | 369 | 253 | 140.5 | 128 | 134.25 | 352.59 | 265.35 | 308.97 | 61.68 | 0.20 |
| Estrogen Receptor-alpha (Ab-106) | 2346 | 2800 | 140.5 | 128 | 134.25 | 2241.64 | 2936.72 | 2589.18 | 491.49 | 0.19 |
| Estrogen Receptor-alpha (Ab-118) | 111 | 93 | 140.5 | 128 | 134.25 | 106.06 | 97.54 | 101.80 | 6.03 | 0.06 |
| Estrogen Receptor-alpha (Ab-167) | 167 | 122 | 140.5 | 128 | 134.25 | 159.57 | 127.96 | 143.76 | 22.35 | 0.16 |
| Estrogen Receptor-alpha (Phospho-Ser104) | 128 | 111 | 140.5 | 128 | 134.25 | 122.31 | 116.42 | 119.36 | 4.16 | 0.03 |
| Estrogen Receptor-alpha (Phospho-Ser106) | 252 | 118 | 140.5 | 128 | 134.25 | 240.79 | 123.76 | 182.28 | 82.75 | 0.45 |
| Estrogen Receptor-alpha (Phospho-Ser118) | 207 | 157 | 140.5 | 128 | 134.25 | 197.79 | 164.67 | 181.23 | 23.42 | 0.13 |
| Estrogen Receptor-alpha (Phospho-Ser167) | 161 | 208 | 140.5 | 128 | 134.25 | 153.84 | 218.16 | 186.00 | 45.48 | 0.24 |
| ETK (Ab-40) | 146 | 125 | 140.5 | 128 | 134.25 | 139.51 | 131.10 | 135.30 | 5.94 | 0.04 |
| ETK (Ab-566) | 88 | 99 | 140.5 | 128 | 134.25 | 84.09 | 103.83 | 93.96 | 13.96 | 0.15 |
| ETK (Phospho-Tyr40) | 89 | 65 | 140.5 | 128 | 134.25 | 85.04 | 68.17 | 76.61 | 11.93 | 0.16 |
| Ezrin (Ab-353) | 159 | 131 | 140.5 | 128 | 134.25 | 151.93 | 137.40 | 144.66 | 10.27 | 0.07 |
| Ezrin (Ab-478) | 92 | 87 | 140.5 | 128 | 134.25 | 87.91 | 91.25 | 89.58 | 2.36 | 0.03 |
| Ezrin (Ab-566) | 1011 | 773 | 140.5 | 128 | 134.25 | 966.03 | 810.74 | 888.39 | 109.80 | 0.12 |
| Ezrin (Phospho-Thr566) | 477 | 115 | 140.5 | 128 | 134.25 | 455.78 | 120.62 | 288.20 | 237.00 | 0.82 |
| Ezrin (Phospho-Tyr353) | 475 | 105 | 140.5 | 128 | 134.25 | 453.87 | 110.13 | 282.00 | 243.06 | 0.86 |
| Ezrin (Phospho-Tyr478) | 80 | 301 | 140.5 | 128 | 134.25 | 76.44 | 315.70 | 196.07 | 169.18 | 0.86 |
| FADD (Phospho-Ser194) | 483 | 494 | 140.5 | 128 | 134.25 | 461.51 | 518.12 | 489.82 | 40.03 | 0.08 |
| FAK (Ab-397) | 347 | 217 | 140.5 | 128 | 134.25 | 331.56 | 227.60 | 279.58 | 73.52 | 0.26 |
| FAK (Ab-407) | 83 | 74 | 140.5 | 128 | 134.25 | 79.31 | 77.61 | 78.46 | 1.20 | 0.02 |
| FAK (Ab-576) | 98 | 84 | 140.5 | 128 | 134.25 | 93.64 | 88.10 | 90.87 | 3.92 | 0.04 |
| FAK (Ab-861) | 114 | 123 | 140.5 | 128 | 134.25 | 108.93 | 129.01 | 118.97 | 14.20 | 0.12 |
| FAK (Ab-910) | 2118 | 2539 | 140.5 | 128 | 134.25 | 2023.78 | 2662.97 | 2343.38 | 451.98 | 0.19 |
| FAK (Ab-925) | 117 | 99 | 140.5 | 128 | 134.25 | 111.80 | 103.83 | 107.81 | 5.63 | 0.05 |
| FAK (Phospho-Ser910) | 523 | 427 | 140.5 | 128 | 134.25 | 499.73 | 447.85 | 473.79 | 36.69 | 0.08 |
| FAK (Phospho-Tyr397) | 296 | 260 | 140.5 | 128 | 134.25 | 282.83 | 272.70 | 277.76 | 7.17 | 0.03 |
| FAK (Phospho-Tyr407) | 72 | 68 | 140.5 | 128 | 134.25 | 68.80 | 71.32 | 70.06 | 1.78 | 0.03 |
| FAK (Phospho-Tyr576) | 125 | 113 | 140.5 | 128 | 134.25 | 119.44 | 118.52 | 118.98 | 0.65 | 0.01 |
| FAK (Phospho-Tyr861) | 106 | 309 | 140.5 | 128 | 134.25 | 101.28 | 324.09 | 212.69 | 157.55 | 0.74 |
| FAK (Phospho-Tyr925) | 245 | 104 | 140.5 | 128 | 134.25 | 234.10 | 109.08 | 171.59 | 88.40 | 0.52 |
| FAS (Ab-291) | 282 | 235 | 140.5 | 128 | 134.25 | 269.46 | 246.47 | 257.97 | 16.25 | 0.06 |
| FAS (Phospho-Tyr291) | 153 | 113 | 140.5 | 128 | 134.25 | 146.19 | 118.52 | 132.36 | 19.57 | 0.15 |
| FER (Ab-402) | 336 | 296 | 140.5 | 128 | 134.25 | 321.05 | 310.45 | 315.75 | 7.50 | 0.02 |
| FER (Phospho-Tyr402) | 738 | 820 | 140.5 | 128 | 134.25 | 705.17 | 860.04 | 782.60 | 109.51 | 0.14 |
| FGFR1 (Ab-154) | 117 | 116 | 140.5 | 128 | 134.25 | 111.80 | 121.66 | 116.73 | 6.98 | 0.06 |
| FGFR1 (Ab-766) | 152 | 121 | 140.5 | 128 | 134.25 | 145.24 | 126.91 | 136.07 | 12.96 | 0.10 |
| FGFR1 (Phospho-Tyr154) | 130 | 265 | 140.5 | 128 | 134.25 | 124.22 | 277.94 | 201.08 | 108.70 | 0.54 |
| FGFR1 (Phospho-Tyr654) | 577 | 592 | 140.5 | 128 | 134.25 | 551.33 | 620.91 | 586.12 | 49.20 | 0.08 |
| FGFR1 (Phospho-Tyr766) | 96 | 219 | 140.5 | 128 | 134.25 | 91.73 | 229.69 | 160.71 | 97.56 | 0.61 |
| Filamin A (Ab-2152) | 262 | 226 | 140.5 | 128 | 134.25 | 250.35 | 237.04 | 243.69 | 9.41 | 0.04 |
| Filamin A (Phospho-Ser2152) | 165 | 132 | 140.5 | 128 | 134.25 | 157.66 | 138.45 | 148.05 | 13.59 | 0.09 |
| FKHR (Ab-256) | 218 | 96 | 140.5 | 128 | 134.25 | 208.30 | 100.69 | 154.49 | 76.10 | 0.49 |
| FKHR (Ab-319) | 107 | 87 | 140.5 | 128 | 134.25 | 102.24 | 91.25 | 96.74 | 7.77 | 0.08 |
| FKHR (Phospho-Ser256) | 103 | 97 | 140.5 | 128 | 134.25 | 98.42 | 101.74 | 100.08 | 2.35 | 0.02 |
| FKHR (Phospho-Ser319) | 112 | 101 | 140.5 | 128 | 134.25 | 107.02 | 105.93 | 106.47 | 0.77 | 0.01 |
| FKHR/FOXO1A (Ab-329) | 108 | 79 | 140.5 | 128 | 134.25 | 103.20 | 82.86 | 93.03 | 14.38 | 0.15 |
| FKHR/FOXO1A (Phospho-Ser329) | 70 | 71 | 140.5 | 128 | 134.25 | 66.89 | 74.47 | 70.68 | 5.36 | 0.08 |
| FKHRL1/FOXO3A (Ab-253) | 421 | 398 | 140.5 | 128 | 134.25 | 402.27 | 417.43 | 409.85 | 10.72 | 0.03 |
| FKHRL1/FOXO3A (Phospho-Ser253) | 101 | 127 | 140.5 | 128 | 134.25 | 96.51 | 133.20 | 114.85 | 25.95 | 0.23 |
| FLT3 (Ab-599) | 92 | 82 | 140.5 | 128 | 134.25 | 87.91 | 86.00 | 86.96 | 1.35 | 0.02 |
| FLT3 (Phospho-Tyr599) | 93 | 96 | 140.5 | 128 | 134.25 | 88.86 | 100.69 | 94.78 | 8.36 | 0.09 |
| FLT3 (Phospho-Tyr842) | 561 | 87 | 140.5 | 128 | 134.25 | 536.04 | 91.25 | 313.65 | 314.52 | 1.00 |
| FLT3 (Phospho-Tyr969) | 109 | 77 | 140.5 | 128 | 134.25 | 104.15 | 80.76 | 92.46 | 16.54 | 0.18 |
| Fos (Ab-232) | 150 | 162 | 140.5 | 128 | 134.25 | 143.33 | 169.91 | 156.62 | 18.80 | 0.12 |
| Fos (Ab-374) | 264 | 255 | 140.5 | 128 | 134.25 | 252.26 | 267.45 | 259.85 | 10.74 | 0.04 |
| Fos (Phospho-Ser362) | 92 | 77 | 140.5 | 128 | 134.25 | 87.91 | 80.76 | 84.33 | 5.05 | 0.06 |
| Fos (Phospho-Thr232) | 87 | 113 | 140.5 | 128 | 134.25 | 83.13 | 118.52 | 100.82 | 25.02 | 0.25 |
| FosB (Ab-27) | 95 | 92 | 140.5 | 128 | 134.25 | 90.77 | 96.49 | 93.63 | 4.04 | 0.04 |
| FosB (Phospho-Ser27) | 226 | 164 | 140.5 | 128 | 134.25 | 215.95 | 172.01 | 193.98 | 31.07 | 0.16 |
| FOXO1/3/4-pan (Ab-24/32) | 131 | 123 | 140.5 | 128 | 134.25 | 125.17 | 129.01 | 127.09 | 2.71 | 0.02 |
| FOXO1/3/4-pan (Phospho-Thr24/32) | 71 | 274 | 140.5 | 128 | 134.25 | 67.84 | 287.38 | 177.61 | 155.24 | 0.87 |
| FOXO1A/3A (Phospho-Ser322/325) | 82 | 91 | 140.5 | 128 | 134.25 | 78.35 | 95.44 | 86.90 | 12.09 | 0.14 |
| FRS2 (Phospho-Tyr436) | 85 | 80 | 140.5 | 128 | 134.25 | 81.22 | 83.91 | 82.56 | 1.90 | 0.02 |
| Fyn (Phospho-Tyr530) | 72 | 65 | 140.5 | 128 | 134.25 | 68.80 | 68.17 | 68.49 | 0.44 | 0.01 |
| G3BP-1 (Ab-232) | 259 | 241 | 140.5 | 128 | 134.25 | 247.48 | 252.77 | 250.12 | 3.74 | 0.01 |
| G3BP-1 (Phospho-Ser232) | 206 | 184 | 140.5 | 128 | 134.25 | 196.84 | 192.98 | 194.91 | 2.72 | 0.01 |
| Gab1 (Ab-627) | 181 | 152 | 140.5 | 128 | 134.25 | 172.95 | 159.42 | 166.19 | 9.56 | 0.06 |
| Gab1 (Ab-659) | 89 | 103 | 140.5 | 128 | 134.25 | 85.04 | 108.03 | 96.54 | 16.26 | 0.17 |
| Gab1 (Phospho-Tyr627) | 97 | 345 | 140.5 | 128 | 134.25 | 92.69 | 361.85 | 227.27 | 190.33 | 0.84 |
| Gab1 (Phospho-Tyr659) | 108 | 90 | 140.5 | 128 | 134.25 | 103.20 | 94.39 | 98.80 | 6.22 | 0.06 |
| Gab2 (Ab-159) | 76 | 69 | 140.5 | 128 | 134.25 | 72.62 | 72.37 | 72.49 | 0.18 | 0.00 |
| Gab2 (Ab-623) | 116 | 122 | 140.5 | 128 | 134.25 | 110.84 | 127.96 | 119.40 | 12.10 | 0.10 |
| Gab2 (Phospho-Ser159) | 98 | 91 | 140.5 | 128 | 134.25 | 93.64 | 95.44 | 94.54 | 1.27 | 0.01 |
| Gab2 (Phospho-Tyr643) | 1430 | 1333 | 140.5 | 128 | 134.25 | 1366.39 | 1398.09 | 1382.24 | 22.42 | 0.02 |
| GABA-RB (Ab-434) | 85 | 81 | 140.5 | 128 | 134.25 | 81.22 | 84.96 | 83.09 | 2.64 | 0.03 |
| GABA-RB (Phospho-Ser434) | 84 | 225 | 140.5 | 128 | 134.25 | 80.26 | 235.99 | 158.12 | 110.11 | 0.70 |
| GAP43 (Ab-41) | 195 | 199 | 140.5 | 128 | 134.25 | 186.33 | 208.72 | 197.52 | 15.83 | 0.08 |
| GAP43 (Phospho-Ser41) | 238 | 205 | 140.5 | 128 | 134.25 | 227.41 | 215.01 | 221.21 | 8.77 | 0.04 |
| GAPDH | 253 | 249 | 140.5 | 128 | 134.25 | 241.75 | 261.16 | 251.45 | 13.73 | 0.05 |
| GATA1 (Ab-142) | 120 | 107 | 140.5 | 128 | 134.25 | 114.66 | 112.22 | 113.44 | 1.72 | 0.02 |
| GATA1 (Ab-310) | 200 | 188 | 140.5 | 128 | 134.25 | 191.10 | 197.18 | 194.14 | 4.30 | 0.02 |
| GATA1 (Phospho-Ser142) | 384 | 144 | 140.5 | 128 | 134.25 | 366.92 | 151.03 | 258.97 | 152.66 | 0.59 |
| GATA1 (Phospho-Ser310) | 139 | 402 | 140.5 | 128 | 134.25 | 132.82 | 421.63 | 277.22 | 204.22 | 0.74 |
| GluR1 (Ab-849) | 3672 | 4066 | 140.5 | 128 | 134.25 | 3508.65 | 4264.54 | 3886.59 | 534.49 | 0.14 |
| GluR1 (Ab-863) | 95 | 83 | 140.5 | 128 | 134.25 | 90.77 | 87.05 | 88.91 | 2.63 | 0.03 |
| GluR1 (Phospho-Ser849) | 138 | 161 | 140.5 | 128 | 134.25 | 131.86 | 168.86 | 150.36 | 26.16 | 0.17 |
| GluR1 (Phospho-Ser863) | 201 | 147 | 140.5 | 128 | 134.25 | 192.06 | 154.18 | 173.12 | 26.79 | 0.15 |
| GluR2 (Ab-880) | 1975 | 1861 | 140.5 | 128 | 134.25 | 1887.14 | 1951.87 | 1919.51 | 45.77 | 0.02 |
| GluR2 (Phospho-Ser880) | 122 | 121 | 140.5 | 128 | 134.25 | 116.57 | 126.91 | 121.74 | 7.31 | 0.06 |
| GRB10/Growth factor receptor-bound protein 10 (Ab-67) | 97 | 84 | 140.5 | 128 | 134.25 | 92.69 | 88.10 | 90.39 | 3.24 | 0.04 |
| GRB10/Growth factor receptor-bound protein 10 (Phospho-Tyr67) | 78 | 74 | 140.5 | 128 | 134.25 | 74.53 | 77.61 | 76.07 | 2.18 | 0.03 |
| GRF-1 (Phospho-Tyr1105) | 291 | 93 | 140.5 | 128 | 134.25 | 278.06 | 97.54 | 187.80 | 127.64 | 0.68 |
| GRK1 (Ab-21) | 100 | 91 | 140.5 | 128 | 134.25 | 95.55 | 95.44 | 95.50 | 0.08 | 0.00 |
| GRK1 (Phospho-Ser21) | 107 | 98 | 140.5 | 128 | 134.25 | 102.24 | 102.79 | 102.51 | 0.39 | 0.00 |
| GRK2 (Ab-29) | 763 | 738 | 140.5 | 128 | 134.25 | 729.06 | 774.04 | 751.55 | 31.80 | 0.04 |
| GRK2 (Phospho-Ser29) | 191 | 256 | 140.5 | 128 | 134.25 | 182.50 | 268.50 | 225.50 | 60.81 | 0.27 |
| GRK2 (Phospho-Ser685) | 91 | 74 | 140.5 | 128 | 134.25 | 86.95 | 77.61 | 82.28 | 6.60 | 0.08 |
| GSK3 alpha (Ab-21) | 203 | 231 | 140.5 | 128 | 134.25 | 193.97 | 242.28 | 218.12 | 34.16 | 0.16 |
| GSK3 alpha (Phospho-Ser21) | 485 | 223 | 140.5 | 128 | 134.25 | 463.43 | 233.89 | 348.66 | 162.31 | 0.47 |
| GSK3 alpha/beta (Ab-216/279) | 92 | 77 | 140.5 | 128 | 134.25 | 87.91 | 80.76 | 84.33 | 5.05 | 0.06 |
| GSK3 alpha/beta (Phospho-Tyr216/279) | 139 | 174 | 140.5 | 128 | 134.25 | 132.82 | 182.50 | 157.66 | 35.13 | 0.22 |
| GSK3 beta (Ab-9) | 136 | 125 | 140.5 | 128 | 134.25 | 129.95 | 131.10 | 130.53 | 0.82 | 0.01 |
| GSK3 beta (Phospho-Ser9) | 185 | 354 | 140.5 | 128 | 134.25 | 176.77 | 371.29 | 274.03 | 137.54 | 0.50 |
| GTPase activating protein (Ab-387) | 96 | 86 | 140.5 | 128 | 134.25 | 91.73 | 90.20 | 90.96 | 1.08 | 0.01 |
| GTPase activating protein (Phospho-Ser387) | 71 | 62 | 140.5 | 128 | 134.25 | 67.84 | 65.03 | 66.43 | 1.99 | 0.03 |
| HCK (Phospho-Tyr410) | 194 | 630 | 140.5 | 128 | 134.25 | 185.37 | 660.76 | 423.07 | 336.15 | 0.79 |
| HDAC1 (Ab-421) | 4892 | 6089 | 140.5 | 128 | 134.25 | 4674.38 | 6386.31 | 5530.35 | 1210.52 | 0.22 |
| HDAC1 (Phospho-Ser421) | 434 | 368 | 140.5 | 128 | 134.25 | 414.69 | 385.97 | 400.33 | 20.31 | 0.05 |
| HDAC2 (Ab-394) | 254 | 328 | 140.5 | 128 | 134.25 | 242.70 | 344.02 | 293.36 | 71.64 | 0.24 |
| HDAC2 (Phospho-Ser394) | 243 | 261 | 140.5 | 128 | 134.25 | 232.19 | 273.74 | 252.97 | 29.38 | 0.12 |
| HDAC3 (Ab-424) | 544 | 490 | 140.5 | 128 | 134.25 | 519.80 | 513.93 | 516.86 | 4.15 | 0.01 |
| HDAC3 (Phospho-Ser424) | 96 | 97 | 140.5 | 128 | 134.25 | 91.73 | 101.74 | 96.73 | 7.08 | 0.07 |
| HDAC4 (Ab-632) | 87 | 71 | 140.5 | 128 | 134.25 | 83.13 | 74.47 | 78.80 | 6.13 | 0.08 |
| HDAC4 (Phospho-Ser632) | 228 | 209 | 140.5 | 128 | 134.25 | 217.86 | 219.21 | 218.53 | 0.95 | 0.00 |
| HDAC5 (Ab-259) | 121 | 111 | 140.5 | 128 | 134.25 | 115.62 | 116.42 | 116.02 | 0.57 | 0.00 |
| HDAC5 (Ab-498) | 151 | 136 | 140.5 | 128 | 134.25 | 144.28 | 142.64 | 143.46 | 1.16 | 0.01 |
| HDAC5 (Phospho-Ser259) | 105 | 171 | 140.5 | 128 | 134.25 | 100.33 | 179.35 | 139.84 | 55.88 | 0.40 |
| HDAC5 (Phospho-Ser498) | 207 | 361 | 140.5 | 128 | 134.25 | 197.79 | 378.63 | 288.21 | 127.87 | 0.44 |
| HDAC6 (Ab-22) | 926 | 876 | 140.5 | 128 | 134.25 | 884.81 | 918.77 | 901.79 | 24.02 | 0.03 |
| HDAC6 (Phospho-Ser22) | 153 | 115 | 140.5 | 128 | 134.25 | 146.19 | 120.62 | 133.40 | 18.09 | 0.14 |
| HDAC8 (Ab-39) | 270 | 210 | 140.5 | 128 | 134.25 | 257.99 | 220.25 | 239.12 | 26.68 | 0.11 |
| HDAC8 (Phospho-Ser39) | 200 | 213 | 140.5 | 128 | 134.25 | 191.10 | 223.40 | 207.25 | 22.84 | 0.11 |
| HER2 (Ab-1112) | 151 | 157 | 140.5 | 128 | 134.25 | 144.28 | 164.67 | 154.47 | 14.41 | 0.09 |
| HER2 (Ab-1221/1222) | 557 | 554 | 140.5 | 128 | 134.25 | 532.22 | 581.05 | 556.64 | 34.53 | 0.06 |
| HER2 (Ab-1248) | 210 | 205 | 140.5 | 128 | 134.25 | 200.66 | 215.01 | 207.83 | 10.15 | 0.05 |
| HER2 (Ab-686) | 108 | 103 | 140.5 | 128 | 134.25 | 103.20 | 108.03 | 105.61 | 3.42 | 0.03 |
| HER2 (Ab-877) | 118 | 94 | 140.5 | 128 | 134.25 | 112.75 | 98.59 | 105.67 | 10.01 | 0.09 |
| HER2 (Phospho-Thr686) | 246 | 190 | 140.5 | 128 | 134.25 | 235.06 | 199.28 | 217.17 | 25.30 | 0.12 |
| HER2 (Phospho-Tyr1221/Tyr1222) | 142 | 257 | 140.5 | 128 | 134.25 | 135.68 | 269.55 | 202.62 | 94.66 | 0.47 |
| HER2 (Phospho-Tyr1248) | 170 | 150 | 140.5 | 128 | 134.25 | 162.44 | 157.32 | 159.88 | 3.62 | 0.02 |
| HER2 (Phospho-Tyr877) | 126 | 216 | 140.5 | 128 | 134.25 | 120.40 | 226.55 | 173.47 | 75.06 | 0.43 |
| HER3/ErbB3 (Ab-1222) | 108 | 101 | 140.5 | 128 | 134.25 | 103.20 | 105.93 | 104.56 | 1.93 | 0.02 |
| HER3/ErbB3 (Ab-1289) | 119 | 111 | 140.5 | 128 | 134.25 | 113.71 | 116.42 | 115.06 | 1.92 | 0.02 |
| HER3/ErbB3 (Phospho-Tyr1222) | 98 | 74 | 140.5 | 128 | 134.25 | 93.64 | 77.61 | 85.63 | 11.33 | 0.13 |
| HER3/ErbB3 (Phospho-Tyr1289) | 100 | 71 | 140.5 | 128 | 134.25 | 95.55 | 74.47 | 85.01 | 14.91 | 0.18 |
| HER4/ErbB4 (Ab-1284) | 587 | 619 | 140.5 | 128 | 134.25 | 560.89 | 649.22 | 605.06 | 62.46 | 0.10 |
| HER4/ErbB4 (Phospho-Tyr1284) | 591 | 515 | 140.5 | 128 | 134.25 | 564.71 | 540.15 | 552.43 | 17.37 | 0.03 |
| Histone H2A.X (Ab-139) | 594 | 541 | 140.5 | 128 | 134.25 | 567.58 | 567.42 | 567.50 | 0.11 | 0.00 |
| Histone H2A.X (Phospho-Ser139) | 397 | 377 | 140.5 | 128 | 134.25 | 379.34 | 395.41 | 387.37 | 11.36 | 0.03 |
| Histone H3.1 (Ab-10) | 233 | 199 | 140.5 | 128 | 134.25 | 222.64 | 208.72 | 215.68 | 9.84 | 0.05 |
| Histone H3.1 (Phospho-Ser10) | 146 | 127 | 140.5 | 128 | 134.25 | 139.51 | 133.20 | 136.35 | 4.46 | 0.03 |
| HNF4 alpha (Ab-313) | 105 | 86 | 140.5 | 128 | 134.25 | 100.33 | 90.20 | 95.26 | 7.16 | 0.08 |
| HNF4 alpha (Phospho-Ser313) | 102 | 140 | 140.5 | 128 | 134.25 | 97.46 | 146.84 | 122.15 | 34.91 | 0.29 |
| HRS (Ab-334) | 82 | 79 | 140.5 | 128 | 134.25 | 78.35 | 82.86 | 80.60 | 3.19 | 0.04 |
| HRS (Phospho-Tyr334) | 102 | 95 | 140.5 | 128 | 134.25 | 97.46 | 99.64 | 98.55 | 1.54 | 0.02 |
| HSF1 (Ab-303) | 316 | 120 | 140.5 | 128 | 134.25 | 301.94 | 125.86 | 213.90 | 124.51 | 0.58 |
| HSF1 (Phospho-Ser303) | 114 | 114 | 140.5 | 128 | 134.25 | 108.93 | 119.57 | 114.25 | 7.52 | 0.07 |
| HSL (Ab-552/563) | 275 | 177 | 140.5 | 128 | 134.25 | 262.77 | 185.64 | 224.20 | 54.54 | 0.24 |
| HSL (Ab-554) | 603 | 88 | 140.5 | 128 | 134.25 | 576.18 | 92.30 | 334.24 | 342.15 | 1.02 |
| HSL (Phospho-Ser552/563) | 112 | 97 | 140.5 | 128 | 134.25 | 107.02 | 101.74 | 104.38 | 3.73 | 0.04 |
| HSL (Phospho-Ser554) | 195 | 230 | 140.5 | 128 | 134.25 | 186.33 | 241.23 | 213.78 | 38.82 | 0.18 |
| HSP27 (Ab-15) | 168 | 143 | 140.5 | 128 | 134.25 | 160.53 | 149.98 | 155.25 | 7.46 | 0.05 |
| HSP27 (Ab-78) | 73 | 75 | 140.5 | 128 | 134.25 | 69.75 | 78.66 | 74.21 | 6.30 | 0.08 |
| HSP27 (Ab-82) | 213 | 91 | 140.5 | 128 | 134.25 | 203.52 | 95.44 | 149.48 | 76.43 | 0.51 |
| HSP27 (Phospho-Ser15) | 261 | 447 | 140.5 | 128 | 134.25 | 249.39 | 468.83 | 359.11 | 155.17 | 0.43 |
| HSP27 (Phospho-Ser78) | 96 | 91 | 140.5 | 128 | 134.25 | 91.73 | 95.44 | 93.59 | 2.63 | 0.03 |
| HSP27 (Phospho-Ser82) | 321 | 225 | 140.5 | 128 | 134.25 | 306.72 | 235.99 | 271.35 | 50.02 | 0.18 |
| HSP90 co-chaperone Cdc37 (Ab-13) | 92 | 84 | 140.5 | 128 | 134.25 | 87.91 | 88.10 | 88.00 | 0.14 | 0.00 |
| HSP90 co-chaperone Cdc37 (Phospho-Ser13) | 184 | 82 | 140.5 | 128 | 134.25 | 175.81 | 86.00 | 130.91 | 63.51 | 0.49 |
| HSP90B (Ab-226) | 83 | 76 | 140.5 | 128 | 134.25 | 79.31 | 79.71 | 79.51 | 0.29 | 0.00 |
| HSP90B (Ab-254) | 122 | 97 | 140.5 | 128 | 134.25 | 116.57 | 101.74 | 109.15 | 10.49 | 0.10 |
| HSP90B (Phospho-Ser226) | 230 | 238 | 140.5 | 128 | 134.25 | 219.77 | 249.62 | 234.69 | 21.11 | 0.09 |
| HSP90B (Phospho-Ser254) | 93 | 79 | 140.5 | 128 | 134.25 | 88.86 | 82.86 | 85.86 | 4.25 | 0.05 |
| ICAM-1 (Ab-512) | 89 | 79 | 140.5 | 128 | 134.25 | 85.04 | 82.86 | 83.95 | 1.54 | 0.02 |
| ICAM-1 (Phospho-Tyr512) | 127 | 111 | 140.5 | 128 | 134.25 | 121.35 | 116.42 | 118.89 | 3.49 | 0.03 |
| ICK (Phospho-Tyr159) | 138 | 99 | 140.5 | 128 | 134.25 | 131.86 | 103.83 | 117.85 | 19.82 | 0.17 |
| IGF1R (Ab-1161) | 101 | 100 | 140.5 | 128 | 134.25 | 96.51 | 104.88 | 100.69 | 5.92 | 0.06 |
| IGF1R (Ab-1165/1166) | 96 | 95 | 140.5 | 128 | 134.25 | 91.73 | 99.64 | 95.68 | 5.59 | 0.06 |
| IGF1R (Phospho-Tyr1161) | 238 | 193 | 140.5 | 128 | 134.25 | 227.41 | 202.42 | 214.92 | 17.67 | 0.08 |
| IGF1R (Phospho-Tyr1165/1166) | 178 | 234 | 140.5 | 128 | 134.25 | 170.08 | 245.43 | 207.75 | 53.28 | 0.26 |
| IGF2R (Ab-2409) | 104 | 91 | 140.5 | 128 | 134.25 | 99.37 | 95.44 | 97.41 | 2.78 | 0.03 |
| IGF2R (Phospho-Ser2409) | 93 | 73 | 140.5 | 128 | 134.25 | 88.86 | 76.56 | 82.71 | 8.70 | 0.11 |
| IGFBP-3 (Ab-183) | 126 | 113 | 140.5 | 128 | 134.25 | 120.40 | 118.52 | 119.46 | 1.33 | 0.01 |
| IkB-alpha (Ab-32/36) | 217 | 140 | 140.5 | 128 | 134.25 | 207.35 | 146.84 | 177.09 | 42.79 | 0.24 |
| IkB-alpha (Ab-42) | 125 | 208 | 140.5 | 128 | 134.25 | 119.44 | 218.16 | 168.80 | 69.80 | 0.41 |
| IkB-alpha (Phospho-Ser32/36) | 1734 | 1822 | 140.5 | 128 | 134.25 | 1656.86 | 1910.96 | 1783.91 | 179.68 | 0.10 |
| IkB-alpha (Phospho-Tyr305) | 76 | 63 | 140.5 | 128 | 134.25 | 72.62 | 66.08 | 69.35 | 4.63 | 0.07 |
| IkB-alpha (Phospho-Tyr42) | 83 | 71 | 140.5 | 128 | 134.25 | 79.31 | 74.47 | 76.89 | 3.42 | 0.04 |
| IkB-beta (Ab-19) | 95 | 82 | 140.5 | 128 | 134.25 | 90.77 | 86.00 | 88.39 | 3.37 | 0.04 |
| IkB-beta (Phospho-Ser23) | 95 | 81 | 140.5 | 128 | 134.25 | 90.77 | 84.96 | 87.86 | 4.11 | 0.05 |
| IkB-beta (Phospho-Thr19) | 355 | 223 | 140.5 | 128 | 134.25 | 339.21 | 233.89 | 286.55 | 74.47 | 0.26 |
| IkB-epsilon (Ab-22) | 1026 | 987 | 140.5 | 128 | 134.25 | 980.36 | 1035.19 | 1007.78 | 38.77 | 0.04 |
| IkB-epsilon (Phospho-Ser22) | 127 | 112 | 140.5 | 128 | 134.25 | 121.35 | 117.47 | 119.41 | 2.74 | 0.02 |
| IKK-alpha (Ab-23) | 281 | 216 | 140.5 | 128 | 134.25 | 268.50 | 226.55 | 247.52 | 29.67 | 0.12 |
| IKK-alpha (Phospho-Thr23) | 151 | 128 | 140.5 | 128 | 134.25 | 144.28 | 134.25 | 139.27 | 7.09 | 0.05 |
| IKK-alpha/beta (Ab-176/177) | 1683 | 2370 | 140.5 | 128 | 134.25 | 1608.13 | 2485.72 | 2046.93 | 620.55 | 0.30 |
| IKK-alpha/beta (Ab-180/181) | 94 | 132 | 140.5 | 128 | 134.25 | 89.82 | 138.45 | 114.13 | 34.38 | 0.30 |
| IKK-alpha/beta (Phospho-Ser180/181) | 81 | 249 | 140.5 | 128 | 134.25 | 77.40 | 261.16 | 169.28 | 129.94 | 0.77 |
| IKK-beta (Ab-188) | 271 | 211 | 140.5 | 128 | 134.25 | 258.94 | 221.30 | 240.12 | 26.62 | 0.11 |
| IKK-beta (Ab-199) | 923 | 852 | 140.5 | 128 | 134.25 | 881.94 | 893.60 | 887.77 | 8.25 | 0.01 |
| IKK-beta (Phospho-Tyr188) | 90 | 65 | 140.5 | 128 | 134.25 | 86.00 | 68.17 | 77.09 | 12.60 | 0.16 |
| IKK-beta (Phospho-Tyr199) | 222 | 78 | 140.5 | 128 | 134.25 | 212.12 | 81.81 | 146.97 | 92.15 | 0.63 |
| IKK-gamma (Ab-31) | 1350 | 1999 | 140.5 | 128 | 134.25 | 1289.95 | 2096.61 | 1693.28 | 570.40 | 0.34 |
| IKK-gamma (Ab-85) | 173 | 151 | 140.5 | 128 | 134.25 | 165.30 | 158.37 | 161.84 | 4.90 | 0.03 |
| IKK-gamma (Phospho-Ser31) | 180 | 207 | 140.5 | 128 | 134.25 | 171.99 | 217.11 | 194.55 | 31.90 | 0.16 |
| IKK-gamma (Phospho-Ser85) | 227 | 168 | 140.5 | 128 | 134.25 | 216.90 | 176.20 | 196.55 | 28.78 | 0.15 |
| IL-10R-alpha (Ab-496) | 213 | 173 | 140.5 | 128 | 134.25 | 203.52 | 181.45 | 192.49 | 15.61 | 0.08 |
| IL-10R-alpha (Phospho-Tyr496) | 145 | 144 | 140.5 | 128 | 134.25 | 138.55 | 151.03 | 144.79 | 8.83 | 0.06 |
| IL-13R/CD213a1 (Ab-405) | 125 | 117 | 140.5 | 128 | 134.25 | 119.44 | 122.71 | 121.08 | 2.31 | 0.02 |
| IL-13R/CD213a1 (Phospho-Tyr405) | 204 | 1103 | 140.5 | 128 | 134.25 | 194.93 | 1156.86 | 675.89 | 680.19 | 1.01 |
| IL-2RA/CD25 (Ab-268) | 83 | 77 | 140.5 | 128 | 134.25 | 79.31 | 80.76 | 80.03 | 1.03 | 0.01 |
| IL-2RA/CD25 (Phospho-Ser268) | 77 | 288 | 140.5 | 128 | 134.25 | 73.57 | 302.06 | 187.82 | 161.57 | 0.86 |
| IL3RB (Ab-593) | 157 | 170 | 140.5 | 128 | 134.25 | 150.02 | 178.30 | 164.16 | 20.00 | 0.12 |
| IL3RB (Phospho-Tyr593) | 88 | 127 | 140.5 | 128 | 134.25 | 84.09 | 133.20 | 108.64 | 34.73 | 0.32 |
| IL-4R/CD124 (Ab-497) | 169 | 209 | 140.5 | 128 | 134.25 | 161.48 | 219.21 | 190.34 | 40.82 | 0.21 |
| IL-4R/CD124 (Phospho-Tyr497) | 818 | 108 | 140.5 | 128 | 134.25 | 781.61 | 113.27 | 447.44 | 472.59 | 1.06 |
| IL7R/CD127 (Phospho-Tyr449) | 258 | 548 | 140.5 | 128 | 134.25 | 246.52 | 574.76 | 410.64 | 232.10 | 0.57 |
| Integrin beta-1 (Ab-788) | 178 | 162 | 140.5 | 128 | 134.25 | 170.08 | 169.91 | 170.00 | 0.12 | 0.00 |
| Integrin beta-1 (Ab-789) | 520 | 531 | 140.5 | 128 | 134.25 | 496.87 | 556.93 | 526.90 | 42.47 | 0.08 |
| Integrin beta-1 (Phospho-Thr788) | 147 | 131 | 140.5 | 128 | 134.25 | 140.46 | 137.40 | 138.93 | 2.17 | 0.02 |
| Integrin beta-3 (Ab-773) | 149 | 139 | 140.5 | 128 | 134.25 | 142.37 | 145.79 | 144.08 | 2.41 | 0.02 |
| Integrin beta-3 (Ab-785) | 97 | 116 | 140.5 | 128 | 134.25 | 92.69 | 121.66 | 107.17 | 20.49 | 0.19 |
| Integrin beta-3 (Phospho-Tyr773) | 533 | 462 | 140.5 | 128 | 134.25 | 509.29 | 484.56 | 496.92 | 17.49 | 0.04 |
| Integrin beta-3 (Phospho-Tyr785) | 105 | 102 | 140.5 | 128 | 134.25 | 100.33 | 106.98 | 103.65 | 4.70 | 0.05 |
| Integrin beta-4 (Ab-1510) | 309 | 85 | 140.5 | 128 | 134.25 | 295.25 | 89.15 | 192.20 | 145.74 | 0.76 |
| Integrin beta-4 (Phospho-Tyr1510) | 103 | 95 | 140.5 | 128 | 134.25 | 98.42 | 99.64 | 99.03 | 0.86 | 0.01 |
| Interferon-alpha/beta receptor alpha chain (Ab-466) | 375 | 335 | 140.5 | 128 | 134.25 | 358.32 | 351.36 | 354.84 | 4.92 | 0.01 |
| Interferon-alpha/beta receptor alpha chain (Phospho-Tyr466) | 102 | 93 | 140.5 | 128 | 134.25 | 97.46 | 97.54 | 97.50 | 0.06 | 0.00 |
| Interferon-gamma receptor alpha chain precursor (Ab-457) | 102 | 88 | 140.5 | 128 | 134.25 | 97.46 | 92.30 | 94.88 | 3.65 | 0.04 |
| Interferon-gamma receptor alpha chain precursor (Phospho-Tyr457) | 118 | 97 | 140.5 | 128 | 134.25 | 112.75 | 101.74 | 107.24 | 7.79 | 0.07 |
| IR (Ab-1361) | 93 | 79 | 140.5 | 128 | 134.25 | 88.86 | 82.86 | 85.86 | 4.25 | 0.05 |
| IR (Phospho-Tyr1355) | 127 | 79 | 140.5 | 128 | 134.25 | 121.35 | 82.86 | 102.10 | 27.22 | 0.27 |
| IR (Phospho-Tyr1361) | 102 | 87 | 140.5 | 128 | 134.25 | 97.46 | 91.25 | 94.36 | 4.39 | 0.05 |
| IRS-1 (Ab-307) | 116 | 104 | 140.5 | 128 | 134.25 | 110.84 | 109.08 | 109.96 | 1.25 | 0.01 |
| IRS-1 (Ab-312) | 243 | 283 | 140.5 | 128 | 134.25 | 232.19 | 296.82 | 264.50 | 45.70 | 0.17 |
| IRS-1 (Ab-323) | 238 | 211 | 140.5 | 128 | 134.25 | 227.41 | 221.30 | 224.36 | 4.32 | 0.02 |
| IRS-1 (Ab-636) | 127 | 88 | 140.5 | 128 | 134.25 | 121.35 | 92.30 | 106.82 | 20.54 | 0.19 |
| IRS-1 (Ab-639) | 1305 | 819 | 140.5 | 128 | 134.25 | 1246.95 | 858.99 | 1052.97 | 274.33 | 0.26 |
| IRS-1 (Ab-794) | 85 | 68 | 140.5 | 128 | 134.25 | 81.22 | 71.32 | 76.27 | 7.00 | 0.09 |
| IRS-1 (Phospho-Ser1101) | 116 | 68 | 140.5 | 128 | 134.25 | 110.84 | 71.32 | 91.08 | 27.94 | 0.31 |
| IRS-1 (Phospho-Ser307) | 113 | 118 | 140.5 | 128 | 134.25 | 107.97 | 123.76 | 115.87 | 11.16 | 0.10 |
| IRS-1 (Phospho-Ser312) | 88 | 112 | 140.5 | 128 | 134.25 | 84.09 | 117.47 | 100.78 | 23.61 | 0.23 |
| IRS-1 (Phospho-Ser323) | 272 | 65 | 140.5 | 128 | 134.25 | 259.90 | 68.17 | 164.04 | 135.57 | 0.83 |
| IRS-1 (Phospho-Ser612) | 364 | 61 | 140.5 | 128 | 134.25 | 347.81 | 63.98 | 205.89 | 200.70 | 0.97 |
| IRS-1 (Phospho-Ser636) | 130 | 166 | 140.5 | 128 | 134.25 | 124.22 | 174.11 | 149.16 | 35.28 | 0.24 |
| IRS-1 (Phospho-Ser639) | 91 | 233 | 140.5 | 128 | 134.25 | 86.95 | 244.38 | 165.66 | 111.32 | 0.67 |
| IRS-1 (Phospho-Ser794) | 267 | 233 | 140.5 | 128 | 134.25 | 255.12 | 244.38 | 249.75 | 7.60 | 0.03 |
| JAK1 (Ab-1022) | 184 | 164 | 140.5 | 128 | 134.25 | 175.81 | 172.01 | 173.91 | 2.69 | 0.02 |
| JAK1 (Phospho-Tyr1022) | 89 | 87 | 140.5 | 128 | 134.25 | 85.04 | 91.25 | 88.14 | 4.39 | 0.05 |
| JAK2 (Ab-1007) | 441 | 361 | 140.5 | 128 | 134.25 | 421.38 | 378.63 | 400.00 | 30.23 | 0.08 |
| JAK2 (Ab-221) | 129 | 138 | 140.5 | 128 | 134.25 | 123.26 | 144.74 | 134.00 | 15.19 | 0.11 |
| JAK2 (Phospho-Tyr1007) | 91 | 86 | 140.5 | 128 | 134.25 | 86.95 | 90.20 | 88.58 | 2.30 | 0.03 |
| JAK2 (Phospho-Tyr221) | 92 | 94 | 140.5 | 128 | 134.25 | 87.91 | 98.59 | 93.25 | 7.55 | 0.08 |
| JNK1/2/3 (Ab-183/185) | 91 | 73 | 140.5 | 128 | 134.25 | 86.95 | 76.56 | 81.76 | 7.35 | 0.09 |
| JNK1/2/3 (Phospho-Thr183/Tyr185) | 97 | 74 | 140.5 | 128 | 134.25 | 92.69 | 77.61 | 85.15 | 10.66 | 0.13 |
| JunB (Ab-259) | 175 | 131 | 140.5 | 128 | 134.25 | 167.22 | 137.40 | 152.31 | 21.09 | 0.14 |
| JunB (Ab-79) | 334 | 230 | 140.5 | 128 | 134.25 | 319.14 | 241.23 | 280.19 | 55.09 | 0.20 |
| JunB (Phospho-Ser259) | 485 | 121 | 140.5 | 128 | 134.25 | 463.43 | 126.91 | 295.17 | 237.95 | 0.81 |
| JunB (Phospho-Ser79) | 206 | 136 | 140.5 | 128 | 134.25 | 196.84 | 142.64 | 169.74 | 38.32 | 0.23 |
| JunD (Ab-255) | 117 | 79 | 140.5 | 128 | 134.25 | 111.80 | 82.86 | 97.33 | 20.46 | 0.21 |
| JunD (Phospho-Ser255) | 181 | 187 | 140.5 | 128 | 134.25 | 172.95 | 196.13 | 184.54 | 16.39 | 0.09 |
| Keratin 18 (Ab-33) | 100 | 87 | 140.5 | 128 | 134.25 | 95.55 | 91.25 | 93.40 | 3.04 | 0.03 |
| Keratin 18 (Ab-52) | 93 | 79 | 140.5 | 128 | 134.25 | 88.86 | 82.86 | 85.86 | 4.25 | 0.05 |
| Keratin 18 (Phospho-Ser33) | 85 | 72 | 140.5 | 128 | 134.25 | 81.22 | 75.52 | 78.37 | 4.03 | 0.05 |
| Keratin 18 (Phospho-Ser52) | 102 | 67 | 140.5 | 128 | 134.25 | 97.46 | 70.27 | 83.87 | 19.23 | 0.23 |
| Keratin 8 (Ab-431) | 217 | 239 | 140.5 | 128 | 134.25 | 207.35 | 250.67 | 229.01 | 30.63 | 0.13 |
| Keratin 8 (Ab-73) |  | 129 | 140.5 | 128 | 134.25 |  | 135.30 | 135.30 |  |  |
| Keratin 8 (Phospho-Ser431) | 449 | 449 | 140.5 | 128 | 134.25 | 429.03 | 470.92 | 449.98 | 29.63 | 0.07 |
| Keratin 8 (Phospho-Ser73) | 83 | 72 | 140.5 | 128 | 134.25 | 79.31 | 75.52 | 77.41 | 2.68 | 0.03 |
| KIT (Ab-721) | 118 | 104 | 140.5 | 128 | 134.25 | 112.75 | 109.08 | 110.91 | 2.60 | 0.02 |
| KIT (Ab-936) | 87 | 297 | 140.5 | 128 | 134.25 | 83.13 | 311.50 | 197.32 | 161.48 | 0.82 |
| KIT (Phospho-Tyr703) | 70 | 317 | 140.5 | 128 | 134.25 | 66.89 | 332.48 | 199.68 | 187.80 | 0.94 |
| KIT (Phospho-Tyr721) | 307 | 240 | 140.5 | 128 | 134.25 | 293.34 | 251.72 | 272.53 | 29.43 | 0.11 |
| KIT (Phospho-Tyr936) | 179 | 224 | 140.5 | 128 | 134.25 | 171.04 | 234.94 | 202.99 | 45.18 | 0.22 |
| KSR (Ab-392) | 100 | 70 | 140.5 | 128 | 134.25 | 95.55 | 73.42 | 84.48 | 15.65 | 0.19 |
| KSR (Phospho-Ser392) | 75 | 72 | 140.5 | 128 | 134.25 | 71.66 | 75.52 | 73.59 | 2.72 | 0.04 |
| Kv1.3/KCNA3 (Ab-135) | 166 | 148 | 140.5 | 128 | 134.25 | 158.62 | 155.23 | 156.92 | 2.40 | 0.02 |
| Kv1.3/KCNA3 (Phospho-Tyr135) |  | 710 | 140.5 | 128 | 134.25 |  | 744.67 | 744.67 |  |  |
| Kv2.1/Kcnb1 (Phospho-Tyr128) | 380 | 369 | 140.5 | 128 | 134.25 | 363.10 | 387.02 | 375.06 | 16.92 | 0.05 |
| Lamin A (Ab-22) | 79 | 80 | 140.5 | 128 | 134.25 | 75.49 | 83.91 | 79.70 | 5.95 | 0.07 |
| Lamin A (Phospho-Ser22) | 404 | 322 | 140.5 | 128 | 134.25 | 386.03 | 337.72 | 361.88 | 34.16 | 0.09 |
| Lamin A/C (Ab-392) | 3641 | 3355 | 140.5 | 128 | 134.25 | 3479.03 | 3518.82 | 3498.93 | 28.13 | 0.01 |
| Lamin A/C (Phospho-Ser392) | 83 | 91 | 140.5 | 128 | 134.25 | 79.31 | 95.44 | 87.38 | 11.41 | 0.13 |
| LAT (Ab-161) | 125 | 96 | 140.5 | 128 | 134.25 | 119.44 | 100.69 | 110.06 | 13.26 | 0.12 |
| LAT (Ab-171) | 348 | 301 | 140.5 | 128 | 134.25 | 332.52 | 315.70 | 324.11 | 11.90 | 0.04 |
| LAT (Ab-191) | 270 | 210 | 140.5 | 128 | 134.25 | 257.99 | 220.25 | 239.12 | 26.68 | 0.11 |
| LAT (Phospho-Tyr171) | 78 | 79 | 140.5 | 128 | 134.25 | 74.53 | 82.86 | 78.69 | 5.89 | 0.07 |
| LAT (Phospho-Tyr191) | 193 | 314 | 140.5 | 128 | 134.25 | 184.41 | 329.33 | 256.87 | 102.47 | 0.40 |
| LCK (Ab-192) | 102 | 106 | 140.5 | 128 | 134.25 | 97.46 | 111.18 | 104.32 | 9.70 | 0.09 |
| LCK (Ab-393) | 2334 | 1341 | 140.5 | 128 | 134.25 | 2230.17 | 1406.48 | 1818.33 | 582.44 | 0.32 |
| LCK (Ab-504) | 274 | 268 | 140.5 | 128 | 134.25 | 261.81 | 281.09 | 271.45 | 13.63 | 0.05 |
| LCK (Ab-59) | 88 | 68 | 140.5 | 128 | 134.25 | 84.09 | 71.32 | 77.70 | 9.03 | 0.12 |
| LCK (Phospho-Ser59) | 88 | 112 | 140.5 | 128 | 134.25 | 84.09 | 117.47 | 100.78 | 23.61 | 0.23 |
| LCK (Phospho-Tyr192) | 90 | 74 | 140.5 | 128 | 134.25 | 86.00 | 77.61 | 81.80 | 5.93 | 0.07 |
| LCK (Phospho-Tyr393) | 274 | 240 | 140.5 | 128 | 134.25 | 261.81 | 251.72 | 256.77 | 7.14 | 0.03 |
| LCK (Phospho-Tyr504) | 325 | 280 | 140.5 | 128 | 134.25 | 310.54 | 293.67 | 302.11 | 11.93 | 0.04 |
| LIMK1 (Ab-508) | 343 | 294 | 140.5 | 128 | 134.25 | 327.74 | 308.36 | 318.05 | 13.71 | 0.04 |
| LIMK1 (Phospho-Thr508) | 105 | 92 | 140.5 | 128 | 134.25 | 100.33 | 96.49 | 98.41 | 2.71 | 0.03 |
| LIMK1/2 (Ab-508/505) | 225 | 349 | 140.5 | 128 | 134.25 | 214.99 | 366.04 | 290.52 | 106.81 | 0.37 |
| LKB1 (Ab-189) | 106 | 82 | 140.5 | 128 | 134.25 | 101.28 | 86.00 | 93.64 | 10.81 | 0.12 |
| LKB1 (Ab-334) | 555 | 423 | 140.5 | 128 | 134.25 | 530.31 | 443.65 | 486.98 | 61.28 | 0.13 |
| LKB1 (Ab-428) | 94 | 76 | 140.5 | 128 | 134.25 | 89.82 | 79.71 | 84.76 | 7.15 | 0.08 |
| LKB1 (Phospho-Ser428) | 130 | 140 | 140.5 | 128 | 134.25 | 124.22 | 146.84 | 135.53 | 15.99 | 0.12 |
| LKB1 (Phospho-Thr189) | 103 | 81 | 140.5 | 128 | 134.25 | 98.42 | 84.96 | 91.69 | 9.52 | 0.10 |
| LYN (Ab-507) | 1451 | 2664 | 140.5 | 128 | 134.25 | 1386.45 | 2794.08 | 2090.27 | 995.34 | 0.48 |
| LYN (Phospho-Tyr507) | 188 | 320 | 140.5 | 128 | 134.25 | 179.64 | 335.63 | 257.63 | 110.30 | 0.43 |
| MAP3K1/MEKK1 (Phospho-Thr1381) | 154 | 123 | 140.5 | 128 | 134.25 | 147.15 | 129.01 | 138.08 | 12.83 | 0.09 |
| MAP3K7/TAK1 (Ab-184) | 114 | 101 | 140.5 | 128 | 134.25 | 108.93 | 105.93 | 107.43 | 2.12 | 0.02 |
| MAP3K7/TAK1 (Ab-187) | 94 | 88 | 140.5 | 128 | 134.25 | 89.82 | 92.30 | 91.06 | 1.75 | 0.02 |
| MAP3K7/TAK1 (Ab-439) | 276 | 245 | 140.5 | 128 | 134.25 | 263.72 | 256.96 | 260.34 | 4.78 | 0.02 |
| MAP3K7/TAK1 (Phospho-Thr184) | 157 | 116 | 140.5 | 128 | 134.25 | 150.02 | 121.66 | 135.84 | 20.05 | 0.15 |
| MAP3K8/COT (Ab-290) | 90 | 207 | 140.5 | 128 | 134.25 | 86.00 | 217.11 | 151.55 | 92.71 | 0.61 |
| MAP3K8/COT (Ab-400) | 136 | 102 | 140.5 | 128 | 134.25 | 129.95 | 106.98 | 118.47 | 16.24 | 0.14 |
| MAP3K8/COT (Phospho-Thr290) | 74 | 64 | 140.5 | 128 | 134.25 | 70.71 | 67.13 | 68.92 | 2.53 | 0.04 |
| MAPKAPK2 (Ab-272) | 122 | 126 | 140.5 | 128 | 134.25 | 116.57 | 132.15 | 124.36 | 11.02 | 0.09 |
| MAPKAPK2 (Phospho-Ser272) | 121 | 137 | 140.5 | 128 | 134.25 | 115.62 | 143.69 | 129.65 | 19.85 | 0.15 |
| MAPKAPK2 (Phospho-Thr222) | 101 | 496 | 140.5 | 128 | 134.25 | 96.51 | 520.22 | 308.36 | 299.61 | 0.97 |
| MAPKAPK2 (Phospho-Thr334) | 410 | 190 | 140.5 | 128 | 134.25 | 391.76 | 199.28 | 295.52 | 136.11 | 0.46 |
| MARCKS (Ab-158) | 180 | 167 | 140.5 | 128 | 134.25 | 171.99 | 175.15 | 173.57 | 2.24 | 0.01 |
| MARCKS (Ab-163) | 103 | 91 | 140.5 | 128 | 134.25 | 98.42 | 95.44 | 96.93 | 2.10 | 0.02 |
| MARCKS (Phospho-Ser158) | 132 | 133 | 140.5 | 128 | 134.25 | 126.13 | 139.49 | 132.81 | 9.45 | 0.07 |
| MARCKS (Phospho-Ser163) | 397 | 180 | 140.5 | 128 | 134.25 | 379.34 | 188.79 | 284.06 | 134.74 | 0.47 |
| M-CSF Receptor (Ab-561) | 86 | 83 | 140.5 | 128 | 134.25 | 82.17 | 87.05 | 84.61 | 3.45 | 0.04 |
| M-CSF Receptor (Ab-809) | 115 | 95 | 140.5 | 128 | 134.25 | 109.88 | 99.64 | 104.76 | 7.24 | 0.07 |
| M-CSF Receptor (Phospho-Tyr561) | 314 | 94 | 140.5 | 128 | 134.25 | 300.03 | 98.59 | 199.31 | 142.44 | 0.71 |
| M-CSF Receptor (Phospho-Tyr809) | 292 | 215 | 140.5 | 128 | 134.25 | 279.01 | 225.50 | 252.25 | 37.84 | 0.15 |
| MDM2 (Ab-166) | 5036 | 4836 | 140.5 | 128 | 134.25 | 4811.98 | 5072.13 | 4942.06 | 183.96 | 0.04 |
| MDM2 (Phospho-Ser166) | 98 | 154 | 140.5 | 128 | 134.25 | 93.64 | 161.52 | 127.58 | 48.00 | 0.38 |
| MDM4 (Phospho-Ser367) | 88 | 338 | 140.5 | 128 | 134.25 | 84.09 | 354.50 | 219.29 | 191.21 | 0.87 |
| MEF2A (Ab-312) | 2370 | 1627 | 140.5 | 128 | 134.25 | 2264.57 | 1706.44 | 1985.51 | 394.66 | 0.20 |
| MEF2A (Ab-319) | 320 | 260 | 140.5 | 128 | 134.25 | 305.77 | 272.70 | 289.23 | 23.38 | 0.08 |
| MEF2A (Ab-408) | 534 | 572 | 140.5 | 128 | 134.25 | 510.25 | 599.93 | 555.09 | 63.42 | 0.11 |
| MEF2A (Phospho-Ser408) | 105 | 104 | 140.5 | 128 | 134.25 | 100.33 | 109.08 | 104.70 | 6.19 | 0.06 |
| MEF2A (Phospho-Thr312) | 348 | 334 | 140.5 | 128 | 134.25 | 332.52 | 350.31 | 341.41 | 12.58 | 0.04 |
| MEF2A (Phospho-Thr319) | 373 | 377 | 140.5 | 128 | 134.25 | 356.41 | 395.41 | 375.91 | 27.58 | 0.07 |
| MEF2C (Ab-396) | 112 | 137 | 140.5 | 128 | 134.25 | 107.02 | 143.69 | 125.35 | 25.93 | 0.21 |
| MEF2C (Phospho-Ser396) | 100 | 287 | 140.5 | 128 | 134.25 | 95.55 | 301.01 | 198.28 | 145.28 | 0.73 |
| MEF2D (Phospho-Ser444) | 115 | 80 | 140.5 | 128 | 134.25 | 109.88 | 83.91 | 96.90 | 18.37 | 0.19 |
| MEK1 (Ab-217) | 160 | 156 | 140.5 | 128 | 134.25 | 152.88 | 163.62 | 158.25 | 7.59 | 0.05 |
| MEK1 (Ab-221) | 104 | 94 | 140.5 | 128 | 134.25 | 99.37 | 98.59 | 98.98 | 0.55 | 0.01 |
| MEK1 (Ab-286) | 304 | 313 | 140.5 | 128 | 134.25 | 290.48 | 328.28 | 309.38 | 26.73 | 0.09 |
| MEK1 (Ab-291) | 268 | 262 | 140.5 | 128 | 134.25 | 256.08 | 274.79 | 265.44 | 13.23 | 0.05 |
| MEK1 (Ab-298) | 372 | 391 | 140.5 | 128 | 134.25 | 355.45 | 410.09 | 382.77 | 38.64 | 0.10 |
| MEK1 (Phospho-Ser217) | 536 | 95 | 140.5 | 128 | 134.25 | 512.16 | 99.64 | 305.90 | 291.69 | 0.95 |
| MEK1 (Phospho-Ser221) | 141 | 122 | 140.5 | 128 | 134.25 | 134.73 | 127.96 | 131.34 | 4.79 | 0.04 |
| MEK1 (Phospho-Ser298) | 250 | 77 | 140.5 | 128 | 134.25 | 238.88 | 80.76 | 159.82 | 111.81 | 0.70 |
| MEK1 (Phospho-Thr286) | 816 | 678 | 140.5 | 128 | 134.25 | 779.70 | 711.11 | 745.40 | 48.50 | 0.07 |
| MEK1 (Phospho-Thr291) | 158 | 266 | 140.5 | 128 | 134.25 | 150.97 | 278.99 | 214.98 | 90.52 | 0.42 |
| MEK2 (Ab-394) | 170 | 200 | 140.5 | 128 | 134.25 | 162.44 | 209.77 | 186.10 | 33.47 | 0.18 |
| MEK2 (Phospho-Thr394) | 572 | 470 | 140.5 | 128 | 134.25 | 546.56 | 492.95 | 519.75 | 37.91 | 0.07 |
| MER/SKY (Phospho-Tyr749/Tyr681) | 176 | 83 | 140.5 | 128 | 134.25 | 168.17 | 87.05 | 127.61 | 57.36 | 0.45 |
| Merlin (Ab-10) | 870 | 118 | 140.5 | 128 | 134.25 | 831.30 | 123.76 | 477.53 | 500.30 | 1.05 |
| Merlin (Ab-518) | 93 | 81 | 140.5 | 128 | 134.25 | 88.86 | 84.96 | 86.91 | 2.76 | 0.03 |
| Merlin (Phospho-Ser10) | 95 | 91 | 140.5 | 128 | 134.25 | 90.77 | 95.44 | 93.11 | 3.30 | 0.04 |
| Merlin (Phospho-Ser518) | 128 | 116 | 140.5 | 128 | 134.25 | 122.31 | 121.66 | 121.99 | 0.45 | 0.00 |
| Met (Ab-1003) | 106 | 175 | 140.5 | 128 | 134.25 | 101.28 | 183.54 | 142.41 | 58.17 | 0.41 |
| Met (Ab-1234) | 200 | 136 | 140.5 | 128 | 134.25 | 191.10 | 142.64 | 166.87 | 34.27 | 0.21 |
| Met (Ab-1349) | 152 | 125 | 140.5 | 128 | 134.25 | 145.24 | 131.10 | 138.17 | 9.99 | 0.07 |
| Met (Phospho-Tyr1003) | 94 | 66 | 140.5 | 128 | 134.25 | 89.82 | 69.22 | 79.52 | 14.56 | 0.18 |
| Met (Phospho-Tyr1234) | 190 | 211 | 140.5 | 128 | 134.25 | 181.55 | 221.30 | 201.43 | 28.11 | 0.14 |
| Met (Phospho-Tyr1349) | 112 | 125 | 140.5 | 128 | 134.25 | 107.02 | 131.10 | 119.06 | 17.03 | 0.14 |
| Met (Phospho-Tyr1356) | 4107 | 3360 | 140.5 | 128 | 134.25 | 3924.30 | 3524.06 | 3724.18 | 283.01 | 0.08 |
| MITF (Ab-73) | 575 | 557 | 140.5 | 128 | 134.25 | 549.42 | 584.20 | 566.81 | 24.59 | 0.04 |
| MITF (Phospho-Ser73) | 299 | 207 | 140.5 | 128 | 134.25 | 285.70 | 217.11 | 251.40 | 48.50 | 0.19 |
| MKK3/MAP2K3 (Ab-189) | 203 | 236 | 140.5 | 128 | 134.25 | 193.97 | 247.52 | 220.75 | 37.87 | 0.17 |
| MKK3/MAP2K3 (Ab-222) | 77 | 71 | 140.5 | 128 | 134.25 | 73.57 | 74.47 | 74.02 | 0.63 | 0.01 |
| MKK3/MAP2K3 (Phospho-Ser189) | 123 | 95 | 140.5 | 128 | 134.25 | 117.53 | 99.64 | 108.58 | 12.65 | 0.12 |
| MKK3/MAP2K3 (Phospho-Thr222) | 117 | 98 | 140.5 | 128 | 134.25 | 111.80 | 102.79 | 107.29 | 6.37 | 0.06 |
| MKK4/SEK1 (Ab-257) | 87 | 78 | 140.5 | 128 | 134.25 | 83.13 | 81.81 | 82.47 | 0.93 | 0.01 |
| MKK4/SEK1 (Ab-261) | 1097 | 980 | 140.5 | 128 | 134.25 | 1048.20 | 1027.85 | 1038.03 | 14.39 | 0.01 |
| MKK4/SEK1 (Ab-80) | 775 | 697 | 140.5 | 128 | 134.25 | 740.52 | 731.03 | 735.78 | 6.71 | 0.01 |
| MKK4/SEK1 (Phospho-Ser257) | 73 | 73 | 140.5 | 128 | 134.25 | 69.75 | 76.56 | 73.16 | 4.82 | 0.07 |
| MKK4/SEK1 (Phospho-Ser80) | 182 | 181 | 140.5 | 128 | 134.25 | 173.90 | 189.84 | 181.87 | 11.27 | 0.06 |
| MKK4/SEK1 (Phospho-Thr261) | 100 | 95 | 140.5 | 128 | 134.25 | 95.55 | 99.64 | 97.60 | 2.89 | 0.03 |
| MKK6/MAP2K6 (Ab-207) | 714 | 708 | 140.5 | 128 | 134.25 | 682.24 | 742.57 | 712.40 | 42.66 | 0.06 |
| MKK6/MAP2K6 (Phospho-Ser207) | 353 | 236 | 140.5 | 128 | 134.25 | 337.30 | 247.52 | 292.41 | 63.48 | 0.22 |
| MKK7/MAP2K7 (Ab-271) | 130 | 116 | 140.5 | 128 | 134.25 | 124.22 | 121.66 | 122.94 | 1.81 | 0.01 |
| MKK7/MAP2K7 (Phospho-Ser271) | 85 | 70 | 140.5 | 128 | 134.25 | 81.22 | 73.42 | 77.32 | 5.52 | 0.07 |
| MKK7/MAP2K7 (Phospho-Thr275) | 237 | 68 | 140.5 | 128 | 134.25 | 226.46 | 71.32 | 148.89 | 109.70 | 0.74 |
| MKP-1 (Ab-359) | 134 | 116 | 140.5 | 128 | 134.25 | 128.04 | 121.66 | 124.85 | 4.51 | 0.04 |
| MKP-1 (Phospho-Ser359) | 204 | 77 | 140.5 | 128 | 134.25 | 194.93 | 80.76 | 137.84 | 80.73 | 0.59 |
| MKP-1/2 (Ab-296/318) | 233 | 260 | 140.5 | 128 | 134.25 | 222.64 | 272.70 | 247.67 | 35.40 | 0.14 |
| MKP-1/2 (Phospho-Ser296/318) | 354 | 320 | 140.5 | 128 | 134.25 | 338.25 | 335.63 | 336.94 | 1.86 | 0.01 |
| Mnk1 (Ab-385) | 101 | 124 | 140.5 | 128 | 134.25 | 96.51 | 130.05 | 113.28 | 23.72 | 0.21 |
| Mnk1 (Phospho-Thr385) | 109 | 105 | 140.5 | 128 | 134.25 | 104.15 | 110.13 | 107.14 | 4.23 | 0.04 |
| MSK1 (Ab-360) | 2430 | 1495 | 140.5 | 128 | 134.25 | 2321.90 | 1568.00 | 1944.95 | 533.09 | 0.27 |
| MSK1 (Ab-376) | 169 | 128 | 140.5 | 128 | 134.25 | 161.48 | 134.25 | 147.87 | 19.26 | 0.13 |
| MSK1 (Ab-581) | 278 | 196 | 140.5 | 128 | 134.25 | 265.63 | 205.57 | 235.60 | 42.47 | 0.18 |
| MSK1 (Phospho-Ser212) | 612 | 520 | 140.5 | 128 | 134.25 | 584.78 | 545.39 | 565.08 | 27.85 | 0.05 |
| MSK1 (Phospho-Ser360) | 345 | 480 | 140.5 | 128 | 134.25 | 329.65 | 503.44 | 416.55 | 122.88 | 0.30 |
| MSK1 (Phospho-Ser376) | 476 | 276 | 140.5 | 128 | 134.25 | 454.83 | 289.48 | 372.15 | 116.92 | 0.31 |
| MSK1 (Phospho-Thr581) | 92 | 84 | 140.5 | 128 | 134.25 | 87.91 | 88.10 | 88.00 | 0.14 | 0.00 |
| MSK2 (Phospho-Thr568) | 116 | 88 | 140.5 | 128 | 134.25 | 110.84 | 92.30 | 101.57 | 13.11 | 0.13 |
| Mst1/Mst2 (Ab-183) | 85 | 88 | 140.5 | 128 | 134.25 | 81.22 | 92.30 | 86.76 | 7.83 | 0.09 |
| Mst1/Mst2 (Phospho-Thr183) | 113 | 69 | 140.5 | 128 | 134.25 | 107.97 | 72.37 | 90.17 | 25.18 | 0.28 |
| mTOR (Ab-2446) | 71 | 59 | 140.5 | 128 | 134.25 | 67.84 | 61.88 | 64.86 | 4.21 | 0.06 |
| mTOR (Ab-2448) | 93 | 70 | 140.5 | 128 | 134.25 | 88.86 | 73.42 | 81.14 | 10.92 | 0.13 |
| mTOR (Ab-2481) | 121 | 103 | 140.5 | 128 | 134.25 | 115.62 | 108.03 | 111.82 | 5.37 | 0.05 |
| mTOR (Phospho-Ser2448) | 242 | 109 | 140.5 | 128 | 134.25 | 231.23 | 114.32 | 172.78 | 82.67 | 0.48 |
| mTOR (Phospho-Ser2481) | 161 | 163 | 140.5 | 128 | 134.25 | 153.84 | 170.96 | 162.40 | 12.11 | 0.07 |
| mTOR (Phospho-Thr2446) | 74 | 203 | 140.5 | 128 | 134.25 | 70.71 | 212.91 | 141.81 | 100.55 | 0.71 |
| Myc (Ab-358) | 3488 | 3200 | 140.5 | 128 | 134.25 | 3332.84 | 3356.25 | 3344.54 | 16.55 | 0.00 |
| Myc (Ab-373) | 242 | 196 | 140.5 | 128 | 134.25 | 231.23 | 205.57 | 218.40 | 18.15 | 0.08 |
| Myc (Ab-58) | 126 | 78 | 140.5 | 128 | 134.25 | 120.40 | 81.81 | 101.10 | 27.28 | 0.27 |
| Myc (Ab-62) | 109 | 105 | 140.5 | 128 | 134.25 | 104.15 | 110.13 | 107.14 | 4.23 | 0.04 |
| Myc (Phospho-Ser373) | 123 | 113 | 140.5 | 128 | 134.25 | 117.53 | 118.52 | 118.02 | 0.70 | 0.01 |
| Myc (Phospho-Ser62) | 319 | 336 | 140.5 | 128 | 134.25 | 304.81 | 352.41 | 328.61 | 33.66 | 0.10 |
| Myc (Phospho-Thr358) | 198 | 476 | 140.5 | 128 | 134.25 | 189.19 | 499.24 | 344.22 | 219.24 | 0.64 |
| Myc (Phospho-Thr58) | 113 | 294 | 140.5 | 128 | 134.25 | 107.97 | 308.36 | 208.16 | 141.69 | 0.68 |
| Myosin regulatory light chain 2 (Ab-18) | 236 | 235 | 140.5 | 128 | 134.25 | 225.50 | 246.47 | 235.99 | 14.83 | 0.06 |
| Myosin regulatory light chain 2 (Phospho-Ser18) | 172 | 147 | 140.5 | 128 | 134.25 | 164.35 | 154.18 | 159.26 | 7.19 | 0.05 |
| MYPT1 (Phospho-Thr696) | 248 | 87 | 140.5 | 128 | 134.25 | 236.97 | 91.25 | 164.11 | 103.04 | 0.63 |
| MYPT1 (Phospho-Thr853) | 197 | 175 | 140.5 | 128 | 134.25 | 188.24 | 183.54 | 185.89 | 3.32 | 0.02 |
| MYT1 (Ab-83) | 263 | 241 | 140.5 | 128 | 134.25 | 251.30 | 252.77 | 252.03 | 1.04 | 0.00 |
| NFAT3 (Ab-168/170) | 122 | 135 | 140.5 | 128 | 134.25 | 116.57 | 141.59 | 129.08 | 17.69 | 0.14 |
| NFAT3 (Ab-676) | 297 | 271 | 140.5 | 128 | 134.25 | 283.79 | 284.23 | 284.01 | 0.31 | 0.00 |
| NFAT4 (Ab-165) | 84 | 82 | 140.5 | 128 | 134.25 | 80.26 | 86.00 | 83.13 | 4.06 | 0.05 |
| NFAT4 (Phospho-Ser165) | 131 | 67 | 140.5 | 128 | 134.25 | 125.17 | 70.27 | 97.72 | 38.82 | 0.40 |
| NFkB-p100/p52 (Ab-865) | 1017 | 974 | 140.5 | 128 | 134.25 | 971.76 | 1021.56 | 996.66 | 35.21 | 0.04 |
| NFkB-p100/p52 (Ab-869) | 375 | 155 | 140.5 | 128 | 134.25 | 358.32 | 162.57 | 260.44 | 138.42 | 0.53 |
| NFkB-p100/p52 (Phospho-Ser865) | 535 | 364 | 140.5 | 128 | 134.25 | 511.20 | 381.77 | 446.49 | 91.52 | 0.20 |
| NFkB-p100/p52 (Phospho-Ser869) | 171 | 374 | 140.5 | 128 | 134.25 | 163.39 | 392.26 | 277.83 | 161.83 | 0.58 |
| NFkB-p100/p52 (Phospho-Ser872) | 699 | 722 | 140.5 | 128 | 134.25 | 667.91 | 757.25 | 712.58 | 63.18 | 0.09 |
| NFkB-p105/p50 (Ab-337) | 130 | 122 | 140.5 | 128 | 134.25 | 124.22 | 127.96 | 126.09 | 2.64 | 0.02 |
| NFkB-p105/p50 (Ab-893) | 212 | 144 | 140.5 | 128 | 134.25 | 202.57 | 151.03 | 176.80 | 36.44 | 0.21 |
| NFkB-p105/p50 (Ab-907) | 235 | 224 | 140.5 | 128 | 134.25 | 224.55 | 234.94 | 229.74 | 7.35 | 0.03 |
| NFkB-p105/p50 (Ab-927) | 1416 | 935 | 140.5 | 128 | 134.25 | 1353.01 | 980.65 | 1166.83 | 263.30 | 0.23 |
| NFkB-p105/p50 (Ab-932) | 91 | 74 | 140.5 | 128 | 134.25 | 86.95 | 77.61 | 82.28 | 6.60 | 0.08 |
| NFkB-p105/p50 (Phospho-Ser337) | 1201 | 1124 | 140.5 | 128 | 134.25 | 1147.57 | 1178.88 | 1163.23 | 22.14 | 0.02 |
| NFkB-p105/p50 (Phospho-Ser893) | 125 | 100 | 140.5 | 128 | 134.25 | 119.44 | 104.88 | 112.16 | 10.29 | 0.09 |
| NFkB-p105/p50 (Phospho-Ser907) | 157 | 131 | 140.5 | 128 | 134.25 | 150.02 | 137.40 | 143.71 | 8.92 | 0.06 |
| NFkB-p105/p50 (Phospho-Ser927) | 90 | 298 | 140.5 | 128 | 134.25 | 86.00 | 312.55 | 199.27 | 160.20 | 0.80 |
| NFkB-p105/p50 (Phospho-Ser932) | 111 | 91 | 140.5 | 128 | 134.25 | 106.06 | 95.44 | 100.75 | 7.51 | 0.07 |
| NFkB-p65 (Ab-254) | 116 | 107 | 140.5 | 128 | 134.25 | 110.84 | 112.22 | 111.53 | 0.98 | 0.01 |
| NFkB-p65 (Ab-276) | 120 | 111 | 140.5 | 128 | 134.25 | 114.66 | 116.42 | 115.54 | 1.24 | 0.01 |
| NFkB-p65 (Ab-281) | 115 | 132 | 140.5 | 128 | 134.25 | 109.88 | 138.45 | 124.16 | 20.20 | 0.16 |
| NFkB-p65 (Ab-311) | 142 | 134 | 140.5 | 128 | 134.25 | 135.68 | 140.54 | 138.11 | 3.44 | 0.02 |
| NFkB-p65 (Ab-435) | 143 | 115 | 140.5 | 128 | 134.25 | 136.64 | 120.62 | 128.63 | 11.33 | 0.09 |
| NFkB-p65 (Ab-468) | 225 | 184 | 140.5 | 128 | 134.25 | 214.99 | 192.98 | 203.99 | 15.56 | 0.08 |
| NFkB-p65 (Ab-505) | 239 | 231 | 140.5 | 128 | 134.25 | 228.37 | 242.28 | 235.32 | 9.84 | 0.04 |
| NFkB-p65 (Ab-529) | 294 | 239 | 140.5 | 128 | 134.25 | 280.92 | 250.67 | 265.80 | 21.39 | 0.08 |
| NFkB-p65 (Ab-536) | 117 | 93 | 140.5 | 128 | 134.25 | 111.80 | 97.54 | 104.67 | 10.08 | 0.10 |
| NFkB-p65 (Phospho-Ser276) | 322 | 374 | 140.5 | 128 | 134.25 | 307.68 | 392.26 | 349.97 | 59.81 | 0.17 |
| NFkB-p65 (Phospho-Ser311) | 120 | 129 | 140.5 | 128 | 134.25 | 114.66 | 135.30 | 124.98 | 14.59 | 0.12 |
| NFkB-p65 (Phospho-Ser468) | 754 | 313 | 140.5 | 128 | 134.25 | 720.46 | 328.28 | 524.37 | 277.31 | 0.53 |
| NFkB-p65 (Phospho-Ser529) | 157 | 391 | 140.5 | 128 | 134.25 | 150.02 | 410.09 | 280.05 | 183.90 | 0.66 |
| NFkB-p65 (Phospho-Ser536) | 102 | 81 | 140.5 | 128 | 134.25 | 97.46 | 84.96 | 91.21 | 8.84 | 0.10 |
| NFkB-p65 (Phospho-Thr254) | 135 | 103 | 140.5 | 128 | 134.25 | 128.99 | 108.03 | 118.51 | 14.82 | 0.13 |
| NFkB-p65 (Phospho-Thr435) | 154 | 146 | 140.5 | 128 | 134.25 | 147.15 | 153.13 | 150.14 | 4.23 | 0.03 |
| NFkB-p65 (Phospho-Thr505) | 172 | 153 | 140.5 | 128 | 134.25 | 164.35 | 160.47 | 162.41 | 2.74 | 0.02 |
| NMDAR1 (Ab-897) | 124 | 88 | 140.5 | 128 | 134.25 | 118.48 | 92.30 | 105.39 | 18.52 | 0.18 |
| NMDAR1 (Phospho-Ser897) | 360 | 281 | 140.5 | 128 | 134.25 | 343.99 | 294.72 | 319.35 | 34.84 | 0.11 |
| NMDAR2A/B (Phospho-Tyr1246/1252) | 219 | 515 | 140.5 | 128 | 134.25 | 209.26 | 540.15 | 374.70 | 233.97 | 0.62 |
| NMDAR2B (Ab-1472) | 1673 | 1598 | 140.5 | 128 | 134.25 | 1598.58 | 1676.03 | 1637.30 | 54.76 | 0.03 |
| NMDAR2B (Phospho-Tyr1472) | 115 | 91 | 140.5 | 128 | 134.25 | 109.88 | 95.44 | 102.66 | 10.21 | 0.10 |
| Opioid Receptor (Ab-375) | 242 | 73 | 140.5 | 128 | 134.25 | 231.23 | 76.56 | 153.90 | 109.37 | 0.71 |
| Opioid Receptor (Phospho-Ser375) | 354 | 245 | 140.5 | 128 | 134.25 | 338.25 | 256.96 | 297.61 | 57.48 | 0.19 |
| p130Cas (Ab-165) | 105 | 97 | 140.5 | 128 | 134.25 | 100.33 | 101.74 | 101.03 | 1.00 | 0.01 |
| p130Cas (Ab-410) | 73 | 67 | 140.5 | 128 | 134.25 | 69.75 | 70.27 | 70.01 | 0.37 | 0.01 |
| p130Cas (Phospho-Tyr165) | 77 | 69 | 140.5 | 128 | 134.25 | 73.57 | 72.37 | 72.97 | 0.85 | 0.01 |
| p130Cas (Phospho-Tyr410) | 75 | 77 | 140.5 | 128 | 134.25 | 71.66 | 80.76 | 76.21 | 6.43 | 0.08 |
| p21Cip1 (Ab-145) | 132 | 110 | 140.5 | 128 | 134.25 | 126.13 | 115.37 | 120.75 | 7.61 | 0.06 |
| p21Cip1 (Phospho-Thr145) | 140 | 155 | 140.5 | 128 | 134.25 | 133.77 | 162.57 | 148.17 | 20.36 | 0.14 |
| p27Kip1 (Ab-10) | 100 | 84 | 140.5 | 128 | 134.25 | 95.55 | 88.10 | 91.83 | 5.27 | 0.06 |
| p27Kip1 (Ab-187) | 104 | 83 | 140.5 | 128 | 134.25 | 99.37 | 87.05 | 93.21 | 8.71 | 0.09 |
| p27Kip1 (Phospho-Ser10) | 141 | 245 | 140.5 | 128 | 134.25 | 134.73 | 256.96 | 195.85 | 86.43 | 0.44 |
| p27Kip1 (Phospho-Thr187) | 131 | 360 | 140.5 | 128 | 134.25 | 125.17 | 377.58 | 251.38 | 178.48 | 0.71 |
| p300 (Ab-89) | 406 | 432 | 140.5 | 128 | 134.25 | 387.94 | 453.09 | 420.52 | 46.07 | 0.11 |
| p38 MAPK (Ab-180) | 77 | 72 | 140.5 | 128 | 134.25 | 73.57 | 75.52 | 74.55 | 1.37 | 0.02 |
| p38 MAPK (Ab-182) | 2536 | 2390 | 140.5 | 128 | 134.25 | 2423.19 | 2506.70 | 2464.94 | 59.05 | 0.02 |
| p38 MAPK (Ab-322) | 85 | 71 | 140.5 | 128 | 134.25 | 81.22 | 74.47 | 77.84 | 4.77 | 0.06 |
| p38 MAPK (Phospho-Thr180) | 134 | 92 | 140.5 | 128 | 134.25 | 128.04 | 96.49 | 112.27 | 22.31 | 0.20 |
| p38 MAPK (Phospho-Tyr182) | 190 | 92 | 140.5 | 128 | 134.25 | 181.55 | 96.49 | 139.02 | 60.14 | 0.43 |
| p38 MAPK (Phospho-Tyr322) | 207 | 79 | 140.5 | 128 | 134.25 | 197.79 | 82.86 | 140.32 | 81.27 | 0.58 |
| p44/42 MAPK (Ab-202) | 115 | 94 | 140.5 | 128 | 134.25 | 109.88 | 98.59 | 104.24 | 7.99 | 0.08 |
| p44/42 MAPK (Ab-204) | 93 | 72 | 140.5 | 128 | 134.25 | 88.86 | 75.52 | 82.19 | 9.44 | 0.11 |
| p44/42 MAPK (Phospho-Thr202) | 148 | 310 | 140.5 | 128 | 134.25 | 141.42 | 325.14 | 233.28 | 129.91 | 0.56 |
| p44/42 MAPK (Phospho-Tyr204) | 246 | 227 | 140.5 | 128 | 134.25 | 235.06 | 238.08 | 236.57 | 2.14 | 0.01 |
| p53 (Ab-15) | 120 | 113 | 140.5 | 128 | 134.25 | 114.66 | 118.52 | 116.59 | 2.73 | 0.02 |
| p53 (Ab-18) | 265 | 253 | 140.5 | 128 | 134.25 | 253.21 | 265.35 | 259.28 | 8.59 | 0.03 |
| p53 (Ab-20) | 581 | 524 | 140.5 | 128 | 134.25 | 555.15 | 549.59 | 552.37 | 3.94 | 0.01 |
| p53 (Ab-315) | 633 | 725 | 140.5 | 128 | 134.25 | 604.84 | 760.40 | 682.62 | 110.00 | 0.16 |
| p53 (Ab-33) | 186 | 153 | 140.5 | 128 | 134.25 | 177.73 | 160.47 | 169.10 | 12.20 | 0.07 |
| p53 (Ab-37) | 181 | 146 | 140.5 | 128 | 134.25 | 172.95 | 153.13 | 163.04 | 14.01 | 0.09 |
| p53 (Ab-376) | 219 | 79 | 140.5 | 128 | 134.25 | 209.26 | 82.86 | 146.06 | 89.38 | 0.61 |
| p53 (Ab-378) | 73 | 65 | 140.5 | 128 | 134.25 | 69.75 | 68.17 | 68.96 | 1.12 | 0.02 |
| p53 (Ab-387) | 142 | 152 | 140.5 | 128 | 134.25 | 135.68 | 159.42 | 147.55 | 16.79 | 0.11 |
| p53 (Ab-392) | 294 | 125 | 140.5 | 128 | 134.25 | 280.92 | 131.10 | 206.01 | 105.94 | 0.51 |
| p53 (Ab-46) | 127 | 95 | 140.5 | 128 | 134.25 | 121.35 | 99.64 | 110.49 | 15.35 | 0.14 |
| p53 (Ab-6) | 102 | 96 | 140.5 | 128 | 134.25 | 97.46 | 100.69 | 99.08 | 2.28 | 0.02 |
| p53 (Ab-9) | 166 | 162 | 140.5 | 128 | 134.25 | 158.62 | 169.91 | 164.26 | 7.99 | 0.05 |
| p53 (Phospho-Ser15) | 168 | 179 | 140.5 | 128 | 134.25 | 160.53 | 187.74 | 174.13 | 19.24 | 0.11 |
| p53 (Phospho-Ser20) | 143 | 120 | 140.5 | 128 | 134.25 | 136.64 | 125.86 | 131.25 | 7.62 | 0.06 |
| p53 (Phospho-Ser315) | 220 | 206 | 140.5 | 128 | 134.25 | 210.21 | 216.06 | 213.14 | 4.13 | 0.02 |
| p53 (Phospho-Ser33) | 121 | 173 | 140.5 | 128 | 134.25 | 115.62 | 181.45 | 148.53 | 46.55 | 0.31 |
| p53 (Phospho-Ser366) | 367 | 367 | 140.5 | 128 | 134.25 | 350.67 | 384.92 | 367.80 | 24.22 | 0.07 |
| p53 (Phospho-Ser37) | 217 | 180 | 140.5 | 128 | 134.25 | 207.35 | 188.79 | 198.07 | 13.12 | 0.07 |
| p53 (Phospho-Ser378) | 76 | 393 | 140.5 | 128 | 134.25 | 72.62 | 412.19 | 242.40 | 240.11 | 0.99 |
| p53 (Phospho-Ser392) | 79 | 170 | 140.5 | 128 | 134.25 | 75.49 | 178.30 | 126.89 | 72.70 | 0.57 |
| p53 (Phospho-Ser46) | 302 | 335 | 140.5 | 128 | 134.25 | 288.57 | 351.36 | 319.96 | 44.40 | 0.14 |
| p53 (Phospho-Ser6) | 124 | 162 | 140.5 | 128 | 134.25 | 118.48 | 169.91 | 144.20 | 36.36 | 0.25 |
| p53 (Phospho-Ser9) | 92 | 501 | 140.5 | 128 | 134.25 | 87.91 | 525.46 | 306.69 | 309.40 | 1.01 |
| p53 (Phospho-Thr18) | 215 | 166 | 140.5 | 128 | 134.25 | 205.44 | 174.11 | 189.77 | 22.15 | 0.12 |
| p53 (Phospho-Thr81) | 199 | 144 | 140.5 | 128 | 134.25 | 190.15 | 151.03 | 170.59 | 27.66 | 0.16 |
| p63 (Phospho-Ser455) | 92 | 167 | 140.5 | 128 | 134.25 | 87.91 | 175.15 | 131.53 | 61.69 | 0.47 |
| P70S6K (Ab-229) | 227 | 166 | 140.5 | 128 | 134.25 | 216.90 | 174.11 | 195.50 | 30.26 | 0.15 |
| P70S6K (Ab-371) | 234 | 93 | 140.5 | 128 | 134.25 | 223.59 | 97.54 | 160.57 | 89.13 | 0.56 |
| P70S6K (Ab-411) | 85 | 102 | 140.5 | 128 | 134.25 | 81.22 | 106.98 | 94.10 | 18.22 | 0.19 |
| P70S6K (Ab-418) | 79 | 71 | 140.5 | 128 | 134.25 | 75.49 | 74.47 | 74.98 | 0.72 | 0.01 |
| P70S6K (Ab-421) | 122 | 106 | 140.5 | 128 | 134.25 | 116.57 | 111.18 | 113.87 | 3.82 | 0.03 |
| P70S6K (Ab-424) | 105 | 99 | 140.5 | 128 | 134.25 | 100.33 | 103.83 | 102.08 | 2.48 | 0.02 |
| P70S6K (Ab-427) | 81 | 84 | 140.5 | 128 | 134.25 | 77.40 | 88.10 | 82.75 | 7.57 | 0.09 |
| P70S6K (Phospho-Ser371) | 409 | 99 | 140.5 | 128 | 134.25 | 390.81 | 103.83 | 247.32 | 202.92 | 0.82 |
| P70S6K (Phospho-Ser411) | 84 | 75 | 140.5 | 128 | 134.25 | 80.26 | 78.66 | 79.46 | 1.13 | 0.01 |
| P70S6K (Phospho-Ser418) | 167 | 80 | 140.5 | 128 | 134.25 | 159.57 | 83.91 | 121.74 | 53.50 | 0.44 |
| P70S6K (Phospho-Ser424) | 99 | 106 | 140.5 | 128 | 134.25 | 94.60 | 111.18 | 102.89 | 11.72 | 0.11 |
| P70S6K (Phospho-Thr229) | 147 | 157 | 140.5 | 128 | 134.25 | 140.46 | 164.67 | 152.56 | 17.12 | 0.11 |
| P70S6K (Phospho-Thr389) | 147 | 203 | 140.5 | 128 | 134.25 | 140.46 | 212.91 | 176.69 | 51.23 | 0.29 |
| P70S6K (Phospho-Thr421) | 215 | 140 | 140.5 | 128 | 134.25 | 205.44 | 146.84 | 176.14 | 41.44 | 0.24 |
| P70S6K-beta (Ab-423) | 238 | 240 | 140.5 | 128 | 134.25 | 227.41 | 251.72 | 239.57 | 17.19 | 0.07 |
| P70S6K-beta (Phospho-Ser423) | 378 | 343 | 140.5 | 128 | 134.25 | 361.19 | 359.75 | 360.47 | 1.02 | 0.00 |
| P73 (Ab-99) | 255 | 228 | 140.5 | 128 | 134.25 | 243.66 | 239.13 | 241.39 | 3.20 | 0.01 |
| P73 (Phospho-Tyr99) | 139 | 132 | 140.5 | 128 | 134.25 | 132.82 | 138.45 | 135.63 | 3.98 | 0.03 |
| P90RSK (Ab-359/363) | 4383 | 3523 | 140.5 | 128 | 134.25 | 4188.03 | 3695.02 | 3941.52 | 348.61 | 0.09 |
| P90RSK (Ab-380) | 104 | 104 | 140.5 | 128 | 134.25 | 99.37 | 109.08 | 104.23 | 6.86 | 0.07 |
| P90RSK (Ab-573) | 953 | 478 | 140.5 | 128 | 134.25 | 910.61 | 501.34 | 705.97 | 289.40 | 0.41 |
| P90RSK (Phospho-Ser380) | 235 | 80 | 140.5 | 128 | 134.25 | 224.55 | 83.91 | 154.23 | 99.45 | 0.64 |
| P90RSK (Phospho-Thr359/Ser363) | 119 | 331 | 140.5 | 128 | 134.25 | 113.71 | 347.16 | 230.43 | 165.08 | 0.72 |
| P90RSK (Phospho-Thr573) | 93 | 349 | 140.5 | 128 | 134.25 | 88.86 | 366.04 | 227.45 | 195.99 | 0.86 |
| P95/NBS1 (Ab-343) | 587 | 572 | 140.5 | 128 | 134.25 | 560.89 | 599.93 | 580.41 | 27.61 | 0.05 |
| P95/NBS1 (Phospho-Ser343) | 108 | 298 | 140.5 | 128 | 134.25 | 103.20 | 312.55 | 207.87 | 148.04 | 0.71 |
| PAK1 (Ab-204) | 101 | 102 | 140.5 | 128 | 134.25 | 96.51 | 106.98 | 101.74 | 7.41 | 0.07 |
| PAK1 (Ab-212) | 165 | 143 | 140.5 | 128 | 134.25 | 157.66 | 149.98 | 153.82 | 5.43 | 0.04 |
| PAK1 (Phospho-Ser204) | 312 | 324 | 140.5 | 128 | 134.25 | 298.12 | 339.82 | 318.97 | 29.49 | 0.09 |
| PAK1 (Phospho-Thr212) | 275 | 301 | 140.5 | 128 | 134.25 | 262.77 | 315.70 | 289.23 | 37.43 | 0.13 |
| PAK1/2 (Ab-199) | 105 | 90 | 140.5 | 128 | 134.25 | 100.33 | 94.39 | 97.36 | 4.20 | 0.04 |
| PAK1/2 (Phospho-Ser199) | 81 | 145 | 140.5 | 128 | 134.25 | 77.40 | 152.08 | 114.74 | 52.81 | 0.46 |
| PAK1/2/3 (Ab-141) | 90 | 85 | 140.5 | 128 | 134.25 | 86.00 | 89.15 | 87.57 | 2.23 | 0.03 |
| PAK1/2/3 (Ab-423/402/421) | 4933 | 4442 | 140.5 | 128 | 134.25 | 4713.56 | 4658.89 | 4686.23 | 38.65 | 0.01 |
| PAK1/2/3 (Phospho-Ser141) | 79 | 340 | 140.5 | 128 | 134.25 | 75.49 | 356.60 | 216.04 | 198.78 | 0.92 |
| PAK1/2/3 (Phospho-Thr423/402/421) | 291 | 249 | 140.5 | 128 | 134.25 | 278.06 | 261.16 | 269.61 | 11.95 | 0.04 |
| PAK2 (Ab-192) | 76 | 74 | 140.5 | 128 | 134.25 | 72.62 | 77.61 | 75.12 | 3.53 | 0.05 |
| PAK2 (Ab-197) | 182 | 184 | 140.5 | 128 | 134.25 | 173.90 | 192.98 | 183.44 | 13.49 | 0.07 |
| PAK2 (Phospho-Ser192) | 336 | 88 | 140.5 | 128 | 134.25 | 321.05 | 92.30 | 206.68 | 161.76 | 0.78 |
| PAK2 (Phospho-Ser20) | 91 | 90 | 140.5 | 128 | 134.25 | 86.95 | 94.39 | 90.67 | 5.26 | 0.06 |
| PAK3 (Ab-154) | 101 | 92 | 140.5 | 128 | 134.25 | 96.51 | 96.49 | 96.50 | 0.01 | 0.00 |
| PAK3 (Phospho-Ser154) | 90 | 288 | 140.5 | 128 | 134.25 | 86.00 | 302.06 | 194.03 | 152.78 | 0.79 |
| PAK4 (Ab-474) | 389 | 335 | 140.5 | 128 | 134.25 | 371.70 | 351.36 | 361.53 | 14.38 | 0.04 |
| Paxillin (Ab-118) | 100 | 92 | 140.5 | 128 | 134.25 | 95.55 | 96.49 | 96.02 | 0.67 | 0.01 |
| Paxillin (Ab-31) | 106 | 93 | 140.5 | 128 | 134.25 | 101.28 | 97.54 | 99.41 | 2.65 | 0.03 |
| Paxillin (Phospho-Tyr118) | 169 | 339 | 140.5 | 128 | 134.25 | 161.48 | 355.55 | 258.52 | 137.23 | 0.53 |
| Paxillin (Phospho-Tyr31) | 117 | 97 | 140.5 | 128 | 134.25 | 111.80 | 101.74 | 106.77 | 7.11 | 0.07 |
| PDGFR alpha (Ab-849) | 294 | 74 | 140.5 | 128 | 134.25 | 280.92 | 77.61 | 179.27 | 143.76 | 0.80 |
| PDGFR alpha (Phospho-Tyr849) | 97 | 101 | 140.5 | 128 | 134.25 | 92.69 | 105.93 | 99.31 | 9.37 | 0.09 |
| PDGFR beta (Ab-1009) | 102 | 91 | 140.5 | 128 | 134.25 | 97.46 | 95.44 | 96.45 | 1.43 | 0.01 |
| PDGFR beta (Ab-1021) | 116 | 92 | 140.5 | 128 | 134.25 | 110.84 | 96.49 | 103.67 | 10.15 | 0.10 |
| PDGFR beta (Ab-740) | 139 | 126 | 140.5 | 128 | 134.25 | 132.82 | 132.15 | 132.48 | 0.47 | 0.00 |
| PDGFR beta (Ab-751) | 299 | 171 | 140.5 | 128 | 134.25 | 285.70 | 179.35 | 232.52 | 75.20 | 0.32 |
| PDGFR beta (Phospho-Tyr1021) | 280 | 268 | 140.5 | 128 | 134.25 | 267.54 | 281.09 | 274.32 | 9.58 | 0.03 |
| PDGFR beta (Phospho-Tyr740) | 184 | 182 | 140.5 | 128 | 134.25 | 175.81 | 190.89 | 183.35 | 10.66 | 0.06 |
| PDGFR beta (Phospho-Tyr751) | 123 | 107 | 140.5 | 128 | 134.25 | 117.53 | 112.22 | 114.88 | 3.75 | 0.03 |
| PDK1 (Ab-241) | 1588 | 1376 | 140.5 | 128 | 134.25 | 1517.36 | 1443.19 | 1480.27 | 52.45 | 0.04 |
| PDK1 (Phospho-Ser241) | 612 | 549 | 140.5 | 128 | 134.25 | 584.78 | 575.81 | 580.29 | 6.34 | 0.01 |
| PEA-15 (Ab-116) | 75 | 67 | 140.5 | 128 | 134.25 | 71.66 | 70.27 | 70.97 | 0.98 | 0.01 |
| PEA-15 (Phospho-Ser104) | 158 | 108 | 140.5 | 128 | 134.25 | 150.97 | 113.27 | 132.12 | 26.66 | 0.20 |
| PEA-15 (Phospho-Ser116) | 94 | 86 | 140.5 | 128 | 134.25 | 89.82 | 90.20 | 90.01 | 0.27 | 0.00 |
| PECAM-1 (Ab-713) | 2834 | 2535 | 140.5 | 128 | 134.25 | 2707.93 | 2658.78 | 2683.36 | 34.76 | 0.01 |
| PECAM-1 (Phospho-Tyr713) | 160 | 152 | 140.5 | 128 | 134.25 | 152.88 | 159.42 | 156.15 | 4.62 | 0.03 |
| PI3-kinase p85-alpha (Phospho-Tyr607) | 82 | 64 | 140.5 | 128 | 134.25 | 78.35 | 67.13 | 72.74 | 7.94 | 0.11 |
| PI3-kinase p85-subunit alpha/gamma (Ab-467/199) | 102 | 83 | 140.5 | 128 | 134.25 | 97.46 | 87.05 | 92.26 | 7.36 | 0.08 |
| PI3-kinase p85-subunit alpha/gamma (Phospho-Tyr467/Tyr199) | 621 | 535 | 140.5 | 128 | 134.25 | 593.38 | 561.12 | 577.25 | 22.81 | 0.04 |
| Pim-1 (Ab-309) | 132 | 111 | 140.5 | 128 | 134.25 | 126.13 | 116.42 | 121.27 | 6.86 | 0.06 |
| Pim-1 (Phospho-Tyr309) | 110 | 79 | 140.5 | 128 | 134.25 | 105.11 | 82.86 | 93.98 | 15.73 | 0.17 |
| PIP5K (Phospho-Ser307) | 77 | 73 | 140.5 | 128 | 134.25 | 73.57 | 76.56 | 75.07 | 2.11 | 0.03 |
| PKA CAT (Ab-197) | 123 | 123 | 140.5 | 128 | 134.25 | 117.53 | 129.01 | 123.27 | 8.12 | 0.07 |
| PKA CAT (Phospho-Thr197) | 126 | 107 | 140.5 | 128 | 134.25 | 120.40 | 112.22 | 116.31 | 5.78 | 0.05 |
| PKA-R2B (Phospho-Ser113) | 298 | 204 | 140.5 | 128 | 134.25 | 284.74 | 213.96 | 249.35 | 50.05 | 0.20 |
| PKC alpha (Ab-657) | 91 | 65 | 140.5 | 128 | 134.25 | 86.95 | 68.17 | 77.56 | 13.28 | 0.17 |
| PKC alpha (Phospho-Tyr657) | 238 | 203 | 140.5 | 128 | 134.25 | 227.41 | 212.91 | 220.16 | 10.25 | 0.05 |
| PKC alpha/beta II (Ab-638) | 97 | 73 | 140.5 | 128 | 134.25 | 92.69 | 76.56 | 84.62 | 11.40 | 0.13 |
| PKC alpha/beta II (Phospho-Thr638) | 76 | 68 | 140.5 | 128 | 134.25 | 72.62 | 71.32 | 71.97 | 0.92 | 0.01 |
| PKC beta/PKCB (Ab-661) | 101 | 95 | 140.5 | 128 | 134.25 | 96.51 | 99.64 | 98.07 | 2.21 | 0.02 |
| PKC beta/PKCB (Phospho-Ser661) | 149 | 153 | 140.5 | 128 | 134.25 | 142.37 | 160.47 | 151.42 | 12.80 | 0.08 |
| PKC delta (Ab-505) | 119 | 86 | 140.5 | 128 | 134.25 | 113.71 | 90.20 | 101.95 | 16.62 | 0.16 |
| PKC delta (Ab-645) | 394 | 373 | 140.5 | 128 | 134.25 | 376.47 | 391.21 | 383.84 | 10.42 | 0.03 |
| PKC delta (Phospho-Ser645) | 476 | 81 | 140.5 | 128 | 134.25 | 454.83 | 84.96 | 269.89 | 261.54 | 0.97 |
| PKC delta (Phospho-Thr505) | 101 | 95 | 140.5 | 128 | 134.25 | 96.51 | 99.64 | 98.07 | 2.21 | 0.02 |
| PKC delta (Phospho-Tyr313) | 96 | 82 | 140.5 | 128 | 134.25 | 91.73 | 86.00 | 88.87 | 4.05 | 0.05 |
| PKC delta (Phospho-Tyr52) | 169 | 139 | 140.5 | 128 | 134.25 | 161.48 | 145.79 | 153.63 | 11.10 | 0.07 |
| PKC delta (Phospho-Tyr64) | 108 | 102 | 140.5 | 128 | 134.25 | 103.20 | 106.98 | 105.09 | 2.68 | 0.03 |
| PKC epsilon (Ab-729) | 100 | 81 | 140.5 | 128 | 134.25 | 95.55 | 84.96 | 90.25 | 7.49 | 0.08 |
| PKC epsilon (Phospho-Ser729) | 160 | 66 | 140.5 | 128 | 134.25 | 152.88 | 69.22 | 111.05 | 59.16 | 0.53 |
| PKC pan activation site | 160 | 113 | 140.5 | 128 | 134.25 | 152.88 | 118.52 | 135.70 | 24.30 | 0.18 |
| PKC pan activation site (Phospho) | 208 | 180 | 140.5 | 128 | 134.25 | 198.75 | 188.79 | 193.77 | 7.04 | 0.04 |
| PKC theta (Ab-676) | 140 | 117 | 140.5 | 128 | 134.25 | 133.77 | 122.71 | 128.24 | 7.82 | 0.06 |
| PKC theta (Phospho-Ser676) | 113 | 106 | 140.5 | 128 | 134.25 | 107.97 | 111.18 | 109.57 | 2.26 | 0.02 |
| PKC theta (Phospho-Thr538) | 73 | 74 | 140.5 | 128 | 134.25 | 69.75 | 77.61 | 73.68 | 5.56 | 0.08 |
| PKC zeta (Ab-410) | 216 | 182 | 140.5 | 128 | 134.25 | 206.39 | 190.89 | 198.64 | 10.96 | 0.06 |
| PKC zeta (Ab-560) | 98 | 102 | 140.5 | 128 | 134.25 | 93.64 | 106.98 | 100.31 | 9.43 | 0.09 |
| PKC zeta (Phospho-Thr410) | 144 | 171 | 140.5 | 128 | 134.25 | 137.59 | 179.35 | 158.47 | 29.53 | 0.19 |
| PKC zeta (Phospho-Thr560) | 200 | 75 | 140.5 | 128 | 134.25 | 191.10 | 78.66 | 134.88 | 79.51 | 0.59 |
| PKD1/PKC mu (Ab-205) | 116 | 121 | 140.5 | 128 | 134.25 | 110.84 | 126.91 | 118.87 | 11.36 | 0.10 |
| PKD1/PKC mu (Ab-463) | 245 | 247 | 140.5 | 128 | 134.25 | 234.10 | 259.06 | 246.58 | 17.65 | 0.07 |
| PKD1/PKC mu (Ab-744/748) | 228 | 159 | 140.5 | 128 | 134.25 | 217.86 | 166.76 | 192.31 | 36.13 | 0.19 |
| PKD1/PKC mu (Ab-910) | 104 | 87 | 140.5 | 128 | 134.25 | 99.37 | 91.25 | 95.31 | 5.75 | 0.06 |
| PKD1/PKC mu (Phospho-Ser205) | 449 | 76 | 140.5 | 128 | 134.25 | 429.03 | 79.71 | 254.37 | 247.00 | 0.97 |
| PKD1/PKC mu (Phospho-Ser910) | 115 | 63 | 140.5 | 128 | 134.25 | 109.88 | 66.08 | 87.98 | 30.98 | 0.35 |
| PKD1/PKC mu (Phospho-Tyr463) | 127 | 134 | 140.5 | 128 | 134.25 | 121.35 | 140.54 | 130.95 | 13.57 | 0.10 |
| PKD2 (Ab-876) | 75 | 77 | 140.5 | 128 | 134.25 | 71.66 | 80.76 | 76.21 | 6.43 | 0.08 |
| PKD2 (Phospho-Ser876) | 163 | 178 | 140.5 | 128 | 134.25 | 155.75 | 186.69 | 171.22 | 21.88 | 0.13 |
| PKR (Ab-446) | 86 | 84 | 140.5 | 128 | 134.25 | 82.17 | 88.10 | 85.14 | 4.19 | 0.05 |
| PKR (Ab-451) | 128 | 119 | 140.5 | 128 | 134.25 | 122.31 | 124.81 | 123.56 | 1.77 | 0.01 |
| PKR (Phospho-Thr446) | 98 | 91 | 140.5 | 128 | 134.25 | 93.64 | 95.44 | 94.54 | 1.27 | 0.01 |
| PKR (Phospho-Thr451) | 144 | 497 | 140.5 | 128 | 134.25 | 137.59 | 521.27 | 329.43 | 271.30 | 0.82 |
| PLC beta3 (Ab-1105) | 230 | 191 | 140.5 | 128 | 134.25 | 219.77 | 200.33 | 210.05 | 13.75 | 0.07 |
| PLC beta3 (Ab-537) | 86 | 77 | 140.5 | 128 | 134.25 | 82.17 | 80.76 | 81.47 | 1.00 | 0.01 |
| PLC beta3 (Phospho-Ser1105) | 92 | 75 | 140.5 | 128 | 134.25 | 87.91 | 78.66 | 83.28 | 6.54 | 0.08 |
| PLC beta3 (Phospho-Ser537) | 93 | 77 | 140.5 | 128 | 134.25 | 88.86 | 80.76 | 84.81 | 5.73 | 0.07 |
| PLCG1 (Ab-771) | 80 | 102 | 140.5 | 128 | 134.25 | 76.44 | 106.98 | 91.71 | 21.59 | 0.24 |
| PLCG1 (Ab-783) | 96 | 92 | 140.5 | 128 | 134.25 | 91.73 | 96.49 | 94.11 | 3.37 | 0.04 |
| PLCG1 (Phospho-Tyr1253) | 203 | 81 | 140.5 | 128 | 134.25 | 193.97 | 84.96 | 139.46 | 77.09 | 0.55 |
| PLCG1 (Phospho-Tyr771) | 83 | 83 | 140.5 | 128 | 134.25 | 79.31 | 87.05 | 83.18 | 5.48 | 0.07 |
| PLCG1 (Phospho-Tyr783) | 175 | 192 | 140.5 | 128 | 134.25 | 167.22 | 201.38 | 184.30 | 24.15 | 0.13 |
| PLCG2 (Ab-1217) | 174 | 167 | 140.5 | 128 | 134.25 | 166.26 | 175.15 | 170.71 | 6.29 | 0.04 |
| PLCG2 (Ab-753) | 179 | 229 | 140.5 | 128 | 134.25 | 171.04 | 240.18 | 205.61 | 48.89 | 0.24 |
| PLCG2 (Phospho-Tyr1217) | 121 | 131 | 140.5 | 128 | 134.25 | 115.62 | 137.40 | 126.51 | 15.40 | 0.12 |
| PLCG2 (Phospho-Tyr753) | 78 | 83 | 140.5 | 128 | 134.25 | 74.53 | 87.05 | 80.79 | 8.85 | 0.11 |
| PLD1 (Ab-561) | 110 | 109 | 140.5 | 128 | 134.25 | 105.11 | 114.32 | 109.71 | 6.52 | 0.06 |
| PLD1 (Phospho-Ser561) | 356 | 68 | 140.5 | 128 | 134.25 | 340.16 | 71.32 | 205.74 | 190.10 | 0.92 |
| PLD2 (Phospho-Tyr169) | 154 | 143 | 140.5 | 128 | 134.25 | 147.15 | 149.98 | 148.57 | 2.00 | 0.01 |
| PLK1 (Ab-210) | 216 | 215 | 140.5 | 128 | 134.25 | 206.39 | 225.50 | 215.94 | 13.51 | 0.06 |
| PLK1 (Phospho-Thr210) | 62 | 59 | 140.5 | 128 | 134.25 | 59.24 | 61.88 | 60.56 | 1.87 | 0.03 |
| PP1 alpha (Ab-320) | 149 | 156 | 140.5 | 128 | 134.25 | 142.37 | 163.62 | 152.99 | 15.02 | 0.10 |
| PP1 alpha (Phospho-Thr320) | 199 | 71 | 140.5 | 128 | 134.25 | 190.15 | 74.47 | 132.31 | 81.80 | 0.62 |
| PP2A-alpha (Ab-307) | 814 | 1121 | 140.5 | 128 | 134.25 | 777.79 | 1175.74 | 976.76 | 281.39 | 0.29 |
| PP2A-alpha (Phospho-Tyr307) | 97 |  | 140.5 | 128 | 134.25 | 92.69 |  | 92.69 |  |  |
| PPAR-BP (Ab-1457) | 125 | 191 | 140.5 | 128 | 134.25 | 119.44 | 200.33 | 159.88 | 57.20 | 0.36 |
| PPAR-BP (Phospho-Thr1457) | 142 | 76 | 140.5 | 128 | 134.25 | 135.68 | 79.71 | 107.70 | 39.58 | 0.37 |
| PPAR-gamma (Ab-112) | 2951 | 3139 | 140.5 | 128 | 134.25 | 2819.73 | 3292.27 | 3056.00 | 334.14 | 0.11 |
| PPAR-gamma (Phospho-Ser112) | 89 | 82 | 140.5 | 128 | 134.25 | 85.04 | 86.00 | 85.52 | 0.68 | 0.01 |
| Progesterone Receptor (Ab-190) | 354 | 283 | 140.5 | 128 | 134.25 | 338.25 | 296.82 | 317.54 | 29.30 | 0.09 |
| Progesterone Receptor (Phospho-Ser190) | 115 | 98 | 140.5 | 128 | 134.25 | 109.88 | 102.79 | 106.33 | 5.02 | 0.05 |
| PTEN (Ab-370) | 591 | 560 | 140.5 | 128 | 134.25 | 564.71 | 587.34 | 576.03 | 16.00 | 0.03 |
| PTEN (Ab-380) | 806 | 721 | 140.5 | 128 | 134.25 | 770.15 | 756.21 | 763.18 | 9.86 | 0.01 |
| PTEN (Ab-380/382/383) | 463 | 435 | 140.5 | 128 | 134.25 | 442.40 | 456.24 | 449.32 | 9.78 | 0.02 |
| PTEN (Phospho-Ser370) | 128 | 128 | 140.5 | 128 | 134.25 | 122.31 | 134.25 | 128.28 | 8.45 | 0.07 |
| PTEN (Phospho-Ser380) | 145 | 131 | 140.5 | 128 | 134.25 | 138.55 | 137.40 | 137.97 | 0.82 | 0.01 |
| PTEN (Phospho-Ser380/Thr382/Thr383) | 144 | 165 | 140.5 | 128 | 134.25 | 137.59 | 173.06 | 155.33 | 25.08 | 0.16 |
| PTPRA (Phospho-Tyr798) | 123 | 104 | 140.5 | 128 | 134.25 | 117.53 | 109.08 | 113.30 | 5.98 | 0.05 |
| Pyk2 (Ab-402) | 100 | 85 | 140.5 | 128 | 134.25 | 95.55 | 89.15 | 92.35 | 4.53 | 0.05 |
| Pyk2 (Ab-580) | 943 | 920 | 140.5 | 128 | 134.25 | 901.05 | 964.92 | 932.99 | 45.16 | 0.05 |
| Pyk2 (Ab-881) | 539 | 456 | 140.5 | 128 | 134.25 | 515.02 | 478.27 | 496.64 | 25.99 | 0.05 |
| Pyk2 (Phospho-Tyr402) | 145 | 368 | 140.5 | 128 | 134.25 | 138.55 | 385.97 | 262.26 | 174.95 | 0.67 |
| Pyk2 (Phospho-Tyr579) | 163 | 152 | 140.5 | 128 | 134.25 | 155.75 | 159.42 | 157.59 | 2.60 | 0.02 |
| Pyk2 (Phospho-Tyr580) | 113 | 108 | 140.5 | 128 | 134.25 | 107.97 | 113.27 | 110.62 | 3.75 | 0.03 |
| Pyk2 (Phospho-Tyr881) | 258 | 267 | 140.5 | 128 | 134.25 | 246.52 | 280.04 | 263.28 | 23.70 | 0.09 |
| Rac1/cdc42 (Ab-71) | 143 | 120 | 140.5 | 128 | 134.25 | 136.64 | 125.86 | 131.25 | 7.62 | 0.06 |
| Rac1/cdc42 (Phospho-Ser71) | 103 |  | 140.5 | 128 | 134.25 | 98.42 |  | 98.42 |  |  |
| RAD51 (Ab-309) | 121 | 74 | 140.5 | 128 | 134.25 | 115.62 | 77.61 | 96.62 | 26.87 | 0.28 |
| RAD51 (Phospho-Tyr315) | 149 | 193 | 140.5 | 128 | 134.25 | 142.37 | 202.42 | 172.40 | 42.46 | 0.25 |
| RAD52 (Phospho-Tyr104) | 163 | 156 | 140.5 | 128 | 134.25 | 155.75 | 163.62 | 159.68 | 5.56 | 0.03 |
| Raf1 (Ab-259) | 387 | 212 | 140.5 | 128 | 134.25 | 369.78 | 222.35 | 296.07 | 104.25 | 0.35 |
| Raf1 (Ab-289) | 303 | 331 | 140.5 | 128 | 134.25 | 289.52 | 347.16 | 318.34 | 40.76 | 0.13 |
| Raf1 (Ab-296) | 204 | 132 | 140.5 | 128 | 134.25 | 194.93 | 138.45 | 166.69 | 39.94 | 0.24 |
| Raf1 (Ab-338) | 164 | 141 | 140.5 | 128 | 134.25 | 156.70 | 147.88 | 152.29 | 6.24 | 0.04 |
| Raf1 (Ab-341) | 192 | 168 | 140.5 | 128 | 134.25 | 183.46 | 176.20 | 179.83 | 5.13 | 0.03 |
| Raf1 (Ab-43) | 156 | 179 | 140.5 | 128 | 134.25 | 149.06 | 187.74 | 168.40 | 27.35 | 0.16 |
| Raf1 (Ab-621) | 137 | 187 | 140.5 | 128 | 134.25 | 130.91 | 196.13 | 163.52 | 46.12 | 0.28 |
| Raf1 (Phospho-Ser259) | 339 | 299 | 140.5 | 128 | 134.25 | 323.92 | 313.60 | 318.76 | 7.30 | 0.02 |
| Raf1 (Phospho-Ser289) | 74 | 68 | 140.5 | 128 | 134.25 | 70.71 | 71.32 | 71.01 | 0.43 | 0.01 |
| Raf1 (Phospho-Ser296) | 97 | 85 | 140.5 | 128 | 134.25 | 92.69 | 89.15 | 90.92 | 2.50 | 0.03 |
| Raf1 (Phospho-Ser338) | 98 | 100 | 140.5 | 128 | 134.25 | 93.64 | 104.88 | 99.26 | 7.95 | 0.08 |
| Raf1 (Phospho-Ser43) | 250 | 195 | 140.5 | 128 | 134.25 | 238.88 | 204.52 | 221.70 | 24.29 | 0.11 |
| Raf1 (Phospho-Ser621) | 243 | 255 | 140.5 | 128 | 134.25 | 232.19 | 267.45 | 249.82 | 24.93 | 0.10 |
| Raf1 (Phospho-Tyr341) | 153 | 105 | 140.5 | 128 | 134.25 | 146.19 | 110.13 | 128.16 | 25.50 | 0.20 |
| RapGEF1 (Phospho-Tyr504) | 79 | 71 | 140.5 | 128 | 134.25 | 75.49 | 74.47 | 74.98 | 0.72 | 0.01 |
| Ras-GRF1 (Ab-916) | 381 | 491 | 140.5 | 128 | 134.25 | 364.05 | 514.97 | 439.51 | 106.72 | 0.24 |
| Ras-GRF1 (Phospho-Ser916) | 89 | 81 | 140.5 | 128 | 134.25 | 85.04 | 84.96 | 85.00 | 0.06 | 0.00 |
| Rb (Ab-608) | 129 | 116 | 140.5 | 128 | 134.25 | 123.26 | 121.66 | 122.46 | 1.13 | 0.01 |
| Rb (Ab-780) | 303 | 288 | 140.5 | 128 | 134.25 | 289.52 | 302.06 | 295.79 | 8.87 | 0.03 |
| Rb (Ab-795) | 287 | 266 | 140.5 | 128 | 134.25 | 274.23 | 278.99 | 276.61 | 3.36 | 0.01 |
| Rb (Ab-807) | 656 | 570 | 140.5 | 128 | 134.25 | 626.82 | 597.83 | 612.33 | 20.50 | 0.03 |
| Rb (Ab-811) | 162 | 193 | 140.5 | 128 | 134.25 | 154.79 | 202.42 | 178.61 | 33.68 | 0.19 |
| Rb (Phospho-Ser608) | 89 | 84 | 140.5 | 128 | 134.25 | 85.04 | 88.10 | 86.57 | 2.16 | 0.02 |
| Rb (Phospho-Ser780) | 89 | 76 | 140.5 | 128 | 134.25 | 85.04 | 79.71 | 82.38 | 3.77 | 0.05 |
| Rb (Phospho-Ser795) | 174 | 144 | 140.5 | 128 | 134.25 | 166.26 | 151.03 | 158.65 | 10.77 | 0.07 |
| Rb (Phospho-Ser807) | 111 | 290 | 140.5 | 128 | 134.25 | 106.06 | 304.16 | 205.11 | 140.08 | 0.68 |
| Rb (Phospho-Ser811) | 77 | 159 | 140.5 | 128 | 134.25 | 73.57 | 166.76 | 120.17 | 65.89 | 0.55 |
| Rb (Phospho-Thr821) | 75 | 79 | 140.5 | 128 | 134.25 | 71.66 | 82.86 | 77.26 | 7.92 | 0.10 |
| Rb-like-2 (RBL2) (Ab-952) | 79 | 83 | 140.5 | 128 | 134.25 | 75.49 | 87.05 | 81.27 | 8.18 | 0.10 |
| Rel (Ab-503) | 178 | 150 | 140.5 | 128 | 134.25 | 170.08 | 157.32 | 163.70 | 9.02 | 0.06 |
| Rel (Phospho-Ser503) | 117 | 613 | 140.5 | 128 | 134.25 | 111.80 | 642.93 | 377.36 | 375.57 | 1.00 |
| RelB (Ab-552) | 152 | 110 | 140.5 | 128 | 134.25 | 145.24 | 115.37 | 130.30 | 21.12 | 0.16 |
| RelB (Phospho-Ser552) | 289 | 438 | 140.5 | 128 | 134.25 | 276.14 | 459.39 | 367.77 | 129.57 | 0.35 |
| Ret (Ab-905) | 102 | 105 | 140.5 | 128 | 134.25 | 97.46 | 110.13 | 103.79 | 8.96 | 0.09 |
| Ret (Phospho-Tyr905) | 114 | 105 | 140.5 | 128 | 134.25 | 108.93 | 110.13 | 109.53 | 0.85 | 0.01 |
| RGS16 (Phospho-Tyr168) | 117 | 71 | 140.5 | 128 | 134.25 | 111.80 | 74.47 | 93.13 | 26.40 | 0.28 |
| Rho/Rac guanine nucleotide exchange factor 2 (Ab-885) | 71 | 62 | 140.5 | 128 | 134.25 | 67.84 | 65.03 | 66.43 | 1.99 | 0.03 |
| Rho/Rac guanine nucleotide exchange factor 2 (Phospho-Ser885) | 154 | 184 | 140.5 | 128 | 134.25 | 147.15 | 192.98 | 170.07 | 32.41 | 0.19 |
| RhoA (Ab-188) | 596 | 518 | 140.5 | 128 | 134.25 | 569.49 | 543.29 | 556.39 | 18.52 | 0.03 |
| RSK1/2/3/4 (Ab-221/227/218/232) | 257 | 111 | 140.5 | 128 | 134.25 | 245.57 | 116.42 | 180.99 | 91.32 | 0.50 |
| RSK1/2/3/4 (Phospho-Ser221/227/218/232) | 89 | 83 | 140.5 | 128 | 134.25 | 85.04 | 87.05 | 86.05 | 1.42 | 0.02 |
| RyR2 (Ab-2808) | 170 | 253 | 140.5 | 128 | 134.25 | 162.44 | 265.35 | 213.90 | 72.77 | 0.34 |
| RyR2 (Phospho-Ser2808) | 322 | 71 | 140.5 | 128 | 134.25 | 307.68 | 74.47 | 191.07 | 164.90 | 0.86 |
| S6 Ribosomal Protein (Ab-235) | 111 | 87 | 140.5 | 128 | 134.25 | 106.06 | 91.25 | 98.66 | 10.48 | 0.11 |
| S6 Ribosomal Protein (Phospho-Ser235) | 88 | 95 | 140.5 | 128 | 134.25 | 84.09 | 99.64 | 91.86 | 11.00 | 0.12 |
| SAPK/JNK (Ab-183) | 116 | 81 | 140.5 | 128 | 134.25 | 110.84 | 84.96 | 97.90 | 18.30 | 0.19 |
| SAPK/JNK (Ab-185) | 127 | 106 | 140.5 | 128 | 134.25 | 121.35 | 111.18 | 116.26 | 7.19 | 0.06 |
| SAPK/JNK (Phospho-Thr183) | 288 | 187 | 140.5 | 128 | 134.25 | 275.19 | 196.13 | 235.66 | 55.90 | 0.24 |
| SAPK/JNK (Phospho-Tyr185) | 101 | 82 | 140.5 | 128 | 134.25 | 96.51 | 86.00 | 91.26 | 7.43 | 0.08 |
| Shc (Ab-349) | 1040 | 818 | 140.5 | 128 | 134.25 | 993.74 | 857.94 | 925.84 | 96.02 | 0.10 |
| Shc (Ab-427) | 540 | 491 | 140.5 | 128 | 134.25 | 515.98 | 514.97 | 515.48 | 0.71 | 0.00 |
| Shc (Phospho-Tyr349) | 136 | 133 | 140.5 | 128 | 134.25 | 129.95 | 139.49 | 134.72 | 6.75 | 0.05 |
| Shc (Phospho-Tyr427) | 115 | 100 | 140.5 | 128 | 134.25 | 109.88 | 104.88 | 107.38 | 3.54 | 0.03 |
| SHP-1 (Phospho-Tyr536) | 99 | 526 | 140.5 | 128 | 134.25 | 94.60 | 551.68 | 323.14 | 323.21 | 1.00 |
| SHP-2 (Ab-542) | 112 | 102 | 140.5 | 128 | 134.25 | 107.02 | 106.98 | 107.00 | 0.03 | 0.00 |
| SHP-2 (Ab-580) | 220 | 165 | 140.5 | 128 | 134.25 | 210.21 | 173.06 | 191.64 | 26.27 | 0.14 |
| SHP-2 (Phospho-Tyr542) | 85 | 315 | 140.5 | 128 | 134.25 | 81.22 | 330.38 | 205.80 | 176.18 | 0.86 |
| SHP-2 (Phospho-Tyr580) | 111 | 299 | 140.5 | 128 | 134.25 | 106.06 | 313.60 | 209.83 | 146.75 | 0.70 |
| SLP-76 (Ab-128) | 246 | 196 | 140.5 | 128 | 134.25 | 235.06 | 205.57 | 220.31 | 20.85 | 0.09 |
| SLP-76 (Phospho-Tyr128) | 124 | 135 | 140.5 | 128 | 134.25 | 118.48 | 141.59 | 130.04 | 16.34 | 0.13 |
| Smad1 (Ab-187) | 101 | 100 | 140.5 | 128 | 134.25 | 96.51 | 104.88 | 100.69 | 5.92 | 0.06 |
| Smad1 (Ab-465) | 74 | 70 | 140.5 | 128 | 134.25 | 70.71 | 73.42 | 72.06 | 1.92 | 0.03 |
| Smad1 (Phospho-Ser187) | 93 | 66 | 140.5 | 128 | 134.25 | 88.86 | 69.22 | 79.04 | 13.89 | 0.18 |
| Smad1 (Phospho-Ser465) | 74 | 62 | 140.5 | 128 | 134.25 | 70.71 | 65.03 | 67.87 | 4.02 | 0.06 |
| Smad2 (Ab-220) | 252 | 76 | 140.5 | 128 | 134.25 | 240.79 | 79.71 | 160.25 | 113.90 | 0.71 |
| Smad2 (Ab-245) | 78 | 66 | 140.5 | 128 | 134.25 | 74.53 | 69.22 | 71.88 | 3.75 | 0.05 |
| Smad2 (Ab-250) | 100 | 90 | 140.5 | 128 | 134.25 | 95.55 | 94.39 | 94.97 | 0.82 | 0.01 |
| Smad2 (Ab-255) | 419 | 393 | 140.5 | 128 | 134.25 | 400.36 | 412.19 | 406.28 | 8.36 | 0.02 |
| Smad2 (Ab-467) | 106 | 105 | 140.5 | 128 | 134.25 | 101.28 | 110.13 | 105.71 | 6.25 | 0.06 |
| Smad2 (Phospho-Ser250) | 115 | 69 | 140.5 | 128 | 134.25 | 109.88 | 72.37 | 91.13 | 26.53 | 0.29 |
| Smad2 (Phospho-Ser467) | 292 | 211 | 140.5 | 128 | 134.25 | 279.01 | 221.30 | 250.16 | 40.81 | 0.16 |
| Smad2 (Phospho-Thr220) | 250 | 199 | 140.5 | 128 | 134.25 | 238.88 | 208.72 | 223.80 | 21.33 | 0.10 |
| Smad2/3 (Ab-8) | 256 | 227 | 140.5 | 128 | 134.25 | 244.61 | 238.08 | 241.35 | 4.62 | 0.02 |
| Smad2/3 (Phospho-Thr8) | 545 | 434 | 140.5 | 128 | 134.25 | 520.76 | 455.19 | 487.97 | 46.36 | 0.10 |
| Smad3 (Ab-179) | 205 | 173 | 140.5 | 128 | 134.25 | 195.88 | 181.45 | 188.66 | 10.21 | 0.05 |
| Smad3 (Ab-204) | 142 | 127 | 140.5 | 128 | 134.25 | 135.68 | 133.20 | 134.44 | 1.76 | 0.01 |
| Smad3 (Ab-213) | 73 | 66 | 140.5 | 128 | 134.25 | 69.75 | 69.22 | 69.49 | 0.37 | 0.01 |
| Smad3 (Ab-425) | 102 | 91 | 140.5 | 128 | 134.25 | 97.46 | 95.44 | 96.45 | 1.43 | 0.01 |
| Smad3 (Phospho-Ser204) | 86 | 58 | 140.5 | 128 | 134.25 | 82.17 | 60.83 | 71.50 | 15.09 | 0.21 |
| Smad3 (Phospho-Ser208) | 103 | 72 | 140.5 | 128 | 134.25 | 98.42 | 75.52 | 86.97 | 16.19 | 0.19 |
| Smad3 (Phospho-Ser213) | 706 | 236 | 140.5 | 128 | 134.25 | 674.59 | 247.52 | 461.06 | 301.98 | 0.65 |
| Smad3 (Phospho-Ser425) | 85 | 184 | 140.5 | 128 | 134.25 | 81.22 | 192.98 | 137.10 | 79.03 | 0.58 |
| Smad3 (Phospho-Thr179) | 170 | 63 | 140.5 | 128 | 134.25 | 162.44 | 66.08 | 114.26 | 68.14 | 0.60 |
| SMC1 (Ab-957) | 96 | 246 | 140.5 | 128 | 134.25 | 91.73 | 258.01 | 174.87 | 117.58 | 0.67 |
| SMC1 (Phospho-Ser957) | 133 | 264 | 140.5 | 128 | 134.25 | 127.08 | 276.89 | 201.99 | 105.93 | 0.52 |
| SP1 (Ab-739) | 105 | 90 | 140.5 | 128 | 134.25 | 100.33 | 94.39 | 97.36 | 4.20 | 0.04 |
| SP1 (Phospho-Thr739) | 123 | 113 | 140.5 | 128 | 134.25 | 117.53 | 118.52 | 118.02 | 0.70 | 0.01 |
| Src (Ab-418) | 3392 | 3188 | 140.5 | 128 | 134.25 | 3241.11 | 3343.66 | 3292.39 | 72.52 | 0.02 |
| Src (Ab-529) | 118 | 131 | 140.5 | 128 | 134.25 | 112.75 | 137.40 | 125.07 | 17.43 | 0.14 |
| Src (Ab-75) | 112 | 94 | 140.5 | 128 | 134.25 | 107.02 | 98.59 | 102.80 | 5.96 | 0.06 |
| Src (Phospho-Ser75) | 73 | 334 | 140.5 | 128 | 134.25 | 69.75 | 350.31 | 210.03 | 198.38 | 0.94 |
| Src (Phospho-Tyr216) | 247 | 238 | 140.5 | 128 | 134.25 | 236.01 | 249.62 | 242.82 | 9.62 | 0.04 |
| Src (Phospho-Tyr418) | 131 | 128 | 140.5 | 128 | 134.25 | 125.17 | 134.25 | 129.71 | 6.42 | 0.05 |
| Src (Phospho-Tyr529) | 106 | 95 | 140.5 | 128 | 134.25 | 101.28 | 99.64 | 100.46 | 1.16 | 0.01 |
| SREBP-1 (Ab-439) | 109 | 106 | 140.5 | 128 | 134.25 | 104.15 | 111.18 | 107.66 | 4.97 | 0.05 |
| SREBP-1 (Phospho-Ser439) | 84 | 81 | 140.5 | 128 | 134.25 | 80.26 | 84.96 | 82.61 | 3.32 | 0.04 |
| SRF (Ab-77) | 95 | 75 | 140.5 | 128 | 134.25 | 90.77 | 78.66 | 84.72 | 8.56 | 0.10 |
| SRF (Ab-99) | 633 | 747 | 140.5 | 128 | 134.25 | 604.84 | 783.47 | 694.16 | 126.31 | 0.18 |
| SRF (Phospho-Ser77) | 111 | 74 | 140.5 | 128 | 134.25 | 106.06 | 77.61 | 91.84 | 20.12 | 0.22 |
| SRF (Phospho-Ser99) | 145 | 143 | 140.5 | 128 | 134.25 | 138.55 | 149.98 | 144.27 | 8.08 | 0.06 |
| STAM2 (Ab-192) | 94 | 81 | 140.5 | 128 | 134.25 | 89.82 | 84.96 | 87.39 | 3.44 | 0.04 |
| STAM2 (Phospho-Tyr192) | 288 | 234 | 140.5 | 128 | 134.25 | 275.19 | 245.43 | 260.31 | 21.05 | 0.08 |
| STAT1 (Ab-701) | 105 | 87 | 140.5 | 128 | 134.25 | 100.33 | 91.25 | 95.79 | 6.42 | 0.07 |
| STAT1 (Ab-727) | 172 | 160 | 140.5 | 128 | 134.25 | 164.35 | 167.81 | 166.08 | 2.45 | 0.01 |
| STAT1 (Phospho-Ser727) | 101 | 92 | 140.5 | 128 | 134.25 | 96.51 | 96.49 | 96.50 | 0.01 | 0.00 |
| STAT1 (Phospho-Tyr701) | 418 | 396 | 140.5 | 128 | 134.25 | 399.41 | 415.34 | 407.37 | 11.26 | 0.03 |
| STAT2 (Ab-690) | 103 | 97 | 140.5 | 128 | 134.25 | 98.42 | 101.74 | 100.08 | 2.35 | 0.02 |
| STAT2 (Phospho-Tyr690) | 120 | 243 | 140.5 | 128 | 134.25 | 114.66 | 254.87 | 184.76 | 99.14 | 0.54 |
| STAT3 (Ab-705) | 224 | 199 | 140.5 | 128 | 134.25 | 214.04 | 208.72 | 211.38 | 3.76 | 0.02 |
| STAT3 (Ab-727) | 191 | 80 | 140.5 | 128 | 134.25 | 182.50 | 83.91 | 133.20 | 69.72 | 0.52 |
| STAT3 (Phospho-Ser727) | 95 | 111 | 140.5 | 128 | 134.25 | 90.77 | 116.42 | 103.60 | 18.13 | 0.18 |
| STAT3 (Phospho-Tyr705) | 379 | 159 | 140.5 | 128 | 134.25 | 362.14 | 166.76 | 264.45 | 138.15 | 0.52 |
| STAT4 (Ab-693) | 106 | 94 | 140.5 | 128 | 134.25 | 101.28 | 98.59 | 99.94 | 1.91 | 0.02 |
| STAT4 (Phospho-Tyr693) | 350 | 237 | 140.5 | 128 | 134.25 | 334.43 | 248.57 | 291.50 | 60.71 | 0.21 |
| STAT5A (Ab-694) | 91 | 80 | 140.5 | 128 | 134.25 | 86.95 | 83.91 | 85.43 | 2.15 | 0.03 |
| STAT5A (Ab-780) | 95 | 77 | 140.5 | 128 | 134.25 | 90.77 | 80.76 | 85.77 | 7.08 | 0.08 |
| STAT5A (Phospho-Ser725) | 112 | 84 | 140.5 | 128 | 134.25 | 107.02 | 88.10 | 97.56 | 13.38 | 0.14 |
| STAT5A (Phospho-Ser780) | 300 | 188 | 140.5 | 128 | 134.25 | 286.65 | 197.18 | 241.92 | 63.27 | 0.26 |
| STAT5A (Phospho-Tyr694) | 125 | 116 | 140.5 | 128 | 134.25 | 119.44 | 121.66 | 120.55 | 1.57 | 0.01 |
| STAT5B (Ab-731) | 103 | 85 | 140.5 | 128 | 134.25 | 98.42 | 89.15 | 93.78 | 6.55 | 0.07 |
| STAT5B (Phospho-Ser731) | 86 | 84 | 140.5 | 128 | 134.25 | 82.17 | 88.10 | 85.14 | 4.19 | 0.05 |
| STAT6 (Ab-641) | 138 | 122 | 140.5 | 128 | 134.25 | 131.86 | 127.96 | 129.91 | 2.76 | 0.02 |
| STAT6 (Ab-645) | 1110 | 1184 | 140.5 | 128 | 134.25 | 1060.62 | 1241.81 | 1151.22 | 128.12 | 0.11 |
| STAT6 (Phospho-Thr645) | 226 | 430 | 140.5 | 128 | 134.25 | 215.95 | 451.00 | 333.47 | 166.21 | 0.50 |
| STAT6 (Phospho-Tyr641) | 325 | 279 | 140.5 | 128 | 134.25 | 310.54 | 292.62 | 301.58 | 12.67 | 0.04 |
| Stathmin 1 (Ab-15) | 106 | 97 | 140.5 | 128 | 134.25 | 101.28 | 101.74 | 101.51 | 0.32 | 0.00 |
| Stathmin 1 (Ab-24) | 102 | 71 | 140.5 | 128 | 134.25 | 97.46 | 74.47 | 85.96 | 16.26 | 0.19 |
| Stathmin 1 (Ab-37) | 114 | 81 | 140.5 | 128 | 134.25 | 108.93 | 84.96 | 96.94 | 16.95 | 0.17 |
| Stathmin 1 (Phospho-Ser15) | 151 | 125 | 140.5 | 128 | 134.25 | 144.28 | 131.10 | 137.69 | 9.32 | 0.07 |
| Stathmin 1 (Phospho-Ser24) | 131 | 113 | 140.5 | 128 | 134.25 | 125.17 | 118.52 | 121.85 | 4.71 | 0.04 |
| Stathmin 1 (Phospho-Ser37) | 100 | 85 | 140.5 | 128 | 134.25 | 95.55 | 89.15 | 92.35 | 4.53 | 0.05 |
| Survivin (Ab-117) | 82 | 69 | 140.5 | 128 | 134.25 | 78.35 | 72.37 | 75.36 | 4.23 | 0.06 |
| Survivin (Phospho-Thr117) | 76 | 66 | 140.5 | 128 | 134.25 | 72.62 | 69.22 | 70.92 | 2.40 | 0.03 |
| SYK (Ab-348) | 237 | 286 | 140.5 | 128 | 134.25 | 226.46 | 299.96 | 263.21 | 51.98 | 0.20 |
| SYK (Ab-525) | 782 | 805 | 140.5 | 128 | 134.25 | 747.21 | 844.31 | 795.76 | 68.66 | 0.09 |
| SYK (Phospho-Tyr323) | 106 | 78 | 140.5 | 128 | 134.25 | 101.28 | 81.81 | 91.55 | 13.77 | 0.15 |
| SYK (Phospho-Tyr348) | 84 | 81 | 140.5 | 128 | 134.25 | 80.26 | 84.96 | 82.61 | 3.32 | 0.04 |
| SYK (Phospho-Tyr525) | 228 | 241 | 140.5 | 128 | 134.25 | 217.86 | 252.77 | 235.31 | 24.69 | 0.10 |
| Synapsin (Ab-62) | 105 | 157 | 140.5 | 128 | 134.25 | 100.33 | 164.67 | 132.50 | 45.49 | 0.34 |
| Synapsin (Ab-9) | 97 | 84 | 140.5 | 128 | 134.25 | 92.69 | 88.10 | 90.39 | 3.24 | 0.04 |
| Synapsin (Phospho-Ser62) | 290 | 261 | 140.5 | 128 | 134.25 | 277.10 | 273.74 | 275.42 | 2.37 | 0.01 |
| Synapsin (Phospho-Ser9) | 123 | 143 | 140.5 | 128 | 134.25 | 117.53 | 149.98 | 133.76 | 22.95 | 0.17 |
| Synaptotagmin (Ab-202) | 257 | 99 | 140.5 | 128 | 134.25 | 245.57 | 103.83 | 174.70 | 100.22 | 0.57 |
| Synaptotagmin (Ab-309) | 98 | 89 | 140.5 | 128 | 134.25 | 93.64 | 93.35 | 93.49 | 0.21 | 0.00 |
| Synaptotagmin (Phospho-Ser309) | 86 | 80 | 140.5 | 128 | 134.25 | 82.17 | 83.91 | 83.04 | 1.22 | 0.01 |
| Synaptotagmin (Phospho-Thr202) | 93 | 65 | 140.5 | 128 | 134.25 | 88.86 | 68.17 | 78.52 | 14.63 | 0.19 |
| Synuclein alpha (Ab-125) | 289 | 261 | 140.5 | 128 | 134.25 | 276.14 | 273.74 | 274.94 | 1.70 | 0.01 |
| Synuclein alpha (Ab-133) | 127 | 118 | 140.5 | 128 | 134.25 | 121.35 | 123.76 | 122.56 | 1.70 | 0.01 |
| Synuclein alpha (Phospho-Tyr125) | 148 | 154 | 140.5 | 128 | 134.25 | 141.42 | 161.52 | 151.47 | 14.22 | 0.09 |
| Synuclein alpha (Phospho-Tyr133) | 126 | 126 | 140.5 | 128 | 134.25 | 120.40 | 132.15 | 126.27 | 8.31 | 0.07 |
| Synuclein alpha (Phospho-Tyr136) | 110 | 271 | 140.5 | 128 | 134.25 | 105.11 | 284.23 | 194.67 | 126.66 | 0.65 |
| Tau (Ab-181) | 133 | 110 | 140.5 | 128 | 134.25 | 127.08 | 115.37 | 121.23 | 8.28 | 0.07 |
| Tau (Ab-205) | 346 | 300 | 140.5 | 128 | 134.25 | 330.61 | 314.65 | 322.63 | 11.29 | 0.03 |
| Tau (Ab-212) | 1288 | 1104 | 140.5 | 128 | 134.25 | 1230.70 | 1157.91 | 1194.31 | 51.48 | 0.04 |
| Tau (Ab-214) | 238 | 220 | 140.5 | 128 | 134.25 | 227.41 | 230.74 | 229.08 | 2.35 | 0.01 |
| Tau (Ab-231) | 113 | 110 | 140.5 | 128 | 134.25 | 107.97 | 115.37 | 111.67 | 5.23 | 0.05 |
| Tau (Ab-235) | 5473 | 4547 | 140.5 | 128 | 134.25 | 5229.54 | 4769.02 | 4999.28 | 325.64 | 0.07 |
| Tau (Ab-262) | 112 | 122 | 140.5 | 128 | 134.25 | 107.02 | 127.96 | 117.49 | 14.81 | 0.13 |
| Tau (Ab-356) | 2135 | 2408 | 140.5 | 128 | 134.25 | 2040.03 | 2525.58 | 2282.80 | 343.34 | 0.15 |
| Tau (Ab-396) | 292 | 369 | 140.5 | 128 | 134.25 | 279.01 | 387.02 | 333.01 | 76.37 | 0.23 |
| Tau (Ab-404) | 360 | 308 | 140.5 | 128 | 134.25 | 343.99 | 323.04 | 333.51 | 14.81 | 0.04 |
| Tau (Ab-422) | 197 | 82 | 140.5 | 128 | 134.25 | 188.24 | 86.00 | 137.12 | 72.29 | 0.53 |
| Tau (Phospho-Ser214) | 121 | 102 | 140.5 | 128 | 134.25 | 115.62 | 106.98 | 111.30 | 6.11 | 0.05 |
| Tau (Phospho-Ser235) | 291 | 256 | 140.5 | 128 | 134.25 | 278.06 | 268.50 | 273.28 | 6.76 | 0.02 |
| Tau (Phospho-Ser262) | 115 | 82 | 140.5 | 128 | 134.25 | 109.88 | 86.00 | 97.94 | 16.89 | 0.17 |
| Tau (Phospho-Ser356) | 122 | 116 | 140.5 | 128 | 134.25 | 116.57 | 121.66 | 119.12 | 3.60 | 0.03 |
| Tau (Phospho-Ser396) | 116 | 455 | 140.5 | 128 | 134.25 | 110.84 | 477.22 | 294.03 | 259.07 | 0.88 |
| Tau (Phospho-Ser404) | 141 | 111 | 140.5 | 128 | 134.25 | 134.73 | 116.42 | 125.57 | 12.95 | 0.10 |
| Tau (Phospho-Ser422) | 134 | 108 | 140.5 | 128 | 134.25 | 128.04 | 113.27 | 120.66 | 10.44 | 0.09 |
| Tau (Phospho-Thr181) | 358 | 156 | 140.5 | 128 | 134.25 | 342.07 | 163.62 | 252.85 | 126.19 | 0.50 |
| Tau (Phospho-Thr205) | 156 | 162 | 140.5 | 128 | 134.25 | 149.06 | 169.91 | 159.49 | 14.74 | 0.09 |
| Tau (Phospho-Thr212) | 160 | 144 | 140.5 | 128 | 134.25 | 152.88 | 151.03 | 151.96 | 1.31 | 0.01 |
| Tau (Phospho-Thr231) | 410 | 206 | 140.5 | 128 | 134.25 | 391.76 | 216.06 | 303.91 | 124.24 | 0.41 |
| TFII-I (Phospho-Tyr248) | 85 | 115 | 140.5 | 128 | 134.25 | 81.22 | 120.62 | 100.92 | 27.86 | 0.28 |
| TGFBR1 (Ab-165) | 98 | 88 | 140.5 | 128 | 134.25 | 93.64 | 92.30 | 92.97 | 0.95 | 0.01 |
| TGFBR2 (Ab-250) | 903 | 986 | 140.5 | 128 | 134.25 | 862.83 | 1034.14 | 948.49 | 121.14 | 0.13 |
| TIE2 (Phospho-Tyr1108) | 145 | 137 | 140.5 | 128 | 134.25 | 138.55 | 143.69 | 141.12 | 3.63 | 0.03 |
| TIF-IA (Ab-649) | 112 | 92 | 140.5 | 128 | 134.25 | 107.02 | 96.49 | 101.75 | 7.44 | 0.07 |
| TIF-IA (Phospho-Ser649) | 143 | 135 | 140.5 | 128 | 134.25 | 136.64 | 141.59 | 139.12 | 3.50 | 0.03 |
| TLK1 (Ab-764) | 534 | 450 | 140.5 | 128 | 134.25 | 510.25 | 471.97 | 491.11 | 27.06 | 0.06 |
| TOP2A/DNA topoisomerase II (Ab-1106) | 307 | 270 | 140.5 | 128 | 134.25 | 293.34 | 283.18 | 288.26 | 7.18 | 0.02 |
| TOP2A/DNA topoisomerase II (Phospho-Ser1106) | 155 | 79 | 140.5 | 128 | 134.25 | 148.10 | 82.86 | 115.48 | 46.14 | 0.40 |
| Trk A (Ab-496) | 115 | 120 | 140.5 | 128 | 134.25 | 109.88 | 125.86 | 117.87 | 11.30 | 0.10 |
| Trk A (Phospho-Tyr680/681) | 89 | 77 | 140.5 | 128 | 134.25 | 85.04 | 80.76 | 82.90 | 3.03 | 0.04 |
| Trk A (Phospho-Tyr701) | 116 | 118 | 140.5 | 128 | 134.25 | 110.84 | 123.76 | 117.30 | 9.14 | 0.08 |
| Trk A (Phospho-Tyr791) | 96 | 81 | 140.5 | 128 | 134.25 | 91.73 | 84.96 | 88.34 | 4.79 | 0.05 |
| Trk B (Ab-515) | 525 | 606 | 140.5 | 128 | 134.25 | 501.65 | 635.59 | 568.62 | 94.71 | 0.17 |
| Trk B (Phospho-Tyr515) | 220 | 186 | 140.5 | 128 | 134.25 | 210.21 | 195.08 | 202.65 | 10.70 | 0.05 |
| Trk B (Phospho-Tyr705) | 112 | 75 | 140.5 | 128 | 134.25 | 107.02 | 78.66 | 92.84 | 20.05 | 0.22 |
| Tuberin/TSC2 (Ab-1462) | 78 | 81 | 140.5 | 128 | 134.25 | 74.53 | 84.96 | 79.74 | 7.37 | 0.09 |
| Tuberin/TSC2 (Ab-939) | 705 | 653 | 140.5 | 128 | 134.25 | 673.64 | 684.88 | 679.26 | 7.95 | 0.01 |
| Tuberin/TSC2 (Ab-981) | 125 | 154 | 140.5 | 128 | 134.25 | 119.44 | 161.52 | 140.48 | 29.76 | 0.21 |
| Tuberin/TSC2 (Phospho-Ser939) | 93 | 91 | 140.5 | 128 | 134.25 | 88.86 | 95.44 | 92.15 | 4.65 | 0.05 |
| Tuberin/TSC2 (Phospho-Thr1462) | 154 | 165 | 140.5 | 128 | 134.25 | 147.15 | 173.06 | 160.10 | 18.32 | 0.11 |
| TYK2 (Ab-1054) | 101 | 100 | 140.5 | 128 | 134.25 | 96.51 | 104.88 | 100.69 | 5.92 | 0.06 |
| TYK2 (Phospho-Tyr1054) | 295 | 141 | 140.5 | 128 | 134.25 | 281.88 | 147.88 | 214.88 | 94.75 | 0.44 |
| Tyrosine Hydroxylase (Ab-19) | 193 | 182 | 140.5 | 128 | 134.25 | 184.41 | 190.89 | 187.65 | 4.58 | 0.02 |
| Tyrosine Hydroxylase (Ab-31) | 170 | 110 | 140.5 | 128 | 134.25 | 162.44 | 115.37 | 138.90 | 33.28 | 0.24 |
| Tyrosine Hydroxylase (Ab-40) | 235 | 188 | 140.5 | 128 | 134.25 | 224.55 | 197.18 | 210.86 | 19.35 | 0.09 |
| Tyrosine Hydroxylase (Ab-8) | 239 | 230 | 140.5 | 128 | 134.25 | 228.37 | 241.23 | 234.80 | 9.09 | 0.04 |
| Tyrosine Hydroxylase (Phospho-Ser19) | 127 | 101 | 140.5 | 128 | 134.25 | 121.35 | 105.93 | 113.64 | 10.90 | 0.10 |
| Tyrosine Hydroxylase (Phospho-Ser31) | 97 | 87 | 140.5 | 128 | 134.25 | 92.69 | 91.25 | 91.97 | 1.02 | 0.01 |
| Tyrosine Hydroxylase (Phospho-Ser40) | 93 | 377 | 140.5 | 128 | 134.25 | 88.86 | 395.41 | 242.14 | 216.76 | 0.90 |
| Tyrosine Hydroxylase (Phospho-Ser8) | 110 | 99 | 140.5 | 128 | 134.25 | 105.11 | 103.83 | 104.47 | 0.90 | 0.01 |
| VASP (Ab-157) | 855 | 1125 | 140.5 | 128 | 134.25 | 816.97 | 1179.93 | 998.45 | 256.66 | 0.26 |
| VASP (Ab-238) | 99 | 101 | 140.5 | 128 | 134.25 | 94.60 | 105.93 | 100.26 | 8.02 | 0.08 |
| VASP (Phospho-Ser157) | 451 | 183 | 140.5 | 128 | 134.25 | 430.94 | 191.94 | 311.44 | 169.00 | 0.54 |
| VASP (Phospho-Ser238) | 112 | 102 | 140.5 | 128 | 134.25 | 107.02 | 106.98 | 107.00 | 0.03 | 0.00 |
| VAV1 (Ab-160) | 292 | 277 | 140.5 | 128 | 134.25 | 279.01 | 290.53 | 284.77 | 8.14 | 0.03 |
| VAV1 (Ab-174) | 172 | 162 | 140.5 | 128 | 134.25 | 164.35 | 169.91 | 167.13 | 3.93 | 0.02 |
| VAV1 (Phospho-Tyr174) | 415 | 82 | 140.5 | 128 | 134.25 | 396.54 | 86.00 | 241.27 | 219.58 | 0.91 |
| VAV2 (Ab-142) | 79 | 436 | 140.5 | 128 | 134.25 | 75.49 | 457.29 | 266.39 | 269.98 | 1.01 |
| VAV2 (Phospho-Tyr142) | 204 | 192 | 140.5 | 128 | 134.25 | 194.93 | 201.38 | 198.15 | 4.56 | 0.02 |
| VE-Cadherin (Phospho-Tyr731) | 1177 | 1656 | 140.5 | 128 | 134.25 | 1124.64 | 1736.86 | 1430.75 | 432.90 | 0.30 |
| VEGFR1 (Ab-1333) | 139 | 113 | 140.5 | 128 | 134.25 | 132.82 | 118.52 | 125.67 | 10.11 | 0.08 |
| VEGFR1 (Phospho-Tyr1333) | 152 | 276 | 140.5 | 128 | 134.25 | 145.24 | 289.48 | 217.36 | 101.99 | 0.47 |
| VEGFR2 (Ab-1054) | 101 | 86 | 140.5 | 128 | 134.25 | 96.51 | 90.20 | 93.35 | 4.46 | 0.05 |
| VEGFR2 (Ab-1059) | 95 | 63 | 140.5 | 128 | 134.25 | 90.77 | 66.08 | 78.43 | 17.46 | 0.22 |
| VEGFR2 (Ab-1175) | 175 | 170 | 140.5 | 128 | 134.25 | 167.22 | 178.30 | 172.76 | 7.84 | 0.05 |
| VEGFR2 (Ab-1214) | 3018 | 3163 | 140.5 | 128 | 134.25 | 2883.75 | 3317.44 | 3100.60 | 306.67 | 0.10 |
| VEGFR2 (Ab-951) | 4821 | 3842 | 140.5 | 128 | 134.25 | 4606.54 | 4029.60 | 4318.07 | 407.96 | 0.09 |
| VEGFR2 (Phospho-Tyr1054) | 122 | 112 | 140.5 | 128 | 134.25 | 116.57 | 117.47 | 117.02 | 0.63 | 0.01 |
| VEGFR2 (Phospho-Tyr1059) | 210 | 204 | 140.5 | 128 | 134.25 | 200.66 | 213.96 | 207.31 | 9.41 | 0.05 |
| VEGFR2 (Phospho-Tyr1175) | 131 | 122 | 140.5 | 128 | 134.25 | 125.17 | 127.96 | 126.56 | 1.97 | 0.02 |
| VEGFR2 (Phospho-Tyr1214) | 413 | 160 | 140.5 | 128 | 134.25 | 394.63 | 167.81 | 281.22 | 160.38 | 0.57 |
| VEGFR2 (Phospho-Tyr951) | 194 | 173 | 140.5 | 128 | 134.25 | 185.37 | 181.45 | 183.41 | 2.77 | 0.02 |
| Vinculin (Ab-821) | 74 | 59 | 140.5 | 128 | 134.25 | 70.71 | 61.88 | 66.29 | 6.24 | 0.09 |
| Vinculin (Phospho-Tyr821) | 403 | 173 | 140.5 | 128 | 134.25 | 385.07 | 181.45 | 283.26 | 143.99 | 0.51 |
| WASP (Ab-290) | 241 | 170 | 140.5 | 128 | 134.25 | 230.28 | 178.30 | 204.29 | 36.75 | 0.18 |
| WASP (Phospho-Tyr290) | 80 | 87 | 140.5 | 128 | 134.25 | 76.44 | 91.25 | 83.84 | 10.47 | 0.12 |
| WAVE1 (Ab-125) | 102 | 86 | 140.5 | 128 | 134.25 | 97.46 | 90.20 | 93.83 | 5.14 | 0.05 |
| WAVE1 (Phospho-Tyr125) | 87 | 79 | 140.5 | 128 | 134.25 | 83.13 | 82.86 | 82.99 | 0.19 | 0.00 |
| WEE1 (Ab-53) | 99 | 84 | 140.5 | 128 | 134.25 | 94.60 | 88.10 | 91.35 | 4.59 | 0.05 |
| WEE1 (Phospho-Ser53) | 83 | 76 | 140.5 | 128 | 134.25 | 79.31 | 79.71 | 79.51 | 0.29 | 0.00 |
| WEE1 (Phospho-Ser642) | 126 | 107 | 140.5 | 128 | 134.25 | 120.40 | 112.22 | 116.31 | 5.78 | 0.05 |
| WWOX (Phospho-Tyr33) | 244 | 121 | 140.5 | 128 | 134.25 | 233.15 | 126.91 | 180.03 | 75.12 | 0.42 |
| XIAP (Ab-87) | 175 | 169 | 140.5 | 128 | 134.25 | 167.22 | 177.25 | 172.23 | 7.10 | 0.04 |
| XIAP (Phospho-Ser87) | 85 | 67 | 140.5 | 128 | 134.25 | 81.22 | 70.27 | 75.75 | 7.74 | 0.10 |
| Zap-70 (Ab-292) | 285 | 131 | 140.5 | 128 | 134.25 | 272.32 | 137.40 | 204.86 | 95.41 | 0.47 |
| Zap-70 (Ab-319) | 489 | 439 | 140.5 | 128 | 134.25 | 467.25 | 460.44 | 463.84 | 4.82 | 0.01 |
| Zap-70 (Ab-493) | 214 | 190 | 140.5 | 128 | 134.25 | 204.48 | 199.28 | 201.88 | 3.68 | 0.02 |
| Zap-70 (Phospho-Tyr292) | 81 | 79 | 140.5 | 128 | 134.25 | 77.40 | 82.86 | 80.13 | 3.86 | 0.05 |
| Zap-70 (Phospho-Tyr315) | 136 | 134 | 140.5 | 128 | 134.25 | 129.95 | 140.54 | 135.25 | 7.49 | 0.06 |
| Zap-70 (Phospho-Tyr319) | 200 | 172 | 140.5 | 128 | 134.25 | 191.10 | 180.40 | 185.75 | 7.57 | 0.04 |
| Zap-70 (Phospho-Tyr493) | 148 | 162 | 140.5 | 128 | 134.25 | 141.42 | 169.91 | 155.66 | 20.15 | 0.13 |

**Supplementary Table 3. The standard detection value of PZH group**

| Name | FI_i_ | FI_j_ | X | Y | Z | I_i_ | I_j_ | Average | SD | CV |
| --- | --- | --- | --- | --- | --- | --- | --- | --- | --- | --- |
| Positive Marker | 4070 | 4231 | 135 | 124 | 129.5 | 3904.19 | 4418.67 | 4161.43 | 363.79 | 0.09 |
| Positive Marker | 3802 | 4545 | 135 | 124 | 129.5 | 3647.10 | 4746.59 | 4196.85 | 777.46 | 0.19 |
| Negative control | 142 | 139 | 135 | 124 | 129.5 | 136.21 | 145.17 | 140.69 | 6.33 | 0.04 |
| Negative control | 137 | 136 | 135 | 124 | 129.5 | 131.42 | 142.03 | 136.73 | 7.51 | 0.05 |
| Negative control | 141 | 135 | 135 | 124 | 129.5 | 135.26 | 140.99 | 138.12 | 4.05 | 0.03 |
| Negative control | 137 | 136 | 135 | 124 | 129.5 | 131.42 | 142.03 | 136.73 | 7.51 | 0.05 |
| Empty | 54 | 48 | 135 | 124 | 129.5 | 51.80 | 50.13 | 50.96 | 1.18 | 0.02 |
| Empty | 54 | 46 | 135 | 124 | 129.5 | 51.80 | 48.04 | 49.92 | 2.66 | 0.05 |
| Empty | 54 | 44 | 135 | 124 | 129.5 | 51.80 | 45.95 | 48.88 | 4.14 | 0.08 |
| Empty | 54 | 44 | 135 | 124 | 129.5 | 51.80 | 45.95 | 48.88 | 4.14 | 0.08 |
| 14-3-3 beta/zeta (Ab-184/186) | 305 | 250 | 135 | 124 | 129.5 | 292.57 | 261.09 | 276.83 | 22.26 | 0.08 |
| 14-3-3 beta/zeta (Phospho-Ser184/186) | 91 | 154 | 135 | 124 | 129.5 | 87.29 | 160.83 | 124.06 | 52.00 | 0.42 |
| 14-3-3 theta/tau (Ab-232) | 87 | 72 | 135 | 124 | 129.5 | 83.46 | 75.19 | 79.32 | 5.84 | 0.07 |
| 14-3-3 theta/tau (Phospho-Ser232) | 149 | 146 | 135 | 124 | 129.5 | 142.93 | 152.48 | 147.70 | 6.75 | 0.05 |
| 14-3-3 zeta (Ab-58) | 105 | 95 | 135 | 124 | 129.5 | 100.72 | 99.21 | 99.97 | 1.07 | 0.01 |
| 14-3-3 zeta (Phospho-Ser58) | 109 | 124 | 135 | 124 | 129.5 | 104.56 | 129.50 | 117.03 | 17.64 | 0.15 |
| 14-3-3 zeta/delta (Ab-232) | 772 | 1800 | 135 | 124 | 129.5 | 740.55 | 1879.84 | 1310.19 | 805.60 | 0.61 |
| 14-3-3 zeta/delta (Phospho-Thr232) | 255 | 235 | 135 | 124 | 129.5 | 244.61 | 245.42 | 245.02 | 0.57 | 0.00 |
| 4E-BP1 (Ab-36) | 92 | 73 | 135 | 124 | 129.5 | 88.25 | 76.24 | 82.24 | 8.50 | 0.10 |
| 4E-BP1 (Ab-45) | 109 | 89 | 135 | 124 | 129.5 | 104.56 | 92.95 | 98.75 | 8.21 | 0.08 |
| 4E-BP1 (Ab-65) | 1274 | 2298 | 135 | 124 | 129.5 | 1222.10 | 2399.93 | 1811.01 | 832.85 | 0.46 |
| 4E-BP1 (Ab-70) | 212 | 228 | 135 | 124 | 129.5 | 203.36 | 238.11 | 220.74 | 24.57 | 0.11 |
| 4E-BP1 (Phospho-Ser65) | 89 | 78 | 135 | 124 | 129.5 | 85.37 | 81.46 | 83.42 | 2.77 | 0.03 |
| 4E-BP1 (Phospho-Thr36) | 130 | 197 | 135 | 124 | 129.5 | 124.70 | 205.74 | 165.22 | 57.30 | 0.35 |
| 4E-BP1 (Phospho-Thr45) | 115 | 100 | 135 | 124 | 129.5 | 110.31 | 104.44 | 107.38 | 4.16 | 0.04 |
| 4E-BP1 (Phospho-Thr70) | 361 | 76 | 135 | 124 | 129.5 | 346.29 | 79.37 | 212.83 | 188.74 | 0.89 |
| 6-phosphofructo-2-kinase/fructose-2,6-biphosphatase 2 (PFKFB2) (Ab-483) | 101 | 89 | 135 | 124 | 129.5 | 96.89 | 92.95 | 94.92 | 2.78 | 0.03 |
| 6-phosphofructo-2-kinase/fructose-2,6-biphosphatase 2 (PFKFB2) (Phospho-Ser483) | 89 | 83 | 135 | 124 | 129.5 | 85.37 | 86.68 | 86.03 | 0.92 | 0.01 |
| Abl1 (Ab-204) | 636 | 2245 | 135 | 124 | 129.5 | 610.09 | 2344.58 | 1477.33 | 1226.47 | 0.83 |
| Abl1 (Ab-754/735) | 284 | 325 | 135 | 124 | 129.5 | 272.43 | 339.42 | 305.92 | 47.37 | 0.15 |
| Abl1 (Phospho-Thr754/735) | 109 | 92 | 135 | 124 | 129.5 | 104.56 | 96.08 | 100.32 | 6.00 | 0.06 |
| Abl1 (Phospho-Tyr204) | 92 | 328 | 135 | 124 | 129.5 | 88.25 | 342.55 | 215.40 | 179.81 | 0.83 |
| Abl1 (Phospho-Tyr412) | 105 | 74 | 135 | 124 | 129.5 | 100.72 | 77.28 | 89.00 | 16.57 | 0.19 |
| ACC1 (Ab-79) | 139 | 92 | 135 | 124 | 129.5 | 133.34 | 96.08 | 114.71 | 26.34 | 0.23 |
| ACC1 (Ab-80) | 679 | 293 | 135 | 124 | 129.5 | 651.34 | 306.00 | 478.67 | 244.19 | 0.51 |
| ACC1 (Phospho-Ser79) | 271 | 84 | 135 | 124 | 129.5 | 259.96 | 87.73 | 173.84 | 121.79 | 0.70 |
| ACC1 (Phospho-Ser80) | 82 | 74 | 135 | 124 | 129.5 | 78.66 | 77.28 | 77.97 | 0.97 | 0.01 |
| ACK1 (Phospho-Tyr284) | 343 | 333 | 135 | 124 | 129.5 | 329.03 | 347.77 | 338.40 | 13.25 | 0.04 |
| Actin Pan (a/b/g) (Ab-55/53) | 173 | 246 | 135 | 124 | 129.5 | 165.95 | 256.91 | 211.43 | 64.32 | 0.30 |
| Actin Pan (a/b/g) (Phospho-Tyr55/53) | 100 | 93 | 135 | 124 | 129.5 | 95.93 | 97.13 | 96.53 | 0.85 | 0.01 |
| ADD1 (Ab-726) | 135 | 115 | 135 | 124 | 129.5 | 129.50 | 120.10 | 124.80 | 6.65 | 0.05 |
| ADD1 (Phospho-Ser726) | 128 | 161 | 135 | 124 | 129.5 | 122.79 | 168.14 | 145.46 | 32.07 | 0.22 |
| AFX/FOXO4 (Ab-197) | 133 | 122 | 135 | 124 | 129.5 | 127.58 | 127.41 | 127.50 | 0.12 | 0.00 |
| AFX/FOXO4 (Phospho-Ser197) | 108 | 103 | 135 | 124 | 129.5 | 103.60 | 107.57 | 105.58 | 2.81 | 0.03 |
| AKT1 (Ab-124) | 267 | 268 | 135 | 124 | 129.5 | 256.12 | 279.89 | 268.00 | 16.80 | 0.06 |
| AKT1 (Ab-246) | 97 | 84 | 135 | 124 | 129.5 | 93.05 | 87.73 | 90.39 | 3.76 | 0.04 |
| AKT1 (Ab-308) | 197 | 192 | 135 | 124 | 129.5 | 188.97 | 200.52 | 194.75 | 8.16 | 0.04 |
| AKT1 (Ab-326) | 144 | 134 | 135 | 124 | 129.5 | 138.13 | 139.94 | 139.04 | 1.28 | 0.01 |
| AKT1 (Ab-450) | 121 | 312 | 135 | 124 | 129.5 | 116.07 | 325.84 | 220.95 | 148.33 | 0.67 |
| AKT1 (Ab-473) | 102 | 82 | 135 | 124 | 129.5 | 97.84 | 85.64 | 91.74 | 8.63 | 0.09 |
| AKT1 (Ab-474) | 117 | 93 | 135 | 124 | 129.5 | 112.23 | 97.13 | 104.68 | 10.68 | 0.10 |
| AKT1 (Ab-72) | 111 | 99 | 135 | 124 | 129.5 | 106.48 | 103.39 | 104.93 | 2.18 | 0.02 |
| AKT1 (Phospho-Ser124) | 101 | 83 | 135 | 124 | 129.5 | 96.89 | 86.68 | 91.78 | 7.22 | 0.08 |
| AKT1 (Phospho-Ser246) | 84 | 245 | 135 | 124 | 129.5 | 80.58 | 255.87 | 168.22 | 123.95 | 0.74 |
| AKT1 (Phospho-Ser473) | 154 | 85 | 135 | 124 | 129.5 | 147.73 | 88.77 | 118.25 | 41.69 | 0.35 |
| AKT1 (Phospho-Thr308) | 196 | 203 | 135 | 124 | 129.5 | 188.01 | 212.00 | 200.01 | 16.96 | 0.08 |
| AKT1 (Phospho-Thr450) | 76 | 59 | 135 | 124 | 129.5 | 72.90 | 61.62 | 67.26 | 7.98 | 0.12 |
| AKT1 (Phospho-Thr72) | 80 | 66 | 135 | 124 | 129.5 | 76.74 | 68.93 | 72.83 | 5.52 | 0.08 |
| AKT1 (Phospho-Tyr326) | 101 | 96 | 135 | 124 | 129.5 | 96.89 | 100.26 | 98.57 | 2.38 | 0.02 |
| AKT1 (Phospho-Tyr474) | 82 | 184 | 135 | 124 | 129.5 | 78.66 | 192.16 | 135.41 | 80.26 | 0.59 |
| AKT1/2/3 (Ab-315) | 185 | 82 | 135 | 124 | 129.5 | 177.46 | 85.64 | 131.55 | 64.93 | 0.49 |
| AKT1S1 (Ab-246) | 995 | 1137 | 135 | 124 | 129.5 | 954.46 | 1187.43 | 1070.95 | 164.73 | 0.15 |
| AKT1S1 (Phospho-Thr246) | 224 | 181 | 135 | 124 | 129.5 | 214.87 | 189.03 | 201.95 | 18.28 | 0.09 |
| AKT2 (Ab-474) | 111 | 95 | 135 | 124 | 129.5 | 106.48 | 99.21 | 102.85 | 5.14 | 0.05 |
| AKT2 (Phospho-Ser474) | 210 | 188 | 135 | 124 | 129.5 | 201.44 | 196.34 | 198.89 | 3.61 | 0.02 |
| ALK (Ab-1507) | 96 | 95 | 135 | 124 | 129.5 | 92.09 | 99.21 | 95.65 | 5.04 | 0.05 |
| ALK (Ab-1604) | 122 | 141 | 135 | 124 | 129.5 | 117.03 | 147.25 | 132.14 | 21.37 | 0.16 |
| ALK (Phospho-Tyr1507) | 361 | 251 | 135 | 124 | 129.5 | 346.29 | 262.13 | 304.21 | 59.51 | 0.20 |
| ALK (Phospho-Tyr1604) | 603 | 128 | 135 | 124 | 129.5 | 578.43 | 133.68 | 356.06 | 314.49 | 0.88 |
| AMPK beta1 (Ab-182) | 357 | 193 | 135 | 124 | 129.5 | 342.46 | 201.56 | 272.01 | 99.63 | 0.37 |
| AMPK beta1 (Phospho-Ser182) | 81 | 66 | 135 | 124 | 129.5 | 77.70 | 68.93 | 73.31 | 6.20 | 0.08 |
| AMPK1 (Ab-172) | 99 | 92 | 135 | 124 | 129.5 | 94.97 | 96.08 | 95.52 | 0.79 | 0.01 |
| AMPK1 (Phospho-Thr172) | 135 | 113 | 135 | 124 | 129.5 | 129.50 | 118.01 | 123.76 | 8.12 | 0.07 |
| AMPK1/AMPK2 (Ab-485/491) | 110 | 93 | 135 | 124 | 129.5 | 105.52 | 97.13 | 101.32 | 5.94 | 0.06 |
| AMPK1/AMPK2 (Phospho-Ser485/491) | 119 | 128 | 135 | 124 | 129.5 | 114.15 | 133.68 | 123.91 | 13.81 | 0.11 |
| Amyloid beta A4 (Ab-743/668) | 566 | 560 | 135 | 124 | 129.5 | 542.94 | 584.84 | 563.89 | 29.63 | 0.05 |
| Amyloid beta A4 (Phospho-Thr743/668) | 184 | 178 | 135 | 124 | 129.5 | 176.50 | 185.90 | 181.20 | 6.64 | 0.04 |
| Androgen Receptor (Ab-213) | 125 | 124 | 135 | 124 | 129.5 | 119.91 | 129.50 | 124.70 | 6.78 | 0.05 |
| Androgen Receptor (Ab-650) | 159 | 159 | 135 | 124 | 129.5 | 152.52 | 166.05 | 159.29 | 9.57 | 0.06 |
| Androgen Receptor (Phospho-Ser213) | 129 | 117 | 135 | 124 | 129.5 | 123.74 | 122.19 | 122.97 | 1.10 | 0.01 |
| Androgen Receptor (Phospho-Ser650) | 116 | 106 | 135 | 124 | 129.5 | 111.27 | 110.70 | 110.99 | 0.40 | 0.00 |
| A-RAF (Ab-301/302) | 106 | 92 | 135 | 124 | 129.5 | 101.68 | 96.08 | 98.88 | 3.96 | 0.04 |
| A-RAF (Phospho-Tyr301/302) | 88 | 95 | 135 | 124 | 129.5 | 84.41 | 99.21 | 91.81 | 10.46 | 0.11 |
| Arrestin-1 (Ab-412) | 198 | 161 | 135 | 124 | 129.5 | 189.93 | 168.14 | 179.04 | 15.41 | 0.09 |
| Arrestin-1 (Phospho-Ser412) | 132 | 127 | 135 | 124 | 129.5 | 126.62 | 132.63 | 129.63 | 4.25 | 0.03 |
| ASK1 (Ab-83) | 105 | 96 | 135 | 124 | 129.5 | 100.72 | 100.26 | 100.49 | 0.33 | 0.00 |
| ASK1 (Ab-966) | 220 | 223 | 135 | 124 | 129.5 | 211.04 | 232.89 | 221.96 | 15.45 | 0.07 |
| ASK1 (Phospho-Ser83) | 134 | 145 | 135 | 124 | 129.5 | 128.54 | 151.43 | 139.99 | 16.19 | 0.12 |
| ASK1 (Phospho-Ser966) | 125 | 284 | 135 | 124 | 129.5 | 119.91 | 296.60 | 208.25 | 124.94 | 0.60 |
| ATF1 (Ab-63) | 119 | 112 | 135 | 124 | 129.5 | 114.15 | 116.97 | 115.56 | 1.99 | 0.02 |
| ATF1 (Phospho-Ser63) | 92 | 88 | 135 | 124 | 129.5 | 88.25 | 91.90 | 90.08 | 2.58 | 0.03 |
| ATF2 (Ab-112/94) | 106 | 90 | 135 | 124 | 129.5 | 101.68 | 93.99 | 97.84 | 5.44 | 0.06 |
| ATF2 (Ab-62/44) | 1312 | 1235 | 135 | 124 | 129.5 | 1258.55 | 1289.78 | 1274.16 | 22.08 | 0.02 |
| ATF2 (Ab-69/51) | 156 | 125 | 135 | 124 | 129.5 | 149.64 | 130.54 | 140.09 | 13.51 | 0.10 |
| ATF2 (Ab-71/53) | 93 | 79 | 135 | 124 | 129.5 | 89.21 | 82.50 | 85.86 | 4.74 | 0.06 |
| ATF2 (Ab-73/55) | 99 | 84 | 135 | 124 | 129.5 | 94.97 | 87.73 | 91.35 | 5.12 | 0.06 |
| ATF2 (Phospho-Ser112/94) | 303 | 153 | 135 | 124 | 129.5 | 290.66 | 159.79 | 225.22 | 92.54 | 0.41 |
| ATF2 (Phospho-Ser62/44) | 116 | 102 | 135 | 124 | 129.5 | 111.27 | 106.52 | 108.90 | 3.36 | 0.03 |
| ATF2 (Phospho-Thr69/51) | 119 | 104 | 135 | 124 | 129.5 | 114.15 | 108.61 | 111.38 | 3.92 | 0.04 |
| ATF2 (Phospho-Thr71/53) | 126 | 155 | 135 | 124 | 129.5 | 120.87 | 161.88 | 141.37 | 29.00 | 0.21 |
| ATF2 (Phospho-Thr73/55) | 167 | 138 | 135 | 124 | 129.5 | 160.20 | 144.12 | 152.16 | 11.37 | 0.07 |
| ATF4 (Ab-245) | 97 | 84 | 135 | 124 | 129.5 | 93.05 | 87.73 | 90.39 | 3.76 | 0.04 |
| ATF4 (Phospho-Ser245) | 108 | 318 | 135 | 124 | 129.5 | 103.60 | 332.10 | 217.85 | 161.58 | 0.74 |
| ATM (Ab-1981) | 103 | 95 | 135 | 124 | 129.5 | 98.80 | 99.21 | 99.01 | 0.29 | 0.00 |
| ATP1A1/Na+K+ ATPase1 (Ab-23) | 358 | 140 | 135 | 124 | 129.5 | 343.41 | 146.21 | 244.81 | 139.45 | 0.57 |
| ATP1A1/Na+K+ ATPase1 (Phospho-Ser23) | 178 | 163 | 135 | 124 | 129.5 | 170.75 | 170.23 | 170.49 | 0.37 | 0.00 |
| ATPase (Ab-16) | 73 | 62 | 135 | 124 | 129.5 | 70.03 | 64.75 | 67.39 | 3.73 | 0.06 |
| ATPase (Phospho-Ser16) | 84 | 80 | 135 | 124 | 129.5 | 80.58 | 83.55 | 82.06 | 2.10 | 0.03 |
| ATP-Citrate Lyase (Ab-454) | 117 | 100 | 135 | 124 | 129.5 | 112.23 | 104.44 | 108.33 | 5.51 | 0.05 |
| ATP-Citrate Lyase (Phospho-Ser454) | 151 | 103 | 135 | 124 | 129.5 | 144.85 | 107.57 | 126.21 | 26.36 | 0.21 |
| ATRIP (Ab-68/72) | 1192 | 1048 | 135 | 124 | 129.5 | 1143.44 | 1094.48 | 1118.96 | 34.62 | 0.03 |
| ATRIP (Phospho-Ser68/72) | 91 | 83 | 135 | 124 | 129.5 | 87.29 | 86.68 | 86.99 | 0.43 | 0.00 |
| AurA (Ab-288) | 131 | 118 | 135 | 124 | 129.5 | 125.66 | 123.23 | 124.45 | 1.72 | 0.01 |
| AurA (Ab-342) | 104 | 100 | 135 | 124 | 129.5 | 99.76 | 104.44 | 102.10 | 3.30 | 0.03 |
| AurA (Phospho-Ser342) | 97 | 174 | 135 | 124 | 129.5 | 93.05 | 181.72 | 137.38 | 62.70 | 0.46 |
| AurA (Phospho-Thr288) | 421 | 116 | 135 | 124 | 129.5 | 403.85 | 121.15 | 262.50 | 199.90 | 0.76 |
| AurB (Ab-12) | 89 | 83 | 135 | 124 | 129.5 | 85.37 | 86.68 | 86.03 | 0.92 | 0.01 |
| AurB (Ab-232) | 92 | 85 | 135 | 124 | 129.5 | 88.25 | 88.77 | 88.51 | 0.37 | 0.00 |
| AurB (Phospho-Thr232) | 95 | 145 | 135 | 124 | 129.5 | 91.13 | 151.43 | 121.28 | 42.64 | 0.35 |
| AurB (Phospho-Tyr12) | 96 | 86 | 135 | 124 | 129.5 | 92.09 | 89.81 | 90.95 | 1.61 | 0.02 |
| AurB/C (Ab-202/175) | 127 | 120 | 135 | 124 | 129.5 | 121.83 | 125.32 | 123.57 | 2.47 | 0.02 |
| AXL (Phospho-Tyr691) | 103 | 98 | 135 | 124 | 129.5 | 98.80 | 102.35 | 100.58 | 2.51 | 0.02 |
| BAD (Ab-112) | 138 | 126 | 135 | 124 | 129.5 | 132.38 | 131.59 | 131.98 | 0.56 | 0.00 |
| BAD (Ab-134) | 107 | 96 | 135 | 124 | 129.5 | 102.64 | 100.26 | 101.45 | 1.68 | 0.02 |
| BAD (Ab-136) | 109 | 93 | 135 | 124 | 129.5 | 104.56 | 97.13 | 100.84 | 5.26 | 0.05 |
| BAD (Ab-155) | 104 | 87 | 135 | 124 | 129.5 | 99.76 | 90.86 | 95.31 | 6.30 | 0.07 |
| BAD (Ab-91/128) | 233 | 225 | 135 | 124 | 129.5 | 223.51 | 234.98 | 229.24 | 8.11 | 0.04 |
| BAD (Phospho-Ser112) | 139 | 135 | 135 | 124 | 129.5 | 133.34 | 140.99 | 137.16 | 5.41 | 0.04 |
| BAD (Phospho-Ser134) | 99 | 93 | 135 | 124 | 129.5 | 94.97 | 97.13 | 96.05 | 1.53 | 0.02 |
| BAD (Phospho-Ser136) | 107 | 94 | 135 | 124 | 129.5 | 102.64 | 98.17 | 100.41 | 3.16 | 0.03 |
| BAD (Phospho-Ser155) | 102 | 93 | 135 | 124 | 129.5 | 97.84 | 97.13 | 97.48 | 0.51 | 0.01 |
| BAD (Phospho-Ser91/128) | 92 | 103 | 135 | 124 | 129.5 | 88.25 | 107.57 | 97.91 | 13.66 | 0.14 |
| BAX (Ab-167) | 126 | 107 | 135 | 124 | 129.5 | 120.87 | 111.75 | 116.31 | 6.45 | 0.06 |
| BAX (Phospho-Thr167) | 85 | 71 | 135 | 124 | 129.5 | 81.54 | 74.15 | 77.84 | 5.22 | 0.07 |
| BCL-2 (Ab-56) | 124 | 122 | 135 | 124 | 129.5 | 118.95 | 127.41 | 123.18 | 5.98 | 0.05 |
| BCL-2 (Ab-69) | 87 | 71 | 135 | 124 | 129.5 | 83.46 | 74.15 | 78.80 | 6.58 | 0.08 |
| BCL-2 (Ab-70) | 91 | 78 | 135 | 124 | 129.5 | 87.29 | 81.46 | 84.38 | 4.12 | 0.05 |
| BCL-2 (Phospho-Ser70) | 234 | 94 | 135 | 124 | 129.5 | 224.47 | 98.17 | 161.32 | 89.31 | 0.55 |
| BCL-2 (Phospho-Ser87) | 84 | 245 | 135 | 124 | 129.5 | 80.58 | 255.87 | 168.22 | 123.95 | 0.74 |
| BCL-2 (Phospho-Thr56) | 188 | 121 | 135 | 124 | 129.5 | 180.34 | 126.37 | 153.35 | 38.17 | 0.25 |
| BCL-2 (Phospho-Thr69) | 129 | 200 | 135 | 124 | 129.5 | 123.74 | 208.87 | 166.31 | 60.19 | 0.36 |
| BCL-6 (Ab-333) | 270 | 331 | 135 | 124 | 129.5 | 259.00 | 345.68 | 302.34 | 61.29 | 0.20 |
| BCL-XL (Ab-47) | 1304 | 1217 | 135 | 124 | 129.5 | 1250.87 | 1270.98 | 1260.93 | 14.22 | 0.01 |
| BCL-XL (Phospho-Ser62) | 511 | 298 | 135 | 124 | 129.5 | 490.18 | 311.22 | 400.70 | 126.55 | 0.32 |
| BCL-XL (Phospho-Thr47) | 89 | 80 | 135 | 124 | 129.5 | 85.37 | 83.55 | 84.46 | 1.29 | 0.02 |
| BCR (Ab-177) | 150 | 141 | 135 | 124 | 129.5 | 143.89 | 147.25 | 145.57 | 2.38 | 0.02 |
| BCR (Ab-360) | 83 | 80 | 135 | 124 | 129.5 | 79.62 | 83.55 | 81.58 | 2.78 | 0.03 |
| BCR (Phospho-Tyr177) | 637 | 109 | 135 | 124 | 129.5 | 611.05 | 113.83 | 362.44 | 351.58 | 0.97 |
| BCR (Phospho-Tyr360) | 161 | 131 | 135 | 124 | 129.5 | 154.44 | 136.81 | 145.63 | 12.47 | 0.09 |
| Beta actin | 1710 | 1718 | 135 | 124 | 129.5 | 1640.33 | 1794.20 | 1717.27 | 108.80 | 0.06 |
| BID (Ab-78) | 180 | 226 | 135 | 124 | 129.5 | 172.67 | 236.02 | 204.35 | 44.80 | 0.22 |
| BID (Phospho-Ser78) | 106 | 76 | 135 | 124 | 129.5 | 101.68 | 79.37 | 90.53 | 15.78 | 0.17 |
| BIM (Ab-69/65) | 136 | 114 | 135 | 124 | 129.5 | 130.46 | 119.06 | 124.76 | 8.06 | 0.06 |
| BIM (Phospho-Ser69/65) | 158 | 412 | 135 | 124 | 129.5 | 151.56 | 430.27 | 290.92 | 197.08 | 0.68 |
| BLNK (Ab-96) | 865 | 777 | 135 | 124 | 129.5 | 829.76 | 811.46 | 820.61 | 12.94 | 0.02 |
| BLNK (Phospho-Tyr84) | 99 | 100 | 135 | 124 | 129.5 | 94.97 | 104.44 | 99.70 | 6.70 | 0.07 |
| BLNK (Phospho-Tyr96) | 295 | 369 | 135 | 124 | 129.5 | 282.98 | 385.37 | 334.17 | 72.40 | 0.22 |
| B-RAF (Ab-446) | 92 | 80 | 135 | 124 | 129.5 | 88.25 | 83.55 | 85.90 | 3.33 | 0.04 |
| B-RAF (Ab-598) | 1754 | 616 | 135 | 124 | 129.5 | 1682.54 | 643.32 | 1162.93 | 734.84 | 0.63 |
| B-RAF (Ab-601) | 96 | 72 | 135 | 124 | 129.5 | 92.09 | 75.19 | 83.64 | 11.95 | 0.14 |
| B-RAF (Phospho-Ser446) | 134 | 92 | 135 | 124 | 129.5 | 128.54 | 96.08 | 112.31 | 22.95 | 0.20 |
| B-RAF (Phospho-Ser601) | 89 | 94 | 135 | 124 | 129.5 | 85.37 | 98.17 | 91.77 | 9.05 | 0.10 |
| B-RAF (Phospho-Thr598) | 94 | 88 | 135 | 124 | 129.5 | 90.17 | 91.90 | 91.04 | 1.23 | 0.01 |
| BRCA1 (Ab-1457) | 341 | 98 | 135 | 124 | 129.5 | 327.11 | 102.35 | 214.73 | 158.93 | 0.74 |
| BRCA1 (Ab-1524) | 361 | 356 | 135 | 124 | 129.5 | 346.29 | 371.79 | 359.04 | 18.03 | 0.05 |
| BRCA1 (Phospho-Ser1423) | 652 | 821 | 135 | 124 | 129.5 | 625.44 | 857.42 | 741.43 | 164.03 | 0.22 |
| BRCA1 (Phospho-Ser1457) | 362 | 259 | 135 | 124 | 129.5 | 347.25 | 270.49 | 308.87 | 54.28 | 0.18 |
| BRCA1 (Phospho-Ser1524) | 164 | 136 | 135 | 124 | 129.5 | 157.32 | 142.03 | 149.68 | 10.81 | 0.07 |
| Breast tumor kinase (Phospho-Tyr447) | 105 | 93 | 135 | 124 | 129.5 | 100.72 | 97.13 | 98.92 | 2.54 | 0.03 |
| BTK (Ab-223) | 270 | 249 | 135 | 124 | 129.5 | 259.00 | 260.04 | 259.52 | 0.74 | 0.00 |
| BTK (Phospho-Tyr223) | 139 | 141 | 135 | 124 | 129.5 | 133.34 | 147.25 | 140.30 | 9.84 | 0.07 |
| BTK (Phospho-Tyr550) | 469 | 76 | 135 | 124 | 129.5 | 449.89 | 79.37 | 264.63 | 262.00 | 0.99 |
| c-Abl (Ab-412) | 242 | 169 | 135 | 124 | 129.5 | 232.14 | 176.50 | 204.32 | 39.35 | 0.19 |
| c-Abl (Phospho-Tyr245) | 186 | 89 | 135 | 124 | 129.5 | 178.42 | 92.95 | 135.68 | 60.44 | 0.45 |
| c-Abl (Phospho-Tyr412) | 272 | 173 | 135 | 124 | 129.5 | 260.92 | 180.67 | 220.80 | 56.74 | 0.26 |
| Calmodulin (Ab-79/81) | 93 | 198 | 135 | 124 | 129.5 | 89.21 | 206.78 | 148.00 | 83.14 | 0.56 |
| Calmodulin (Phospho-Thr79/Ser81) | 84 | 80 | 135 | 124 | 129.5 | 80.58 | 83.55 | 82.06 | 2.10 | 0.03 |
| Calsenilin/KCNIP3 (Ab-63) | 1504 | 1328 | 135 | 124 | 129.5 | 1442.73 | 1386.90 | 1414.81 | 39.47 | 0.03 |
| Calsenilin/KCNIP3 (Phospho-Ser63) | 654 | 635 | 135 | 124 | 129.5 | 627.36 | 663.17 | 645.26 | 25.32 | 0.04 |
| CaMK1-alpha (Ab-177) | 80 | 69 | 135 | 124 | 129.5 | 76.74 | 72.06 | 74.40 | 3.31 | 0.04 |
| CaMK1-alpha (Phospho-Thr177) | 112 | 98 | 135 | 124 | 129.5 | 107.44 | 102.35 | 104.89 | 3.60 | 0.03 |
| CaMK2 alpha/beta/delta (Phospho-Thr305) | 154 | 140 | 135 | 124 | 129.5 | 147.73 | 146.21 | 146.97 | 1.07 | 0.01 |
| CaMK2A (Ab-286) | 181 | 164 | 135 | 124 | 129.5 | 173.63 | 171.27 | 172.45 | 1.66 | 0.01 |
| CaMK2A (Phospho-Thr286) | 96 | 87 | 135 | 124 | 129.5 | 92.09 | 90.86 | 91.47 | 0.87 | 0.01 |
| CaMK2-beta/gamma/delta (Ab-287) | 430 | 349 | 135 | 124 | 129.5 | 412.48 | 364.48 | 388.48 | 33.94 | 0.09 |
| CaMK2-beta/gamma/delta (Phospho-Thr287) | 192 | 198 | 135 | 124 | 129.5 | 184.18 | 206.78 | 195.48 | 15.98 | 0.08 |
| CaMK4 (Ab-196/200) | 85 | 73 | 135 | 124 | 129.5 | 81.54 | 76.24 | 78.89 | 3.75 | 0.05 |
| CaMK4 (Phospho-Thr196/200) | 88 | 75 | 135 | 124 | 129.5 | 84.41 | 78.33 | 81.37 | 4.31 | 0.05 |
| Caspase 1 (Ab-376) | 173 | 146 | 135 | 124 | 129.5 | 165.95 | 152.48 | 159.21 | 9.53 | 0.06 |
| Caspase 1 (Phospho-Ser376) | 159 | 136 | 135 | 124 | 129.5 | 152.52 | 142.03 | 147.28 | 7.42 | 0.05 |
| Caspase 2 (Ab-157) | 197 | 195 | 135 | 124 | 129.5 | 188.97 | 203.65 | 196.31 | 10.38 | 0.05 |
| Caspase 2 (Phospho-Ser157) | 210 | 158 | 135 | 124 | 129.5 | 201.44 | 165.01 | 183.23 | 25.76 | 0.14 |
| Caspase 3 (Ab-150) | 77 | 68 | 135 | 124 | 129.5 | 73.86 | 71.02 | 72.44 | 2.01 | 0.03 |
| Caspase 3 (Phospho-Ser150) | 72 | 92 | 135 | 124 | 129.5 | 69.07 | 96.08 | 82.57 | 19.10 | 0.23 |
| Caspase 6 (Ab-257) | 122 | 139 | 135 | 124 | 129.5 | 117.03 | 145.17 | 131.10 | 19.89 | 0.15 |
| Caspase 6 (Phospho-Ser257) | 228 | 80 | 135 | 124 | 129.5 | 218.71 | 83.55 | 151.13 | 95.57 | 0.63 |
| Caspase 8 (Ab-347) | 119 | 117 | 135 | 124 | 129.5 | 114.15 | 122.19 | 118.17 | 5.68 | 0.05 |
| Caspase 8 (Phospho-Ser347) | 78 | 75 | 135 | 124 | 129.5 | 74.82 | 78.33 | 76.57 | 2.48 | 0.03 |
| Caspase 9 (Ab-125) | 306 | 311 | 135 | 124 | 129.5 | 293.53 | 324.79 | 309.16 | 22.10 | 0.07 |
| Caspase 9 (Ab-144) | 72 | 61 | 135 | 124 | 129.5 | 69.07 | 63.71 | 66.39 | 3.79 | 0.06 |
| Caspase 9 (Ab-153) | 169 | 149 | 135 | 124 | 129.5 | 162.11 | 155.61 | 158.86 | 4.60 | 0.03 |
| Caspase 9 (Ab-196) | 1242 | 1171 | 135 | 124 | 129.5 | 1191.40 | 1222.94 | 1207.17 | 22.30 | 0.02 |
| Caspase 9 (Phospho-Ser144) | 260 | 87 | 135 | 124 | 129.5 | 249.41 | 90.86 | 170.13 | 112.11 | 0.66 |
| Caspase 9 (Phospho-Ser196) | 736 | 642 | 135 | 124 | 129.5 | 706.01 | 670.48 | 688.25 | 25.13 | 0.04 |
| Caspase 9 (Phospho-Thr125) | 76 | 71 | 135 | 124 | 129.5 | 72.90 | 74.15 | 73.53 | 0.88 | 0.01 |
| Caspase 9 (Phospho-Tyr153) | 368 | 122 | 135 | 124 | 129.5 | 353.01 | 127.41 | 240.21 | 159.52 | 0.66 |
| Catalase (Ab-385) | 807 | 664 | 135 | 124 | 129.5 | 774.12 | 693.45 | 733.79 | 57.04 | 0.08 |
| Catalase (Phospho-Tyr385) | 142 | 147 | 135 | 124 | 129.5 | 136.21 | 153.52 | 144.87 | 12.24 | 0.08 |
| Catenin beta (Ab-33) | 87 | 74 | 135 | 124 | 129.5 | 83.46 | 77.28 | 80.37 | 4.37 | 0.05 |
| Catenin beta (Ab-37) | 295 | 264 | 135 | 124 | 129.5 | 282.98 | 275.71 | 279.35 | 5.14 | 0.02 |
| Catenin beta (Ab-41/45) | 183 | 143 | 135 | 124 | 129.5 | 175.54 | 149.34 | 162.44 | 18.53 | 0.11 |
| Catenin beta (Ab-489) | 84 | 76 | 135 | 124 | 129.5 | 80.58 | 79.37 | 79.97 | 0.85 | 0.01 |
| Catenin beta (Ab-654) | 170 | 168 | 135 | 124 | 129.5 | 163.07 | 175.45 | 169.26 | 8.75 | 0.05 |
| Catenin beta (Phospho-Ser33) | 274 | 242 | 135 | 124 | 129.5 | 262.84 | 252.73 | 257.79 | 7.14 | 0.03 |
| Catenin beta (Phospho-Ser37) | 114 | 95 | 135 | 124 | 129.5 | 109.36 | 99.21 | 104.28 | 7.17 | 0.07 |
| Catenin beta (Phospho-Thr41/Ser45) | 130 | 121 | 135 | 124 | 129.5 | 124.70 | 126.37 | 125.54 | 1.18 | 0.01 |
| Catenin beta (Phospho-Tyr489) | 94 | 78 | 135 | 124 | 129.5 | 90.17 | 81.46 | 85.82 | 6.16 | 0.07 |
| Catenin beta (Phospho-Tyr654) | 92 | 84 | 135 | 124 | 129.5 | 88.25 | 87.73 | 87.99 | 0.37 | 0.00 |
| Catenin delta-1 (Ab-228) | 164 | 161 | 135 | 124 | 129.5 | 157.32 | 168.14 | 162.73 | 7.65 | 0.05 |
| Catenin delta-1 (Phospho-Tyr228) | 305 | 267 | 135 | 124 | 129.5 | 292.57 | 278.84 | 285.71 | 9.71 | 0.03 |
| Caveolin-1 (Ab-14) | 102 | 94 | 135 | 124 | 129.5 | 97.84 | 98.17 | 98.01 | 0.23 | 0.00 |
| Caveolin-1 (Phospho-Tyr14) | 124 | 108 | 135 | 124 | 129.5 | 118.95 | 112.79 | 115.87 | 4.35 | 0.04 |
| CBL (Phospho-Tyr700) | 118 | 351 | 135 | 124 | 129.5 | 113.19 | 366.57 | 239.88 | 179.16 | 0.75 |
| CBL (Phospho-Tyr774) | 279 | 78 | 135 | 124 | 129.5 | 267.63 | 81.46 | 174.55 | 131.64 | 0.75 |
| CD19 (Ab-531) | 137 | 64 | 135 | 124 | 129.5 | 131.42 | 66.84 | 99.13 | 45.66 | 0.46 |
| CD19 (Phospho-Tyr531) | 779 | 143 | 135 | 124 | 129.5 | 747.26 | 149.34 | 448.30 | 422.79 | 0.94 |
| CD22/BL-CAM (Phospho-Tyr807) | 195 | 92 | 135 | 124 | 129.5 | 187.06 | 96.08 | 141.57 | 64.33 | 0.45 |
| CD227/mucin 1 (Ab-1243) | 98 | 79 | 135 | 124 | 129.5 | 94.01 | 82.50 | 88.26 | 8.13 | 0.09 |
| CD227/mucin 1 (Phospho-Tyr1243) | 174 | 202 | 135 | 124 | 129.5 | 166.91 | 210.96 | 188.94 | 31.15 | 0.16 |
| CD28 (Phospho-Tyr218) | 103 | 104 | 135 | 124 | 129.5 | 98.80 | 108.61 | 103.71 | 6.94 | 0.07 |
| CD32 (FcgammaRIIb) (Ab-292) | 605 | 494 | 135 | 124 | 129.5 | 580.35 | 515.91 | 548.13 | 45.57 | 0.08 |
| CD3Z (Ab-142) | 196 | 153 | 135 | 124 | 129.5 | 188.01 | 159.79 | 173.90 | 19.96 | 0.11 |
| CD3Z (Phospho-Tyr142) | 102 | 88 | 135 | 124 | 129.5 | 97.84 | 91.90 | 94.87 | 4.20 | 0.04 |
| CD4 (Ab-433) | 1210 | 906 | 135 | 124 | 129.5 | 1160.70 | 946.19 | 1053.44 | 151.69 | 0.14 |
| CD4 (Phospho-Ser433) | 160 | 343 | 135 | 124 | 129.5 | 153.48 | 358.21 | 255.85 | 144.77 | 0.57 |
| CD45 (Phospho-Ser1007) | 93 | 336 | 135 | 124 | 129.5 | 89.21 | 350.90 | 220.06 | 185.04 | 0.84 |
| CD5 (Ab-453) | 488 | 453 | 135 | 124 | 129.5 | 468.12 | 473.09 | 470.61 | 3.52 | 0.01 |
| CD5 (Phospho-Tyr453) | 150 | 133 | 135 | 124 | 129.5 | 143.89 | 138.90 | 141.39 | 3.53 | 0.02 |
| CDC25A (Ab-124) | 77 | 68 | 135 | 124 | 129.5 | 73.86 | 71.02 | 72.44 | 2.01 | 0.03 |
| CDC25A (Ab-178) | 126 | 145 | 135 | 124 | 129.5 | 120.87 | 151.43 | 136.15 | 21.61 | 0.16 |
| CDC25A (Ab-75) | 258 | 260 | 135 | 124 | 129.5 | 247.49 | 271.53 | 259.51 | 17.00 | 0.07 |
| CDC25A (Phospho-Ser124) | 164 | 134 | 135 | 124 | 129.5 | 157.32 | 139.94 | 148.63 | 12.29 | 0.08 |
| CDC25A (Phospho-Ser75) | 116 | 106 | 135 | 124 | 129.5 | 111.27 | 110.70 | 110.99 | 0.40 | 0.00 |
| CDC25B (Ab-323) | 87 | 72 | 135 | 124 | 129.5 | 83.46 | 75.19 | 79.32 | 5.84 | 0.07 |
| CDC25B (Ab-353) | 75 | 64 | 135 | 124 | 129.5 | 71.94 | 66.84 | 69.39 | 3.61 | 0.05 |
| CDC25B (Phospho-Ser323) | 103 | 115 | 135 | 124 | 129.5 | 98.80 | 120.10 | 109.45 | 15.06 | 0.14 |
| CDC25B (Phospho-Ser353) | 365 | 305 | 135 | 124 | 129.5 | 350.13 | 318.53 | 334.33 | 22.35 | 0.07 |
| CDC25C (Ab-216) | 2100 | 1953 | 135 | 124 | 129.5 | 2014.44 | 2039.63 | 2027.03 | 17.81 | 0.01 |
| CDC25C (Phospho-Ser216) | 221 | 140 | 135 | 124 | 129.5 | 212.00 | 146.21 | 179.10 | 46.52 | 0.26 |
| CDC25C (Phospho-Thr48) | 101 | 126 | 135 | 124 | 129.5 | 96.89 | 131.59 | 114.24 | 24.54 | 0.21 |
| CDK1/CDC2 (Ab-14) | 107 | 86 | 135 | 124 | 129.5 | 102.64 | 89.81 | 96.23 | 9.07 | 0.09 |
| CDK1/CDC2 (Ab-15) | 101 | 81 | 135 | 124 | 129.5 | 96.89 | 84.59 | 90.74 | 8.69 | 0.10 |
| CDK1/CDC2 (Phospho-Thr14) | 169 | 163 | 135 | 124 | 129.5 | 162.11 | 170.23 | 166.17 | 5.74 | 0.03 |
| CDK1/CDC2 (Phospho-Tyr15) | 195 | 212 | 135 | 124 | 129.5 | 187.06 | 221.40 | 204.23 | 24.29 | 0.12 |
| CDK2 (Ab-160) | 140 | 102 | 135 | 124 | 129.5 | 134.30 | 106.52 | 120.41 | 19.64 | 0.16 |
| CDK2 (Phospho-Thr160) | 249 | 99 | 135 | 124 | 129.5 | 238.86 | 103.39 | 171.12 | 95.79 | 0.56 |
| CDK5 (Ab-15) | 1428 | 1110 | 135 | 124 | 129.5 | 1369.82 | 1159.23 | 1264.53 | 148.91 | 0.12 |
| CDK5 (Phospho-Tyr15) | 260 | 167 | 135 | 124 | 129.5 | 249.41 | 174.41 | 211.91 | 53.03 | 0.25 |
| CDK7 (Ab-170) | 89 | 77 | 135 | 124 | 129.5 | 85.37 | 80.42 | 82.89 | 3.51 | 0.04 |
| CDK7 (Phospho-Thr170) | 402 | 422 | 135 | 124 | 129.5 | 385.62 | 440.72 | 413.17 | 38.96 | 0.09 |
| Chk1 (Ab-280) | 89 | 102 | 135 | 124 | 129.5 | 85.37 | 106.52 | 95.95 | 14.96 | 0.16 |
| Chk1 (Ab-286) | 498 | 572 | 135 | 124 | 129.5 | 477.71 | 597.37 | 537.54 | 84.61 | 0.16 |
| Chk1 (Ab-317) | 390 | 356 | 135 | 124 | 129.5 | 374.11 | 371.79 | 372.95 | 1.64 | 0.00 |
| Chk1 (Ab-345) | 130 | 118 | 135 | 124 | 129.5 | 124.70 | 123.23 | 123.97 | 1.04 | 0.01 |
| Chk1 (Phospho-Ser280) | 110 | 144 | 135 | 124 | 129.5 | 105.52 | 150.39 | 127.95 | 31.73 | 0.25 |
| Chk1 (Phospho-Ser286) | 241 | 82 | 135 | 124 | 129.5 | 231.18 | 85.64 | 158.41 | 102.92 | 0.65 |
| Chk1 (Phospho-Ser296) | 175 | 168 | 135 | 124 | 129.5 | 167.87 | 175.45 | 171.66 | 5.36 | 0.03 |
| Chk1 (Phospho-Ser301) | 89 | 64 | 135 | 124 | 129.5 | 85.37 | 66.84 | 76.11 | 13.11 | 0.17 |
| Chk1 (Phospho-Ser317) | 89 | 101 | 135 | 124 | 129.5 | 85.37 | 105.48 | 95.43 | 14.22 | 0.15 |
| Chk1 (Phospho-Ser345) | 167 | 91 | 135 | 124 | 129.5 | 160.20 | 95.04 | 127.62 | 46.08 | 0.36 |
| Chk2 (Ab-383) | 184 | 184 | 135 | 124 | 129.5 | 176.50 | 192.16 | 184.33 | 11.07 | 0.06 |
| Chk2 (Ab-387) | 354 | 216 | 135 | 124 | 129.5 | 339.58 | 225.58 | 282.58 | 80.61 | 0.29 |
| Chk2 (Ab-516) | 258 | 256 | 135 | 124 | 129.5 | 247.49 | 267.35 | 257.42 | 14.05 | 0.05 |
| Chk2 (Ab-68) | 1344 | 1254 | 135 | 124 | 129.5 | 1289.24 | 1309.62 | 1299.43 | 14.41 | 0.01 |
| Chk2 (Phospho-Ser516) | 142 | 128 | 135 | 124 | 129.5 | 136.21 | 133.68 | 134.95 | 1.79 | 0.01 |
| Chk2 (Phospho-Thr383) | 261 | 77 | 135 | 124 | 129.5 | 250.37 | 80.42 | 165.39 | 120.17 | 0.73 |
| Chk2 (Phospho-Thr387) | 282 | 76 | 135 | 124 | 129.5 | 270.51 | 79.37 | 174.94 | 135.16 | 0.77 |
| Chk2 (Phospho-Thr68) | 333 | 402 | 135 | 124 | 129.5 | 319.43 | 419.83 | 369.63 | 70.99 | 0.19 |
| c-Jun (Ab-170) | 218 | 87 | 135 | 124 | 129.5 | 209.12 | 90.86 | 149.99 | 83.62 | 0.56 |
| c-Jun (Ab-239) | 224 | 159 | 135 | 124 | 129.5 | 214.87 | 166.05 | 190.46 | 34.52 | 0.18 |
| c-Jun (Ab-243) | 132 | 117 | 135 | 124 | 129.5 | 126.62 | 122.19 | 124.41 | 3.13 | 0.03 |
| c-Jun (Ab-63) | 202 | 166 | 135 | 124 | 129.5 | 193.77 | 173.36 | 183.57 | 14.43 | 0.08 |
| c-Jun (Ab-91) | 105 | 107 | 135 | 124 | 129.5 | 100.72 | 111.75 | 106.23 | 7.79 | 0.07 |
| c-Jun (Ab-93) | 94 | 78 | 135 | 124 | 129.5 | 90.17 | 81.46 | 85.82 | 6.16 | 0.07 |
| c-Jun (Phospho-Ser243) | 170 | 193 | 135 | 124 | 129.5 | 163.07 | 201.56 | 182.32 | 27.21 | 0.15 |
| c-Jun (Phospho-Ser63) | 214 | 105 | 135 | 124 | 129.5 | 205.28 | 109.66 | 157.47 | 67.62 | 0.43 |
| c-Jun (Phospho-Ser73) | 303 | 231 | 135 | 124 | 129.5 | 290.66 | 241.25 | 265.95 | 34.94 | 0.13 |
| c-Jun (Phospho-Thr239) | 127 | 116 | 135 | 124 | 129.5 | 121.83 | 121.15 | 121.49 | 0.48 | 0.00 |
| c-Jun (Phospho-Thr91) | 179 | 128 | 135 | 124 | 129.5 | 171.71 | 133.68 | 152.69 | 26.89 | 0.18 |
| c-Jun (Phospho-Thr93) | 141 | 178 | 135 | 124 | 129.5 | 135.26 | 185.90 | 160.58 | 35.81 | 0.22 |
| c-Jun (Phospho-Tyr170) | 108 | 189 | 135 | 124 | 129.5 | 103.60 | 197.38 | 150.49 | 66.31 | 0.44 |
| CK1-A (Ab-321) | 90 | 83 | 135 | 124 | 129.5 | 86.33 | 86.68 | 86.51 | 0.25 | 0.00 |
| CK1-A (Phospho-Thr321) | 321 | 321 | 135 | 124 | 129.5 | 307.92 | 335.24 | 321.58 | 19.32 | 0.06 |
| CK1-A/A2 (Phospho-Tyr294) | 110 | 92 | 135 | 124 | 129.5 | 105.52 | 96.08 | 100.80 | 6.67 | 0.07 |
| CK2-b (Ab-209) | 98 | 92 | 135 | 124 | 129.5 | 94.01 | 96.08 | 95.04 | 1.47 | 0.02 |
| CK2-b (Phospho-Ser209) | 107 | 96 | 135 | 124 | 129.5 | 102.64 | 100.26 | 101.45 | 1.68 | 0.02 |
| Claudin 3 (Ab-219) | 91 | 128 | 135 | 124 | 129.5 | 87.29 | 133.68 | 110.49 | 32.80 | 0.30 |
| Claudin 3 (Phospho-Tyr219) | 265 | 270 | 135 | 124 | 129.5 | 254.20 | 281.98 | 268.09 | 19.64 | 0.07 |
| Claudin 6 (Phospho-Tyr219) | 136 | 122 | 135 | 124 | 129.5 | 130.46 | 127.41 | 128.94 | 2.16 | 0.02 |
| Claudin 7 (Ab-210) | 95 | 84 | 135 | 124 | 129.5 | 91.13 | 87.73 | 89.43 | 2.41 | 0.03 |
| Claudin 7 (Phospho-Tyr210) | 219 | 212 | 135 | 124 | 129.5 | 210.08 | 221.40 | 215.74 | 8.01 | 0.04 |
| Coagulation Factor III (Phospho-Ser290) | 99 | 158 | 135 | 124 | 129.5 | 94.97 | 165.01 | 129.99 | 49.53 | 0.38 |
| Cofilin (Ab-3) | 122 | 103 | 135 | 124 | 129.5 | 117.03 | 107.57 | 112.30 | 6.69 | 0.06 |
| Cofilin (Phospho-Ser3) | 141 | 124 | 135 | 124 | 129.5 | 135.26 | 129.50 | 132.38 | 4.07 | 0.03 |
| Connexin 43 (Ab-367) | 261 | 205 | 135 | 124 | 129.5 | 250.37 | 214.09 | 232.23 | 25.65 | 0.11 |
| Connexin 43 (Phospho-Ser367) | 382 | 348 | 135 | 124 | 129.5 | 366.44 | 363.44 | 364.94 | 2.12 | 0.01 |
| Cortactin (Ab-421) | 91 | 79 | 135 | 124 | 129.5 | 87.29 | 82.50 | 84.90 | 3.39 | 0.04 |
| Cortactin (Ab-466) | 148 | 129 | 135 | 124 | 129.5 | 141.97 | 134.72 | 138.35 | 5.13 | 0.04 |
| Cortactin (Phospho-Tyr421) | 138 | 125 | 135 | 124 | 129.5 | 132.38 | 130.54 | 131.46 | 1.30 | 0.01 |
| Cortactin (Phospho-Tyr466) | 121 | 102 | 135 | 124 | 129.5 | 116.07 | 106.52 | 111.30 | 6.75 | 0.06 |
| CPI17 alpha (Ab-38) | 131 | 117 | 135 | 124 | 129.5 | 125.66 | 122.19 | 123.93 | 2.46 | 0.02 |
| CPI17 alpha (Phospho-Thr38) | 123 | 115 | 135 | 124 | 129.5 | 117.99 | 120.10 | 119.04 | 1.49 | 0.01 |
| c-PLA2 (Ab-505) | 90 | 74 | 135 | 124 | 129.5 | 86.33 | 77.28 | 81.81 | 6.40 | 0.08 |
| c-PLA2 (Phospho-Ser505) | 87 | 311 | 135 | 124 | 129.5 | 83.46 | 324.79 | 204.12 | 170.65 | 0.84 |
| CREB (Ab-100) | 89 | 84 | 135 | 124 | 129.5 | 85.37 | 87.73 | 86.55 | 1.66 | 0.02 |
| CREB (Ab-121) | 113 | 106 | 135 | 124 | 129.5 | 108.40 | 110.70 | 109.55 | 1.63 | 0.01 |
| CREB (Ab-129) | 79 | 68 | 135 | 124 | 129.5 | 75.78 | 71.02 | 73.40 | 3.37 | 0.05 |
| CREB (Ab-133) | 97 | 78 | 135 | 124 | 129.5 | 93.05 | 81.46 | 87.25 | 8.19 | 0.09 |
| CREB (Ab-142) | 208 | 129 | 135 | 124 | 129.5 | 199.53 | 134.72 | 167.12 | 45.82 | 0.27 |
| CREB (Phospho-Ser121) | 181 | 139 | 135 | 124 | 129.5 | 173.63 | 145.17 | 159.40 | 20.12 | 0.13 |
| CREB (Phospho-Ser129) | 317 | 142 | 135 | 124 | 129.5 | 304.09 | 148.30 | 226.19 | 110.16 | 0.49 |
| CREB (Phospho-Ser133) | 101 | 356 | 135 | 124 | 129.5 | 96.89 | 371.79 | 234.34 | 194.39 | 0.83 |
| CREB (Phospho-Ser142) | 119 | 92 | 135 | 124 | 129.5 | 114.15 | 96.08 | 105.12 | 12.78 | 0.12 |
| CREB (Phospho-Thr100) | 188 | 92 | 135 | 124 | 129.5 | 180.34 | 96.08 | 138.21 | 59.58 | 0.43 |
| CrkII (Ab-221) | 464 | 368 | 135 | 124 | 129.5 | 445.10 | 384.32 | 414.71 | 42.97 | 0.10 |
| CrkII (Phospho-Tyr221) | 252 | 354 | 135 | 124 | 129.5 | 241.73 | 369.70 | 305.72 | 90.49 | 0.30 |
| CrkL (Phospho-Tyr207) | 92 | 92 | 135 | 124 | 129.5 | 88.25 | 96.08 | 92.17 | 5.54 | 0.06 |
| CXCR4 (Phospho-Ser339) | 76 | 63 | 135 | 124 | 129.5 | 72.90 | 65.79 | 69.35 | 5.03 | 0.07 |
| Cyclin B1 (Ab-126) | 130 | 114 | 135 | 124 | 129.5 | 124.70 | 119.06 | 121.88 | 3.99 | 0.03 |
| Cyclin B1 (Ab-147) | 96 | 76 | 135 | 124 | 129.5 | 92.09 | 79.37 | 85.73 | 8.99 | 0.10 |
| Cyclin B1 (Phospho-Ser126) | 168 | 101 | 135 | 124 | 129.5 | 161.16 | 105.48 | 133.32 | 39.37 | 0.30 |
| Cyclin B1 (Phospho-Ser147) | 104 | 83 | 135 | 124 | 129.5 | 99.76 | 86.68 | 93.22 | 9.25 | 0.10 |
| Cyclin C (Phospho-Ser275) | 114 | 110 | 135 | 124 | 129.5 | 109.36 | 114.88 | 112.12 | 3.91 | 0.03 |
| Cyclin D1 (Ab-286) | 1009 | 1081 | 135 | 124 | 129.5 | 967.89 | 1128.95 | 1048.42 | 113.88 | 0.11 |
| Cyclin D1 (Ab-90) | 126 | 114 | 135 | 124 | 129.5 | 120.87 | 119.06 | 119.96 | 1.28 | 0.01 |
| Cyclin D1 (Phospho-Thr286) | 445 | 408 | 135 | 124 | 129.5 | 426.87 | 426.10 | 426.48 | 0.55 | 0.00 |
| Cyclin D2 (Ab-280) | 467 | 89 | 135 | 124 | 129.5 | 447.97 | 92.95 | 270.46 | 251.04 | 0.93 |
| Cyclin D3 (Ab-283) | 345 | 384 | 135 | 124 | 129.5 | 330.94 | 401.03 | 365.99 | 49.56 | 0.14 |
| Cyclin D3 (Phospho-Thr283) | 86 | 66 | 135 | 124 | 129.5 | 82.50 | 68.93 | 75.71 | 9.59 | 0.13 |
| Cyclin E1 (Ab-395) | 123 | 109 | 135 | 124 | 129.5 | 117.99 | 113.83 | 115.91 | 2.94 | 0.03 |
| Cyclin E1 (Ab-77) | 1687 | 1519 | 135 | 124 | 129.5 | 1618.27 | 1586.38 | 1602.32 | 22.55 | 0.01 |
| Cyclin E1 (Phospho-Thr395) | 129 | 118 | 135 | 124 | 129.5 | 123.74 | 123.23 | 123.49 | 0.36 | 0.00 |
| Cyclin E1 (Phospho-Thr77) | 494 | 460 | 135 | 124 | 129.5 | 473.87 | 480.40 | 477.14 | 4.62 | 0.01 |
| Cyclin E2 (Ab-392) | 145 | 120 | 135 | 124 | 129.5 | 139.09 | 125.32 | 132.21 | 9.74 | 0.07 |
| Cyclin E2 (Phospho-Thr392) | 91 | 76 | 135 | 124 | 129.5 | 87.29 | 79.37 | 83.33 | 5.60 | 0.07 |
| DAB1 (Ab-220) | 130 | 117 | 135 | 124 | 129.5 | 124.70 | 122.19 | 123.45 | 1.78 | 0.01 |
| DAB1 (Ab-232) | 89 | 75 | 135 | 124 | 129.5 | 85.37 | 78.33 | 81.85 | 4.98 | 0.06 |
| DAB1 (Phospho-Tyr220) | 193 | 125 | 135 | 124 | 129.5 | 185.14 | 130.54 | 157.84 | 38.60 | 0.24 |
| DAB1 (Phospho-Tyr232) | 82 | 70 | 135 | 124 | 129.5 | 78.66 | 73.10 | 75.88 | 3.93 | 0.05 |
| DAPP1 (Ab-139) | 102 | 92 | 135 | 124 | 129.5 | 97.84 | 96.08 | 96.96 | 1.25 | 0.01 |
| DAPP1 (Phospho-Tyr139) | 87 | 64 | 135 | 124 | 129.5 | 83.46 | 66.84 | 75.15 | 11.75 | 0.16 |
| DARPP-32 (Ab-34) | 214 | 435 | 135 | 124 | 129.5 | 205.28 | 454.29 | 329.79 | 176.08 | 0.53 |
| DARPP-32 (Ab-75) | 186 | 152 | 135 | 124 | 129.5 | 178.42 | 158.74 | 168.58 | 13.92 | 0.08 |
| DARPP-32 (Phospho-Thr34) | 153 | 139 | 135 | 124 | 129.5 | 146.77 | 145.17 | 145.97 | 1.13 | 0.01 |
| DARPP-32 (Phospho-Thr75) | 216 | 134 | 135 | 124 | 129.5 | 207.20 | 139.94 | 173.57 | 47.56 | 0.27 |
| DAXX (Phospho-Ser668) | 561 | 444 | 135 | 124 | 129.5 | 538.14 | 463.69 | 500.92 | 52.64 | 0.11 |
| DDX5/DEAD-box protein 5 (Ab-593) | 112 | 89 | 135 | 124 | 129.5 | 107.44 | 92.95 | 100.19 | 10.25 | 0.10 |
| DDX5/DEAD-box protein 5 (Phospho-Tyr593) | 133 | 401 | 135 | 124 | 129.5 | 127.58 | 418.79 | 273.18 | 205.91 | 0.75 |
| DNA-PK (Ab-2056) | 73 | 61 | 135 | 124 | 129.5 | 70.03 | 63.71 | 66.87 | 4.47 | 0.07 |
| DNA-PK (Ab-2638) | 143 | 132 | 135 | 124 | 129.5 | 137.17 | 137.85 | 137.51 | 0.48 | 0.00 |
| DNA-PK (Ab-2647) | 81 | 69 | 135 | 124 | 129.5 | 77.70 | 72.06 | 74.88 | 3.99 | 0.05 |
| DNA-PK (Phospho-Thr2638) | 126 | 114 | 135 | 124 | 129.5 | 120.87 | 119.06 | 119.96 | 1.28 | 0.01 |
| DNA-PK (Phospho-Thr2647) | 97 | 81 | 135 | 124 | 129.5 | 93.05 | 84.59 | 88.82 | 5.98 | 0.07 |
| Dok-1 (Ab-362) | 121 | 105 | 135 | 124 | 129.5 | 116.07 | 109.66 | 112.86 | 4.53 | 0.04 |
| Dok-1 (Ab-398) | 265 | 197 | 135 | 124 | 129.5 | 254.20 | 205.74 | 229.97 | 34.27 | 0.15 |
| Dok-1 (Phospho-Tyr362) | 107 | 96 | 135 | 124 | 129.5 | 102.64 | 100.26 | 101.45 | 1.68 | 0.02 |
| Dok-1 (Phospho-Tyr398) | 94 | 84 | 135 | 124 | 129.5 | 90.17 | 87.73 | 88.95 | 1.73 | 0.02 |
| Dok-2 (Ab-299) | 150 | 282 | 135 | 124 | 129.5 | 143.89 | 294.51 | 219.20 | 106.50 | 0.49 |
| Dok-2 (Phospho-Tyr299) | 311 | 350 | 135 | 124 | 129.5 | 298.33 | 365.52 | 331.93 | 47.51 | 0.14 |
| DYN1 (Ab-774) | 2572 | 3026 | 135 | 124 | 129.5 | 2467.21 | 3160.22 | 2813.72 | 490.03 | 0.17 |
| DYN1 (Phospho-Ser774) | 288 | 263 | 135 | 124 | 129.5 | 276.27 | 274.67 | 275.47 | 1.13 | 0.00 |
| E2F1 (Ab-433) | 383 | 312 | 135 | 124 | 129.5 | 367.40 | 325.84 | 346.62 | 29.39 | 0.08 |
| E2F1 (Phospho-Thr433) | 110 | 82 | 135 | 124 | 129.5 | 105.52 | 85.64 | 95.58 | 14.06 | 0.15 |
| EEF2 (Ab-56) | 591 | 469 | 135 | 124 | 129.5 | 566.92 | 489.80 | 528.36 | 54.53 | 0.10 |
| EEF2 (Phospho-Thr56) | 142 | 185 | 135 | 124 | 129.5 | 136.21 | 193.21 | 164.71 | 40.30 | 0.24 |
| eEF2K (Ab-366) | 449 | 339 | 135 | 124 | 129.5 | 430.71 | 354.04 | 392.37 | 54.21 | 0.14 |
| eEF2K (Phospho-Ser366) | 258 | 223 | 135 | 124 | 129.5 | 247.49 | 232.89 | 240.19 | 10.32 | 0.04 |
| EGFR (Ab-1016) | 144 | 137 | 135 | 124 | 129.5 | 138.13 | 143.08 | 140.60 | 3.50 | 0.02 |
| EGFR (Ab-1069) | 70 | 57 | 135 | 124 | 129.5 | 67.15 | 59.53 | 63.34 | 5.39 | 0.09 |
| EGFR (Ab-1070) | 764 | 822 | 135 | 124 | 129.5 | 732.87 | 858.46 | 795.67 | 88.80 | 0.11 |
| EGFR (Ab-1092) | 186 | 171 | 135 | 124 | 129.5 | 178.42 | 178.58 | 178.50 | 0.11 | 0.00 |
| EGFR (Ab-1110) | 168 | 169 | 135 | 124 | 129.5 | 161.16 | 176.50 | 168.83 | 10.85 | 0.06 |
| EGFR (Ab-1172) | 713 | 713 | 135 | 124 | 129.5 | 683.95 | 744.63 | 714.29 | 42.90 | 0.06 |
| EGFR (Ab-1197) | 158 | 121 | 135 | 124 | 129.5 | 151.56 | 126.37 | 138.96 | 17.82 | 0.13 |
| EGFR (Ab-678) | 97 | 88 | 135 | 124 | 129.5 | 93.05 | 91.90 | 92.48 | 0.81 | 0.01 |
| EGFR (Ab-693) | 352 | 345 | 135 | 124 | 129.5 | 337.66 | 360.30 | 348.98 | 16.01 | 0.05 |
| EGFR (Ab-869) | 1100 | 1290 | 135 | 124 | 129.5 | 1055.19 | 1347.22 | 1201.20 | 206.50 | 0.17 |
| EGFR (Ab-998) | 139 | 134 | 135 | 124 | 129.5 | 133.34 | 139.94 | 136.64 | 4.67 | 0.03 |
| EGFR (Phospho-Ser1070) | 118 | 100 | 135 | 124 | 129.5 | 113.19 | 104.44 | 108.81 | 6.19 | 0.06 |
| EGFR (Phospho-Thr678) | 216 | 97 | 135 | 124 | 129.5 | 207.20 | 101.30 | 154.25 | 74.88 | 0.49 |
| EGFR (Phospho-Thr693) | 202 | 200 | 135 | 124 | 129.5 | 193.77 | 208.87 | 201.32 | 10.68 | 0.05 |
| EGFR (Phospho-Tyr1016) | 267 | 228 | 135 | 124 | 129.5 | 256.12 | 238.11 | 247.12 | 12.73 | 0.05 |
| EGFR (Phospho-Tyr1069) | 132 | 151 | 135 | 124 | 129.5 | 126.62 | 157.70 | 142.16 | 21.97 | 0.15 |
| EGFR (Phospho-Tyr1092) | 113 | 101 | 135 | 124 | 129.5 | 108.40 | 105.48 | 106.94 | 2.06 | 0.02 |
| EGFR (Phospho-Tyr1110) | 121 | 263 | 135 | 124 | 129.5 | 116.07 | 274.67 | 195.37 | 112.14 | 0.57 |
| EGFR (Phospho-Tyr1172) | 104 | 84 | 135 | 124 | 129.5 | 99.76 | 87.73 | 93.74 | 8.51 | 0.09 |
| EGFR (Phospho-Tyr1197) | 387 | 108 | 135 | 124 | 129.5 | 371.23 | 112.79 | 242.01 | 182.75 | 0.76 |
| EGFR (Phospho-Tyr869) | 471 | 139 | 135 | 124 | 129.5 | 451.81 | 145.17 | 298.49 | 216.83 | 0.73 |
| eIF2A (Ab-51) | 221 | 212 | 135 | 124 | 129.5 | 212.00 | 221.40 | 216.70 | 6.65 | 0.03 |
| eIF2A (Phospho-Ser51) | 150 | 177 | 135 | 124 | 129.5 | 143.89 | 184.85 | 164.37 | 28.96 | 0.18 |
| eIF4B (Phospho-Ser422) | 82 | 67 | 135 | 124 | 129.5 | 78.66 | 69.97 | 74.32 | 6.14 | 0.08 |
| eIF4E (Ab-209) | 106 | 88 | 135 | 124 | 129.5 | 101.68 | 91.90 | 96.79 | 6.91 | 0.07 |
| eIF4E (Phospho-Ser209) | 174 | 230 | 135 | 124 | 129.5 | 166.91 | 240.20 | 203.56 | 51.82 | 0.25 |
| eIF4G (Ab-1108) | 317 | 295 | 135 | 124 | 129.5 | 304.09 | 308.08 | 306.08 | 2.83 | 0.01 |
| eIF4G (Phospho-Ser1108) | 95 | 93 | 135 | 124 | 129.5 | 91.13 | 97.13 | 94.13 | 4.24 | 0.05 |
| Elk1 (Ab-383) | 121 | 113 | 135 | 124 | 129.5 | 116.07 | 118.01 | 117.04 | 1.37 | 0.01 |
| Elk1 (Ab-389) | 249 | 169 | 135 | 124 | 129.5 | 238.86 | 176.50 | 207.68 | 44.09 | 0.21 |
| Elk1 (Ab-417) | 138 | 114 | 135 | 124 | 129.5 | 132.38 | 119.06 | 125.72 | 9.42 | 0.07 |
| Elk1 (Phospho-Ser383) | 612 | 518 | 135 | 124 | 129.5 | 587.07 | 540.98 | 564.02 | 32.59 | 0.06 |
| Elk1 (Phospho-Ser389) | 95 | 75 | 135 | 124 | 129.5 | 91.13 | 78.33 | 84.73 | 9.05 | 0.11 |
| Elk1 (Phospho-Thr417) | 567 | 115 | 135 | 124 | 129.5 | 543.90 | 120.10 | 332.00 | 299.67 | 0.90 |
| eNOS (Ab-1177) | 317 | 155 | 135 | 124 | 129.5 | 304.09 | 161.88 | 232.98 | 100.56 | 0.43 |
| eNOS (Ab-1179) | 150 | 294 | 135 | 124 | 129.5 | 143.89 | 307.04 | 225.46 | 115.37 | 0.51 |
| eNOS (Ab-495) | 89 | 74 | 135 | 124 | 129.5 | 85.37 | 77.28 | 81.33 | 5.72 | 0.07 |
| eNOS (Ab-615) | 95 | 89 | 135 | 124 | 129.5 | 91.13 | 92.95 | 92.04 | 1.29 | 0.01 |
| eNOS (Phospho-Ser1177) | 157 | 140 | 135 | 124 | 129.5 | 150.60 | 146.21 | 148.41 | 3.11 | 0.02 |
| eNOS (Phospho-Ser615) | 135 | 143 | 135 | 124 | 129.5 | 129.50 | 149.34 | 139.42 | 14.03 | 0.10 |
| eNOS (Phospho-Thr495) | 219 | 221 | 135 | 124 | 129.5 | 210.08 | 230.80 | 220.44 | 14.65 | 0.07 |
| EPB41 (Ab-418/660) | 194 | 253 | 135 | 124 | 129.5 | 186.10 | 264.22 | 225.16 | 55.24 | 0.25 |
| EPB41 (Phospho-Tyr418/660) | 159 | 166 | 135 | 124 | 129.5 | 152.52 | 173.36 | 162.94 | 14.74 | 0.09 |
| EPHA2/3/4 (Ab-588/596) | 122 | 112 | 135 | 124 | 129.5 | 117.03 | 116.97 | 117.00 | 0.04 | 0.00 |
| EPHA2/3/4 (Phospho-Tyr588/596) | 96 | 138 | 135 | 124 | 129.5 | 92.09 | 144.12 | 118.10 | 36.79 | 0.31 |
| EPHB1/2 (Ab-594/604) | 76 | 68 | 135 | 124 | 129.5 | 72.90 | 71.02 | 71.96 | 1.33 | 0.02 |
| EPHB1/2 (Phospho-Tyr594/604) | 102 | 99 | 135 | 124 | 129.5 | 97.84 | 103.39 | 100.62 | 3.92 | 0.04 |
| Ephrin B1 (Ab-317) | 84 | 76 | 135 | 124 | 129.5 | 80.58 | 79.37 | 79.97 | 0.85 | 0.01 |
| Ephrin B1 (Phospho-Tyr317) | 93 | 90 | 135 | 124 | 129.5 | 89.21 | 93.99 | 91.60 | 3.38 | 0.04 |
| Ephrin B1/B2/B3 (Phospho-Tyr324) | 96 | 84 | 135 | 124 | 129.5 | 92.09 | 87.73 | 89.91 | 3.09 | 0.03 |
| Ephrin B2 (Ab-330) | 195 | 207 | 135 | 124 | 129.5 | 187.06 | 216.18 | 201.62 | 20.60 | 0.10 |
| Ephrin B2 (Phospho-Tyr330) | 88 | 80 | 135 | 124 | 129.5 | 84.41 | 83.55 | 83.98 | 0.61 | 0.01 |
| Epo-R (Ab-368) | 127 | 110 | 135 | 124 | 129.5 | 121.83 | 114.88 | 118.35 | 4.91 | 0.04 |
| Epo-R (Phospho-Tyr368) | 114 | 112 | 135 | 124 | 129.5 | 109.36 | 116.97 | 113.16 | 5.38 | 0.05 |
| ERK3 (Ab-189) | 76 | 65 | 135 | 124 | 129.5 | 72.90 | 67.88 | 70.39 | 3.55 | 0.05 |
| ERK3 (Phospho-Ser189) | 391 | 65 | 135 | 124 | 129.5 | 375.07 | 67.88 | 221.48 | 217.21 | 0.98 |
| ERK8 (Phospho-Thr175/Tyr177) | 95 | 383 | 135 | 124 | 129.5 | 91.13 | 399.99 | 245.56 | 218.40 | 0.89 |
| Estrogen Receptor-alpha (Ab-104) | 292 | 265 | 135 | 124 | 129.5 | 280.10 | 276.75 | 278.43 | 2.37 | 0.01 |
| Estrogen Receptor-alpha (Ab-106) | 1681 | 1890 | 135 | 124 | 129.5 | 1612.51 | 1973.83 | 1793.17 | 255.49 | 0.14 |
| Estrogen Receptor-alpha (Ab-118) | 101 | 84 | 135 | 124 | 129.5 | 96.89 | 87.73 | 92.31 | 6.48 | 0.07 |
| Estrogen Receptor-alpha (Ab-167) | 114 | 98 | 135 | 124 | 129.5 | 109.36 | 102.35 | 105.85 | 4.96 | 0.05 |
| Estrogen Receptor-alpha (Phospho-Ser104) | 115 | 233 | 135 | 124 | 129.5 | 110.31 | 243.33 | 176.82 | 94.06 | 0.53 |
| Estrogen Receptor-alpha (Phospho-Ser106) | 134 | 128 | 135 | 124 | 129.5 | 128.54 | 133.68 | 131.11 | 3.63 | 0.03 |
| Estrogen Receptor-alpha (Phospho-Ser118) | 237 | 284 | 135 | 124 | 129.5 | 227.34 | 296.60 | 261.97 | 48.97 | 0.19 |
| Estrogen Receptor-alpha (Phospho-Ser167) | 155 | 152 | 135 | 124 | 129.5 | 148.69 | 158.74 | 153.71 | 7.11 | 0.05 |
| ETK (Ab-40) | 157 | 129 | 135 | 124 | 129.5 | 150.60 | 134.72 | 142.66 | 11.23 | 0.08 |
| ETK (Ab-566) | 114 | 153 | 135 | 124 | 129.5 | 109.36 | 159.79 | 134.57 | 35.66 | 0.26 |
| ETK (Phospho-Tyr40) | 284 | 76 | 135 | 124 | 129.5 | 272.43 | 79.37 | 175.90 | 136.51 | 0.78 |
| Ezrin (Ab-353) | 121 | 127 | 135 | 124 | 129.5 | 116.07 | 132.63 | 124.35 | 11.71 | 0.09 |
| Ezrin (Ab-478) | 87 | 79 | 135 | 124 | 129.5 | 83.46 | 82.50 | 82.98 | 0.67 | 0.01 |
| Ezrin (Ab-566) | 630 | 546 | 135 | 124 | 129.5 | 604.33 | 570.22 | 587.28 | 24.12 | 0.04 |
| Ezrin (Phospho-Thr566) | 126 | 100 | 135 | 124 | 129.5 | 120.87 | 104.44 | 112.65 | 11.62 | 0.10 |
| Ezrin (Phospho-Tyr353) | 407 | 94 | 135 | 124 | 129.5 | 390.42 | 98.17 | 244.29 | 206.65 | 0.85 |
| Ezrin (Phospho-Tyr478) | 84 | 302 | 135 | 124 | 129.5 | 80.58 | 315.40 | 197.99 | 166.04 | 0.84 |
| FADD (Phospho-Ser194) | 592 | 550 | 135 | 124 | 129.5 | 567.88 | 574.40 | 571.14 | 4.61 | 0.01 |
| FAK (Ab-397) | 180 | 130 | 135 | 124 | 129.5 | 172.67 | 135.77 | 154.22 | 26.09 | 0.17 |
| FAK (Ab-407) | 88 | 81 | 135 | 124 | 129.5 | 84.41 | 84.59 | 84.50 | 0.13 | 0.00 |
| FAK (Ab-576) | 97 | 90 | 135 | 124 | 129.5 | 93.05 | 93.99 | 93.52 | 0.67 | 0.01 |
| FAK (Ab-861) | 111 | 94 | 135 | 124 | 129.5 | 106.48 | 98.17 | 102.32 | 5.87 | 0.06 |
| FAK (Ab-910) | 1724 | 2029 | 135 | 124 | 129.5 | 1653.76 | 2119.00 | 1886.38 | 328.97 | 0.17 |
| FAK (Ab-925) | 159 | 139 | 135 | 124 | 129.5 | 152.52 | 145.17 | 148.84 | 5.20 | 0.03 |
| FAK (Phospho-Ser910) | 384 | 363 | 135 | 124 | 129.5 | 368.36 | 379.10 | 373.73 | 7.60 | 0.02 |
| FAK (Phospho-Tyr397) | 82 | 258 | 135 | 124 | 129.5 | 78.66 | 269.44 | 174.05 | 134.90 | 0.78 |
| FAK (Phospho-Tyr407) | 87 | 233 | 135 | 124 | 129.5 | 83.46 | 243.33 | 163.40 | 113.05 | 0.69 |
| FAK (Phospho-Tyr576) | 155 | 144 | 135 | 124 | 129.5 | 148.69 | 150.39 | 149.54 | 1.20 | 0.01 |
| FAK (Phospho-Tyr861) | 99 | 152 | 135 | 124 | 129.5 | 94.97 | 158.74 | 126.85 | 45.10 | 0.36 |
| FAK (Phospho-Tyr925) | 123 | 114 | 135 | 124 | 129.5 | 117.99 | 119.06 | 118.52 | 0.75 | 0.01 |
| FAS (Ab-291) | 266 | 231 | 135 | 124 | 129.5 | 255.16 | 241.25 | 248.20 | 9.84 | 0.04 |
| FAS (Phospho-Tyr291) | 76 | 169 | 135 | 124 | 129.5 | 72.90 | 176.50 | 124.70 | 73.25 | 0.59 |
| FER (Ab-402) | 238 | 287 | 135 | 124 | 129.5 | 228.30 | 299.73 | 264.02 | 50.51 | 0.19 |
| FER (Phospho-Tyr402) | 333 | 565 | 135 | 124 | 129.5 | 319.43 | 590.06 | 454.75 | 191.36 | 0.42 |
| FGFR1 (Ab-154) | 151 | 97 | 135 | 124 | 129.5 | 144.85 | 101.30 | 123.08 | 30.79 | 0.25 |
| FGFR1 (Ab-766) | 135 | 119 | 135 | 124 | 129.5 | 129.50 | 124.28 | 126.89 | 3.69 | 0.03 |
| FGFR1 (Phospho-Tyr154) | 373 | 123 | 135 | 124 | 129.5 | 357.80 | 128.46 | 243.13 | 162.17 | 0.67 |
| FGFR1 (Phospho-Tyr654) | 313 | 291 | 135 | 124 | 129.5 | 300.25 | 303.91 | 302.08 | 2.59 | 0.01 |
| FGFR1 (Phospho-Tyr766) | 93 | 82 | 135 | 124 | 129.5 | 89.21 | 85.64 | 87.42 | 2.53 | 0.03 |
| Filamin A (Ab-2152) | 358 | 359 | 135 | 124 | 129.5 | 343.41 | 374.92 | 359.17 | 22.28 | 0.06 |
| Filamin A (Phospho-Ser2152) | 123 | 118 | 135 | 124 | 129.5 | 117.99 | 123.23 | 120.61 | 3.71 | 0.03 |
| FKHR (Ab-256) | 117 | 114 | 135 | 124 | 129.5 | 112.23 | 119.06 | 115.64 | 4.82 | 0.04 |
| FKHR (Ab-319) | 107 | 96 | 135 | 124 | 129.5 | 102.64 | 100.26 | 101.45 | 1.68 | 0.02 |
| FKHR (Phospho-Ser256) | 110 | 100 | 135 | 124 | 129.5 | 105.52 | 104.44 | 104.98 | 0.77 | 0.01 |
| FKHR (Phospho-Ser319) | 110 | 110 | 135 | 124 | 129.5 | 105.52 | 114.88 | 110.20 | 6.62 | 0.06 |
| FKHR/FOXO1A (Ab-329) | 92 | 121 | 135 | 124 | 129.5 | 88.25 | 126.37 | 107.31 | 26.95 | 0.25 |
| FKHR/FOXO1A (Phospho-Ser329) | 78 | 61 | 135 | 124 | 129.5 | 74.82 | 63.71 | 69.26 | 7.86 | 0.11 |
| FKHRL1/FOXO3A (Ab-253) | 224 | 203 | 135 | 124 | 129.5 | 214.87 | 212.00 | 213.44 | 2.03 | 0.01 |
| FKHRL1/FOXO3A (Phospho-Ser253) | 101 | 432 | 135 | 124 | 129.5 | 96.89 | 451.16 | 274.02 | 250.51 | 0.91 |
| FLT3 (Ab-599) | 96 | 81 | 135 | 124 | 129.5 | 92.09 | 84.59 | 88.34 | 5.30 | 0.06 |
| FLT3 (Phospho-Tyr599) | 99 | 95 | 135 | 124 | 129.5 | 94.97 | 99.21 | 97.09 | 3.00 | 0.03 |
| FLT3 (Phospho-Tyr842) | 100 | 85 | 135 | 124 | 129.5 | 95.93 | 88.77 | 92.35 | 5.06 | 0.05 |
| FLT3 (Phospho-Tyr969) | 123 | 98 | 135 | 124 | 129.5 | 117.99 | 102.35 | 110.17 | 11.06 | 0.10 |
| Fos (Ab-232) | 200 | 187 | 135 | 124 | 129.5 | 191.85 | 195.29 | 193.57 | 2.43 | 0.01 |
| Fos (Ab-374) | 272 | 305 | 135 | 124 | 129.5 | 260.92 | 318.53 | 289.72 | 40.74 | 0.14 |
| Fos (Phospho-Ser362) | 370 | 78 | 135 | 124 | 129.5 | 354.93 | 81.46 | 218.19 | 193.37 | 0.89 |
| Fos (Phospho-Thr232) | 93 | 85 | 135 | 124 | 129.5 | 89.21 | 88.77 | 88.99 | 0.31 | 0.00 |
| FosB (Ab-27) | 90 | 84 | 135 | 124 | 129.5 | 86.33 | 87.73 | 87.03 | 0.98 | 0.01 |
| FosB (Phospho-Ser27) | 81 | 70 | 135 | 124 | 129.5 | 77.70 | 73.10 | 75.40 | 3.25 | 0.04 |
| FOXO1/3/4-pan (Ab-24/32) | 128 | 114 | 135 | 124 | 129.5 | 122.79 | 119.06 | 120.92 | 2.64 | 0.02 |
| FOXO1/3/4-pan (Phospho-Thr24/32) | 249 | 72 | 135 | 124 | 129.5 | 238.86 | 75.19 | 157.02 | 115.73 | 0.74 |
| FOXO1A/3A (Phospho-Ser322/325) | 92 | 85 | 135 | 124 | 129.5 | 88.25 | 88.77 | 88.51 | 0.37 | 0.00 |
| FRS2 (Phospho-Tyr436) | 91 | 106 | 135 | 124 | 129.5 | 87.29 | 110.70 | 99.00 | 16.55 | 0.17 |
| Fyn (Phospho-Tyr530) | 76 | 69 | 135 | 124 | 129.5 | 72.90 | 72.06 | 72.48 | 0.60 | 0.01 |
| G3BP-1 (Ab-232) | 262 | 279 | 135 | 124 | 129.5 | 251.33 | 291.38 | 271.35 | 28.32 | 0.10 |
| G3BP-1 (Phospho-Ser232) | 178 | 178 | 135 | 124 | 129.5 | 170.75 | 185.90 | 178.32 | 10.71 | 0.06 |
| Gab1 (Ab-627) | 252 | 238 | 135 | 124 | 129.5 | 241.73 | 248.56 | 245.14 | 4.82 | 0.02 |
| Gab1 (Ab-659) | 199 | 78 | 135 | 124 | 129.5 | 190.89 | 81.46 | 136.18 | 77.38 | 0.57 |
| Gab1 (Phospho-Tyr627) | 305 | 256 | 135 | 124 | 129.5 | 292.57 | 267.35 | 279.96 | 17.83 | 0.06 |
| Gab1 (Phospho-Tyr659) | 109 | 105 | 135 | 124 | 129.5 | 104.56 | 109.66 | 107.11 | 3.60 | 0.03 |
| Gab2 (Ab-159) | 79 | 65 | 135 | 124 | 129.5 | 75.78 | 67.88 | 71.83 | 5.59 | 0.08 |
| Gab2 (Ab-623) | 107 | 90 | 135 | 124 | 129.5 | 102.64 | 93.99 | 98.32 | 6.12 | 0.06 |
| Gab2 (Phospho-Ser159) | 110 | 89 | 135 | 124 | 129.5 | 105.52 | 92.95 | 99.23 | 8.89 | 0.09 |
| Gab2 (Phospho-Tyr643) | 1024 | 977 | 135 | 124 | 129.5 | 982.28 | 1020.33 | 1001.31 | 26.91 | 0.03 |
| GABA-RB (Ab-434) | 81 | 72 | 135 | 124 | 129.5 | 77.70 | 75.19 | 76.45 | 1.77 | 0.02 |
| GABA-RB (Phospho-Ser434) | 255 | 130 | 135 | 124 | 129.5 | 244.61 | 135.77 | 190.19 | 76.97 | 0.40 |
| GAP43 (Ab-41) | 272 | 249 | 135 | 124 | 129.5 | 260.92 | 260.04 | 260.48 | 0.62 | 0.00 |
| GAP43 (Phospho-Ser41) | 404 | 130 | 135 | 124 | 129.5 | 387.54 | 135.77 | 261.65 | 178.03 | 0.68 |
| GAPDH | 189 | 198 | 135 | 124 | 129.5 | 181.30 | 206.78 | 194.04 | 18.02 | 0.09 |
| GATA1 (Ab-142) | 196 | 147 | 135 | 124 | 129.5 | 188.01 | 153.52 | 170.77 | 24.39 | 0.14 |
| GATA1 (Ab-310) | 189 | 172 | 135 | 124 | 129.5 | 181.30 | 179.63 | 180.46 | 1.18 | 0.01 |
| GATA1 (Phospho-Ser142) | 127 | 297 | 135 | 124 | 129.5 | 121.83 | 310.17 | 216.00 | 133.18 | 0.62 |
| GATA1 (Phospho-Ser310) | 285 | 251 | 135 | 124 | 129.5 | 273.39 | 262.13 | 267.76 | 7.96 | 0.03 |
| GluR1 (Ab-849) | 2564 | 2481 | 135 | 124 | 129.5 | 2459.54 | 2591.04 | 2525.29 | 92.99 | 0.04 |
| GluR1 (Ab-863) | 82 | 68 | 135 | 124 | 129.5 | 78.66 | 71.02 | 74.84 | 5.40 | 0.07 |
| GluR1 (Phospho-Ser849) | 151 | 141 | 135 | 124 | 129.5 | 144.85 | 147.25 | 146.05 | 1.70 | 0.01 |
| GluR1 (Phospho-Ser863) | 555 | 83 | 135 | 124 | 129.5 | 532.39 | 86.68 | 309.54 | 315.16 | 1.02 |
| GluR2 (Ab-880) | 1743 | 1405 | 135 | 124 | 129.5 | 1671.99 | 1467.32 | 1569.65 | 144.72 | 0.09 |
| GluR2 (Phospho-Ser880) | 111 | 96 | 135 | 124 | 129.5 | 106.48 | 100.26 | 103.37 | 4.40 | 0.04 |
| GRB10/Growth factor receptor-bound protein 10 (Ab-67) | 88 | 83 | 135 | 124 | 129.5 | 84.41 | 86.68 | 85.55 | 1.60 | 0.02 |
| GRB10/Growth factor receptor-bound protein 10 (Phospho-Tyr67) | 83 | 69 | 135 | 124 | 129.5 | 79.62 | 72.06 | 75.84 | 5.34 | 0.07 |
| GRF-1 (Phospho-Tyr1105) | 90 | 77 | 135 | 124 | 129.5 | 86.33 | 80.42 | 83.37 | 4.18 | 0.05 |
| GRK1 (Ab-21) | 92 | 86 | 135 | 124 | 129.5 | 88.25 | 89.81 | 89.03 | 1.10 | 0.01 |
| GRK1 (Phospho-Ser21) | 106 | 91 | 135 | 124 | 129.5 | 101.68 | 95.04 | 98.36 | 4.70 | 0.05 |
| GRK2 (Ab-29) | 532 | 476 | 135 | 124 | 129.5 | 510.33 | 497.11 | 503.72 | 9.34 | 0.02 |
| GRK2 (Phospho-Ser29) | 185 | 248 | 135 | 124 | 129.5 | 177.46 | 259.00 | 218.23 | 57.66 | 0.26 |
| GRK2 (Phospho-Ser685) | 75 | 81 | 135 | 124 | 129.5 | 71.94 | 84.59 | 78.27 | 8.94 | 0.11 |
| GSK3 alpha (Ab-21) | 206 | 185 | 135 | 124 | 129.5 | 197.61 | 193.21 | 195.41 | 3.11 | 0.02 |
| GSK3 alpha (Phospho-Ser21) | 162 | 123 | 135 | 124 | 129.5 | 155.40 | 128.46 | 141.93 | 19.05 | 0.13 |
| GSK3 alpha/beta (Ab-216/279) | 84 | 73 | 135 | 124 | 129.5 | 80.58 | 76.24 | 78.41 | 3.07 | 0.04 |
| GSK3 alpha/beta (Phospho-Tyr216/279) | 107 | 113 | 135 | 124 | 129.5 | 102.64 | 118.01 | 110.33 | 10.87 | 0.10 |
| GSK3 beta (Ab-9) | 112 | 107 | 135 | 124 | 129.5 | 107.44 | 111.75 | 109.59 | 3.05 | 0.03 |
| GSK3 beta (Phospho-Ser9) | 144 | 483 | 135 | 124 | 129.5 | 138.13 | 504.42 | 321.28 | 259.01 | 0.81 |
| GTPase activating protein (Ab-387) | 83 | 79 | 135 | 124 | 129.5 | 79.62 | 82.50 | 81.06 | 2.04 | 0.03 |
| GTPase activating protein (Phospho-Ser387) | 86 | 65 | 135 | 124 | 129.5 | 82.50 | 67.88 | 75.19 | 10.33 | 0.14 |
| HCK (Phospho-Tyr410) | 270 | 248 | 135 | 124 | 129.5 | 259.00 | 259.00 | 259.00 | 0.00 | 0.00 |
| HDAC1 (Ab-421) | 970 | 5095 | 135 | 124 | 129.5 | 930.48 | 5320.99 | 3125.73 | 3104.56 | 0.99 |
| HDAC1 (Phospho-Ser421) | 319 | 458 | 135 | 124 | 129.5 | 306.00 | 478.31 | 392.16 | 121.84 | 0.31 |
| HDAC2 (Ab-394) | 302 | 264 | 135 | 124 | 129.5 | 289.70 | 275.71 | 282.70 | 9.89 | 0.03 |
| HDAC2 (Phospho-Ser394) | 270 | 235 | 135 | 124 | 129.5 | 259.00 | 245.42 | 252.21 | 9.60 | 0.04 |
| HDAC3 (Ab-424) | 372 | 485 | 135 | 124 | 129.5 | 356.84 | 506.51 | 431.68 | 105.83 | 0.25 |
| HDAC3 (Phospho-Ser424) | 97 | 84 | 135 | 124 | 129.5 | 93.05 | 87.73 | 90.39 | 3.76 | 0.04 |
| HDAC4 (Ab-632) | 88 | 81 | 135 | 124 | 129.5 | 84.41 | 84.59 | 84.50 | 0.13 | 0.00 |
| HDAC4 (Phospho-Ser632) | 160 | 238 | 135 | 124 | 129.5 | 153.48 | 248.56 | 201.02 | 67.23 | 0.33 |
| HDAC5 (Ab-259) | 328 | 109 | 135 | 124 | 129.5 | 314.64 | 113.83 | 214.24 | 141.99 | 0.66 |
| HDAC5 (Ab-498) | 149 | 157 | 135 | 124 | 129.5 | 142.93 | 163.96 | 153.45 | 14.87 | 0.10 |
| HDAC5 (Phospho-Ser259) | 97 | 364 | 135 | 124 | 129.5 | 93.05 | 380.15 | 236.60 | 203.01 | 0.86 |
| HDAC5 (Phospho-Ser498) | 220 | 180 | 135 | 124 | 129.5 | 211.04 | 187.98 | 199.51 | 16.30 | 0.08 |
| HDAC6 (Ab-22) | 513 | 491 | 135 | 124 | 129.5 | 492.10 | 512.78 | 502.44 | 14.62 | 0.03 |
| HDAC6 (Phospho-Ser22) | 270 | 100 | 135 | 124 | 129.5 | 259.00 | 104.44 | 181.72 | 109.29 | 0.60 |
| HDAC8 (Ab-39) | 184 | 158 | 135 | 124 | 129.5 | 176.50 | 165.01 | 170.76 | 8.13 | 0.05 |
| HDAC8 (Phospho-Ser39) | 227 | 213 | 135 | 124 | 129.5 | 217.75 | 222.45 | 220.10 | 3.32 | 0.02 |
| HER2 (Ab-1112) | 180 | 179 | 135 | 124 | 129.5 | 172.67 | 186.94 | 179.80 | 10.09 | 0.06 |
| HER2 (Ab-1221/1222) | 436 | 436 | 135 | 124 | 129.5 | 418.24 | 455.34 | 436.79 | 26.23 | 0.06 |
| HER2 (Ab-1248) | 186 | 181 | 135 | 124 | 129.5 | 178.42 | 189.03 | 183.73 | 7.50 | 0.04 |
| HER2 (Ab-686) | 95 | 96 | 135 | 124 | 129.5 | 91.13 | 100.26 | 95.69 | 6.45 | 0.07 |
| HER2 (Ab-877) | 111 | 99 | 135 | 124 | 129.5 | 106.48 | 103.39 | 104.93 | 2.18 | 0.02 |
| HER2 (Phospho-Thr686) | 96 | 202 | 135 | 124 | 129.5 | 92.09 | 210.96 | 151.52 | 84.05 | 0.55 |
| HER2 (Phospho-Tyr1221/Tyr1222) | 127 | 115 | 135 | 124 | 129.5 | 121.83 | 120.10 | 120.96 | 1.22 | 0.01 |
| HER2 (Phospho-Tyr1248) | 156 | 146 | 135 | 124 | 129.5 | 149.64 | 152.48 | 151.06 | 2.00 | 0.01 |
| HER2 (Phospho-Tyr877) | 534 | 475 | 135 | 124 | 129.5 | 512.24 | 496.07 | 504.16 | 11.44 | 0.02 |
| HER3/ErbB3 (Ab-1222) | 97 | 95 | 135 | 124 | 129.5 | 93.05 | 99.21 | 96.13 | 4.36 | 0.05 |
| HER3/ErbB3 (Ab-1289) | 119 | 110 | 135 | 124 | 129.5 | 114.15 | 114.88 | 114.52 | 0.51 | 0.00 |
| HER3/ErbB3 (Phospho-Tyr1222) | 101 | 80 | 135 | 124 | 129.5 | 96.89 | 83.55 | 90.22 | 9.43 | 0.10 |
| HER3/ErbB3 (Phospho-Tyr1289) | 96 | 78 | 135 | 124 | 129.5 | 92.09 | 81.46 | 86.77 | 7.52 | 0.09 |
| HER4/ErbB4 (Ab-1284) | 312 | 372 | 135 | 124 | 129.5 | 299.29 | 388.50 | 343.89 | 63.08 | 0.18 |
| HER4/ErbB4 (Phospho-Tyr1284) | 342 | 327 | 135 | 124 | 129.5 | 328.07 | 341.50 | 334.79 | 9.50 | 0.03 |
| Histone H2A.X (Ab-139) | 388 | 373 | 135 | 124 | 129.5 | 372.19 | 389.54 | 380.87 | 12.27 | 0.03 |
| Histone H2A.X (Phospho-Ser139) | 351 | 313 | 135 | 124 | 129.5 | 336.70 | 326.88 | 331.79 | 6.94 | 0.02 |
| Histone H3.1 (Ab-10) | 255 | 218 | 135 | 124 | 129.5 | 244.61 | 227.67 | 236.14 | 11.98 | 0.05 |
| Histone H3.1 (Phospho-Ser10) | 124 | 103 | 135 | 124 | 129.5 | 118.95 | 107.57 | 113.26 | 8.05 | 0.07 |
| HNF4 alpha (Ab-313) | 107 | 88 | 135 | 124 | 129.5 | 102.64 | 91.90 | 97.27 | 7.59 | 0.08 |
| HNF4 alpha (Phospho-Ser313) | 130 | 481 | 135 | 124 | 129.5 | 124.70 | 502.33 | 313.52 | 267.03 | 0.85 |
| HRS (Ab-334) | 104 | 89 | 135 | 124 | 129.5 | 99.76 | 92.95 | 96.36 | 4.82 | 0.05 |
| HRS (Phospho-Tyr334) | 101 | 84 | 135 | 124 | 129.5 | 96.89 | 87.73 | 92.31 | 6.48 | 0.07 |
| HSF1 (Ab-303) | 92 | 78 | 135 | 124 | 129.5 | 88.25 | 81.46 | 84.86 | 4.80 | 0.06 |
| HSF1 (Phospho-Ser303) | 107 | 90 | 135 | 124 | 129.5 | 102.64 | 93.99 | 98.32 | 6.12 | 0.06 |
| HSL (Ab-552/563) | 253 | 249 | 135 | 124 | 129.5 | 242.69 | 260.04 | 251.37 | 12.27 | 0.05 |
| HSL (Ab-554) | 512 | 75 | 135 | 124 | 129.5 | 491.14 | 78.33 | 284.73 | 291.90 | 1.03 |
| HSL (Phospho-Ser552/563) | 99 | 206 | 135 | 124 | 129.5 | 94.97 | 215.14 | 155.05 | 84.97 | 0.55 |
| HSL (Phospho-Ser554) | 86 | 86 | 135 | 124 | 129.5 | 82.50 | 89.81 | 86.16 | 5.17 | 0.06 |
| HSP27 (Ab-15) | 165 | 156 | 135 | 124 | 129.5 | 158.28 | 162.92 | 160.60 | 3.28 | 0.02 |
| HSP27 (Ab-78) | 81 | 62 | 135 | 124 | 129.5 | 77.70 | 64.75 | 71.23 | 9.16 | 0.13 |
| HSP27 (Ab-82) | 168 | 82 | 135 | 124 | 129.5 | 161.16 | 85.64 | 123.40 | 53.40 | 0.43 |
| HSP27 (Phospho-Ser15) | 208 | 378 | 135 | 124 | 129.5 | 199.53 | 394.77 | 297.15 | 138.06 | 0.46 |
| HSP27 (Phospho-Ser78) | 358 | 78 | 135 | 124 | 129.5 | 343.41 | 81.46 | 212.44 | 185.23 | 0.87 |
| HSP27 (Phospho-Ser82) | 176 | 170 | 135 | 124 | 129.5 | 168.83 | 177.54 | 173.18 | 6.16 | 0.04 |
| HSP90 co-chaperone Cdc37 (Ab-13) | 153 | 141 | 135 | 124 | 129.5 | 146.77 | 147.25 | 147.01 | 0.34 | 0.00 |
| HSP90 co-chaperone Cdc37 (Phospho-Ser13) | 202 | 178 | 135 | 124 | 129.5 | 193.77 | 185.90 | 189.83 | 5.57 | 0.03 |
| HSP90B (Ab-226) | 92 | 135 | 135 | 124 | 129.5 | 88.25 | 140.99 | 114.62 | 37.29 | 0.33 |
| HSP90B (Ab-254) | 210 | 80 | 135 | 124 | 129.5 | 201.44 | 83.55 | 142.50 | 83.37 | 0.59 |
| HSP90B (Phospho-Ser226) | 263 | 212 | 135 | 124 | 129.5 | 252.29 | 221.40 | 236.84 | 21.84 | 0.09 |
| HSP90B (Phospho-Ser254) | 92 | 87 | 135 | 124 | 129.5 | 88.25 | 90.86 | 89.56 | 1.84 | 0.02 |
| ICAM-1 (Ab-512) | 81 | 73 | 135 | 124 | 129.5 | 77.70 | 76.24 | 76.97 | 1.03 | 0.01 |
| ICAM-1 (Phospho-Tyr512) | 292 | 103 | 135 | 124 | 129.5 | 280.10 | 107.57 | 193.84 | 122.00 | 0.63 |
| ICK (Phospho-Tyr159) | 110 | 96 | 135 | 124 | 129.5 | 105.52 | 100.26 | 102.89 | 3.72 | 0.04 |
| IGF1R (Ab-1161) | 84 | 78 | 135 | 124 | 129.5 | 80.58 | 81.46 | 81.02 | 0.62 | 0.01 |
| IGF1R (Ab-1165/1166) | 92 | 74 | 135 | 124 | 129.5 | 88.25 | 77.28 | 82.77 | 7.76 | 0.09 |
| IGF1R (Phospho-Tyr1161) | 141 | 240 | 135 | 124 | 129.5 | 135.26 | 250.65 | 192.95 | 81.59 | 0.42 |
| IGF1R (Phospho-Tyr1165/1166) | 240 | 299 | 135 | 124 | 129.5 | 230.22 | 312.26 | 271.24 | 58.01 | 0.21 |
| IGF2R (Ab-2409) | 104 | 100 | 135 | 124 | 129.5 | 99.76 | 104.44 | 102.10 | 3.30 | 0.03 |
| IGF2R (Phospho-Ser2409) | 84 | 71 | 135 | 124 | 129.5 | 80.58 | 74.15 | 77.36 | 4.55 | 0.06 |
| IGFBP-3 (Ab-183) | 127 | 121 | 135 | 124 | 129.5 | 121.83 | 126.37 | 124.10 | 3.21 | 0.03 |
| IkB-alpha (Ab-32/36) | 108 | 101 | 135 | 124 | 129.5 | 103.60 | 105.48 | 104.54 | 1.33 | 0.01 |
| IkB-alpha (Ab-42) | 227 | 158 | 135 | 124 | 129.5 | 217.75 | 165.01 | 191.38 | 37.30 | 0.19 |
| IkB-alpha (Phospho-Ser32/36) | 1517 | 1665 | 135 | 124 | 129.5 | 1455.20 | 1738.85 | 1597.02 | 200.57 | 0.13 |
| IkB-alpha (Phospho-Tyr305) | 80 | 63 | 135 | 124 | 129.5 | 76.74 | 65.79 | 71.27 | 7.74 | 0.11 |
| IkB-alpha (Phospho-Tyr42) | 112 | 109 | 135 | 124 | 129.5 | 107.44 | 113.83 | 110.64 | 4.52 | 0.04 |
| IkB-beta (Ab-19) | 88 | 80 | 135 | 124 | 129.5 | 84.41 | 83.55 | 83.98 | 0.61 | 0.01 |
| IkB-beta (Phospho-Ser23) | 97 | 86 | 135 | 124 | 129.5 | 93.05 | 89.81 | 91.43 | 2.29 | 0.03 |
| IkB-beta (Phospho-Thr19) | 78 | 325 | 135 | 124 | 129.5 | 74.82 | 339.42 | 207.12 | 187.10 | 0.90 |
| IkB-epsilon (Ab-22) | 624 | 574 | 135 | 124 | 129.5 | 598.58 | 599.46 | 599.02 | 0.62 | 0.00 |
| IkB-epsilon (Phospho-Ser22) | 127 | 122 | 135 | 124 | 129.5 | 121.83 | 127.41 | 124.62 | 3.95 | 0.03 |
| IKK-alpha (Ab-23) | 195 | 171 | 135 | 124 | 129.5 | 187.06 | 178.58 | 182.82 | 5.99 | 0.03 |
| IKK-alpha (Phospho-Thr23) | 141 | 131 | 135 | 124 | 129.5 | 135.26 | 136.81 | 136.03 | 1.10 | 0.01 |
| IKK-alpha/beta (Ab-176/177) | 2647 | 2746 | 135 | 124 | 129.5 | 2539.16 | 2867.80 | 2703.48 | 232.38 | 0.09 |
| IKK-alpha/beta (Ab-180/181) | 583 | 103 | 135 | 124 | 129.5 | 559.25 | 107.57 | 333.41 | 319.39 | 0.96 |
| IKK-alpha/beta (Phospho-Ser180/181) | 266 | 281 | 135 | 124 | 129.5 | 255.16 | 293.46 | 274.31 | 27.08 | 0.10 |
| IKK-beta (Ab-188) | 211 | 194 | 135 | 124 | 129.5 | 202.40 | 202.60 | 202.50 | 0.14 | 0.00 |
| IKK-beta (Ab-199) | 645 | 527 | 135 | 124 | 129.5 | 618.72 | 550.38 | 584.55 | 48.33 | 0.08 |
| IKK-beta (Phospho-Tyr188) | 91 | 80 | 135 | 124 | 129.5 | 87.29 | 83.55 | 85.42 | 2.65 | 0.03 |
| IKK-beta (Phospho-Tyr199) | 214 | 197 | 135 | 124 | 129.5 | 205.28 | 205.74 | 205.51 | 0.32 | 0.00 |
| IKK-gamma (Ab-31) | 2025 | 820 | 135 | 124 | 129.5 | 1942.50 | 856.37 | 1399.44 | 768.01 | 0.55 |
| IKK-gamma (Ab-85) | 195 | 157 | 135 | 124 | 129.5 | 187.06 | 163.96 | 175.51 | 16.33 | 0.09 |
| IKK-gamma (Phospho-Ser31) | 115 | 66 | 135 | 124 | 129.5 | 110.31 | 68.93 | 89.62 | 29.27 | 0.33 |
| IKK-gamma (Phospho-Ser85) | 81 | 187 | 135 | 124 | 129.5 | 77.70 | 195.29 | 136.50 | 83.15 | 0.61 |
| IL-10R-alpha (Ab-496) | 140 | 123 | 135 | 124 | 129.5 | 134.30 | 128.46 | 131.38 | 4.13 | 0.03 |
| IL-10R-alpha (Phospho-Tyr496) | 504 | 171 | 135 | 124 | 129.5 | 483.47 | 178.58 | 331.03 | 215.58 | 0.65 |
| IL-13R/CD213a1 (Ab-405) | 113 | 99 | 135 | 124 | 129.5 | 108.40 | 103.39 | 105.89 | 3.54 | 0.03 |
| IL-13R/CD213a1 (Phospho-Tyr405) | 1133 | 228 | 135 | 124 | 129.5 | 1086.84 | 238.11 | 662.48 | 600.14 | 0.91 |
| IL-2RA/CD25 (Ab-268) | 85 | 78 | 135 | 124 | 129.5 | 81.54 | 81.46 | 81.50 | 0.05 | 0.00 |
| IL-2RA/CD25 (Phospho-Ser268) | 81 | 74 | 135 | 124 | 129.5 | 77.70 | 77.28 | 77.49 | 0.30 | 0.00 |
| IL3RB (Ab-593) | 99 | 189 | 135 | 124 | 129.5 | 94.97 | 197.38 | 146.17 | 72.42 | 0.50 |
| IL3RB (Phospho-Tyr593) | 304 | 321 | 135 | 124 | 129.5 | 291.61 | 335.24 | 313.43 | 30.85 | 0.10 |
| IL-4R/CD124 (Ab-497) | 159 | 153 | 135 | 124 | 129.5 | 152.52 | 159.79 | 156.15 | 5.14 | 0.03 |
| IL-4R/CD124 (Phospho-Tyr497) | 134 | 118 | 135 | 124 | 129.5 | 128.54 | 123.23 | 125.89 | 3.75 | 0.03 |
| IL7R/CD127 (Phospho-Tyr449) | 440 | 531 | 135 | 124 | 129.5 | 422.07 | 554.55 | 488.31 | 93.68 | 0.19 |
| Integrin beta-1 (Ab-788) | 273 | 232 | 135 | 124 | 129.5 | 261.88 | 242.29 | 252.08 | 13.85 | 0.05 |
| Integrin beta-1 (Ab-789) | 265 | 265 | 135 | 124 | 129.5 | 254.20 | 276.75 | 265.48 | 15.95 | 0.06 |
| Integrin beta-1 (Phospho-Thr788) | 100 | 399 | 135 | 124 | 129.5 | 95.93 | 416.70 | 256.31 | 226.82 | 0.88 |
| Integrin beta-3 (Ab-773) | 134 | 127 | 135 | 124 | 129.5 | 128.54 | 132.63 | 130.59 | 2.89 | 0.02 |
| Integrin beta-3 (Ab-785) | 96 | 79 | 135 | 124 | 129.5 | 92.09 | 82.50 | 87.30 | 6.78 | 0.08 |
| Integrin beta-3 (Phospho-Tyr773) | 468 | 434 | 135 | 124 | 129.5 | 448.93 | 453.25 | 451.09 | 3.05 | 0.01 |
| Integrin beta-3 (Phospho-Tyr785) | 105 | 90 | 135 | 124 | 129.5 | 100.72 | 93.99 | 97.36 | 4.76 | 0.05 |
| Integrin beta-4 (Ab-1510) | 95 | 89 | 135 | 124 | 129.5 | 91.13 | 92.95 | 92.04 | 1.29 | 0.01 |
| Integrin beta-4 (Phospho-Tyr1510) | 108 | 352 | 135 | 124 | 129.5 | 103.60 | 367.61 | 235.61 | 186.69 | 0.79 |
| Interferon-alpha/beta receptor alpha chain (Ab-466) | 253 | 241 | 135 | 124 | 129.5 | 242.69 | 251.69 | 247.19 | 6.36 | 0.03 |
| Interferon-alpha/beta receptor alpha chain (Phospho-Tyr466) | 93 | 85 | 135 | 124 | 129.5 | 89.21 | 88.77 | 88.99 | 0.31 | 0.00 |
| Interferon-gamma receptor alpha chain precursor (Ab-457) | 107 | 98 | 135 | 124 | 129.5 | 102.64 | 102.35 | 102.49 | 0.21 | 0.00 |
| Interferon-gamma receptor alpha chain precursor (Phospho-Tyr457) | 115 | 97 | 135 | 124 | 129.5 | 110.31 | 101.30 | 105.81 | 6.37 | 0.06 |
| IR (Ab-1361) | 100 | 87 | 135 | 124 | 129.5 | 95.93 | 90.86 | 93.39 | 3.58 | 0.04 |
| IR (Phospho-Tyr1355) | 107 | 97 | 135 | 124 | 129.5 | 102.64 | 101.30 | 101.97 | 0.95 | 0.01 |
| IR (Phospho-Tyr1361) | 151 | 481 | 135 | 124 | 129.5 | 144.85 | 502.33 | 323.59 | 252.78 | 0.78 |
| IRS-1 (Ab-307) | 136 | 113 | 135 | 124 | 129.5 | 130.46 | 118.01 | 124.24 | 8.80 | 0.07 |
| IRS-1 (Ab-312) | 274 | 242 | 135 | 124 | 129.5 | 262.84 | 252.73 | 257.79 | 7.14 | 0.03 |
| IRS-1 (Ab-323) | 151 | 253 | 135 | 124 | 129.5 | 144.85 | 264.22 | 204.53 | 84.41 | 0.41 |
| IRS-1 (Ab-636) | 95 | 79 | 135 | 124 | 129.5 | 91.13 | 82.50 | 86.82 | 6.10 | 0.07 |
| IRS-1 (Ab-639) | 1214 | 1436 | 135 | 124 | 129.5 | 1164.54 | 1499.69 | 1332.12 | 236.99 | 0.18 |
| IRS-1 (Ab-794) | 85 | 70 | 135 | 124 | 129.5 | 81.54 | 73.10 | 77.32 | 5.96 | 0.08 |
| IRS-1 (Phospho-Ser1101) | 110 | 100 | 135 | 124 | 129.5 | 105.52 | 104.44 | 104.98 | 0.77 | 0.01 |
| IRS-1 (Phospho-Ser307) | 115 | 202 | 135 | 124 | 129.5 | 110.31 | 210.96 | 160.64 | 71.17 | 0.44 |
| IRS-1 (Phospho-Ser312) | 230 | 106 | 135 | 124 | 129.5 | 220.63 | 110.70 | 165.67 | 77.73 | 0.47 |
| IRS-1 (Phospho-Ser323) | 228 | 64 | 135 | 124 | 129.5 | 218.71 | 66.84 | 142.77 | 107.39 | 0.75 |
| IRS-1 (Phospho-Ser612) | 294 | 245 | 135 | 124 | 129.5 | 282.02 | 255.87 | 268.94 | 18.49 | 0.07 |
| IRS-1 (Phospho-Ser636) | 529 | 92 | 135 | 124 | 129.5 | 507.45 | 96.08 | 301.76 | 290.88 | 0.96 |
| IRS-1 (Phospho-Ser639) | 96 | 235 | 135 | 124 | 129.5 | 92.09 | 245.42 | 168.76 | 108.42 | 0.64 |
| IRS-1 (Phospho-Ser794) | 77 | 69 | 135 | 124 | 129.5 | 73.86 | 72.06 | 72.96 | 1.27 | 0.02 |
| JAK1 (Ab-1022) | 216 | 195 | 135 | 124 | 129.5 | 207.20 | 203.65 | 205.42 | 2.51 | 0.01 |
| JAK1 (Phospho-Tyr1022) | 99 | 96 | 135 | 124 | 129.5 | 94.97 | 100.26 | 97.61 | 3.74 | 0.04 |
| JAK2 (Ab-1007) | 340 | 327 | 135 | 124 | 129.5 | 326.15 | 341.50 | 333.83 | 10.86 | 0.03 |
| JAK2 (Ab-221) | 134 | 168 | 135 | 124 | 129.5 | 128.54 | 175.45 | 152.00 | 33.17 | 0.22 |
| JAK2 (Phospho-Tyr1007) | 93 | 86 | 135 | 124 | 129.5 | 89.21 | 89.81 | 89.51 | 0.43 | 0.00 |
| JAK2 (Phospho-Tyr221) | 102 | 96 | 135 | 124 | 129.5 | 97.84 | 100.26 | 99.05 | 1.71 | 0.02 |
| JNK1/2/3 (Ab-183/185) | 221 | 198 | 135 | 124 | 129.5 | 212.00 | 206.78 | 209.39 | 3.69 | 0.02 |
| JNK1/2/3 (Phospho-Thr183/Tyr185) | 103 | 91 | 135 | 124 | 129.5 | 98.80 | 95.04 | 96.92 | 2.66 | 0.03 |
| JunB (Ab-259) | 146 | 124 | 135 | 124 | 129.5 | 140.05 | 129.50 | 134.78 | 7.46 | 0.06 |
| JunB (Ab-79) | 141 | 164 | 135 | 124 | 129.5 | 135.26 | 171.27 | 153.26 | 25.47 | 0.17 |
| JunB (Phospho-Ser259) | 179 | 194 | 135 | 124 | 129.5 | 171.71 | 202.60 | 187.16 | 21.85 | 0.12 |
| JunB (Phospho-Ser79) | 193 | 182 | 135 | 124 | 129.5 | 185.14 | 190.07 | 187.60 | 3.49 | 0.02 |
| JunD (Ab-255) | 97 | 80 | 135 | 124 | 129.5 | 93.05 | 83.55 | 88.30 | 6.72 | 0.08 |
| JunD (Phospho-Ser255) | 583 | 143 | 135 | 124 | 129.5 | 559.25 | 149.34 | 354.30 | 289.85 | 0.82 |
| Keratin 18 (Ab-33) | 105 | 96 | 135 | 124 | 129.5 | 100.72 | 100.26 | 100.49 | 0.33 | 0.00 |
| Keratin 18 (Ab-52) | 83 | 72 | 135 | 124 | 129.5 | 79.62 | 75.19 | 77.41 | 3.13 | 0.04 |
| Keratin 18 (Phospho-Ser33) | 86 | 83 | 135 | 124 | 129.5 | 82.50 | 86.68 | 84.59 | 2.96 | 0.03 |
| Keratin 18 (Phospho-Ser52) | 92 | 72 | 135 | 124 | 129.5 | 88.25 | 75.19 | 81.72 | 9.23 | 0.11 |
| Keratin 8 (Ab-431) | 150 | 139 | 135 | 124 | 129.5 | 143.89 | 145.17 | 144.53 | 0.90 | 0.01 |
| Keratin 8 (Ab-73) | 97 | 151 | 135 | 124 | 129.5 | 93.05 | 157.70 | 125.37 | 45.71 | 0.36 |
| Keratin 8 (Phospho-Ser431) | 401 | 349 | 135 | 124 | 129.5 | 384.66 | 364.48 | 374.57 | 14.27 | 0.04 |
| Keratin 8 (Phospho-Ser73) | 86 | 79 | 135 | 124 | 129.5 | 82.50 | 82.50 | 82.50 | 0.01 | 0.00 |
| KIT (Ab-721) | 121 | 92 | 135 | 124 | 129.5 | 116.07 | 96.08 | 106.08 | 14.13 | 0.13 |
| KIT (Ab-936) | 252 | 118 | 135 | 124 | 129.5 | 241.73 | 123.23 | 182.48 | 83.79 | 0.46 |
| KIT (Phospho-Tyr703) | 85 | 436 | 135 | 124 | 129.5 | 81.54 | 455.34 | 268.44 | 264.32 | 0.98 |
| KIT (Phospho-Tyr721) | 237 | 215 | 135 | 124 | 129.5 | 227.34 | 224.54 | 225.94 | 1.99 | 0.01 |
| KIT (Phospho-Tyr936) | 219 | 236 | 135 | 124 | 129.5 | 210.08 | 246.47 | 228.27 | 25.73 | 0.11 |
| KSR (Ab-392) | 91 | 83 | 135 | 124 | 129.5 | 87.29 | 86.68 | 86.99 | 0.43 | 0.00 |
| KSR (Phospho-Ser392) | 88 | 77 | 135 | 124 | 129.5 | 84.41 | 80.42 | 82.42 | 2.83 | 0.03 |
| Kv1.3/KCNA3 (Ab-135) | 161 | 161 | 135 | 124 | 129.5 | 154.44 | 168.14 | 161.29 | 9.69 | 0.06 |
| Kv1.3/KCNA3 (Phospho-Tyr135) | 193 | 150 | 135 | 124 | 129.5 | 185.14 | 156.65 | 170.90 | 20.14 | 0.12 |
| Kv2.1/Kcnb1 (Phospho-Tyr128) | 326 | 308 | 135 | 124 | 129.5 | 312.72 | 321.66 | 317.19 | 6.32 | 0.02 |
| Lamin A (Ab-22) | 79 | 78 | 135 | 124 | 129.5 | 75.78 | 81.46 | 78.62 | 4.02 | 0.05 |
| Lamin A (Phospho-Ser22) | 376 | 320 | 135 | 124 | 129.5 | 360.68 | 334.19 | 347.44 | 18.73 | 0.05 |
| Lamin A/C (Ab-392) | 3093 | 2650 | 135 | 124 | 129.5 | 2966.99 | 2767.54 | 2867.26 | 141.03 | 0.05 |
| Lamin A/C (Phospho-Ser392) | 90 | 78 | 135 | 124 | 129.5 | 86.33 | 81.46 | 83.90 | 3.45 | 0.04 |
| LAT (Ab-161) | 109 | 96 | 135 | 124 | 129.5 | 104.56 | 100.26 | 102.41 | 3.04 | 0.03 |
| LAT (Ab-171) | 249 | 254 | 135 | 124 | 129.5 | 238.86 | 265.27 | 252.06 | 18.68 | 0.07 |
| LAT (Ab-191) | 297 | 271 | 135 | 124 | 129.5 | 284.90 | 283.02 | 283.96 | 1.33 | 0.00 |
| LAT (Phospho-Tyr171) | 78 | 68 | 135 | 124 | 129.5 | 74.82 | 71.02 | 72.92 | 2.69 | 0.04 |
| LAT (Phospho-Tyr191) | 268 | 252 | 135 | 124 | 129.5 | 257.08 | 263.18 | 260.13 | 4.31 | 0.02 |
| LCK (Ab-192) | 113 | 115 | 135 | 124 | 129.5 | 108.40 | 120.10 | 114.25 | 8.28 | 0.07 |
| LCK (Ab-393) | 2102 | 1755 | 135 | 124 | 129.5 | 2016.36 | 1832.84 | 1924.60 | 129.77 | 0.07 |
| LCK (Ab-504) | 119 | 112 | 135 | 124 | 129.5 | 114.15 | 116.97 | 115.56 | 1.99 | 0.02 |
| LCK (Ab-59) | 82 | 69 | 135 | 124 | 129.5 | 78.66 | 72.06 | 75.36 | 4.67 | 0.06 |
| LCK (Phospho-Ser59) | 171 | 121 | 135 | 124 | 129.5 | 164.03 | 126.37 | 145.20 | 26.63 | 0.18 |
| LCK (Phospho-Tyr192) | 82 | 62 | 135 | 124 | 129.5 | 78.66 | 64.75 | 71.70 | 9.84 | 0.14 |
| LCK (Phospho-Tyr393) | 350 | 209 | 135 | 124 | 129.5 | 335.74 | 218.27 | 277.01 | 83.06 | 0.30 |
| LCK (Phospho-Tyr504) | 87 | 260 | 135 | 124 | 129.5 | 83.46 | 271.53 | 177.49 | 132.99 | 0.75 |
| LIMK1 (Ab-508) | 370 | 371 | 135 | 124 | 129.5 | 354.93 | 387.46 | 371.19 | 23.00 | 0.06 |
| LIMK1 (Phospho-Thr508) | 111 | 103 | 135 | 124 | 129.5 | 106.48 | 107.57 | 107.02 | 0.77 | 0.01 |
| LIMK1/2 (Ab-508/505) | 313 | 1421 | 135 | 124 | 129.5 | 300.25 | 1484.03 | 892.14 | 837.06 | 0.94 |
| LKB1 (Ab-189) | 106 | 85 | 135 | 124 | 129.5 | 101.68 | 88.77 | 95.23 | 9.13 | 0.10 |
| LKB1 (Ab-334) | 241 | 253 | 135 | 124 | 129.5 | 231.18 | 264.22 | 247.70 | 23.36 | 0.09 |
| LKB1 (Ab-428) | 84 | 71 | 135 | 124 | 129.5 | 80.58 | 74.15 | 77.36 | 4.55 | 0.06 |
| LKB1 (Phospho-Ser428) | 161 | 128 | 135 | 124 | 129.5 | 154.44 | 133.68 | 144.06 | 14.68 | 0.10 |
| LKB1 (Phospho-Thr189) | 88 | 88 | 135 | 124 | 129.5 | 84.41 | 91.90 | 88.16 | 5.30 | 0.06 |
| LYN (Ab-507) | 3520 | 3352 | 135 | 124 | 129.5 | 3376.59 | 3500.68 | 3438.64 | 87.74 | 0.03 |
| LYN (Phospho-Tyr507) | 165 | 109 | 135 | 124 | 129.5 | 158.28 | 113.83 | 136.06 | 31.43 | 0.23 |
| MAP3K1/MEKK1 (Phospho-Thr1381) | 152 | 137 | 135 | 124 | 129.5 | 145.81 | 143.08 | 144.44 | 1.93 | 0.01 |
| MAP3K7/TAK1 (Ab-184) | 113 | 104 | 135 | 124 | 129.5 | 108.40 | 108.61 | 108.50 | 0.15 | 0.00 |
| MAP3K7/TAK1 (Ab-187) | 104 | 92 | 135 | 124 | 129.5 | 99.76 | 96.08 | 97.92 | 2.60 | 0.03 |
| MAP3K7/TAK1 (Ab-439) | 109 | 322 | 135 | 124 | 129.5 | 104.56 | 336.28 | 220.42 | 163.85 | 0.74 |
| MAP3K7/TAK1 (Phospho-Thr184) | 156 | 142 | 135 | 124 | 129.5 | 149.64 | 148.30 | 148.97 | 0.95 | 0.01 |
| MAP3K8/COT (Ab-290) | 84 | 191 | 135 | 124 | 129.5 | 80.58 | 199.47 | 140.02 | 84.07 | 0.60 |
| MAP3K8/COT (Ab-400) | 169 | 143 | 135 | 124 | 129.5 | 162.11 | 149.34 | 155.73 | 9.03 | 0.06 |
| MAP3K8/COT (Phospho-Thr290) | 79 | 74 | 135 | 124 | 129.5 | 75.78 | 77.28 | 76.53 | 1.06 | 0.01 |
| MAPKAPK2 (Ab-272) | 121 | 108 | 135 | 124 | 129.5 | 116.07 | 112.79 | 114.43 | 2.32 | 0.02 |
| MAPKAPK2 (Phospho-Ser272) | 108 | 108 | 135 | 124 | 129.5 | 103.60 | 112.79 | 108.20 | 6.50 | 0.06 |
| MAPKAPK2 (Phospho-Thr222) | 95 | 83 | 135 | 124 | 129.5 | 91.13 | 86.68 | 88.91 | 3.15 | 0.04 |
| MAPKAPK2 (Phospho-Thr334) | 255 | 214 | 135 | 124 | 129.5 | 244.61 | 223.49 | 234.05 | 14.93 | 0.06 |
| MARCKS (Ab-158) | 244 | 159 | 135 | 124 | 129.5 | 234.06 | 166.05 | 200.06 | 48.09 | 0.24 |
| MARCKS (Ab-163) | 94 | 77 | 135 | 124 | 129.5 | 90.17 | 80.42 | 85.29 | 6.90 | 0.08 |
| MARCKS (Phospho-Ser158) | 133 | 120 | 135 | 124 | 129.5 | 127.58 | 125.32 | 126.45 | 1.60 | 0.01 |
| MARCKS (Phospho-Ser163) | 370 | 116 | 135 | 124 | 129.5 | 354.93 | 121.15 | 238.04 | 165.31 | 0.69 |
| M-CSF Receptor (Ab-561) | 92 | 81 | 135 | 124 | 129.5 | 88.25 | 84.59 | 86.42 | 2.59 | 0.03 |
| M-CSF Receptor (Ab-809) | 119 | 94 | 135 | 124 | 129.5 | 114.15 | 98.17 | 106.16 | 11.30 | 0.11 |
| M-CSF Receptor (Phospho-Tyr561) | 162 | 137 | 135 | 124 | 129.5 | 155.40 | 143.08 | 149.24 | 8.71 | 0.06 |
| M-CSF Receptor (Phospho-Tyr809) | 262 | 315 | 135 | 124 | 129.5 | 251.33 | 328.97 | 290.15 | 54.90 | 0.19 |
| MDM2 (Ab-166) | 3888 | 3608 | 135 | 124 | 129.5 | 3729.60 | 3768.03 | 3748.82 | 27.18 | 0.01 |
| MDM2 (Phospho-Ser166) | 173 | 173 | 135 | 124 | 129.5 | 165.95 | 180.67 | 173.31 | 10.41 | 0.06 |
| MDM4 (Phospho-Ser367) | 145 | 82 | 135 | 124 | 129.5 | 139.09 | 85.64 | 112.36 | 37.80 | 0.34 |
| MEF2A (Ab-312) | 2073 | 2166 | 135 | 124 | 129.5 | 1988.54 | 2262.07 | 2125.31 | 193.41 | 0.09 |
| MEF2A (Ab-319) | 333 | 310 | 135 | 124 | 129.5 | 319.43 | 323.75 | 321.59 | 3.05 | 0.01 |
| MEF2A (Ab-408) | 624 | 637 | 135 | 124 | 129.5 | 598.58 | 665.25 | 631.92 | 47.15 | 0.07 |
| MEF2A (Phospho-Ser408) | 106 | 101 | 135 | 124 | 129.5 | 101.68 | 105.48 | 103.58 | 2.69 | 0.03 |
| MEF2A (Phospho-Thr312) | 177 | 278 | 135 | 124 | 129.5 | 169.79 | 290.33 | 230.06 | 85.24 | 0.37 |
| MEF2A (Phospho-Thr319) | 251 | 165 | 135 | 124 | 129.5 | 240.77 | 172.32 | 206.55 | 48.41 | 0.23 |
| MEF2C (Ab-396) | 100 | 111 | 135 | 124 | 129.5 | 95.93 | 115.92 | 105.92 | 14.14 | 0.13 |
| MEF2C (Phospho-Ser396) | 197 | 192 | 135 | 124 | 129.5 | 188.97 | 200.52 | 194.75 | 8.16 | 0.04 |
| MEF2D (Phospho-Ser444) | 111 | 95 | 135 | 124 | 129.5 | 106.48 | 99.21 | 102.85 | 5.14 | 0.05 |
| MEK1 (Ab-217) | 165 | 174 | 135 | 124 | 129.5 | 158.28 | 181.72 | 170.00 | 16.57 | 0.10 |
| MEK1 (Ab-221) | 92 | 82 | 135 | 124 | 129.5 | 88.25 | 85.64 | 86.94 | 1.85 | 0.02 |
| MEK1 (Ab-286) | 181 | 186 | 135 | 124 | 129.5 | 173.63 | 194.25 | 183.94 | 14.58 | 0.08 |
| MEK1 (Ab-291) | 358 | 398 | 135 | 124 | 129.5 | 343.41 | 415.65 | 379.53 | 51.08 | 0.13 |
| MEK1 (Ab-298) | 198 | 367 | 135 | 124 | 129.5 | 189.93 | 383.28 | 286.61 | 136.72 | 0.48 |
| MEK1 (Phospho-Ser217) | 214 | 144 | 135 | 124 | 129.5 | 205.28 | 150.39 | 177.83 | 38.82 | 0.22 |
| MEK1 (Phospho-Ser221) | 147 | 138 | 135 | 124 | 129.5 | 141.01 | 144.12 | 142.57 | 2.20 | 0.02 |
| MEK1 (Phospho-Ser298) | 220 | 219 | 135 | 124 | 129.5 | 211.04 | 228.71 | 219.88 | 12.50 | 0.06 |
| MEK1 (Phospho-Thr286) | 655 | 719 | 135 | 124 | 129.5 | 628.31 | 750.89 | 689.60 | 86.67 | 0.13 |
| MEK1 (Phospho-Thr291) | 168 | 128 | 135 | 124 | 129.5 | 161.16 | 133.68 | 147.42 | 19.43 | 0.13 |
| MEK2 (Ab-394) | 133 | 160 | 135 | 124 | 129.5 | 127.58 | 167.10 | 147.34 | 27.94 | 0.19 |
| MEK2 (Phospho-Thr394) | 403 | 286 | 135 | 124 | 129.5 | 386.58 | 298.69 | 342.63 | 62.15 | 0.18 |
| MER/SKY (Phospho-Tyr749/Tyr681) | 111 | 111 | 135 | 124 | 129.5 | 106.48 | 115.92 | 111.20 | 6.68 | 0.06 |
| Merlin (Ab-10) | 1738 | 1677 | 135 | 124 | 129.5 | 1667.19 | 1751.38 | 1709.29 | 59.53 | 0.03 |
| Merlin (Ab-518) | 89 | 76 | 135 | 124 | 129.5 | 85.37 | 79.37 | 82.37 | 4.24 | 0.05 |
| Merlin (Phospho-Ser10) | 499 | 570 | 135 | 124 | 129.5 | 478.67 | 595.28 | 536.98 | 82.46 | 0.15 |
| Merlin (Phospho-Ser518) | 101 | 85 | 135 | 124 | 129.5 | 96.89 | 88.77 | 92.83 | 5.74 | 0.06 |
| Met (Ab-1003) | 117 | 98 | 135 | 124 | 129.5 | 112.23 | 102.35 | 107.29 | 6.99 | 0.07 |
| Met (Ab-1234) | 214 | 97 | 135 | 124 | 129.5 | 205.28 | 101.30 | 153.29 | 73.52 | 0.48 |
| Met (Ab-1349) | 124 | 119 | 135 | 124 | 129.5 | 118.95 | 124.28 | 121.61 | 3.77 | 0.03 |
| Met (Phospho-Tyr1003) | 72 | 66 | 135 | 124 | 129.5 | 69.07 | 68.93 | 69.00 | 0.10 | 0.00 |
| Met (Phospho-Tyr1234) | 344 | 127 | 135 | 124 | 129.5 | 329.99 | 132.63 | 231.31 | 139.55 | 0.60 |
| Met (Phospho-Tyr1349) | 118 | 100 | 135 | 124 | 129.5 | 113.19 | 104.44 | 108.81 | 6.19 | 0.06 |
| Met (Phospho-Tyr1356) | 1237 | 2915 | 135 | 124 | 129.5 | 1186.60 | 3044.29 | 2115.45 | 1313.59 | 0.62 |
| MITF (Ab-73) | 323 | 325 | 135 | 124 | 129.5 | 309.84 | 339.42 | 324.63 | 20.91 | 0.06 |
| MITF (Phospho-Ser73) | 126 | 160 | 135 | 124 | 129.5 | 120.87 | 167.10 | 143.98 | 32.69 | 0.23 |
| MKK3/MAP2K3 (Ab-189) | 254 | 303 | 135 | 124 | 129.5 | 243.65 | 316.44 | 280.05 | 51.47 | 0.18 |
| MKK3/MAP2K3 (Ab-222) | 86 | 79 | 135 | 124 | 129.5 | 82.50 | 82.50 | 82.50 | 0.01 | 0.00 |
| MKK3/MAP2K3 (Phospho-Ser189) | 100 | 94 | 135 | 124 | 129.5 | 95.93 | 98.17 | 97.05 | 1.59 | 0.02 |
| MKK3/MAP2K3 (Phospho-Thr222) | 102 | 90 | 135 | 124 | 129.5 | 97.84 | 93.99 | 95.92 | 2.72 | 0.03 |
| MKK4/SEK1 (Ab-257) | 80 | 74 | 135 | 124 | 129.5 | 76.74 | 77.28 | 77.01 | 0.38 | 0.00 |
| MKK4/SEK1 (Ab-261) | 902 | 793 | 135 | 124 | 129.5 | 865.25 | 828.17 | 846.71 | 26.22 | 0.03 |
| MKK4/SEK1 (Ab-80) | 432 | 399 | 135 | 124 | 129.5 | 414.40 | 416.70 | 415.55 | 1.62 | 0.00 |
| MKK4/SEK1 (Phospho-Ser257) | 80 | 78 | 135 | 124 | 129.5 | 76.74 | 81.46 | 79.10 | 3.34 | 0.04 |
| MKK4/SEK1 (Phospho-Ser80) | 121 | 115 | 135 | 124 | 129.5 | 116.07 | 120.10 | 118.09 | 2.85 | 0.02 |
| MKK4/SEK1 (Phospho-Thr261) | 106 | 100 | 135 | 124 | 129.5 | 101.68 | 104.44 | 103.06 | 1.95 | 0.02 |
| MKK6/MAP2K6 (Ab-207) | 531 | 498 | 135 | 124 | 129.5 | 509.37 | 520.09 | 514.73 | 7.58 | 0.01 |
| MKK6/MAP2K6 (Phospho-Ser207) | 240 | 240 | 135 | 124 | 129.5 | 230.22 | 250.65 | 240.43 | 14.44 | 0.06 |
| MKK7/MAP2K7 (Ab-271) | 120 | 101 | 135 | 124 | 129.5 | 115.11 | 105.48 | 110.30 | 6.81 | 0.06 |
| MKK7/MAP2K7 (Phospho-Ser271) | 251 | 70 | 135 | 124 | 129.5 | 240.77 | 73.10 | 156.94 | 118.56 | 0.76 |
| MKK7/MAP2K7 (Phospho-Thr275) | 275 | 216 | 135 | 124 | 129.5 | 263.80 | 225.58 | 244.69 | 27.02 | 0.11 |
| MKP-1 (Ab-359) | 135 | 124 | 135 | 124 | 129.5 | 129.50 | 129.50 | 129.50 | 0.00 | 0.00 |
| MKP-1 (Phospho-Ser359) | 89 | 80 | 135 | 124 | 129.5 | 85.37 | 83.55 | 84.46 | 1.29 | 0.02 |
| MKP-1/2 (Ab-296/318) | 227 | 202 | 135 | 124 | 129.5 | 217.75 | 210.96 | 214.36 | 4.80 | 0.02 |
| MKP-1/2 (Phospho-Ser296/318) | 315 | 291 | 135 | 124 | 129.5 | 302.17 | 303.91 | 303.04 | 1.23 | 0.00 |
| Mnk1 (Ab-385) | 107 | 113 | 135 | 124 | 129.5 | 102.64 | 118.01 | 110.33 | 10.87 | 0.10 |
| Mnk1 (Phospho-Thr385) | 573 | 424 | 135 | 124 | 129.5 | 549.66 | 442.81 | 496.23 | 75.55 | 0.15 |
| MSK1 (Ab-360) | 1295 | 1268 | 135 | 124 | 129.5 | 1242.24 | 1324.24 | 1283.24 | 57.98 | 0.05 |
| MSK1 (Ab-376) | 201 | 155 | 135 | 124 | 129.5 | 192.81 | 161.88 | 177.34 | 21.88 | 0.12 |
| MSK1 (Ab-581) | 212 | 202 | 135 | 124 | 129.5 | 203.36 | 210.96 | 207.16 | 5.37 | 0.03 |
| MSK1 (Phospho-Ser212) | 566 | 408 | 135 | 124 | 129.5 | 542.94 | 426.10 | 484.52 | 82.62 | 0.17 |
| MSK1 (Phospho-Ser360) | 239 | 202 | 135 | 124 | 129.5 | 229.26 | 210.96 | 220.11 | 12.94 | 0.06 |
| MSK1 (Phospho-Ser376) | 104 | 187 | 135 | 124 | 129.5 | 99.76 | 195.29 | 147.53 | 67.55 | 0.46 |
| MSK1 (Phospho-Thr581) | 82 | 75 | 135 | 124 | 129.5 | 78.66 | 78.33 | 78.49 | 0.24 | 0.00 |
| MSK2 (Phospho-Thr568) | 106 | 109 | 135 | 124 | 129.5 | 101.68 | 113.83 | 107.76 | 8.59 | 0.08 |
| Mst1/Mst2 (Ab-183) | 88 | 69 | 135 | 124 | 129.5 | 84.41 | 72.06 | 78.24 | 8.74 | 0.11 |
| Mst1/Mst2 (Phospho-Thr183) | 134 | 119 | 135 | 124 | 129.5 | 128.54 | 124.28 | 126.41 | 3.01 | 0.02 |
| mTOR (Ab-2446) | 72 | 68 | 135 | 124 | 129.5 | 69.07 | 71.02 | 70.04 | 1.38 | 0.02 |
| mTOR (Ab-2448) | 85 | 66 | 135 | 124 | 129.5 | 81.54 | 68.93 | 75.23 | 8.92 | 0.12 |
| mTOR (Ab-2481) | 106 | 102 | 135 | 124 | 129.5 | 101.68 | 106.52 | 104.10 | 3.42 | 0.03 |
| mTOR (Phospho-Ser2448) | 217 | 205 | 135 | 124 | 129.5 | 208.16 | 214.09 | 211.13 | 4.20 | 0.02 |
| mTOR (Phospho-Ser2481) | 91 | 121 | 135 | 124 | 129.5 | 87.29 | 126.37 | 106.83 | 27.63 | 0.26 |
| mTOR (Phospho-Thr2446) | 78 | 236 | 135 | 124 | 129.5 | 74.82 | 246.47 | 160.64 | 121.37 | 0.76 |
| Myc (Ab-358) | 2126 | 1908 | 135 | 124 | 129.5 | 2039.39 | 1992.63 | 2016.01 | 33.06 | 0.02 |
| Myc (Ab-373) | 173 | 171 | 135 | 124 | 129.5 | 165.95 | 178.58 | 172.27 | 8.93 | 0.05 |
| Myc (Ab-58) | 95 | 79 | 135 | 124 | 129.5 | 91.13 | 82.50 | 86.82 | 6.10 | 0.07 |
| Myc (Ab-62) | 121 | 115 | 135 | 124 | 129.5 | 116.07 | 120.10 | 118.09 | 2.85 | 0.02 |
| Myc (Phospho-Ser373) | 119 | 96 | 135 | 124 | 129.5 | 114.15 | 100.26 | 107.20 | 9.82 | 0.09 |
| Myc (Phospho-Ser62) | 256 | 208 | 135 | 124 | 129.5 | 245.57 | 217.23 | 231.40 | 20.04 | 0.09 |
| Myc (Phospho-Thr358) | 184 | 266 | 135 | 124 | 129.5 | 176.50 | 277.80 | 227.15 | 71.63 | 0.32 |
| Myc (Phospho-Thr58) | 112 | 275 | 135 | 124 | 129.5 | 107.44 | 287.20 | 197.32 | 127.11 | 0.64 |
| Myosin regulatory light chain 2 (Ab-18) | 225 | 197 | 135 | 124 | 129.5 | 215.83 | 205.74 | 210.79 | 7.14 | 0.03 |
| Myosin regulatory light chain 2 (Phospho-Ser18) | 154 | 144 | 135 | 124 | 129.5 | 147.73 | 150.39 | 149.06 | 1.88 | 0.01 |
| MYPT1 (Phospho-Thr696) | 195 | 100 | 135 | 124 | 129.5 | 187.06 | 104.44 | 145.75 | 58.42 | 0.40 |
| MYPT1 (Phospho-Thr853) | 190 | 92 | 135 | 124 | 129.5 | 182.26 | 96.08 | 139.17 | 60.94 | 0.44 |
| MYT1 (Ab-83) | 293 | 257 | 135 | 124 | 129.5 | 281.06 | 268.40 | 274.73 | 8.95 | 0.03 |
| NFAT3 (Ab-168/170) | 128 | 127 | 135 | 124 | 129.5 | 122.79 | 132.63 | 127.71 | 6.96 | 0.05 |
| NFAT3 (Ab-676) | 219 | 204 | 135 | 124 | 129.5 | 210.08 | 213.05 | 211.56 | 2.10 | 0.01 |
| NFAT4 (Ab-165) | 86 | 77 | 135 | 124 | 129.5 | 82.50 | 80.42 | 81.46 | 1.47 | 0.02 |
| NFAT4 (Phospho-Ser165) | 90 | 96 | 135 | 124 | 129.5 | 86.33 | 100.26 | 93.30 | 9.85 | 0.11 |
| NFkB-p100/p52 (Ab-865) | 658 | 690 | 135 | 124 | 129.5 | 631.19 | 720.60 | 675.90 | 63.22 | 0.09 |
| NFkB-p100/p52 (Ab-869) | 346 | 146 | 135 | 124 | 129.5 | 331.90 | 152.48 | 242.19 | 126.87 | 0.52 |
| NFkB-p100/p52 (Phospho-Ser865) | 320 | 223 | 135 | 124 | 129.5 | 306.96 | 232.89 | 269.93 | 52.38 | 0.19 |
| NFkB-p100/p52 (Phospho-Ser869) | 136 | 137 | 135 | 124 | 129.5 | 130.46 | 143.08 | 136.77 | 8.92 | 0.07 |
| NFkB-p100/p52 (Phospho-Ser872) | 165 | 217 | 135 | 124 | 129.5 | 158.28 | 226.63 | 192.45 | 48.33 | 0.25 |
| NFkB-p105/p50 (Ab-337) | 137 | 132 | 135 | 124 | 129.5 | 131.42 | 137.85 | 134.64 | 4.55 | 0.03 |
| NFkB-p105/p50 (Ab-893) | 125 | 107 | 135 | 124 | 129.5 | 119.91 | 111.75 | 115.83 | 5.77 | 0.05 |
| NFkB-p105/p50 (Ab-907) | 144 | 115 | 135 | 124 | 129.5 | 138.13 | 120.10 | 129.12 | 12.75 | 0.10 |
| NFkB-p105/p50 (Ab-927) | 2037 | 1643 | 135 | 124 | 129.5 | 1954.01 | 1715.88 | 1834.94 | 168.39 | 0.09 |
| NFkB-p105/p50 (Ab-932) | 85 | 71 | 135 | 124 | 129.5 | 81.54 | 74.15 | 77.84 | 5.22 | 0.07 |
| NFkB-p105/p50 (Phospho-Ser337) | 755 | 702 | 135 | 124 | 129.5 | 724.24 | 733.14 | 728.69 | 6.29 | 0.01 |
| NFkB-p105/p50 (Phospho-Ser893) | 113 | 93 | 135 | 124 | 129.5 | 108.40 | 97.13 | 102.76 | 7.97 | 0.08 |
| NFkB-p105/p50 (Phospho-Ser907) | 157 | 143 | 135 | 124 | 129.5 | 150.60 | 149.34 | 149.97 | 0.89 | 0.01 |
| NFkB-p105/p50 (Phospho-Ser927) | 200 | 199 | 135 | 124 | 129.5 | 191.85 | 207.83 | 199.84 | 11.30 | 0.06 |
| NFkB-p105/p50 (Phospho-Ser932) | 106 | 90 | 135 | 124 | 129.5 | 101.68 | 93.99 | 97.84 | 5.44 | 0.06 |
| NFkB-p65 (Ab-254) | 117 | 101 | 135 | 124 | 129.5 | 112.23 | 105.48 | 108.86 | 4.78 | 0.04 |
| NFkB-p65 (Ab-276) | 114 | 102 | 135 | 124 | 129.5 | 109.36 | 106.52 | 107.94 | 2.00 | 0.02 |
| NFkB-p65 (Ab-281) | 153 | 127 | 135 | 124 | 129.5 | 146.77 | 132.63 | 139.70 | 9.99 | 0.07 |
| NFkB-p65 (Ab-311) | 122 | 106 | 135 | 124 | 129.5 | 117.03 | 110.70 | 113.87 | 4.47 | 0.04 |
| NFkB-p65 (Ab-435) | 124 | 111 | 135 | 124 | 129.5 | 118.95 | 115.92 | 117.44 | 2.14 | 0.02 |
| NFkB-p65 (Ab-468) | 251 | 234 | 135 | 124 | 129.5 | 240.77 | 244.38 | 242.58 | 2.55 | 0.01 |
| NFkB-p65 (Ab-505) | 219 | 200 | 135 | 124 | 129.5 | 210.08 | 208.87 | 209.47 | 0.85 | 0.00 |
| NFkB-p65 (Ab-529) | 244 | 202 | 135 | 124 | 129.5 | 234.06 | 210.96 | 222.51 | 16.33 | 0.07 |
| NFkB-p65 (Ab-536) | 133 | 130 | 135 | 124 | 129.5 | 127.58 | 135.77 | 131.67 | 5.79 | 0.04 |
| NFkB-p65 (Phospho-Ser276) | 299 | 317 | 135 | 124 | 129.5 | 286.82 | 331.06 | 308.94 | 31.28 | 0.10 |
| NFkB-p65 (Phospho-Ser311) | 123 | 113 | 135 | 124 | 129.5 | 117.99 | 118.01 | 118.00 | 0.02 | 0.00 |
| NFkB-p65 (Phospho-Ser468) | 1121 | 970 | 135 | 124 | 129.5 | 1075.33 | 1013.02 | 1044.18 | 44.06 | 0.04 |
| NFkB-p65 (Phospho-Ser529) | 436 | 125 | 135 | 124 | 129.5 | 418.24 | 130.54 | 274.39 | 203.43 | 0.74 |
| NFkB-p65 (Phospho-Ser536) | 83 | 57 | 135 | 124 | 129.5 | 79.62 | 59.53 | 69.57 | 14.21 | 0.20 |
| NFkB-p65 (Phospho-Thr254) | 114 | 99 | 135 | 124 | 129.5 | 109.36 | 103.39 | 106.37 | 4.22 | 0.04 |
| NFkB-p65 (Phospho-Thr435) | 280 | 394 | 135 | 124 | 129.5 | 268.59 | 411.48 | 340.03 | 101.03 | 0.30 |
| NFkB-p65 (Phospho-Thr505) | 160 | 148 | 135 | 124 | 129.5 | 153.48 | 154.56 | 154.02 | 0.77 | 0.00 |
| NMDAR1 (Ab-897) | 101 | 92 | 135 | 124 | 129.5 | 96.89 | 96.08 | 96.48 | 0.57 | 0.01 |
| NMDAR1 (Phospho-Ser897) | 203 | 270 | 135 | 124 | 129.5 | 194.73 | 281.98 | 238.35 | 61.69 | 0.26 |
| NMDAR2A/B (Phospho-Tyr1246/1252) | 303 | 445 | 135 | 124 | 129.5 | 290.66 | 464.74 | 377.70 | 123.09 | 0.33 |
| NMDAR2B (Ab-1472) | 998 | 1011 | 135 | 124 | 129.5 | 957.34 | 1055.84 | 1006.59 | 69.65 | 0.07 |
| NMDAR2B (Phospho-Tyr1472) | 104 | 333 | 135 | 124 | 129.5 | 99.76 | 347.77 | 223.77 | 175.37 | 0.78 |
| Opioid Receptor (Ab-375) | 73 | 64 | 135 | 124 | 129.5 | 70.03 | 66.84 | 68.43 | 2.25 | 0.03 |
| Opioid Receptor (Phospho-Ser375) | 268 | 224 | 135 | 124 | 129.5 | 257.08 | 233.94 | 245.51 | 16.37 | 0.07 |
| p130Cas (Ab-165) | 97 | 85 | 135 | 124 | 129.5 | 93.05 | 88.77 | 90.91 | 3.02 | 0.03 |
| p130Cas (Ab-410) | 84 | 66 | 135 | 124 | 129.5 | 80.58 | 68.93 | 74.75 | 8.24 | 0.11 |
| p130Cas (Phospho-Tyr165) | 85 | 85 | 135 | 124 | 129.5 | 81.54 | 88.77 | 85.15 | 5.11 | 0.06 |
| p130Cas (Phospho-Tyr410) | 76 | 73 | 135 | 124 | 129.5 | 72.90 | 76.24 | 74.57 | 2.36 | 0.03 |
| p21Cip1 (Ab-145) | 113 | 104 | 135 | 124 | 129.5 | 108.40 | 108.61 | 108.50 | 0.15 | 0.00 |
| p21Cip1 (Phospho-Thr145) | 189 | 165 | 135 | 124 | 129.5 | 181.30 | 172.32 | 176.81 | 6.35 | 0.04 |
| p27Kip1 (Ab-10) | 89 | 82 | 135 | 124 | 129.5 | 85.37 | 85.64 | 85.51 | 0.19 | 0.00 |
| p27Kip1 (Ab-187) | 121 | 113 | 135 | 124 | 129.5 | 116.07 | 118.01 | 117.04 | 1.37 | 0.01 |
| p27Kip1 (Phospho-Ser10) | 120 | 96 | 135 | 124 | 129.5 | 115.11 | 100.26 | 107.68 | 10.50 | 0.10 |
| p27Kip1 (Phospho-Thr187) | 92 | 283 | 135 | 124 | 129.5 | 88.25 | 295.55 | 191.90 | 146.58 | 0.76 |
| p300 (Ab-89) | 469 | 434 | 135 | 124 | 129.5 | 449.89 | 453.25 | 451.57 | 2.37 | 0.01 |
| p38 MAPK (Ab-180) | 76 | 64 | 135 | 124 | 129.5 | 72.90 | 66.84 | 69.87 | 4.29 | 0.06 |
| p38 MAPK (Ab-182) | 2161 | 2082 | 135 | 124 | 129.5 | 2072.96 | 2174.35 | 2123.65 | 71.69 | 0.03 |
| p38 MAPK (Ab-322) | 80 | 236 | 135 | 124 | 129.5 | 76.74 | 246.47 | 161.60 | 120.02 | 0.74 |
| p38 MAPK (Phospho-Thr180) | 102 | 95 | 135 | 124 | 129.5 | 97.84 | 99.21 | 98.53 | 0.97 | 0.01 |
| p38 MAPK (Phospho-Tyr182) | 109 | 111 | 135 | 124 | 129.5 | 104.56 | 115.92 | 110.24 | 8.04 | 0.07 |
| p38 MAPK (Phospho-Tyr322) | 84 | 80 | 135 | 124 | 129.5 | 80.58 | 83.55 | 82.06 | 2.10 | 0.03 |
| p44/42 MAPK (Ab-202) | 110 | 90 | 135 | 124 | 129.5 | 105.52 | 93.99 | 99.76 | 8.15 | 0.08 |
| p44/42 MAPK (Ab-204) | 88 | 69 | 135 | 124 | 129.5 | 84.41 | 72.06 | 78.24 | 8.74 | 0.11 |
| p44/42 MAPK (Phospho-Thr202) | 220 | 288 | 135 | 124 | 129.5 | 211.04 | 300.77 | 255.91 | 63.45 | 0.25 |
| p44/42 MAPK (Phospho-Tyr204) | 196 | 206 | 135 | 124 | 129.5 | 188.01 | 215.14 | 201.58 | 19.18 | 0.10 |
| p53 (Ab-15) | 105 | 88 | 135 | 124 | 129.5 | 100.72 | 91.90 | 96.31 | 6.24 | 0.06 |
| p53 (Ab-18) | 243 | 207 | 135 | 124 | 129.5 | 233.10 | 216.18 | 224.64 | 11.96 | 0.05 |
| p53 (Ab-20) | 464 | 414 | 135 | 124 | 129.5 | 445.10 | 432.36 | 438.73 | 9.00 | 0.02 |
| p53 (Ab-315) | 807 | 700 | 135 | 124 | 129.5 | 774.12 | 731.05 | 752.59 | 30.46 | 0.04 |
| p53 (Ab-33) | 149 | 133 | 135 | 124 | 129.5 | 142.93 | 138.90 | 140.91 | 2.85 | 0.02 |
| p53 (Ab-37) | 160 | 166 | 135 | 124 | 129.5 | 153.48 | 173.36 | 163.42 | 14.06 | 0.09 |
| p53 (Ab-376) | 234 | 236 | 135 | 124 | 129.5 | 224.47 | 246.47 | 235.47 | 15.56 | 0.07 |
| p53 (Ab-378) | 77 | 76 | 135 | 124 | 129.5 | 73.86 | 79.37 | 76.62 | 3.89 | 0.05 |
| p53 (Ab-387) | 205 | 200 | 135 | 124 | 129.5 | 196.65 | 208.87 | 202.76 | 8.64 | 0.04 |
| p53 (Ab-392) | 121 | 329 | 135 | 124 | 129.5 | 116.07 | 343.59 | 229.83 | 160.88 | 0.70 |
| p53 (Ab-46) | 96 | 80 | 135 | 124 | 129.5 | 92.09 | 83.55 | 87.82 | 6.04 | 0.07 |
| p53 (Ab-6) | 87 | 74 | 135 | 124 | 129.5 | 83.46 | 77.28 | 80.37 | 4.37 | 0.05 |
| p53 (Ab-9) | 148 | 124 | 135 | 124 | 129.5 | 141.97 | 129.50 | 135.74 | 8.82 | 0.06 |
| p53 (Phospho-Ser15) | 176 | 158 | 135 | 124 | 129.5 | 168.83 | 165.01 | 166.92 | 2.70 | 0.02 |
| p53 (Phospho-Ser20) | 117 | 96 | 135 | 124 | 129.5 | 112.23 | 100.26 | 106.25 | 8.47 | 0.08 |
| p53 (Phospho-Ser315) | 228 | 207 | 135 | 124 | 129.5 | 218.71 | 216.18 | 217.45 | 1.79 | 0.01 |
| p53 (Phospho-Ser33) | 181 | 96 | 135 | 124 | 129.5 | 173.63 | 100.26 | 136.94 | 51.88 | 0.38 |
| p53 (Phospho-Ser366) | 328 | 324 | 135 | 124 | 129.5 | 314.64 | 338.37 | 326.50 | 16.78 | 0.05 |
| p53 (Phospho-Ser37) | 199 | 238 | 135 | 124 | 129.5 | 190.89 | 248.56 | 219.72 | 40.77 | 0.19 |
| p53 (Phospho-Ser378) | 310 | 312 | 135 | 124 | 129.5 | 297.37 | 325.84 | 311.60 | 20.13 | 0.06 |
| p53 (Phospho-Ser392) | 196 | 173 | 135 | 124 | 129.5 | 188.01 | 180.67 | 184.34 | 5.19 | 0.03 |
| p53 (Phospho-Ser46) | 293 | 312 | 135 | 124 | 129.5 | 281.06 | 325.84 | 303.45 | 31.66 | 0.10 |
| p53 (Phospho-Ser6) | 95 | 86 | 135 | 124 | 129.5 | 91.13 | 89.81 | 90.47 | 0.93 | 0.01 |
| p53 (Phospho-Ser9) | 94 | 84 | 135 | 124 | 129.5 | 90.17 | 87.73 | 88.95 | 1.73 | 0.02 |
| p53 (Phospho-Thr18) | 182 | 155 | 135 | 124 | 129.5 | 174.59 | 161.88 | 168.23 | 8.99 | 0.05 |
| p53 (Phospho-Thr81) | 159 | 152 | 135 | 124 | 129.5 | 152.52 | 158.74 | 155.63 | 4.40 | 0.03 |
| p63 (Phospho-Ser455) | 83 | 160 | 135 | 124 | 129.5 | 79.62 | 167.10 | 123.36 | 61.86 | 0.50 |
| P70S6K (Ab-229) | 258 | 223 | 135 | 124 | 129.5 | 247.49 | 232.89 | 240.19 | 10.32 | 0.04 |
| P70S6K (Ab-371) | 101 | 231 | 135 | 124 | 129.5 | 96.89 | 241.25 | 169.07 | 102.08 | 0.60 |
| P70S6K (Ab-411) | 78 | 63 | 135 | 124 | 129.5 | 74.82 | 65.79 | 70.31 | 6.38 | 0.09 |
| P70S6K (Ab-418) | 75 | 74 | 135 | 124 | 129.5 | 71.94 | 77.28 | 74.61 | 3.77 | 0.05 |
| P70S6K (Ab-421) | 105 | 93 | 135 | 124 | 129.5 | 100.72 | 97.13 | 98.92 | 2.54 | 0.03 |
| P70S6K (Ab-424) | 99 | 84 | 135 | 124 | 129.5 | 94.97 | 87.73 | 91.35 | 5.12 | 0.06 |
| P70S6K (Ab-427) | 84 | 74 | 135 | 124 | 129.5 | 80.58 | 77.28 | 78.93 | 2.33 | 0.03 |
| P70S6K (Phospho-Ser371) | 215 | 127 | 135 | 124 | 129.5 | 206.24 | 132.63 | 169.44 | 52.05 | 0.31 |
| P70S6K (Phospho-Ser411) | 84 | 69 | 135 | 124 | 129.5 | 80.58 | 72.06 | 76.32 | 6.02 | 0.08 |
| P70S6K (Phospho-Ser418) | 236 | 73 | 135 | 124 | 129.5 | 226.39 | 76.24 | 151.31 | 106.17 | 0.70 |
| P70S6K (Phospho-Ser424) | 123 | 519 | 135 | 124 | 129.5 | 117.99 | 542.02 | 330.00 | 299.84 | 0.91 |
| P70S6K (Phospho-Thr229) | 136 | 139 | 135 | 124 | 129.5 | 130.46 | 145.17 | 137.81 | 10.40 | 0.08 |
| P70S6K (Phospho-Thr389) | 167 | 173 | 135 | 124 | 129.5 | 160.20 | 180.67 | 170.43 | 14.48 | 0.08 |
| P70S6K (Phospho-Thr421) | 201 | 118 | 135 | 124 | 129.5 | 192.81 | 123.23 | 158.02 | 49.20 | 0.31 |
| P70S6K-beta (Ab-423) | 180 | 164 | 135 | 124 | 129.5 | 172.67 | 171.27 | 171.97 | 0.98 | 0.01 |
| P70S6K-beta (Phospho-Ser423) | 220 | 269 | 135 | 124 | 129.5 | 211.04 | 280.93 | 245.98 | 49.42 | 0.20 |
| P73 (Ab-99) | 211 | 204 | 135 | 124 | 129.5 | 202.40 | 213.05 | 207.73 | 7.53 | 0.04 |
| P73 (Phospho-Tyr99) | 135 | 121 | 135 | 124 | 129.5 | 129.50 | 126.37 | 127.93 | 2.22 | 0.02 |
| P90RSK (Ab-359/363) | 2190 | 2175 | 135 | 124 | 129.5 | 2100.78 | 2271.47 | 2186.12 | 120.70 | 0.06 |
| P90RSK (Ab-380) | 139 | 133 | 135 | 124 | 129.5 | 133.34 | 138.90 | 136.12 | 3.93 | 0.03 |
| P90RSK (Ab-573) | 787 | 236 | 135 | 124 | 129.5 | 754.94 | 246.47 | 500.70 | 359.54 | 0.72 |
| P90RSK (Phospho-Ser380) | 83 | 230 | 135 | 124 | 129.5 | 79.62 | 240.20 | 159.91 | 113.55 | 0.71 |
| P90RSK (Phospho-Thr359/Ser363) | 282 | 277 | 135 | 124 | 129.5 | 270.51 | 289.29 | 279.90 | 13.28 | 0.05 |
| P90RSK (Phospho-Thr573) | 286 | 92 | 135 | 124 | 129.5 | 274.35 | 96.08 | 185.21 | 126.05 | 0.68 |
| P95/NBS1 (Ab-343) | 447 | 493 | 135 | 124 | 129.5 | 428.79 | 514.87 | 471.83 | 60.87 | 0.13 |
| P95/NBS1 (Phospho-Ser343) | 132 | 424 | 135 | 124 | 129.5 | 126.62 | 442.81 | 284.71 | 223.58 | 0.79 |
| PAK1 (Ab-204) | 112 | 104 | 135 | 124 | 129.5 | 107.44 | 108.61 | 108.02 | 0.83 | 0.01 |
| PAK1 (Ab-212) | 139 | 135 | 135 | 124 | 129.5 | 133.34 | 140.99 | 137.16 | 5.41 | 0.04 |
| PAK1 (Phospho-Ser204) | 274 | 251 | 135 | 124 | 129.5 | 262.84 | 262.13 | 262.49 | 0.50 | 0.00 |
| PAK1 (Phospho-Thr212) | 154 | 242 | 135 | 124 | 129.5 | 147.73 | 252.73 | 200.23 | 74.25 | 0.37 |
| PAK1/2 (Ab-199) | 141 | 99 | 135 | 124 | 129.5 | 135.26 | 103.39 | 119.32 | 22.53 | 0.19 |
| PAK1/2 (Phospho-Ser199) | 237 | 63 | 135 | 124 | 129.5 | 227.34 | 65.79 | 146.57 | 114.23 | 0.78 |
| PAK1/2/3 (Ab-141) | 116 | 125 | 135 | 124 | 129.5 | 111.27 | 130.54 | 120.91 | 13.63 | 0.11 |
| PAK1/2/3 (Ab-423/402/421) | 3304 | 2978 | 135 | 124 | 129.5 | 3169.39 | 3110.09 | 3139.74 | 41.93 | 0.01 |
| PAK1/2/3 (Phospho-Ser141) | 77 | 64 | 135 | 124 | 129.5 | 73.86 | 66.84 | 70.35 | 4.97 | 0.07 |
| PAK1/2/3 (Phospho-Thr423/402/421) | 107 | 73 | 135 | 124 | 129.5 | 102.64 | 76.24 | 89.44 | 18.67 | 0.21 |
| PAK2 (Ab-192) | 119 | 76 | 135 | 124 | 129.5 | 114.15 | 79.37 | 96.76 | 24.59 | 0.25 |
| PAK2 (Ab-197) | 207 | 212 | 135 | 124 | 129.5 | 198.57 | 221.40 | 209.98 | 16.15 | 0.08 |
| PAK2 (Phospho-Ser192) | 325 | 290 | 135 | 124 | 129.5 | 311.76 | 302.86 | 307.31 | 6.29 | 0.02 |
| PAK2 (Phospho-Ser20) | 98 | 82 | 135 | 124 | 129.5 | 94.01 | 85.64 | 89.82 | 5.92 | 0.07 |
| PAK3 (Ab-154) | 102 | 92 | 135 | 124 | 129.5 | 97.84 | 96.08 | 96.96 | 1.25 | 0.01 |
| PAK3 (Phospho-Ser154) | 317 | 76 | 135 | 124 | 129.5 | 304.09 | 79.37 | 191.73 | 158.90 | 0.83 |
| PAK4 (Ab-474) | 92 | 175 | 135 | 124 | 129.5 | 88.25 | 182.76 | 135.51 | 66.83 | 0.49 |
| Paxillin (Ab-118) | 89 | 79 | 135 | 124 | 129.5 | 85.37 | 82.50 | 83.94 | 2.03 | 0.02 |
| Paxillin (Ab-31) | 100 | 87 | 135 | 124 | 129.5 | 95.93 | 90.86 | 93.39 | 3.58 | 0.04 |
| Paxillin (Phospho-Tyr118) | 193 | 121 | 135 | 124 | 129.5 | 185.14 | 126.37 | 155.75 | 41.56 | 0.27 |
| Paxillin (Phospho-Tyr31) | 137 | 84 | 135 | 124 | 129.5 | 131.42 | 87.73 | 109.57 | 30.90 | 0.28 |
| PDGFR alpha (Ab-849) | 88 | 86 | 135 | 124 | 129.5 | 84.41 | 89.81 | 87.11 | 3.82 | 0.04 |
| PDGFR alpha (Phospho-Tyr849) | 119 | 269 | 135 | 124 | 129.5 | 114.15 | 280.93 | 197.54 | 117.93 | 0.60 |
| PDGFR beta (Ab-1009) | 101 | 97 | 135 | 124 | 129.5 | 96.89 | 101.30 | 99.09 | 3.12 | 0.03 |
| PDGFR beta (Ab-1021) | 109 | 104 | 135 | 124 | 129.5 | 104.56 | 108.61 | 106.59 | 2.87 | 0.03 |
| PDGFR beta (Ab-740) | 165 | 174 | 135 | 124 | 129.5 | 158.28 | 181.72 | 170.00 | 16.57 | 0.10 |
| PDGFR beta (Ab-751) | 423 | 333 | 135 | 124 | 129.5 | 405.77 | 347.77 | 376.77 | 41.01 | 0.11 |
| PDGFR beta (Phospho-Tyr1021) | 245 | 231 | 135 | 124 | 129.5 | 235.02 | 241.25 | 238.13 | 4.40 | 0.02 |
| PDGFR beta (Phospho-Tyr740) | 776 | 202 | 135 | 124 | 129.5 | 744.39 | 210.96 | 477.67 | 377.19 | 0.79 |
| PDGFR beta (Phospho-Tyr751) | 206 | 351 | 135 | 124 | 129.5 | 197.61 | 366.57 | 282.09 | 119.47 | 0.42 |
| PDK1 (Ab-241) | 911 | 1020 | 135 | 124 | 129.5 | 873.89 | 1065.24 | 969.56 | 135.31 | 0.14 |
| PDK1 (Phospho-Ser241) | 463 | 524 | 135 | 124 | 129.5 | 444.14 | 547.24 | 495.69 | 72.91 | 0.15 |
| PEA-15 (Ab-116) | 89 | 76 | 135 | 124 | 129.5 | 85.37 | 79.37 | 82.37 | 4.24 | 0.05 |
| PEA-15 (Phospho-Ser104) | 136 | 142 | 135 | 124 | 129.5 | 130.46 | 148.30 | 139.38 | 12.61 | 0.09 |
| PEA-15 (Phospho-Ser116) | 203 | 199 | 135 | 124 | 129.5 | 194.73 | 207.83 | 201.28 | 9.26 | 0.05 |
| PECAM-1 (Ab-713) | 2108 | 2029 | 135 | 124 | 129.5 | 2022.12 | 2119.00 | 2070.56 | 68.50 | 0.03 |
| PECAM-1 (Phospho-Tyr713) | 163 | 171 | 135 | 124 | 129.5 | 156.36 | 178.58 | 167.47 | 15.72 | 0.09 |
| PI3-kinase p85-alpha (Phospho-Tyr607) | 77 | 136 | 135 | 124 | 129.5 | 73.86 | 142.03 | 107.95 | 48.20 | 0.45 |
| PI3-kinase p85-subunit alpha/gamma (Ab-467/199) | 98 | 98 | 135 | 124 | 129.5 | 94.01 | 102.35 | 98.18 | 5.90 | 0.06 |
| PI3-kinase p85-subunit alpha/gamma (Phospho-Tyr467/Tyr199) | 404 | 108 | 135 | 124 | 129.5 | 387.54 | 112.79 | 250.17 | 194.28 | 0.78 |
| Pim-1 (Ab-309) | 107 | 119 | 135 | 124 | 129.5 | 102.64 | 124.28 | 113.46 | 15.30 | 0.13 |
| Pim-1 (Phospho-Tyr309) | 90 | 74 | 135 | 124 | 129.5 | 86.33 | 77.28 | 81.81 | 6.40 | 0.08 |
| PIP5K (Phospho-Ser307) | 90 | 80 | 135 | 124 | 129.5 | 86.33 | 83.55 | 84.94 | 1.97 | 0.02 |
| PKA CAT (Ab-197) | 242 | 217 | 135 | 124 | 129.5 | 232.14 | 226.63 | 229.38 | 3.90 | 0.02 |
| PKA CAT (Phospho-Thr197) | 123 | 106 | 135 | 124 | 129.5 | 117.99 | 110.70 | 114.35 | 5.15 | 0.05 |
| PKA-R2B (Phospho-Ser113) | 255 | 222 | 135 | 124 | 129.5 | 244.61 | 231.85 | 238.23 | 9.03 | 0.04 |
| PKC alpha (Ab-657) | 79 | 68 | 135 | 124 | 129.5 | 75.78 | 71.02 | 73.40 | 3.37 | 0.05 |
| PKC alpha (Phospho-Tyr657) | 237 | 239 | 135 | 124 | 129.5 | 227.34 | 249.60 | 238.47 | 15.74 | 0.07 |
| PKC alpha/beta II (Ab-638) | 84 | 68 | 135 | 124 | 129.5 | 80.58 | 71.02 | 75.80 | 6.76 | 0.09 |
| PKC alpha/beta II (Phospho-Thr638) | 214 | 199 | 135 | 124 | 129.5 | 205.28 | 207.83 | 206.55 | 1.80 | 0.01 |
| PKC beta/PKCB (Ab-661) | 110 | 91 | 135 | 124 | 129.5 | 105.52 | 95.04 | 100.28 | 7.41 | 0.07 |
| PKC beta/PKCB (Phospho-Ser661) | 163 | 140 | 135 | 124 | 129.5 | 156.36 | 146.21 | 151.28 | 7.18 | 0.05 |
| PKC delta (Ab-505) | 98 | 78 | 135 | 124 | 129.5 | 94.01 | 81.46 | 87.73 | 8.87 | 0.10 |
| PKC delta (Ab-645) | 211 | 330 | 135 | 124 | 129.5 | 202.40 | 344.64 | 273.52 | 100.57 | 0.37 |
| PKC delta (Phospho-Ser645) | 92 | 86 | 135 | 124 | 129.5 | 88.25 | 89.81 | 89.03 | 1.10 | 0.01 |
| PKC delta (Phospho-Thr505) | 107 | 107 | 135 | 124 | 129.5 | 102.64 | 111.75 | 107.19 | 6.44 | 0.06 |
| PKC delta (Phospho-Tyr313) | 95 | 89 | 135 | 124 | 129.5 | 91.13 | 92.95 | 92.04 | 1.29 | 0.01 |
| PKC delta (Phospho-Tyr52) | 148 | 127 | 135 | 124 | 129.5 | 141.97 | 132.63 | 137.30 | 6.60 | 0.05 |
| PKC delta (Phospho-Tyr64) | 105 | 85 | 135 | 124 | 129.5 | 100.72 | 88.77 | 94.75 | 8.45 | 0.09 |
| PKC epsilon (Ab-729) | 81 | 75 | 135 | 124 | 129.5 | 77.70 | 78.33 | 78.01 | 0.44 | 0.01 |
| PKC epsilon (Phospho-Ser729) | 236 | 81 | 135 | 124 | 129.5 | 226.39 | 84.59 | 155.49 | 100.26 | 0.64 |
| PKC pan activation site | 181 | 182 | 135 | 124 | 129.5 | 173.63 | 190.07 | 181.85 | 11.63 | 0.06 |
| PKC pan activation site (Phospho) | 227 | 207 | 135 | 124 | 129.5 | 217.75 | 216.18 | 216.97 | 1.11 | 0.01 |
| PKC theta (Ab-676) | 135 | 109 | 135 | 124 | 129.5 | 129.50 | 113.83 | 121.67 | 11.08 | 0.09 |
| PKC theta (Phospho-Ser676) | 115 | 103 | 135 | 124 | 129.5 | 110.31 | 107.57 | 108.94 | 1.94 | 0.02 |
| PKC theta (Phospho-Thr538) | 193 | 219 | 135 | 124 | 129.5 | 185.14 | 228.71 | 206.93 | 30.81 | 0.15 |
| PKC zeta (Ab-410) | 122 | 125 | 135 | 124 | 129.5 | 117.03 | 130.54 | 123.79 | 9.56 | 0.08 |
| PKC zeta (Ab-560) | 121 | 109 | 135 | 124 | 129.5 | 116.07 | 113.83 | 114.95 | 1.58 | 0.01 |
| PKC zeta (Phospho-Thr410) | 128 | 123 | 135 | 124 | 129.5 | 122.79 | 128.46 | 125.62 | 4.01 | 0.03 |
| PKC zeta (Phospho-Thr560) | 89 | 209 | 135 | 124 | 129.5 | 85.37 | 218.27 | 151.82 | 93.97 | 0.62 |
| PKD1/PKC mu (Ab-205) | 131 | 111 | 135 | 124 | 129.5 | 125.66 | 115.92 | 120.79 | 6.89 | 0.06 |
| PKD1/PKC mu (Ab-463) | 211 | 199 | 135 | 124 | 129.5 | 202.40 | 207.83 | 205.12 | 3.83 | 0.02 |
| PKD1/PKC mu (Ab-744/748) | 185 | 181 | 135 | 124 | 129.5 | 177.46 | 189.03 | 183.25 | 8.18 | 0.04 |
| PKD1/PKC mu (Ab-910) | 87 | 84 | 135 | 124 | 129.5 | 83.46 | 87.73 | 85.59 | 3.02 | 0.04 |
| PKD1/PKC mu (Phospho-Ser205) | 102 | 90 | 135 | 124 | 129.5 | 97.84 | 93.99 | 95.92 | 2.72 | 0.03 |
| PKD1/PKC mu (Phospho-Ser910) | 157 | 123 | 135 | 124 | 129.5 | 150.60 | 128.46 | 139.53 | 15.66 | 0.11 |
| PKD1/PKC mu (Phospho-Tyr463) | 119 | 157 | 135 | 124 | 129.5 | 114.15 | 163.96 | 139.06 | 35.22 | 0.25 |
| PKD2 (Ab-876) | 76 | 63 | 135 | 124 | 129.5 | 72.90 | 65.79 | 69.35 | 5.03 | 0.07 |
| PKD2 (Phospho-Ser876) | 227 | 196 | 135 | 124 | 129.5 | 217.75 | 204.69 | 211.22 | 9.23 | 0.04 |
| PKR (Ab-446) | 82 | 70 | 135 | 124 | 129.5 | 78.66 | 73.10 | 75.88 | 3.93 | 0.05 |
| PKR (Ab-451) | 114 | 94 | 135 | 124 | 129.5 | 109.36 | 98.17 | 103.76 | 7.91 | 0.08 |
| PKR (Phospho-Thr446) | 89 | 78 | 135 | 124 | 129.5 | 85.37 | 81.46 | 83.42 | 2.77 | 0.03 |
| PKR (Phospho-Thr451) | 99 | 85 | 135 | 124 | 129.5 | 94.97 | 88.77 | 91.87 | 4.38 | 0.05 |
| PLC beta3 (Ab-1105) | 185 | 68 | 135 | 124 | 129.5 | 177.46 | 71.02 | 124.24 | 75.27 | 0.61 |
| PLC beta3 (Ab-537) | 91 | 74 | 135 | 124 | 129.5 | 87.29 | 77.28 | 82.29 | 7.08 | 0.09 |
| PLC beta3 (Phospho-Ser1105) | 84 | 80 | 135 | 124 | 129.5 | 80.58 | 83.55 | 82.06 | 2.10 | 0.03 |
| PLC beta3 (Phospho-Ser537) | 99 | 95 | 135 | 124 | 129.5 | 94.97 | 99.21 | 97.09 | 3.00 | 0.03 |
| PLCG1 (Ab-771) | 237 | 238 | 135 | 124 | 129.5 | 227.34 | 248.56 | 237.95 | 15.00 | 0.06 |
| PLCG1 (Ab-783) | 122 | 117 | 135 | 124 | 129.5 | 117.03 | 122.19 | 119.61 | 3.65 | 0.03 |
| PLCG1 (Phospho-Tyr1253) | 91 | 79 | 135 | 124 | 129.5 | 87.29 | 82.50 | 84.90 | 3.39 | 0.04 |
| PLCG1 (Phospho-Tyr771) | 111 | 85 | 135 | 124 | 129.5 | 106.48 | 88.77 | 97.62 | 12.52 | 0.13 |
| PLCG1 (Phospho-Tyr783) | 175 | 166 | 135 | 124 | 129.5 | 167.87 | 173.36 | 170.62 | 3.88 | 0.02 |
| PLCG2 (Ab-1217) | 150 | 137 | 135 | 124 | 129.5 | 143.89 | 143.08 | 143.48 | 0.57 | 0.00 |
| PLCG2 (Ab-753) | 305 | 209 | 135 | 124 | 129.5 | 292.57 | 218.27 | 255.42 | 52.54 | 0.21 |
| PLCG2 (Phospho-Tyr1217) | 150 | 139 | 135 | 124 | 129.5 | 143.89 | 145.17 | 144.53 | 0.90 | 0.01 |
| PLCG2 (Phospho-Tyr753) | 81 | 73 | 135 | 124 | 129.5 | 77.70 | 76.24 | 76.97 | 1.03 | 0.01 |
| PLD1 (Ab-561) | 126 | 120 | 135 | 124 | 129.5 | 120.87 | 125.32 | 123.09 | 3.15 | 0.03 |
| PLD1 (Phospho-Ser561) | 280 | 316 | 135 | 124 | 129.5 | 268.59 | 330.02 | 299.30 | 43.43 | 0.15 |
| PLD2 (Phospho-Tyr169) | 126 | 140 | 135 | 124 | 129.5 | 120.87 | 146.21 | 133.54 | 17.92 | 0.13 |
| PLK1 (Ab-210) | 213 | 171 | 135 | 124 | 129.5 | 204.32 | 178.58 | 191.45 | 18.20 | 0.10 |
| PLK1 (Phospho-Thr210) | 84 | 64 | 135 | 124 | 129.5 | 80.58 | 66.84 | 73.71 | 9.71 | 0.13 |
| PP1 alpha (Ab-320) | 158 | 152 | 135 | 124 | 129.5 | 151.56 | 158.74 | 155.15 | 5.08 | 0.03 |
| PP1 alpha (Phospho-Thr320) | 108 | 88 | 135 | 124 | 129.5 | 103.60 | 91.90 | 97.75 | 8.27 | 0.08 |
| PP2A-alpha (Ab-307) | 690 | 491 | 135 | 124 | 129.5 | 661.89 | 512.78 | 587.33 | 105.44 | 0.18 |
| PP2A-alpha (Phospho-Tyr307) | 238 | 198 | 135 | 124 | 129.5 | 228.30 | 206.78 | 217.54 | 15.22 | 0.07 |
| PPAR-BP (Ab-1457) | 214 | 231 | 135 | 124 | 129.5 | 205.28 | 241.25 | 223.26 | 25.43 | 0.11 |
| PPAR-BP (Phospho-Thr1457) | 196 | 86 | 135 | 124 | 129.5 | 188.01 | 89.81 | 138.91 | 69.44 | 0.50 |
| PPAR-gamma (Ab-112) | 1696 | 1706 | 135 | 124 | 129.5 | 1626.90 | 1781.67 | 1704.29 | 109.44 | 0.06 |
| PPAR-gamma (Phospho-Ser112) | 100 | 98 | 135 | 124 | 129.5 | 95.93 | 102.35 | 99.14 | 4.54 | 0.05 |
| Progesterone Receptor (Ab-190) | 263 | 210 | 135 | 124 | 129.5 | 252.29 | 219.31 | 235.80 | 23.31 | 0.10 |
| Progesterone Receptor (Phospho-Ser190) | 105 | 93 | 135 | 124 | 129.5 | 100.72 | 97.13 | 98.92 | 2.54 | 0.03 |
| PTEN (Ab-370) | 325 | 310 | 135 | 124 | 129.5 | 311.76 | 323.75 | 317.75 | 8.48 | 0.03 |
| PTEN (Ab-380) | 601 | 733 | 135 | 124 | 129.5 | 576.51 | 765.51 | 671.01 | 133.64 | 0.20 |
| PTEN (Ab-380/382/383) | 252 | 216 | 135 | 124 | 129.5 | 241.73 | 225.58 | 233.66 | 11.42 | 0.05 |
| PTEN (Phospho-Ser370) | 175 | 349 | 135 | 124 | 129.5 | 167.87 | 364.48 | 266.18 | 139.02 | 0.52 |
| PTEN (Phospho-Ser380) | 125 | 136 | 135 | 124 | 129.5 | 119.91 | 142.03 | 130.97 | 15.64 | 0.12 |
| PTEN (Phospho-Ser380/Thr382/Thr383) | 120 | 100 | 135 | 124 | 129.5 | 115.11 | 104.44 | 109.77 | 7.55 | 0.07 |
| PTPRA (Phospho-Tyr798) | 113 | 97 | 135 | 124 | 129.5 | 108.40 | 101.30 | 104.85 | 5.02 | 0.05 |
| Pyk2 (Ab-402) | 96 | 86 | 135 | 124 | 129.5 | 92.09 | 89.81 | 90.95 | 1.61 | 0.02 |
| Pyk2 (Ab-580) | 625 | 636 | 135 | 124 | 129.5 | 599.54 | 664.21 | 631.87 | 45.73 | 0.07 |
| Pyk2 (Ab-881) | 566 | 571 | 135 | 124 | 129.5 | 542.94 | 596.33 | 569.63 | 37.75 | 0.07 |
| Pyk2 (Phospho-Tyr402) | 147 | 312 | 135 | 124 | 129.5 | 141.01 | 325.84 | 233.42 | 130.69 | 0.56 |
| Pyk2 (Phospho-Tyr579) | 164 | 166 | 135 | 124 | 129.5 | 157.32 | 173.36 | 165.34 | 11.35 | 0.07 |
| Pyk2 (Phospho-Tyr580) | 124 | 105 | 135 | 124 | 129.5 | 118.95 | 109.66 | 114.30 | 6.57 | 0.06 |
| Pyk2 (Phospho-Tyr881) | 235 | 90 | 135 | 124 | 129.5 | 225.43 | 93.99 | 159.71 | 92.94 | 0.58 |
| Rac1/cdc42 (Ab-71) | 136 | 120 | 135 | 124 | 129.5 | 130.46 | 125.32 | 127.89 | 3.63 | 0.03 |
| Rac1/cdc42 (Phospho-Ser71) | 158 | 330 | 135 | 124 | 129.5 | 151.56 | 344.64 | 248.10 | 136.52 | 0.55 |
| RAD51 (Ab-309) | 105 | 93 | 135 | 124 | 129.5 | 100.72 | 97.13 | 98.92 | 2.54 | 0.03 |
| RAD51 (Phospho-Tyr315) | 100 | 256 | 135 | 124 | 129.5 | 95.93 | 267.35 | 181.64 | 121.22 | 0.67 |
| RAD52 (Phospho-Tyr104) | 160 | 140 | 135 | 124 | 129.5 | 153.48 | 146.21 | 149.85 | 5.14 | 0.03 |
| Raf1 (Ab-259) | 253 | 444 | 135 | 124 | 129.5 | 242.69 | 463.69 | 353.19 | 156.27 | 0.44 |
| Raf1 (Ab-289) | 407 | 264 | 135 | 124 | 129.5 | 390.42 | 275.71 | 333.06 | 81.11 | 0.24 |
| Raf1 (Ab-296) | 205 | 165 | 135 | 124 | 129.5 | 196.65 | 172.32 | 184.48 | 17.20 | 0.09 |
| Raf1 (Ab-338) | 149 | 194 | 135 | 124 | 129.5 | 142.93 | 202.60 | 172.77 | 42.20 | 0.24 |
| Raf1 (Ab-341) | 154 | 122 | 135 | 124 | 129.5 | 147.73 | 127.41 | 137.57 | 14.36 | 0.10 |
| Raf1 (Ab-43) | 126 | 114 | 135 | 124 | 129.5 | 120.87 | 119.06 | 119.96 | 1.28 | 0.01 |
| Raf1 (Ab-621) | 192 | 156 | 135 | 124 | 129.5 | 184.18 | 162.92 | 173.55 | 15.03 | 0.09 |
| Raf1 (Phospho-Ser259) | 213 | 413 | 135 | 124 | 129.5 | 204.32 | 431.32 | 317.82 | 160.51 | 0.51 |
| Raf1 (Phospho-Ser289) | 92 | 80 | 135 | 124 | 129.5 | 88.25 | 83.55 | 85.90 | 3.33 | 0.04 |
| Raf1 (Phospho-Ser296) | 88 | 69 | 135 | 124 | 129.5 | 84.41 | 72.06 | 78.24 | 8.74 | 0.11 |
| Raf1 (Phospho-Ser338) | 88 | 71 | 135 | 124 | 129.5 | 84.41 | 74.15 | 79.28 | 7.26 | 0.09 |
| Raf1 (Phospho-Ser43) | 80 | 231 | 135 | 124 | 129.5 | 76.74 | 241.25 | 158.99 | 116.32 | 0.73 |
| Raf1 (Phospho-Ser621) | 81 | 208 | 135 | 124 | 129.5 | 77.70 | 217.23 | 147.46 | 98.66 | 0.67 |
| Raf1 (Phospho-Tyr341) | 144 | 121 | 135 | 124 | 129.5 | 138.13 | 126.37 | 132.25 | 8.32 | 0.06 |
| RapGEF1 (Phospho-Tyr504) | 78 | 63 | 135 | 124 | 129.5 | 74.82 | 65.79 | 70.31 | 6.38 | 0.09 |
| Ras-GRF1 (Ab-916) | 327 | 253 | 135 | 124 | 129.5 | 313.68 | 264.22 | 288.95 | 34.97 | 0.12 |
| Ras-GRF1 (Phospho-Ser916) | 89 | 92 | 135 | 124 | 129.5 | 85.37 | 96.08 | 90.73 | 7.57 | 0.08 |
| Rb (Ab-608) | 134 | 93 | 135 | 124 | 129.5 | 128.54 | 97.13 | 112.83 | 22.21 | 0.20 |
| Rb (Ab-780) | 422 | 515 | 135 | 124 | 129.5 | 404.81 | 537.84 | 471.33 | 94.07 | 0.20 |
| Rb (Ab-795) | 231 | 223 | 135 | 124 | 129.5 | 221.59 | 232.89 | 227.24 | 7.99 | 0.04 |
| Rb (Ab-807) | 553 | 516 | 135 | 124 | 129.5 | 530.47 | 538.89 | 534.68 | 5.95 | 0.01 |
| Rb (Ab-811) | 179 | 164 | 135 | 124 | 129.5 | 171.71 | 171.27 | 171.49 | 0.31 | 0.00 |
| Rb (Phospho-Ser608) | 109 | 85 | 135 | 124 | 129.5 | 104.56 | 88.77 | 96.66 | 11.16 | 0.12 |
| Rb (Phospho-Ser780) | 98 | 92 | 135 | 124 | 129.5 | 94.01 | 96.08 | 95.04 | 1.47 | 0.02 |
| Rb (Phospho-Ser795) | 155 | 206 | 135 | 124 | 129.5 | 148.69 | 215.14 | 181.91 | 46.99 | 0.26 |
| Rb (Phospho-Ser807) | 110 | 101 | 135 | 124 | 129.5 | 105.52 | 105.48 | 105.50 | 0.03 | 0.00 |
| Rb (Phospho-Ser811) | 166 | 145 | 135 | 124 | 129.5 | 159.24 | 151.43 | 155.33 | 5.52 | 0.04 |
| Rb (Phospho-Thr821) | 80 | 86 | 135 | 124 | 129.5 | 76.74 | 89.81 | 83.28 | 9.24 | 0.11 |
| Rb-like-2 (RBL2) (Ab-952) | 81 | 66 | 135 | 124 | 129.5 | 77.70 | 68.93 | 73.31 | 6.20 | 0.08 |
| Rel (Ab-503) | 137 | 140 | 135 | 124 | 129.5 | 131.42 | 146.21 | 138.81 | 10.46 | 0.08 |
| Rel (Phospho-Ser503) | 332 | 101 | 135 | 124 | 129.5 | 318.47 | 105.48 | 211.98 | 150.61 | 0.71 |
| RelB (Ab-552) | 124 | 100 | 135 | 124 | 129.5 | 118.95 | 104.44 | 111.69 | 10.26 | 0.09 |
| RelB (Phospho-Ser552) | 359 | 377 | 135 | 124 | 129.5 | 344.37 | 393.72 | 369.05 | 34.89 | 0.09 |
| Ret (Ab-905) | 103 | 88 | 135 | 124 | 129.5 | 98.80 | 91.90 | 95.35 | 4.88 | 0.05 |
| Ret (Phospho-Tyr905) | 107 | 94 | 135 | 124 | 129.5 | 102.64 | 98.17 | 100.41 | 3.16 | 0.03 |
| RGS16 (Phospho-Tyr168) | 94 | 86 | 135 | 124 | 129.5 | 90.17 | 89.81 | 89.99 | 0.25 | 0.00 |
| Rho/Rac guanine nucleotide exchange factor 2 (Ab-885) | 78 | 71 | 135 | 124 | 129.5 | 74.82 | 74.15 | 74.49 | 0.48 | 0.01 |
| Rho/Rac guanine nucleotide exchange factor 2 (Phospho-Ser885) | 170 | 63 | 135 | 124 | 129.5 | 163.07 | 65.79 | 114.43 | 68.79 | 0.60 |
| RhoA (Ab-188) | 339 | 314 | 135 | 124 | 129.5 | 325.19 | 327.93 | 326.56 | 1.94 | 0.01 |
| RSK1/2/3/4 (Ab-221/227/218/232) | 210 | 113 | 135 | 124 | 129.5 | 201.44 | 118.01 | 159.73 | 59.00 | 0.37 |
| RSK1/2/3/4 (Phospho-Ser221/227/218/232) | 76 | 63 | 135 | 124 | 129.5 | 72.90 | 65.79 | 69.35 | 5.03 | 0.07 |
| RyR2 (Ab-2808) | 227 | 80 | 135 | 124 | 129.5 | 217.75 | 83.55 | 150.65 | 94.90 | 0.63 |
| RyR2 (Phospho-Ser2808) | 77 | 67 | 135 | 124 | 129.5 | 73.86 | 69.97 | 71.92 | 2.75 | 0.04 |
| S6 Ribosomal Protein (Ab-235) | 100 | 80 | 135 | 124 | 129.5 | 95.93 | 83.55 | 89.74 | 8.75 | 0.10 |
| S6 Ribosomal Protein (Phospho-Ser235) | 119 | 81 | 135 | 124 | 129.5 | 114.15 | 84.59 | 99.37 | 20.90 | 0.21 |
| SAPK/JNK (Ab-183) | 89 | 76 | 135 | 124 | 129.5 | 85.37 | 79.37 | 82.37 | 4.24 | 0.05 |
| SAPK/JNK (Ab-185) | 133 | 102 | 135 | 124 | 129.5 | 127.58 | 106.52 | 117.05 | 14.89 | 0.13 |
| SAPK/JNK (Phospho-Thr183) | 199 | 177 | 135 | 124 | 129.5 | 190.89 | 184.85 | 187.87 | 4.27 | 0.02 |
| SAPK/JNK (Phospho-Tyr185) | 145 | 88 | 135 | 124 | 129.5 | 139.09 | 91.90 | 115.50 | 33.37 | 0.29 |
| Shc (Ab-349) | 958 | 943 | 135 | 124 | 129.5 | 918.97 | 984.83 | 951.90 | 46.57 | 0.05 |
| Shc (Ab-427) | 427 | 319 | 135 | 124 | 129.5 | 409.60 | 333.15 | 371.38 | 54.06 | 0.15 |
| Shc (Phospho-Tyr349) | 166 | 103 | 135 | 124 | 129.5 | 159.24 | 107.57 | 133.40 | 36.54 | 0.27 |
| Shc (Phospho-Tyr427) | 118 | 117 | 135 | 124 | 129.5 | 113.19 | 122.19 | 117.69 | 6.36 | 0.05 |
| SHP-1 (Phospho-Tyr536) | 163 | 97 | 135 | 124 | 129.5 | 156.36 | 101.30 | 128.83 | 38.93 | 0.30 |
| SHP-2 (Ab-542) | 129 | 137 | 135 | 124 | 129.5 | 123.74 | 143.08 | 133.41 | 13.67 | 0.10 |
| SHP-2 (Ab-580) | 150 | 216 | 135 | 124 | 129.5 | 143.89 | 225.58 | 184.73 | 57.76 | 0.31 |
| SHP-2 (Phospho-Tyr542) | 334 | 320 | 135 | 124 | 129.5 | 320.39 | 334.19 | 327.29 | 9.76 | 0.03 |
| SHP-2 (Phospho-Tyr580) | 97 | 95 | 135 | 124 | 129.5 | 93.05 | 99.21 | 96.13 | 4.36 | 0.05 |
| SLP-76 (Ab-128) | 181 | 164 | 135 | 124 | 129.5 | 173.63 | 171.27 | 172.45 | 1.66 | 0.01 |
| SLP-76 (Phospho-Tyr128) | 117 | 106 | 135 | 124 | 129.5 | 112.23 | 110.70 | 111.47 | 1.08 | 0.01 |
| Smad1 (Ab-187) | 103 | 96 | 135 | 124 | 129.5 | 98.80 | 100.26 | 99.53 | 1.03 | 0.01 |
| Smad1 (Ab-465) | 87 | 73 | 135 | 124 | 129.5 | 83.46 | 76.24 | 79.85 | 5.10 | 0.06 |
| Smad1 (Phospho-Ser187) | 87 | 63 | 135 | 124 | 129.5 | 83.46 | 65.79 | 74.62 | 12.49 | 0.17 |
| Smad1 (Phospho-Ser465) | 77 | 72 | 135 | 124 | 129.5 | 73.86 | 75.19 | 74.53 | 0.94 | 0.01 |
| Smad2 (Ab-220) | 86 | 89 | 135 | 124 | 129.5 | 82.50 | 92.95 | 87.72 | 7.39 | 0.08 |
| Smad2 (Ab-245) | 75 | 69 | 135 | 124 | 129.5 | 71.94 | 72.06 | 72.00 | 0.08 | 0.00 |
| Smad2 (Ab-250) | 89 | 84 | 135 | 124 | 129.5 | 85.37 | 87.73 | 86.55 | 1.66 | 0.02 |
| Smad2 (Ab-255) | 394 | 376 | 135 | 124 | 129.5 | 377.95 | 392.68 | 385.31 | 10.42 | 0.03 |
| Smad2 (Ab-467) | 120 | 99 | 135 | 124 | 129.5 | 115.11 | 103.39 | 109.25 | 8.29 | 0.08 |
| Smad2 (Phospho-Ser250) | 102 | 77 | 135 | 124 | 129.5 | 97.84 | 80.42 | 89.13 | 12.32 | 0.14 |
| Smad2 (Phospho-Ser467) | 87 | 257 | 135 | 124 | 129.5 | 83.46 | 268.40 | 175.93 | 130.77 | 0.74 |
| Smad2 (Phospho-Thr220) | 283 | 64 | 135 | 124 | 129.5 | 271.47 | 66.84 | 169.15 | 144.70 | 0.86 |
| Smad2/3 (Ab-8) | 166 | 170 | 135 | 124 | 129.5 | 159.24 | 177.54 | 168.39 | 12.94 | 0.08 |
| Smad2/3 (Phospho-Thr8) | 270 | 384 | 135 | 124 | 129.5 | 259.00 | 401.03 | 330.02 | 100.43 | 0.30 |
| Smad3 (Ab-179) | 171 | 191 | 135 | 124 | 129.5 | 164.03 | 199.47 | 181.75 | 25.06 | 0.14 |
| Smad3 (Ab-204) | 101 | 116 | 135 | 124 | 129.5 | 96.89 | 121.15 | 109.02 | 17.15 | 0.16 |
| Smad3 (Ab-213) | 75 | 70 | 135 | 124 | 129.5 | 71.94 | 73.10 | 72.52 | 0.82 | 0.01 |
| Smad3 (Ab-425) | 96 | 82 | 135 | 124 | 129.5 | 92.09 | 85.64 | 88.86 | 4.56 | 0.05 |
| Smad3 (Phospho-Ser204) | 74 | 59 | 135 | 124 | 129.5 | 70.99 | 61.62 | 66.30 | 6.62 | 0.10 |
| Smad3 (Phospho-Ser208) | 94 | 76 | 135 | 124 | 129.5 | 90.17 | 79.37 | 84.77 | 7.64 | 0.09 |
| Smad3 (Phospho-Ser213) | 466 | 261 | 135 | 124 | 129.5 | 447.01 | 272.58 | 359.80 | 123.35 | 0.34 |
| Smad3 (Phospho-Ser425) | 84 | 84 | 135 | 124 | 129.5 | 80.58 | 87.73 | 84.15 | 5.05 | 0.06 |
| Smad3 (Phospho-Thr179) | 107 | 64 | 135 | 124 | 129.5 | 102.64 | 66.84 | 84.74 | 25.32 | 0.30 |
| SMC1 (Ab-957) | 89 | 100 | 135 | 124 | 129.5 | 85.37 | 104.44 | 94.90 | 13.48 | 0.14 |
| SMC1 (Phospho-Ser957) | 100 | 270 | 135 | 124 | 129.5 | 95.93 | 281.98 | 188.95 | 131.56 | 0.70 |
| SP1 (Ab-739) | 102 | 84 | 135 | 124 | 129.5 | 97.84 | 87.73 | 92.79 | 7.15 | 0.08 |
| SP1 (Phospho-Thr739) | 262 | 114 | 135 | 124 | 129.5 | 251.33 | 119.06 | 185.19 | 93.53 | 0.51 |
| Src (Ab-418) | 2362 | 2728 | 135 | 124 | 129.5 | 2265.77 | 2849.00 | 2557.39 | 412.41 | 0.16 |
| Src (Ab-529) | 126 | 119 | 135 | 124 | 129.5 | 120.87 | 124.28 | 122.57 | 2.41 | 0.02 |
| Src (Ab-75) | 121 | 98 | 135 | 124 | 129.5 | 116.07 | 102.35 | 109.21 | 9.70 | 0.09 |
| Src (Phospho-Ser75) | 79 | 68 | 135 | 124 | 129.5 | 75.78 | 71.02 | 73.40 | 3.37 | 0.05 |
| Src (Phospho-Tyr216) | 260 | 264 | 135 | 124 | 129.5 | 249.41 | 275.71 | 262.56 | 18.60 | 0.07 |
| Src (Phospho-Tyr418) | 123 | 120 | 135 | 124 | 129.5 | 117.99 | 125.32 | 121.66 | 5.19 | 0.04 |
| Src (Phospho-Tyr529) | 92 | 83 | 135 | 124 | 129.5 | 88.25 | 86.68 | 87.47 | 1.11 | 0.01 |
| SREBP-1 (Ab-439) | 126 | 114 | 135 | 124 | 129.5 | 120.87 | 119.06 | 119.96 | 1.28 | 0.01 |
| SREBP-1 (Phospho-Ser439) | 81 | 74 | 135 | 124 | 129.5 | 77.70 | 77.28 | 77.49 | 0.30 | 0.00 |
| SRF (Ab-77) | 223 | 92 | 135 | 124 | 129.5 | 213.91 | 96.08 | 155.00 | 83.32 | 0.54 |
| SRF (Ab-99) | 606 | 568 | 135 | 124 | 129.5 | 581.31 | 593.19 | 587.25 | 8.40 | 0.01 |
| SRF (Phospho-Ser77) | 79 | 74 | 135 | 124 | 129.5 | 75.78 | 77.28 | 76.53 | 1.06 | 0.01 |
| SRF (Phospho-Ser99) | 145 | 143 | 135 | 124 | 129.5 | 139.09 | 149.34 | 144.22 | 7.25 | 0.05 |
| STAM2 (Ab-192) | 90 | 76 | 135 | 124 | 129.5 | 86.33 | 79.37 | 82.85 | 4.92 | 0.06 |
| STAM2 (Phospho-Tyr192) | 308 | 281 | 135 | 124 | 129.5 | 295.45 | 293.46 | 294.46 | 1.41 | 0.00 |
| STAT1 (Ab-701) | 89 | 73 | 135 | 124 | 129.5 | 85.37 | 76.24 | 80.81 | 6.46 | 0.08 |
| STAT1 (Ab-727) | 174 | 156 | 135 | 124 | 129.5 | 166.91 | 162.92 | 164.92 | 2.82 | 0.02 |
| STAT1 (Phospho-Ser727) | 108 | 126 | 135 | 124 | 129.5 | 103.60 | 131.59 | 117.59 | 19.79 | 0.17 |
| STAT1 (Phospho-Tyr701) | 382 | 351 | 135 | 124 | 129.5 | 366.44 | 366.57 | 366.50 | 0.09 | 0.00 |
| STAT2 (Ab-690) | 98 | 85 | 135 | 124 | 129.5 | 94.01 | 88.77 | 91.39 | 3.70 | 0.04 |
| STAT2 (Phospho-Tyr690) | 145 | 230 | 135 | 124 | 129.5 | 139.09 | 240.20 | 189.65 | 71.49 | 0.38 |
| STAT3 (Ab-705) | 179 | 145 | 135 | 124 | 129.5 | 171.71 | 151.43 | 161.57 | 14.34 | 0.09 |
| STAT3 (Ab-727) | 92 | 86 | 135 | 124 | 129.5 | 88.25 | 89.81 | 89.03 | 1.10 | 0.01 |
| STAT3 (Phospho-Ser727) | 90 | 77 | 135 | 124 | 129.5 | 86.33 | 80.42 | 83.37 | 4.18 | 0.05 |
| STAT3 (Phospho-Tyr705) | 157 | 143 | 135 | 124 | 129.5 | 150.60 | 149.34 | 149.97 | 0.89 | 0.01 |
| STAT4 (Ab-693) | 106 | 92 | 135 | 124 | 129.5 | 101.68 | 96.08 | 98.88 | 3.96 | 0.04 |
| STAT4 (Phospho-Tyr693) | 144 | 183 | 135 | 124 | 129.5 | 138.13 | 191.12 | 164.63 | 37.47 | 0.23 |
| STAT5A (Ab-694) | 87 | 71 | 135 | 124 | 129.5 | 83.46 | 74.15 | 78.80 | 6.58 | 0.08 |
| STAT5A (Ab-780) | 77 | 60 | 135 | 124 | 129.5 | 73.86 | 62.66 | 68.26 | 7.92 | 0.12 |
| STAT5A (Phospho-Ser725) | 93 | 76 | 135 | 124 | 129.5 | 89.21 | 79.37 | 84.29 | 6.96 | 0.08 |
| STAT5A (Phospho-Ser780) | 105 | 159 | 135 | 124 | 129.5 | 100.72 | 166.05 | 133.39 | 46.20 | 0.35 |
| STAT5A (Phospho-Tyr694) | 109 | 91 | 135 | 124 | 129.5 | 104.56 | 95.04 | 99.80 | 6.73 | 0.07 |
| STAT5B (Ab-731) | 107 | 94 | 135 | 124 | 129.5 | 102.64 | 98.17 | 100.41 | 3.16 | 0.03 |
| STAT5B (Phospho-Ser731) | 210 | 182 | 135 | 124 | 129.5 | 201.44 | 190.07 | 195.76 | 8.04 | 0.04 |
| STAT6 (Ab-641) | 115 | 102 | 135 | 124 | 129.5 | 110.31 | 106.52 | 108.42 | 2.68 | 0.02 |
| STAT6 (Ab-645) | 955 | 943 | 135 | 124 | 129.5 | 916.09 | 984.83 | 950.46 | 48.60 | 0.05 |
| STAT6 (Phospho-Thr645) | 198 | 268 | 135 | 124 | 129.5 | 189.93 | 279.89 | 234.91 | 63.61 | 0.27 |
| STAT6 (Phospho-Tyr641) | 137 | 264 | 135 | 124 | 129.5 | 131.42 | 275.71 | 203.56 | 102.03 | 0.50 |
| Stathmin 1 (Ab-15) | 104 | 96 | 135 | 124 | 129.5 | 99.76 | 100.26 | 100.01 | 0.35 | 0.00 |
| Stathmin 1 (Ab-24) | 85 | 68 | 135 | 124 | 129.5 | 81.54 | 71.02 | 76.28 | 7.44 | 0.10 |
| Stathmin 1 (Ab-37) | 91 | 71 | 135 | 124 | 129.5 | 87.29 | 74.15 | 80.72 | 9.29 | 0.12 |
| Stathmin 1 (Phospho-Ser15) | 127 | 108 | 135 | 124 | 129.5 | 121.83 | 112.79 | 117.31 | 6.39 | 0.05 |
| Stathmin 1 (Phospho-Ser24) | 118 | 99 | 135 | 124 | 129.5 | 113.19 | 103.39 | 108.29 | 6.93 | 0.06 |
| Stathmin 1 (Phospho-Ser37) | 96 | 87 | 135 | 124 | 129.5 | 92.09 | 90.86 | 91.47 | 0.87 | 0.01 |
| Survivin (Ab-117) | 81 | 76 | 135 | 124 | 129.5 | 77.70 | 79.37 | 78.54 | 1.18 | 0.02 |
| Survivin (Phospho-Thr117) | 82 | 69 | 135 | 124 | 129.5 | 78.66 | 72.06 | 75.36 | 4.67 | 0.06 |
| SYK (Ab-348) | 208 | 193 | 135 | 124 | 129.5 | 199.53 | 201.56 | 200.54 | 1.44 | 0.01 |
| SYK (Ab-525) | 737 | 1591 | 135 | 124 | 129.5 | 706.97 | 1661.57 | 1184.27 | 675.00 | 0.57 |
| SYK (Phospho-Tyr323) | 108 | 100 | 135 | 124 | 129.5 | 103.60 | 104.44 | 104.02 | 0.59 | 0.01 |
| SYK (Phospho-Tyr348) | 98 | 77 | 135 | 124 | 129.5 | 94.01 | 80.42 | 87.21 | 9.61 | 0.11 |
| SYK (Phospho-Tyr525) | 211 | 71 | 135 | 124 | 129.5 | 202.40 | 74.15 | 138.28 | 90.69 | 0.66 |
| Synapsin (Ab-62) | 129 | 113 | 135 | 124 | 129.5 | 123.74 | 118.01 | 120.88 | 4.05 | 0.03 |
| Synapsin (Ab-9) | 81 | 71 | 135 | 124 | 129.5 | 77.70 | 74.15 | 75.92 | 2.51 | 0.03 |
| Synapsin (Phospho-Ser62) | 72 | 70 | 135 | 124 | 129.5 | 69.07 | 73.10 | 71.09 | 2.86 | 0.04 |
| Synapsin (Phospho-Ser9) | 296 | 191 | 135 | 124 | 129.5 | 283.94 | 199.47 | 241.71 | 59.73 | 0.25 |
| Synaptotagmin (Ab-202) | 230 | 95 | 135 | 124 | 129.5 | 220.63 | 99.21 | 159.92 | 85.85 | 0.54 |
| Synaptotagmin (Ab-309) | 107 | 97 | 135 | 124 | 129.5 | 102.64 | 101.30 | 101.97 | 0.95 | 0.01 |
| Synaptotagmin (Phospho-Ser309) | 84 | 78 | 135 | 124 | 129.5 | 80.58 | 81.46 | 81.02 | 0.62 | 0.01 |
| Synaptotagmin (Phospho-Thr202) | 86 | 80 | 135 | 124 | 129.5 | 82.50 | 83.55 | 83.02 | 0.74 | 0.01 |
| Synuclein alpha (Ab-125) | 346 | 290 | 135 | 124 | 129.5 | 331.90 | 302.86 | 317.38 | 20.53 | 0.06 |
| Synuclein alpha (Ab-133) | 132 | 90 | 135 | 124 | 129.5 | 126.62 | 93.99 | 110.31 | 23.07 | 0.21 |
| Synuclein alpha (Phospho-Tyr125) | 587 | 127 | 135 | 124 | 129.5 | 563.09 | 132.63 | 347.86 | 304.38 | 0.87 |
| Synuclein alpha (Phospho-Tyr133) | 102 | 412 | 135 | 124 | 129.5 | 97.84 | 430.27 | 264.06 | 235.06 | 0.89 |
| Synuclein alpha (Phospho-Tyr136) | 115 | 105 | 135 | 124 | 129.5 | 110.31 | 109.66 | 109.99 | 0.46 | 0.00 |
| Tau (Ab-181) | 117 | 103 | 135 | 124 | 129.5 | 112.23 | 107.57 | 109.90 | 3.30 | 0.03 |
| Tau (Ab-205) | 370 | 369 | 135 | 124 | 129.5 | 354.93 | 385.37 | 370.15 | 21.53 | 0.06 |
| Tau (Ab-212) | 892 | 839 | 135 | 124 | 129.5 | 855.66 | 876.21 | 865.94 | 14.53 | 0.02 |
| Tau (Ab-214) | 204 | 195 | 135 | 124 | 129.5 | 195.69 | 203.65 | 199.67 | 5.63 | 0.03 |
| Tau (Ab-231) | 111 | 97 | 135 | 124 | 129.5 | 106.48 | 101.30 | 103.89 | 3.66 | 0.04 |
| Tau (Ab-235) | 4557 | 4266 | 135 | 124 | 129.5 | 4371.34 | 4455.22 | 4413.28 | 59.31 | 0.01 |
| Tau (Ab-262) | 111 | 101 | 135 | 124 | 129.5 | 106.48 | 105.48 | 105.98 | 0.71 | 0.01 |
| Tau (Ab-356) | 2280 | 2371 | 135 | 124 | 129.5 | 2187.11 | 2476.17 | 2331.64 | 204.39 | 0.09 |
| Tau (Ab-396) | 370 | 428 | 135 | 124 | 129.5 | 354.93 | 446.98 | 400.95 | 65.09 | 0.16 |
| Tau (Ab-404) | 297 | 277 | 135 | 124 | 129.5 | 284.90 | 289.29 | 287.09 | 3.10 | 0.01 |
| Tau (Ab-422) | 155 | 130 | 135 | 124 | 129.5 | 148.69 | 135.77 | 142.23 | 9.14 | 0.06 |
| Tau (Phospho-Ser214) | 266 | 481 | 135 | 124 | 129.5 | 255.16 | 502.33 | 378.75 | 174.78 | 0.46 |
| Tau (Phospho-Ser235) | 447 | 377 | 135 | 124 | 129.5 | 428.79 | 393.72 | 411.26 | 24.80 | 0.06 |
| Tau (Phospho-Ser262) | 94 | 97 | 135 | 124 | 129.5 | 90.17 | 101.30 | 95.74 | 7.87 | 0.08 |
| Tau (Phospho-Ser356) | 134 | 121 | 135 | 124 | 129.5 | 128.54 | 126.37 | 127.45 | 1.54 | 0.01 |
| Tau (Phospho-Ser396) | 572 | 100 | 135 | 124 | 129.5 | 548.70 | 104.44 | 326.57 | 314.14 | 0.96 |
| Tau (Phospho-Ser404) | 134 | 122 | 135 | 124 | 129.5 | 128.54 | 127.41 | 127.98 | 0.80 | 0.01 |
| Tau (Phospho-Ser422) | 117 | 99 | 135 | 124 | 129.5 | 112.23 | 103.39 | 107.81 | 6.25 | 0.06 |
| Tau (Phospho-Thr181) | 315 | 289 | 135 | 124 | 129.5 | 302.17 | 301.82 | 301.99 | 0.25 | 0.00 |
| Tau (Phospho-Thr205) | 322 | 301 | 135 | 124 | 129.5 | 308.88 | 314.35 | 311.62 | 3.87 | 0.01 |
| Tau (Phospho-Thr212) | 239 | 132 | 135 | 124 | 129.5 | 229.26 | 137.85 | 183.56 | 64.64 | 0.35 |
| Tau (Phospho-Thr231) | 408 | 363 | 135 | 124 | 129.5 | 391.38 | 379.10 | 385.24 | 8.68 | 0.02 |
| TFII-I (Phospho-Tyr248) | 89 | 87 | 135 | 124 | 129.5 | 85.37 | 90.86 | 88.12 | 3.88 | 0.04 |
| TGFBR1 (Ab-165) | 98 | 90 | 135 | 124 | 129.5 | 94.01 | 93.99 | 94.00 | 0.01 | 0.00 |
| TGFBR2 (Ab-250) | 811 | 865 | 135 | 124 | 129.5 | 777.96 | 903.37 | 840.66 | 88.68 | 0.11 |
| TIE2 (Phospho-Tyr1108) | 129 | 112 | 135 | 124 | 129.5 | 123.74 | 116.97 | 120.36 | 4.79 | 0.04 |
| TIF-IA (Ab-649) | 85 | 114 | 135 | 124 | 129.5 | 81.54 | 119.06 | 100.30 | 26.53 | 0.26 |
| TIF-IA (Phospho-Ser649) | 132 | 150 | 135 | 124 | 129.5 | 126.62 | 156.65 | 141.64 | 21.24 | 0.15 |
| TLK1 (Ab-764) | 418 | 376 | 135 | 124 | 129.5 | 400.97 | 392.68 | 396.82 | 5.86 | 0.01 |
| TOP2A/DNA topoisomerase II (Ab-1106) | 563 | 213 | 135 | 124 | 129.5 | 540.06 | 222.45 | 381.26 | 224.59 | 0.59 |
| TOP2A/DNA topoisomerase II (Phospho-Ser1106) | 104 | 168 | 135 | 124 | 129.5 | 99.76 | 175.45 | 137.61 | 53.52 | 0.39 |
| Trk A (Ab-496) | 138 | 119 | 135 | 124 | 129.5 | 132.38 | 124.28 | 128.33 | 5.73 | 0.04 |
| Trk A (Phospho-Tyr680/681) | 95 | 87 | 135 | 124 | 129.5 | 91.13 | 90.86 | 90.99 | 0.19 | 0.00 |
| Trk A (Phospho-Tyr701) | 341 | 104 | 135 | 124 | 129.5 | 327.11 | 108.61 | 217.86 | 154.50 | 0.71 |
| Trk A (Phospho-Tyr791) | 98 | 95 | 135 | 124 | 129.5 | 94.01 | 99.21 | 96.61 | 3.68 | 0.04 |
| Trk B (Ab-515) | 280 | 313 | 135 | 124 | 129.5 | 268.59 | 326.88 | 297.74 | 41.22 | 0.14 |
| Trk B (Phospho-Tyr515) | 188 | 191 | 135 | 124 | 129.5 | 180.34 | 199.47 | 189.91 | 13.53 | 0.07 |
| Trk B (Phospho-Tyr705) | 130 | 97 | 135 | 124 | 129.5 | 124.70 | 101.30 | 113.00 | 16.55 | 0.15 |
| Tuberin/TSC2 (Ab-1462) | 88 | 74 | 135 | 124 | 129.5 | 84.41 | 77.28 | 80.85 | 5.04 | 0.06 |
| Tuberin/TSC2 (Ab-939) | 465 | 464 | 135 | 124 | 129.5 | 446.06 | 484.58 | 465.32 | 27.24 | 0.06 |
| Tuberin/TSC2 (Ab-981) | 149 | 156 | 135 | 124 | 129.5 | 142.93 | 162.92 | 152.92 | 14.13 | 0.09 |
| Tuberin/TSC2 (Phospho-Ser939) | 128 | 125 | 135 | 124 | 129.5 | 122.79 | 130.54 | 126.66 | 5.49 | 0.04 |
| Tuberin/TSC2 (Phospho-Thr1462) | 143 | 164 | 135 | 124 | 129.5 | 137.17 | 171.27 | 154.22 | 24.11 | 0.16 |
| TYK2 (Ab-1054) | 96 | 90 | 135 | 124 | 129.5 | 92.09 | 93.99 | 93.04 | 1.35 | 0.01 |
| TYK2 (Phospho-Tyr1054) | 119 | 155 | 135 | 124 | 129.5 | 114.15 | 161.88 | 138.01 | 33.75 | 0.24 |
| Tyrosine Hydroxylase (Ab-19) | 248 | 211 | 135 | 124 | 129.5 | 237.90 | 220.36 | 229.13 | 12.40 | 0.05 |
| Tyrosine Hydroxylase (Ab-31) | 103 | 219 | 135 | 124 | 129.5 | 98.80 | 228.71 | 163.76 | 91.86 | 0.56 |
| Tyrosine Hydroxylase (Ab-40) | 184 | 229 | 135 | 124 | 129.5 | 176.50 | 239.16 | 207.83 | 44.30 | 0.21 |
| Tyrosine Hydroxylase (Ab-8) | 174 | 160 | 135 | 124 | 129.5 | 166.91 | 167.10 | 167.00 | 0.13 | 0.00 |
| Tyrosine Hydroxylase (Phospho-Ser19) | 125 | 119 | 135 | 124 | 129.5 | 119.91 | 124.28 | 122.09 | 3.09 | 0.03 |
| Tyrosine Hydroxylase (Phospho-Ser31) | 102 | 95 | 135 | 124 | 129.5 | 97.84 | 99.21 | 98.53 | 0.97 | 0.01 |
| Tyrosine Hydroxylase (Phospho-Ser40) | 110 | 91 | 135 | 124 | 129.5 | 105.52 | 95.04 | 100.28 | 7.41 | 0.07 |
| Tyrosine Hydroxylase (Phospho-Ser8) | 152 | 98 | 135 | 124 | 129.5 | 145.81 | 102.35 | 124.08 | 30.73 | 0.25 |
| VASP (Ab-157) | 807 | 975 | 135 | 124 | 129.5 | 774.12 | 1018.25 | 896.18 | 172.62 | 0.19 |
| VASP (Ab-238) | 96 | 88 | 135 | 124 | 129.5 | 92.09 | 91.90 | 92.00 | 0.13 | 0.00 |
| VASP (Phospho-Ser157) | 191 | 155 | 135 | 124 | 129.5 | 183.22 | 161.88 | 172.55 | 15.09 | 0.09 |
| VASP (Phospho-Ser238) | 328 | 101 | 135 | 124 | 129.5 | 314.64 | 105.48 | 210.06 | 147.90 | 0.70 |
| VAV1 (Ab-160) | 103 | 150 | 135 | 124 | 129.5 | 98.80 | 156.65 | 127.73 | 40.91 | 0.32 |
| VAV1 (Ab-174) | 179 | 180 | 135 | 124 | 129.5 | 171.71 | 187.98 | 179.85 | 11.51 | 0.06 |
| VAV1 (Phospho-Tyr174) | 390 | 369 | 135 | 124 | 129.5 | 374.11 | 385.37 | 379.74 | 7.96 | 0.02 |
| VAV2 (Ab-142) | 353 | 87 | 135 | 124 | 129.5 | 338.62 | 90.86 | 214.74 | 175.19 | 0.82 |
| VAV2 (Phospho-Tyr142) | 409 | 176 | 135 | 124 | 129.5 | 392.34 | 183.81 | 288.07 | 147.45 | 0.51 |
| VE-Cadherin (Phospho-Tyr731) | 513 | 1712 | 135 | 124 | 129.5 | 492.10 | 1787.94 | 1140.02 | 916.29 | 0.80 |
| VEGFR1 (Ab-1333) | 157 | 122 | 135 | 124 | 129.5 | 150.60 | 127.41 | 139.01 | 16.40 | 0.12 |
| VEGFR1 (Phospho-Tyr1333) | 109 | 88 | 135 | 124 | 129.5 | 104.56 | 91.90 | 98.23 | 8.95 | 0.09 |
| VEGFR2 (Ab-1054) | 122 | 117 | 135 | 124 | 129.5 | 117.03 | 122.19 | 119.61 | 3.65 | 0.03 |
| VEGFR2 (Ab-1059) | 85 | 67 | 135 | 124 | 129.5 | 81.54 | 69.97 | 75.75 | 8.18 | 0.11 |
| VEGFR2 (Ab-1175) | 168 | 160 | 135 | 124 | 129.5 | 161.16 | 167.10 | 164.13 | 4.20 | 0.03 |
| VEGFR2 (Ab-1214) | 2603 | 2191 | 135 | 124 | 129.5 | 2496.95 | 2288.18 | 2392.57 | 147.62 | 0.06 |
| VEGFR2 (Ab-951) | 3541 | 2961 | 135 | 124 | 129.5 | 3396.74 | 3092.33 | 3244.54 | 215.24 | 0.07 |
| VEGFR2 (Phospho-Tyr1054) | 122 | 102 | 135 | 124 | 129.5 | 117.03 | 106.52 | 111.78 | 7.43 | 0.07 |
| VEGFR2 (Phospho-Tyr1059) | 174 | 165 | 135 | 124 | 129.5 | 166.91 | 172.32 | 169.61 | 3.82 | 0.02 |
| VEGFR2 (Phospho-Tyr1175) | 115 | 445 | 135 | 124 | 129.5 | 110.31 | 464.74 | 287.53 | 250.61 | 0.87 |
| VEGFR2 (Phospho-Tyr1214) | 157 | 226 | 135 | 124 | 129.5 | 150.60 | 236.02 | 193.31 | 60.40 | 0.31 |
| VEGFR2 (Phospho-Tyr951) | 200 | 218 | 135 | 124 | 129.5 | 191.85 | 227.67 | 209.76 | 25.33 | 0.12 |
| Vinculin (Ab-821) | 74 | 61 | 135 | 124 | 129.5 | 70.99 | 63.71 | 67.35 | 5.15 | 0.08 |
| Vinculin (Phospho-Tyr821) | 183 | 320 | 135 | 124 | 129.5 | 175.54 | 334.19 | 254.87 | 112.18 | 0.44 |
| WASP (Ab-290) | 227 | 114 | 135 | 124 | 129.5 | 217.75 | 119.06 | 168.40 | 69.79 | 0.41 |
| WASP (Phospho-Tyr290) | 94 | 256 | 135 | 124 | 129.5 | 90.17 | 267.35 | 178.76 | 125.29 | 0.70 |
| WAVE1 (Ab-125) | 89 | 74 | 135 | 124 | 129.5 | 85.37 | 77.28 | 81.33 | 5.72 | 0.07 |
| WAVE1 (Phospho-Tyr125) | 97 | 129 | 135 | 124 | 129.5 | 93.05 | 134.72 | 113.88 | 29.47 | 0.26 |
| WEE1 (Ab-53) | 110 | 107 | 135 | 124 | 129.5 | 105.52 | 111.75 | 108.63 | 4.40 | 0.04 |
| WEE1 (Phospho-Ser53) | 83 | 79 | 135 | 124 | 129.5 | 79.62 | 82.50 | 81.06 | 2.04 | 0.03 |
| WEE1 (Phospho-Ser642) | 135 | 168 | 135 | 124 | 129.5 | 129.50 | 175.45 | 152.48 | 32.49 | 0.21 |
| WWOX (Phospho-Tyr33) | 285 | 228 | 135 | 124 | 129.5 | 273.39 | 238.11 | 255.75 | 24.94 | 0.10 |
| XIAP (Ab-87) | 149 | 150 | 135 | 124 | 129.5 | 142.93 | 156.65 | 149.79 | 9.70 | 0.06 |
| XIAP (Phospho-Ser87) | 269 | 75 | 135 | 124 | 129.5 | 258.04 | 78.33 | 168.18 | 127.08 | 0.76 |
| Zap-70 (Ab-292) | 152 | 244 | 135 | 124 | 129.5 | 145.81 | 254.82 | 200.31 | 77.09 | 0.38 |
| Zap-70 (Ab-319) | 655 | 331 | 135 | 124 | 129.5 | 628.31 | 345.68 | 487.00 | 199.85 | 0.41 |
| Zap-70 (Ab-493) | 204 | 197 | 135 | 124 | 129.5 | 195.69 | 205.74 | 200.71 | 7.11 | 0.04 |
| Zap-70 (Phospho-Tyr292) | 83 | 73 | 135 | 124 | 129.5 | 79.62 | 76.24 | 77.93 | 2.39 | 0.03 |
| Zap-70 (Phospho-Tyr315) | 140 | 121 | 135 | 124 | 129.5 | 134.30 | 126.37 | 130.33 | 5.61 | 0.04 |
| Zap-70 (Phospho-Tyr319) | 174 | 163 | 135 | 124 | 129.5 | 166.91 | 170.23 | 168.57 | 2.35 | 0.01 |
| Zap-70 (Phospho-Tyr493) | 148 | 142 | 135 | 124 | 129.5 | 141.97 | 148.30 | 145.13 | 4.47 | 0.03 |
[truncated: 12,246 more chars]
